# Transcriptome and Behavioral Assessment in Larval Zebrafish (*Danio rerio*) Following Exposure to Perfluorononanoic Acid (PFNA)

**DOI:** 10.3390/genes17050558

**Published:** 2026-05-07

**Authors:** Lev Avidan, Cole D. English, Katie A. McDonnell, Emma Ivantsova, Christopher J. Martyniuk

**Affiliations:** 1 Center for Environmental and Human Toxicology, Department of Physiological Sciences, College of Veterinary Medicine, University of Florida, Gainesville, FL 32611, USA; lavidan@ufl.edu (L.A.); englishc@mit.edu (C.D.E.); katiemcdonnell@ufl.edu (K.A.M.); eivantsova@ufl.edu (E.I.); 2 Interdisciplinary Program in Biomedical Sciences Neuroscience, UF Genetics Institute, Gainesville, FL 32611, USA

**Keywords:** zebrafish, aquatic toxicology, neurotoxicity, transcriptomics

## Abstract

**Background/Objectives**: Per- and polyfluoroalkyl substances (PFAS) are environmentally persistent chemicals widely detected in aquatic systems and drinking water. Perfluorononanoic acid (PFNA), a long-chain PFAS, has been reported globally in environmental matrices and fish tissues. Although PFNA has been linked to developmental, metabolic, and neurological toxicity, its effects on lipid-related pathways and neurotoxicity remain poorly characterized. **Methods**: This study evaluated the developmental and neurotoxic effects of PFNA exposure in zebrafish embryos and larvae following a 7-day exposure to environmentally relevant PFNA concentrations. **Results**: PFNA exposure did not significantly affect survival or deformity rates at the concentrations tested. Apoptosis was significantly increased in larvae exposed to 1 µg/L PFNA compared to controls, whereas reactive oxygen species levels were unaffected. Two concentrations (0.1 µg/L and 10 µg/L) were further examined for transcriptomic responses, and the transcriptome response was largely different for each concentration. Low-dose PFNA exposure primarily affected lipid transport, cholesterol metabolism, sphingolipid signaling, and neurodegeneration-related pathways, whereas high-dose PFNA altered transcripts related to synaptic signaling, axon guidance, and thyroid hormone synthesis. Hypoactivity was observed in the movement of larval zebrafish based on a Visual Motor Response test. **Conclusions**: Taken together, PFNA exposure induced molecular changes related to neurotoxicity and lipid metabolism in zebrafish, which may contribute to adverse neurodevelopmental outcomes.

## 1. Introduction

Per- and Polyfluoroalkyl Substances (PFAS) are a novel group of synthetic chemicals that have been widely used in household and industrial goods. Most commonly, PFAS have a terminal carboxylic or sulfonic group, but are best characterized by their alkyl chains containing at least one wholly fluorinated carbon. PFAS are further classified based on their alkyl chains, in which polyfluoroalkyl substances contain at least one, non-fully fluorinated carbon and perfluoroalkyl substances contain a fully fluorinated alkyl chain. Furthermore, the dense carbon–fluorine bonds present on the alkyl backbone grant PFAS a high degree of thermal stability [1]. PFAS are commonly found in cleaning products as PFAS surfactants have been observed to decrease the surface tension of water by 80% [2]. Within the oil industry, PFAS surfactants are used to improve bedrock permeability, which increases rates of secondary oil recovery [3]. PFAS have also been observed to mask the release of toxic aerosols, which explain their usage in metal plating agents [4]. Due to their prevalence in industrial fields, PFAS have recently been found in approximately 45% percent of U.S. water supplies [5]. Internationally, Stefano et al. [6] reported that PFAS chemicals were present in 50% of groundwater and surface water supplies in Southern Brazil. Overall, many PFAS have been phased out of production due to their toxicity, bioaccumulation, and environmental persistence.

Perfluorononanoic acid (PFNA, C_9_HF_17_O_2_) is a long-chain PFAS that has been reported across environmental matrices (freshwater, groundwater, surface water, sediment, soil) worldwide, as well as in various fish tissue. PFNA concentrations across locations vary due to different factors, including emission sources, proximity to emission sources, and environmental conditions among others. For instance, 10,000 ng/L PFNA [7] was recorded in surface water in the United States but has been recorded as low as 0.36 ng/L [8] and 0.39 ng/L [9] in freshwater in Sweden and Canada, respectively. PFNA has also been observed in Georgia’s Consagua River at concentrations ranging from 12.3 to 456 ng/L [10]. Within U.S. Air Force bases, previous studies have identified PFNA to be present in the groundwater at 3 μg/L and 10 μg/L in the surface water [7]. Similar contamination has also been documented at commercial airports. Liu et al. [11] observed maximum surface-water concentrations of 3.41 ng/L PFNA at international airports in China and Koch et al. [8] detected levels of PFNA up to 0.36 ng/L in freshwater sites near Swedish airports. Other research has also identified PFNA in the waterways of large, urban areas—where higher population densities have been positively associated with elevated PFNA pollution levels [12]. For example, maximum concentrations of PFNA have been identified at 14 ng/L within surface rivers of the New York Metropolitan Area; additionally, the river basin of the Tokyo Metropolitan area has previously reported PFNA concentrations of 20.1 ng/L. Within Japan, yearly PFNA sewage emissions have also been estimated to total 2.6 tons per year [12].

Previous studies on zebrafish have documented adverse impacts of PFNA on general health. For examples, PFNA’s role in neurotoxicity has been documented, where PFNA was observed to reduce GABAergic neurons, as well as certain neurotransmitters such as acetylcholine, dopamine, and glutamate [13]. Additionally, Ulhaq et al. [14] reported how exposure to PFNA decreased locomotor activity in the dark periods of a locomotor assay, indicating neurotoxicity. Liu et al. [15] documented multi-generational thyroid-disrupting effects of PFNA on zebrafish, including elevated T3 hormone plasma levels and a dysregulation of gene expression relating to thyroid hormone synthesis. Previous studies have also elucidated a sex-dependent dysregulation of lipid metabolism. Zhang et al. [16] reported that cholesterol levels increased in both male and female zebrafish upon exposure to PFNA; the transcription of fatty acid-binding proteins (FABPs) were upregulated in male zebrafish livers, while downregulated in female zebrafish livers. Taken together, PFNA may have detrimental effects on neural and hormone systems as well as lipid regulation.

The objective of this study was to evaluate the potential toxicity of PFNA on cellular stress responses, behavior, and transcriptomic pathways in developing zebrafish embryos and larvae. We hypothesized that environmentally relevant concentrations (low µg/L) of PFNA would induce molecular and transcriptional alterations, particularly in pathways associated with neurodevelopment, neurotoxicity, lipid regulation, and cellular signaling. Data from this study is expected to provide insight into early-life stage PFNA toxicity and is expected to clarify how environmentally relevant exposures influence molecular and biological processes in aquatic organisms.

## 2. Materials and Methods

### 2.1. Chemical Preparation 

PFNA was purchased from Sigma-Aldrich (CAS no. 375-95-1, purity 97%). Stock solutions of 0.1 µg/L, 1 µg/L, 10 µg/L, 100 µg/L, and 1000 µg/L PFNA were prepared in 0.1% dimethyl sulfoxide (DMSO, CAS 67-68-5, Sigma-Aldrich, Inc., St. Louis, MO, USA) and added to embryo rearing media (ERM) containing the zebrafish embryos. Recipes for ERM were obtained from Westerfield [17] and reported in full in [18]. Exposure solutions were prepared daily to yield final concentrations of 0.1, 1, 10, 100, and 1000 µg/L PFNA. These concentrations presented a broad range to investigate biological effects in zebrafish.

### 2.2. Husbandry and Egg Production of Zebrafish

Adult zebrafish (AB × Tübingen, *Danio rerio*, 6 months of age) were raised in a flow-through Pentair system in the Cancer-Genetics Research Center at the University of Florida and are derived from an ongoing zebrafish breeding colony. Environmental conditions for zebrafish breeding have been previously described [19,20] and are detailed in the Supplemental Methods. Staging of embryos followed that of Kimmel et al. [21]. Fish were fed Zeigler Adult Zebrafish Diet. Fish are maintained at a temperature of 27 ± 1 °C, air saturation at 82%, water pH between 7.2 and 7.3, conductivity of 1500–1600 μS/cm, and 14:10 h light/dark cycle. Institutional Animal Care and Use Committee of University of Florida approved all experiments (UF IACUC202300000140).

### 2.3. PFNA Exposure Regime

The embryonic acute toxicity test followed recommendations from the OECD guideline 236 with modification [22]. Several independent experiments were conducted. For each experiment, approximately 20 fertilized embryos were randomly assigned to 25 mL Pyrex glass beakers with 11 mL sterile embryo rearing media (ERM) with designated concentrations spanning environmentally relevant values from 0.1 µg/L to 1000 µg/L PFNA. Fish were subjected to a cycle of 14 h light and 10 h darkness at 27 ± 1 °C. On each day of the 7-day experiment, dead embryos/larvae were removed, and an 80% water change was conducted with chemical dilutions that were made fresh daily. Either a Keyence All-in-One Fluorescence Microscope BZ-X710 or EVOS^TM^ FL Auto Imaging System (Thermo Scientific, Waltham, MA, USA) were used to collect images of the embryos to document the mortality, hatch rate, and deformities. Four fish per beaker with 6 beakers from each treatment group (*n* = 24 per treatment group) were evaluated. Several replicate experiments (*n* = 6) were required to obtain sufficient larvae for all toxicity assays and sub-lethal endpoints investigated in this study. Tricaine mesylate (Syncaine, Tricaine-S, Ferndale, WA, USA) was used for euthanasia at 250 mg/L buffered with equal part sodium bicarbonate to a pH between 7.0 and 7.5. 

### 2.4. Acridine Orange

The acridine orange (AO) staining method was conducted to detect apoptotic cells in zebrafish larvae. To avoid overlapping with published studies, the complete method is presented in Supplemental Methods.

### 2.5. Reactive Oxygen Species

Embryos were obtained for reactive oxygen species (ROS) assessment as outlined above in Section 2.2. To avoid overlapping with published studies, the complete method is presented in Supplemental Methods.

### 2.6. RNA-Seq

Samples were collected following the 7-day exposure as outlined in Section 2.3 for RNA-seq. Each beaker of fish containing 14–15 individuals were pooled to generate a biological replicate. Total RNA was extracted using TRIzol (Thermo Fisher Scientific, Waltham, MA, USA). The concentration was determined using the Qubit^®^ 2.0 Fluorometer (Thermo Fisher, Grand Island, NY, USA). A total of 12 RNA samples were used for RNA-seq library construction (RIN > 7) (Agilent 2100 Bioanalyzer, Agilent Technologies, Inc.). Biological replicates were as follows: 0.1% DMSO controls (*n* = 3), 0.1 μg/L PFNA (*n* = 3), and 10 μg/L PFNA (*n* = 3).

Total RNA was used for mRNA isolation using the NEBNext Ploy(A) mRNA Magnetic Isolation module (New England Biolabs, Ipswich, MA, USA, catalog # E7490). RNA library construction was then performed with the NEBNext^®^ Ultra™ II Directional RNA Library Prep Kit for Illumina^®^ (New England Biolabs, catalog #E7760) according to the manufacturer’s user guide. Individually prepared libraries were pooled by equimolar and sequenced by GeneWiz (Azenta Life Sciences, Burlington, MA, USA).

Sequence reads were trimmed to remove possible adapter sequences and nucleotides with poor quality using Trimmomatic v.0.36. The trimmed reads were mapped to the *D. rerio* GRCz10.89 reference genome available on ENSEMBL using the STAR aligner v.2.5.2b and BAM files were generated. Statistics of mapping the reads to the reference genome. Unique gene hit counts were calculated by using featureCounts from the Subread package v.1.5.2. The hit counts were summarized and reported using the gene_id feature in the annotation file. Only unique reads that fell within exon regions were counted. If a strand-specific library preparation was performed, the reads were strand-specifically counted. Distribution of read counts in libraries were examined before and after normalization. The original read counts were normalized to adjust for various factors such as variations in sequencing yield between samples. These normalized read counts were used to accurately determine differentially expressed genes (Median of Ratios Method default in DESeq2) (version 1.51.6). After extraction of normalized gene hit counts, the gene hit counts table was used for downstream differential expression analysis. Using DESeq2, a comparison of gene expression between each treatment and the control group was performed. The Wald test was used to generate *p*-values and log2 fold changes. Genes with an adjusted *p*-value < 0.05 and absolute log2 fold change > 1 were called as differentially expressed genes for each comparison. Excel files containing read count, fpkm, log2 fold change, *p*-value are provided in Supplemental Data files. All raw and processed transcriptome data have been deposited in the NCBI Gene Expression Omnibus (GEO) database (GSE324353).

### 2.7. Bioinformatics

Volcano plots were used to reveal significant transcripts with high log2 expression values. Functional analysis (gene ontology) was conducted for each concentration of PFNA (low, 0.1 μg/L; high, 10 μg/L) in iPathway. Functional analysis version 18.1 [Molecular Signature analysis/Gene Ontology Term Enrichment analysis] was done using iPathwayGuideTM (AdvaitaBio Corporation, Ann Arbor, MI, USA, https://ipathwayguide.advaitabio.com/, accessed on 5 March 2026). The Chord Diagrams and Pathway Images were generated using iPathwayGuideTM (AdvaitaBio Corporation, https://ipathwayguide.advaitabio.com/, accessed on 5 March 2026) [23,24]. Analyses utilized pathways from the KEGG (Release 100.0+/11–12 November 2021) and gene ontologies from the Gene Ontology Consortium (4 November 2021). Enrichment analyses incorporated miRNA data from miRBase (v22.1, October 2018) and TargetScan (Mouse v8.0, Human v8.0), regulatory networks from BioGRID (v4.4.203, October 2021), and toxicant and disease associations from the Comparative Toxicogenomics Database and KEGG databases. Compete reports outlining methodology are provided in Supplemental Files.

### 2.8. Real-Time PCR Analysis

To conduct real-time PCR assays, a double batch of fish were bred as per Section 2.2. This breeding event included 2 different sets of parents and yielded over 320 eggs. After the toxicity assay, a subset of fish was flash frozen in liquid nitrogen. Samples were then stored at −80 °C for RNA extraction and real-time PCR analysis (0.1% DMSO, 0.1 μg/L, 1 μg/L, and 10 μg/L µg/L; *n* = 3 to 8 biological replicates per group). Biological replicates varied due to balancing replicates (beakers), concentrations, and larval fish numbers. Fish were treated for 7 days to PFNA prior to sample collection. Real-time PCR followed our established protocols [23]. Samples were run in duplicate and followed RT-qPCR cycling parameters described by us [24]. To avoid overlapping with published studies, the complete method is presented in Supplemental Methods.

Primers used in this study were obtained from the published literature [24,25,26,27,28,29,30,31] and are listed in Supplemental Table S1. The transcripts measured in this study included acetylcholinesterase (*ache*), BCL2-associated X protein (*bax*), B-cell Lymphoma 2 (*bcl2*), Caspase-3 (*casp3*), Catalase (*cat*), ELAV-like RNA-binding protein 3 (elavl3), ELOVL Fatty Acid Elongase 6 (*elovl6*), Growth Associated Protein 43 (*gap43*), Glial Fibrillary Acidic Protein (*gfap*), Heme Oxygenase 1 (*hmox1*), Heat Shock Protein Family A Member 4 (*hspa4*), Mesencephalic Astrocyte-Derived Neurotrophic Factor (*manf*), Microtubule-Associated Protein Tau A (*maptA*), Microtubule-Associated Protein Tau B (*maptB*), Myelin Basic Protein (*mbp*), Mitochondrially Encoded Cytochrome C Oxidase I (*mt-co1*), Nestin (*nes*), Nuclear factor erythroid 2-related factor 2, ((*nrf2*), NAD(P)H Quinone Dehydrogenase 1 (*nqo1*), Tumor Protein p53 (*p53*), Sonic Hedgehog Signaling Molecule (*shh*), Superoxide Dismutase 1 (*sod1*), Superoxide Dismutase 2 (*sod2*), and α1-tubulin (*tub1a1*). Beta-Actin (*bactin*) and Ribosomal Protein S18 (*rps18*) were used to normalize expression levels of all target genes using the CFX Manager (v3.1) software. Normalized expression was obtained for each target gene using CFX Manager™ software (v3.1) (baseline subtracted) and the Cq method was employed. Assessing oxidative stress can be conducted at the level of the transcriptome (antioxidant responses) as well as directly measuring ROS levels. In our previous studies, we have observed no change in ROS levels but an increase in antioxidant gene expression, suggesting that increased gene expression may reflect lower ROS levels following higher exposure concentrations of chemicals. Thus, despite not observing a change in ROS, we also investigated transcript levels for antioxidant genes.

### 2.9. Visual Motor Response Test

The Visual Motor Response (VMR) test was employed following PFNA exposure to test for differences in locomotor activity. Zebrafish embryos were collected for the VMR test after several toxicity tests with embryos. The experimental groups included 0.1% DMSO, ERM, 1 µg/L, 10 µg/L, 100 µg/L, and 1000 µg/L PFNA. Multiple replicate beakers were used for each toxicity experiment (*n* = 5–10 in each experiment). The VMR test proceeded as per our established methods [19,23,32]. Total distance moved was used as an indicator of overall locomotor activity. To avoid overlapping with published studies, the complete method is presented in Supplemental Methods.

### 2.10. Statistical Analysis

Statistical analysis and graphing were conducted using GraphPad v9.5.1 (La Jolla, CA, USA). Data were first assessed for normality using a Shapiro–Wilk test and gene expression data were log(10) transformed to approximate a normal distribution. Survival was analyzed with a Kaplan–Meier test (log-rank Mantel–Cox test). Statistical hypothesis testing was not employed to analyze deformity and hatch data as deformity frequency was low (<2%) in all treatment groups. Apoptosis, reactive oxygen species [relative fluorescence units (µg/mL protein)], gene expression levels, and the VMR for larval zebrafish were analyzed using One-Way ANOVA, followed by a Dunnett’s multiple comparisons test to the control group (mean ± SD). Significance of difference was determined to be *p* < 0.05.

## 3. Results

### 3.1. Survival and Deformity

**Figure 1:**
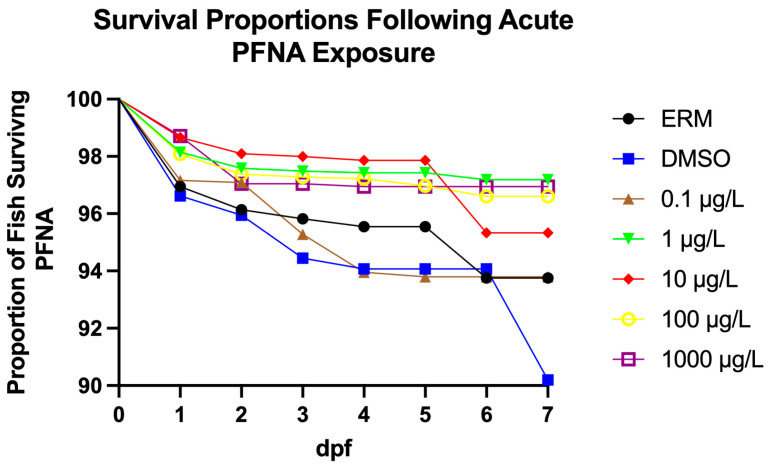
Proportion of surviving zebrafish embryos/larvae following exposure to either ERM, 0.1% DMSO, 0.1, 1, 10, 100, or 1000 µg/L PFNA for 7 days. Data are presented as mean (±SD). Error bars are small and are within the symbol.

**Figure 2:**
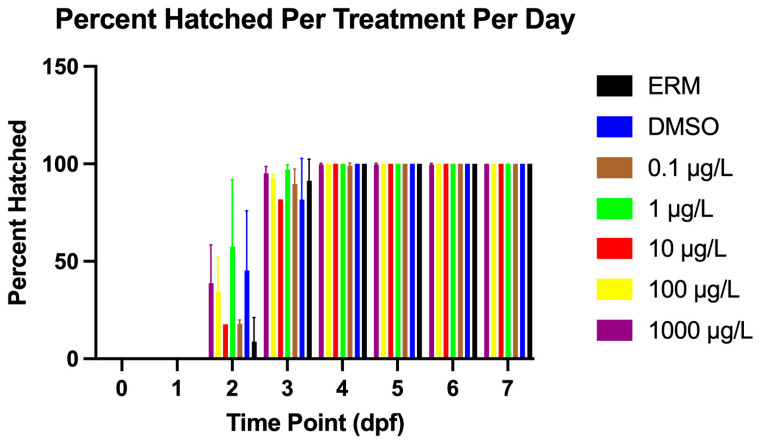
Total percentage of hatched zebrafish embryos/larvae following exposure to either ERM, 0.1% DMSO, 0.1, 1, 10, 100, or 1000 µg/L PFNA for 7 days. Data are presented as mean (±SD). Lack of standard error bars for some treatments indicates no variability in the replicates.

A Log-rank (Mantel–Cox) test revealed differences among groups for survival over time (Chi-square = 69.88, d.f. = 5, *p* value < 0.0001). Relative to the ERM and the DMSO solvent group, zebrafish larvae exposed to PNFA up to 1 mg/L showed higher survival (Figure 1). Thus, PFNA did not reduce survival relative to DMSO. We point out that in all treatments, survival was above 95%, even with the highest concentration tested. Taken together, there was a slight increase in survival with PFNA treatment relative to DMSO. Percent hatch was unaffected by PFNA exposure (Figure 2).

**Figure 3:**
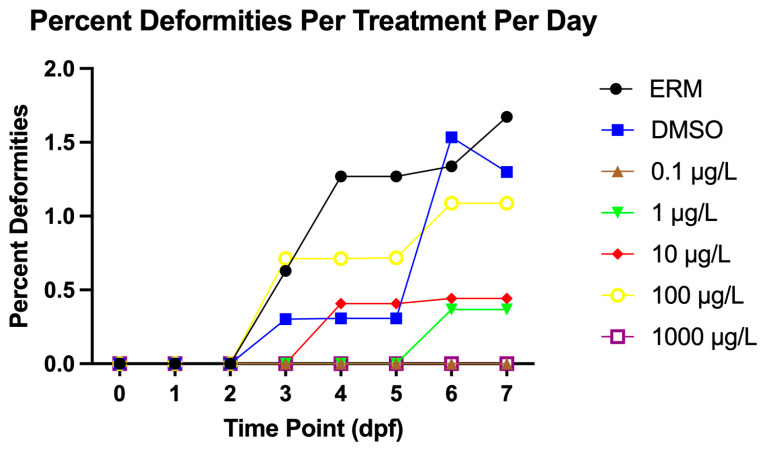
Percent of deformities of zebrafish embryos/larvae exposed to either ERM, 0.1% DMSO, 0.1, 1, 10, 100, or 1000 µg/L PFNA for 7 days.

All experimental groups also showed a low frequency of deformities (<2%) (Figure 3) marked by a few fish presenting with cardiac edema, spinal lordosis, and stage delay. Axial malformations such as kyphosis and caudal tail curvature were also noted in some of these embryos. Taken together, PFNA did not induce significant deformities up to 1000 µg/L.

### 3.2. Acridine Orange

**Figure 4:**
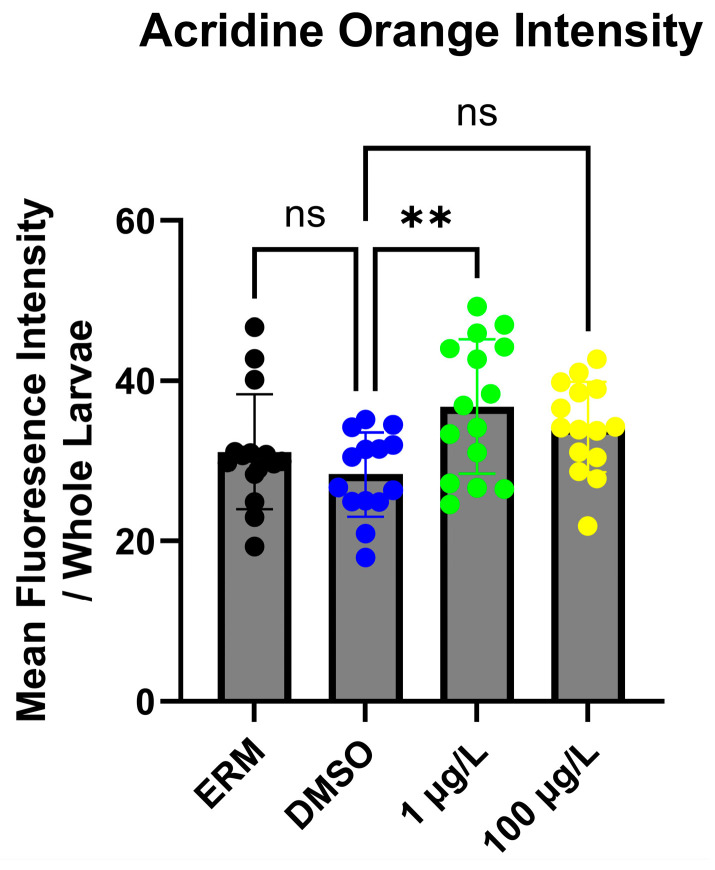
Mean fluorescence intensity in zebrafish larvae exposed to either ERM, 0.1% DMSO, 1, or 100 µg/L PFNA at 7 dpf. Data represented as the mean value of the group (±SD) (One-Way ANOVA followed by Dunnett’s post hoc test, *n* = 50/group). Asterisks denote significant differences across a group and ERM control during an interval (** *p* < 0.01). ns = not significant.

The effect of PFNA on zebrafish larvae apoptosis was assessed at 7 dpf. There was a significant difference noted between the 0.1% DMSO control group and fish exposed to 1 µg/L PFNA (F_(3,55)_ = 4.325; *p* = 0.0083) (Figure 4). 

### 3.3. Reactive Oxygen Species

**Figure 5:**
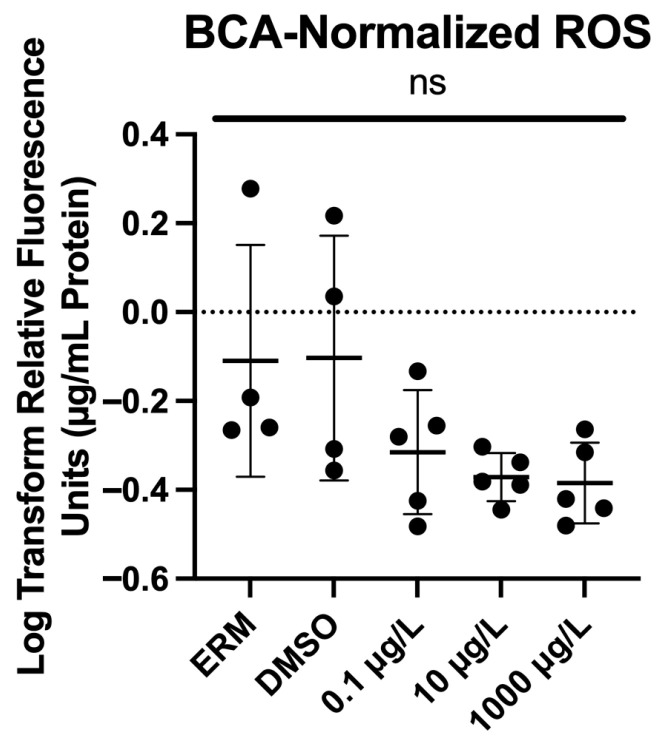
Normalized levels of reactive oxygen species (to μg/mL media/protein). Each circle represents a biological replicate; the mean (±S.D.) is represented by the horizontal line (One-Way ANOVA, Dunnett’s multiple comparisons test, *n* = 4 or 5 biological replicates/experimental group). ns = not significant.

ROS levels were unchanged at the concentrations tested and time point investigated (F_(4,18)_ = 1.883; *p* = 0.157) (Figure 5).

### 3.4. RNA-Seq Analysis

The transcriptomes obtained were of overall high quality and quality control metrics, mapping, and read counts are presented in Supplemental Data S1 and QC reports are also provided. Differential gene expression analysis identified transcripts altered in larvae exposed to 0.1 or 10 μg/L PFNA (FDR *p*-value). Transcripts differentially expressed in larval zebrafish exposed to 0.1 μg/L PFNA totaled 270 (*p* < 0.05), but no gene passed an FDR correction. Thus, any gene enrichment analysis must be interpreted with caution due to a very subtle effect on the transcriptome. However, the transcriptome response was more robust for larval fish exposed to 10 μg/L PFNA and differentially expressed genes totaled 2424 (*p* < 0.05) and 232 (*p*-value adjusted < 0.05) (Supplemental Data S1).

**Figure 6:**
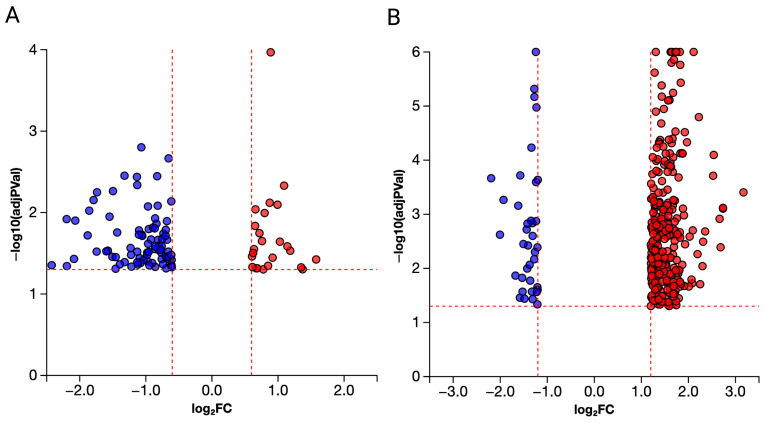
Volcano plots demonstrate differentially expressed genes in blue (downregulated) or red (upregulated) for low ((**A**), 0.1 μg/L) and high ((**B**), 10 μg/L) PFNA exposure in larval zebrafish. These are transcripts that showed a threshold of 0.05 for statistical significance (*p*-value) and a log fold change in expression with absolute value of at least 0.6 (criteria indicated by red, dotted line).

Low-dose PFNA exposure primarily affected lipid transport, cholesterol metabolism, sphingolipid signaling, and neurodegeneration-related pathways, whereas high-dose PFNA altered transcripts related to synaptic signaling, axon guidance, and thyroid hormone synthesis. Table 1 presents the top 10 genes impacted following exposure to 0.1 µg/L and 10 µg/L PFNA based on *p*-value alone. Volcano plots of each of the two concentrations depict differentially expressed genes relative to their fold changes, with red indicating upregulation and blue indicating downregulation (Figure 6) (*p* < 0.05). Genes depicted in the volcano plots were identified using thresholds defined by us. To ensure higher rigor of data, we chose a threshold of 0.05 for statistical significance (*p*-value) and a log fold change in expression with absolute value of at least 0.6. The Supplemental Data presents all differentially expressed transcripts in larval zebrafish following PFNA exposure.

**Figure 7:**
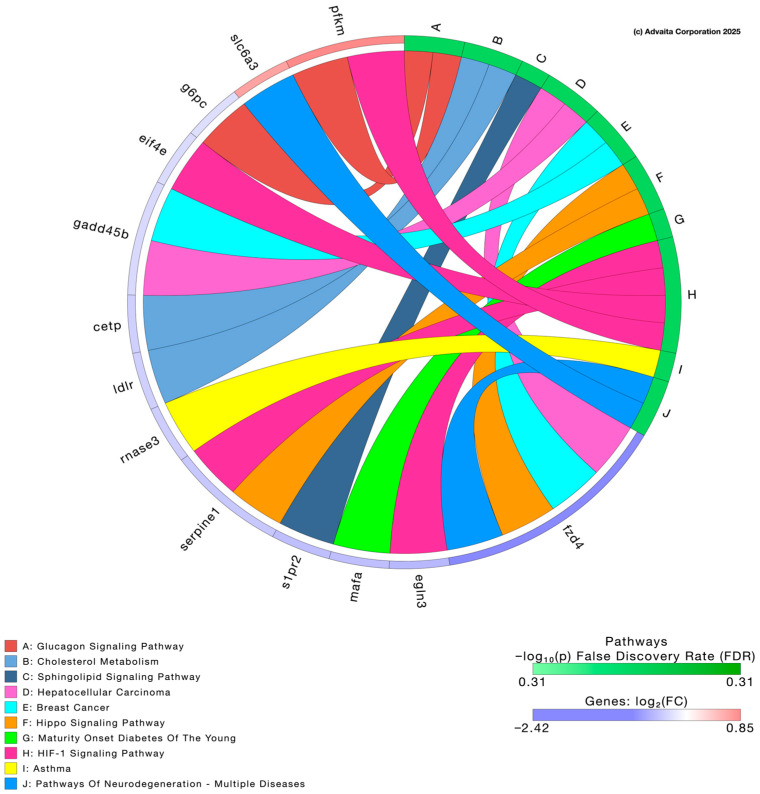
Chord diagram depicting associations between differentially expressed genes and significantly impacted pathways following 0.1 µg/L PFNA exposure.

**Figure 8:**
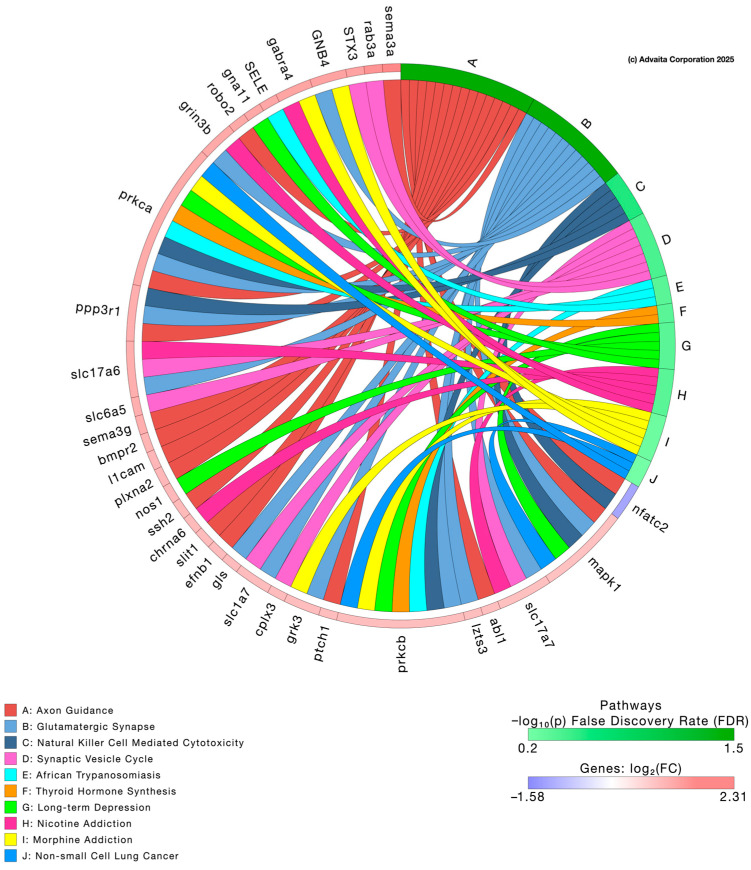
Chord diagram depicting associations between differentially expressed genes and significantly impacted pathways following 10 µg/L PFNA exposure.

Pathway enrichment analyses were conducted using iPathway software version 18.1 (Advaita Corporation, Ann Arbor, MI, USA) to identify biological processes and hallmark signatures related to PFNA exposure. Low-dose PFNA exposure primarily affected lipid transport, cholesterol metabolism, sphingolipid signaling, and neurodegeneration-related pathways, whereas high-dose PFNA altered transcripts related to synaptic signaling, axon guidance, and thyroid hormone synthesis. The analysis revealed a significant number of pathways perturbed by PFNA exposure depicted in the chord diagrams. Pathways impacted by low-dose PFNA (Figure 7) include cholesterol metabolism, sphingolipid signaling pathway, and pathways of neurodegeneration among others whereas high-dose PFNA impacted axon guidance, glutamatergic synapse, and thyroid hormone synthesis among others (Figure 8). Biological processes impacted by low-dose PFNA (Supplemental Figure S1) include nervous system process, lipid transport, and lipid localization among others whereas high-dose PFNA impacted nervous system development, cell communication, and synaptic signaling among others (Supplemental Figure S2). Lastly, enriched hallmark gene signatures by low-dose PFNA (Supplemental Figure S3) include TNFA signaling via NF-κB, cholesterol homeostasis, and IL-2 STAT5 signaling among others whereas high-dose PFNA impacted adipogenesis, mitotic spindle, and hedgehog signaling among others (Supplemental Figure S4). Diseases and clinical parameters associated with PFNA exposure are also reported in the Supplemental Data document for interested readers; however, we focus our attention on biological processes and pathways.

### 3.5. Real-Time PCR Analysis

**Figure 9:**
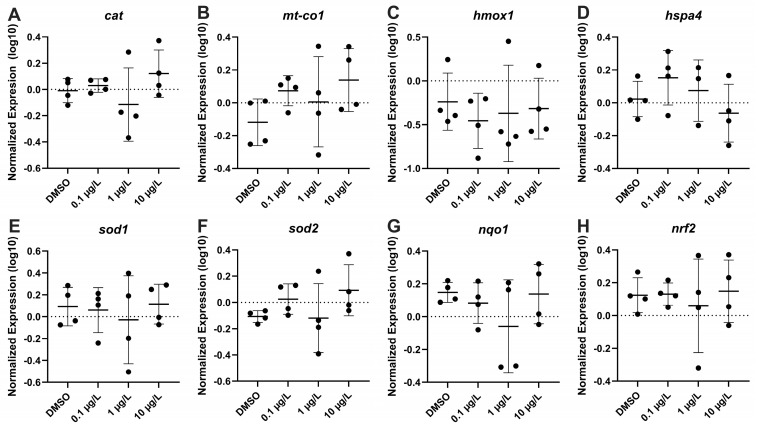
Relative oxidative stress gene expression in zebrafish larvae exposed to solvent control (0.1% DMSO), 0.1 µg/L, 1 µg/L, or 10 µg/L PFNA. (**A**) *cat*, (**B**) *mt-co1*, (**C**) *hmox1*, (**D**) *hspa4*, (**E**) *sod1*, (**F**) *sod2*, (**G**) *nqo1*, and (**H**) *nrf2.* The mean (±standard deviation) is represented by the horizontal line. Data were evaluated using One-Way ANOVA, followed by a Dunnett’s multiple comparisons test to the control group (mean ± SD), significance determined at *p* < 0.05.

**Figure 10:**
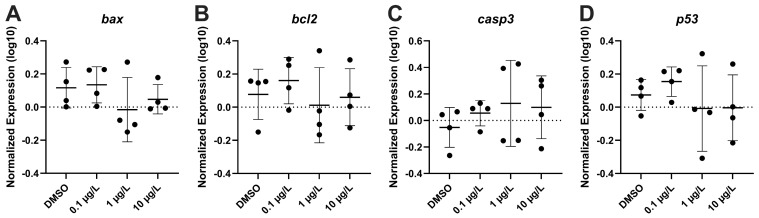
Relative apoptotic gene expression in zebrafish larvae exposed to solvent control (0.1% DMSO), 0.1 µg/L, 1 µg/L, or 10 µg/L PFNA. (**A**) *bax*, (**B**) *bcl2*, (**C**) *casp3*, and (**D**) *p53*. The mean (±standard deviation) is represented by the horizontal line. Data were evaluated using One-Way ANOVA, followed by a Dunnett’s multiple comparisons test to the control group (mean ± SD), significance determined at *p* < 0.05.

**Figure 11:**
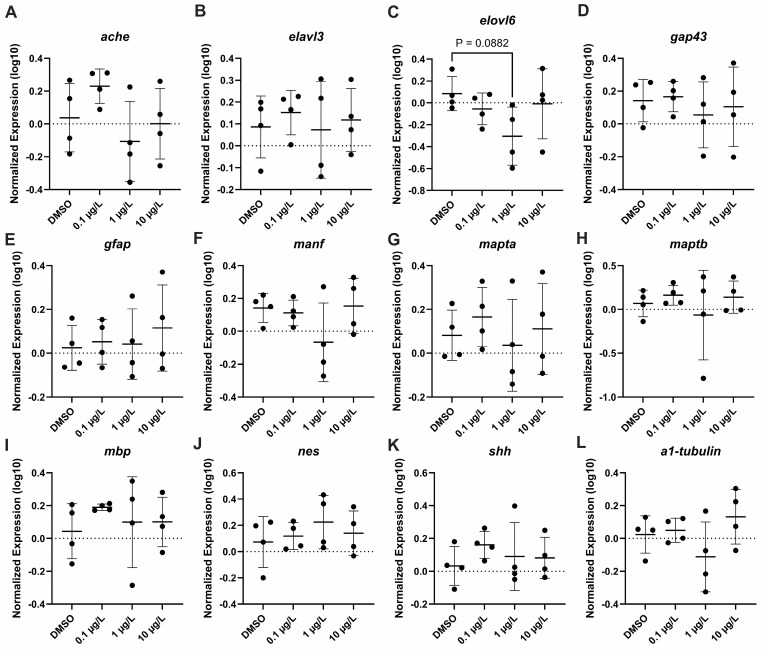
Relative neurotoxic gene expression in zebrafish larvae exposed to solvent control (0.1% DMSO), 0.1 µg/L, 1 µg/L, or 10 µg/L PFNA. (**A**) *ache*, (**B**) *elovl3*, (**C**) *elovl6*, (**D**) *gap43*, (**E**) *gfap*, (**F**) *manf*, (**G**) *mapta*, (**H**) *maptb*, (**I**) *mbp*, (**J**) *nes*, (**K**) *shh*, and (**L**) *a1-tubulin*. Data are represented as mean ± standard deviation. Data were evaluated using One-Way ANOVA, followed by a Dunnett’s multiple comparisons test to the control group (mean ± SD), significance determined at *p* < 0.05.

The effect of PFNA on the mRNA steady state levels of transcripts related to oxidative damage response (*cat*, *mt-co1*, *hmox1*, *hspa4*, *sod1*, *sod2*, *nqo1*, *nfe2l2*) were measured, and no significant difference in transcription levels were recorded (Figure 9). There were also no significant changes observed in apoptosis transcripts (*bax*, *bcl2*, *casp3*, *tp53*) for all tested concentrations (Figure 10). Several transcripts relating to apoptosis (*ache*, *elovl3*, *elovl6*, *gap43*, *gfap*, *manf*, *mapta*, *maptb*, *mbp*, *nes*, *shh*, *tubb3*) were evaluated, but only *elovl6* was downregulated at 1 µg/L PFNA (F_(3,12)_ = 2.028, *p* = 0.088) (Figure 11).

### 3.6. Locomotor Activity Based upon the Visual Motor Response Test

**Figure 12:**
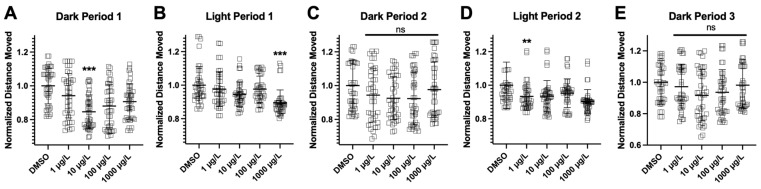
Visual Motor Response (VMR) test for PFNA-exposed zebrafish larvae. Light–dark cycles are organized in 10 min intervals. (**A**) Normalized mean (±SD) distance moved (mm) per minute intervals in dark period 1, (**B**) light period 1, (**C**) dark period 2, (**D**) light period 2, and (**E**) dark period 3. Asterisks denote significant difference across a group and DMSO control during an interval (** *p* < 0.01, *** *p* < 0.001) and ns = not significant (One-Way ANOVA, Dunnett’s multiple comparisons test, *n* = 16 per treatment in each of the 3 independent experiments).

The DanioVision™ (Noldus Information Technology, Leesburg, VA, USA) was used for the VMR test. Three independent trials were conducted with different batches of fish. Concentrations of 0.1% DMSO, 1 µg/L, 10 µg/L, 100 µg/L, and 1000 µg/L PFNA were investigated. Zebrafish larvae showed variability in distance traveled (Figure 12). In the first dark period, zebrafish larvae showed hypoactivity with exposure to 10 µg/L PFNA (*p* < 0.001). In the first light period, zebrafish larvae showed hypoactivity with exposure to 1000 µg/L PFNA (*p* < 0.001). In the second light period, zebrafish larvae showed hypoactivity with exposure to 1 µg/L PFNA (*p* < 0.001). Overall, PFNA decreased locomotor activity of larval fish, but this effect was not consistent with the concentration of PFNA. Overall, there was evidence for hypoactivity in larval fish, but this response was temporal and dose dependent.

## 4. Discussion

Developmental toxicity associated with PFNA exposure on zebrafish has been evaluated across a range of concentrations. We observed no detrimental effects of PFNA exposure on survival nor hatch rate up to 1 mg/L, consistent with previous studies. Jantzen et al. [33] exposed embryonic zebrafish to 9281.6 ng/L up to 0.928 mg/L PFNA and reported no significant acute mortality nor developmental delays. Another study documented mortality in zebrafish exposed to 34.7 mg/L PFNA until 120 hpf [34]. Another study observed a concentration-dependent decrease in hatching rate following exposure to 25–400 µM (11.6–185.6 mg/L) PFNA for 96 h [35], much higher than the current investigation. Overall, acute toxicity was not observed for PFNA up to 1 mg/L in this study.

ROS production is a well-documented response to chemical stressors in aquatic species. We detected no change in ROS levels when zebrafish were exposed to a range of PFNA concentrations (0.1 up to 1000 µg/L PFNA). This was also associated with no changes in basal expression of antioxidant genes (i.e., *cat*, *sod1*, *sod2* and others), corroborating a lack of evidence for oxidative stress. Previous studies have reported oxidative stress in zebrafish at substantially higher PFNA concentrations. For instance, exposure to 0.2 up to 15 mg/L PFNA for 140 h increased SOD activity at intermediate concentrations, while the highest exposure reduced both SOD and catalase activity and elevated malondialdehyde, indicating lipid peroxidation [36]. This exposure concentration was approximately 15x higher than the highest concentration tested in this study. Similarly, zebrafish embryos exposed to PFNA levels ranging 1–100 mg/L showed induction of antioxidant enzymes (i.e., glutathione S-transferase, heat shock protein 70) [37]. In the study, lower exposures ranging from 0.0464 up to 4.64 mg/L altered ROS levels, inhibited glutathione, and increased nitric oxide production [38]. Overall, these findings suggest that oxidative stress can be a response observed in zebrafish following exposure to PFNA. A lack of response in our study may be related to the lower concentrations tested.

Zebrafish are utilized as models for neurotoxicity research for various reasons including their conserved neurotransmitter systems to mammals, observable neurodevelopment, and measurable behavioral outcomes. In our study, transcriptome analysis revealed potential mechanisms of PFNA-induced neurotoxicity. Low-dose exposure mainly affected processes related to neuron function and membrane biology (i.e., lipid transport and cholesterol metabolism), suggesting disruptions in neurotransmission during zebrafish development. As lipids like cholesterol and sphingolipids are essential for synaptic function, these changes may impair neuronal communication before structural damage occurs [39]. In contrast, high-dose PFNA exposure caused altered nervous system development, synaptic signaling, glutamatergic signaling, thyroid hormone synthesis, and axon guidance pathways, suggesting stronger neurotoxic effects that may disrupt neural network formation, excitatory signaling, and endocrine-driven brain development.

Other zebrafish endpoints further substantiate the neurotoxic effects of PFNA exposure. For example, Liu et al. [28] exposed zebrafish embryos up to 100 µg/L PFNA until 120 hpf and reported various impacts on the nervous system. Regarding neurons in the olfactory bulb, numbers were significantly decreased with all treatments in which 10 and 100 µg/L PFNA caused a decrease of 46.86% and 57.85%, respectively, at 96 hpf and 32.26% and 42.86%, respectively, at 120 hpf.

It is noteworthy that our study detected glutamatergic synapse and synaptic vesicle cycle pathway enrichment based on transcriptome analysis following 10 µg/L PFNA exposure. This also suggests compromised excitatory neurotransmission, which may reduce motor output during the dark phase, the phase in which zebrafish typically display heightened activity. Enrichment of axon guidance pathways implies potential alterations in neural connectivity, which may further contribute to impaired locomotor coordination leading to hypoactivity. Taken together, behavioral disruptions align with the enrichment of multiple pathways observed in our chord diagrams (i.e., nervous system processes, synaptic signaling, glutamatergic synapse, and axon guidance), supporting the hypothesis that PFNA exposure can disrupt neurodevelopmental processes, leading to transient or persistent behavioral deficits.

Behavioral alterations reported across zebrafish studies provide support for the neurotoxic pathways identified in the transcriptome response. Jantzen et al. [33] observed decreased swimming velocity following embryonic zebrafish exposure to 9281.6 ng/L, 92,816 ng/L, and 0.928 mg/L PFNA, with mid-level exposure (92,816 ng/L) also increasing distance traveled and time spent in the center of the tank, indicative of stress-related behavior. Similarly, Menger et al. [40] reported disrupted locomotor patterns following PFNA exposure ranging from 92.8 to 185.6 mg/L, where with 49 µM (22.7 mg/L) PFNA reducing swimming distance during the light phase and higher concentrations (6.96 and 22.73 mg/L) increasing burst activity during the dark phase. Phase-dependent hypo- and hyperactivity were further reported by Rericha et al. [35], who found hypoactivity during the dark phase following exposure to 16.24 mg/L PFNA and hypoactivity (1.16 and 16.24 mg/L) and hyperactivity (0.464 and 34.66 mg/L) during the light phase. Lastly, consistent with impaired sensory responsiveness, Lee et al. [41] reported that 450 µM (208.8 mg/L) PFNA increased distance traveled during the light phase while decreasing activity during the dark phase. These studies underscore the complex behavioral responses typically observed with PFNA and corroborate our study which did not detect any evidence for linear dose responses. There are many instances where there is a lack of dose-dependent effects with perfluorinated chemicals, and at relatively low levels (ng/L–µg/L LC_50_ values for PFNA in zebrafish), data can be concentration-dependent without a linear response curve. Several well-documented features of PFAS biology can make their dose response curves flat, U-shaped, or otherwise non-traditional [42,43] due to non-linear internal kinetics, early onset of effects, multiple mechanisms with overlapping thresholds, and chain-length-specific toxicity patterns.

We identified lipid-related processes as biological processes associated with PFNA exposure. These findings are supported by previous studies highlighting PFNA’s effects on lipid metabolism in zebrafish. Qian et al. [44] exposed zebrafish embryos to 100 µM PFNA until 96 hpf and found increased lipid accumulation following exposure. Additionally, KEGG analysis revealed that the most significantly affected pathway by PFNA was the PPAR signaling pathway, followed by fatty acid metabolism, steroid biosynthesis, and apoptotic. Regarding differentially expressed genes, both the PPAR and apoptotic pathway had the highest number of differential genes. Similarly, Liu et al. [34] exposed zebrafish embryos up to 400 µmol/L PFNA until 96 hpf and reported altered gene expression involved with lipid metabolism and oxidative stress. Specifically, *lfabp* (liver fatty acid-binding protein) and *ucp2* (uncoupling protein 2), both involved in lipid handling and mitochondrial function, were upregulated while *gpx1* (glutathione peroxidase 1), *sod1, mt-atp6* (mitochondrial ATP synthase subunit 6), and *mt-nd1* (mitochondrial NADH dehydrogenase subunit 1) were downregulated.

Our findings demonstrate that PFNA exposure at environmentally relevant concentrations does not induce significant developmental toxicity nor oxidative stress in zebrafish, as evidenced by unaltered survival, hatch rate, and ROS production. However, transcriptomic and behavioral analyses revealed dose-dependent alterations, suggestive of neurodevelopmental and behavioral effects, with low-dose exposures primarily disrupting lipid- and membrane-related neuronal processes and higher doses affecting nervous system development, synaptic signaling, glutamatergic pathways, and axon guidance. These molecular disruptions are supported by altered locomotor behavior and align with previous studies reporting PFNA-induced changes in neurotransmitter systems and activity patterns in zebrafish. Taken together, PFNA exposure leads to molecular changes related to neurotoxicity and lipid metabolism in zebrafish, which may contribute to adverse neurodevelopmental outcomes.

## Figures and Tables

**Table 1:** Top 10 genes impacted following exposure to 0.1 µg/L and 10 µg/L PFNA. All measured transcripts are presented in the Supplemental Data files. *p*adj = *p*-value adjusted.

Concentration	Gene Symbol	Gene Name	log_2_FoldChange	*p*-Value	*p*adj
0.1 µg/L	*si:dkey-57a22.15*	Unknown	0.931	6.082 × 10^−6^	1.17 × 10^−1^
	*BX248082.1*	Unknown	−1.622	5.317 × 10^−5^	5.12 × 10^−1^
	*bfsp2*	Beaded Filament Structural Protein 2	0.889	1.076 × 10^−4^	6.90 × 10^−1^
	*elf3*	E74 Like ETS Transcription Factor 3	−1.068	1.579 × 10^−3^	1.00
	*si:ch211-264e16.1*	Unknown	−2.418	1.888 × 10^−3^	1.00
	*ier2a*	Immediate early response protein 2	−0.656	2.160 × 10^−3^	1.00
	*sb:cb1058*	Unknown	−1.201	2.526 × 10^−3^	1.00
	*mafa*	v-maf musculoaponeurotic fibrosarcoma oncogene homolog	−1.323	3.526 × 10^−3^	1.00
	*hspb11*	Heat Shock Protein Family B (Small), Member 11	−0.829	3.577 × 10^−3^	1.00
	*bahcc1a*	BAH Domain and Coiled-Coil Containing 1	−1.135	3.649 × 10^−3^	1.00
10 µg/L	*CABZ01092282.1*	Unknown	1.504	2.820 × 10^−12^	5.24 × 10^−8^
	*klc1a*	Kinesin Light Chain 1	1.817	1.639× 10^−10^	1.52 × 10^−6^
	*isg15*	ISG15 Ubiquitin Like Modifier	1.743	6.974 × 10^−9^	4.32 × 10^−5^
	*cdk5r1b*	Cyclin Dependent Kinase 5 Regulatory Subunit 1	1.645	1.421 × 10^−8^	6.60 × 10^−5^
	*trim23*	Tripartite Motif Containing 23	1.743	4.151 × 10^−8^	1.54 × 10^−4^
	*si:ch1073-44g3.1*	Unknown	1.307	9.859 × 10^−8^	2.91 × 10^−4^
	*kdm2ba*	Lysine Demethylase 2B	1.121	1.095 × 10^−7^	2.91 × 10^−4^
	*l1camb*	L1 Cell Adhesion Molecule	2.084	1.496 × 10^−7^	3.41 × 10^−4^
	*soul4*	Heme-binding protein soul4	1.374	1.653 × 10^−7^	3.41 × 10^−4^
	*xpr1a*	Xenotropic and polytropic retrovirus receptor 1a	1.720	1.989 × 10^−7^	3.70 × 10^−4^

## Data Availability

Transcriptome data have been deposited into NCBI Gene Expression Omnibus and is available at GEO Accession number GSE324353.

## Supplementary Materials

The following supporting information can be downloaded at: https://www.mdpi.com/article/10.3390/genes17050558/s1, QC Reports for libraries, Summary reports for Bioinformatics (iPathway). Figure S1. Biological processes (gene ontology) identified as enriched based on iPathway using a chord diagram (FDR < 0.05). Figure S2. Biological processes (gene ontology) identified as enriched based on iPathway using a chord diagram (FDR < 0.05). Figure S3. Chord diagram illustrates the relationships between differentially expressed genes and enriched Hallmark gene signatures following 0.1 µg/L PFNA exposure. Figure S4. Chord diagram illustrates the relationship between differentially expressed genes and enriched Hallmark gene signatures following 10 µg/L PFNA exposure. Table S1. List of primers used for gene expression analysis.

## Abbreviations

The following abbreviations are used in this manuscript:

ANOVA	Analysis of variance
AO	Acridine orange
BCA	Bicinchoninic acid
CDNA	Complementary deoxyribonucleic acid
CNS	Central nervous system
Cq	Cycle Threshold
CTD	Comparative Toxicogenomics Database
DMSO	Dimethyl sulfoxide
DPF	Days post fertilization
ERM	Embryo rearing media
FABP	Fatty acid-binding protein
GEO	Gene Expression Omnibus
GFP	Green fluorescent protein
GO	Gene ontology
HISAT2	Hierarchal Indexing for Spliced Alignment of Transcripts 2
HPF	Hours post fertilization
H2-DCFDA	2′,7′-Dichlorodihydrofluorescein diacetate
IACUC	Institutional Animal Care and Use Committee
KEGG	Kyoto Encyclopedia of Genes and Genomes
NRT	No reverse transcriptase
OECD	Organization for Economic Co-operation and Development
PBS	Phosphate-buffered saline
PCR	Polymerase chain reaction
PFAS	Per- and polyfluoroalkyl substances
PFNA	Perfluorononanoic acid
PPAR	Peroxisome proliferator activated receptor
RNA	Ribonucleic acid
ROS	Reactive oxygen species
RT-qPCR	Reverse transcription quantitative polymerase chain reaction
SD	Standard deviation
USA	United States of America
VMR	Visual Motor Response
ZIRC	Zebrafish International Resource Center
