# Supplementary material for: Transcriptome and Behavioral Assessment in Larval Zebrafish (Danio rerio) Following Exposure to Perfluorononanoic Acid (PFNA)
Source: Genes (Basel). 2026 May 7;17(5):558. doi: 10.3390/genes17050558 (PMC13206344; doi:10.3390/genes17050558)
Supplement: Supplementary file 1 [file genes-17-00558-s001.zip › PFNA 0.1 Summary Report.pdf]

iPathwayGuide

**Title:**	PFNA 0.1
**Description:**	File: Differential_expression_analysis_table low.txt
**Organism:**	Homo sapiens (9606)
**Contrast**	PFNA 0.1 vs. Control - mRNA (RNA-seq)
**Creation time:**	09-20-2025 09:28 AM

1. Introduction

In this experiment, **107** differentially expressed (DE) genes were identified out of a total of **11,443** genes with measured expression. These were identified using thresholds defined by the user. In this experiment, the user chose a threshold of **0.05** for statistical significance (p-value) and a log fold change of expression with absolute value of at least **0.6**. These data were analyzed in the context of pathways obtained from the Kyoto Encyclopedia of Genes and Genomes (KEGG) database (Release 113.0+/01-01, Jan 25) (Kanehisa *et al*., 2000; Kanehisa *et al*., 2002), gene ontologies from the Gene Ontology Consortium database (2024-Sep20) (Ashburner *et al*., 2000; Gene Ontology Consortium, 2001), miRNAs from the miRBase (MIRBASE Version:Version22.1,10/18) and TARGETSCAN (Targetscan version: Mouse:8.0, Human:8.0) databases (Agarwal *et al*., 2015; Nam *et al*., 2014; Griffiths-Jones et al., 2008; Kozomara and Griffiths-Jones, 2014; Friedman *et al*., 2009; Grimson *et al*., 2007), network of regulatory relations from BioGRID: Biological General Repository for Interaction Datasets v4.4.233. April. 25th, 2024 (Szklarczyk *et al*., 2017), chemicals/drugs/toxicants from the Comparative Toxicogenomics Database January 7, 2025 (17601) (Davis *et al*., 2019), and diseases from the KEGG database (Release 113.0+/01-01, Jan 25) (Kanehisa *et al*., 2000; Kanehisa *et al*., 2002). In summary, **8** pathways were found to be significantly impacted. In addition, **280** Gene Ontology (GO) terms, **81** miRNAs , **108** gene upstream regulators, **150** chemical upstream regulators and **61** diseases were found to be significantly enriched before the correction for multiple comparisons.

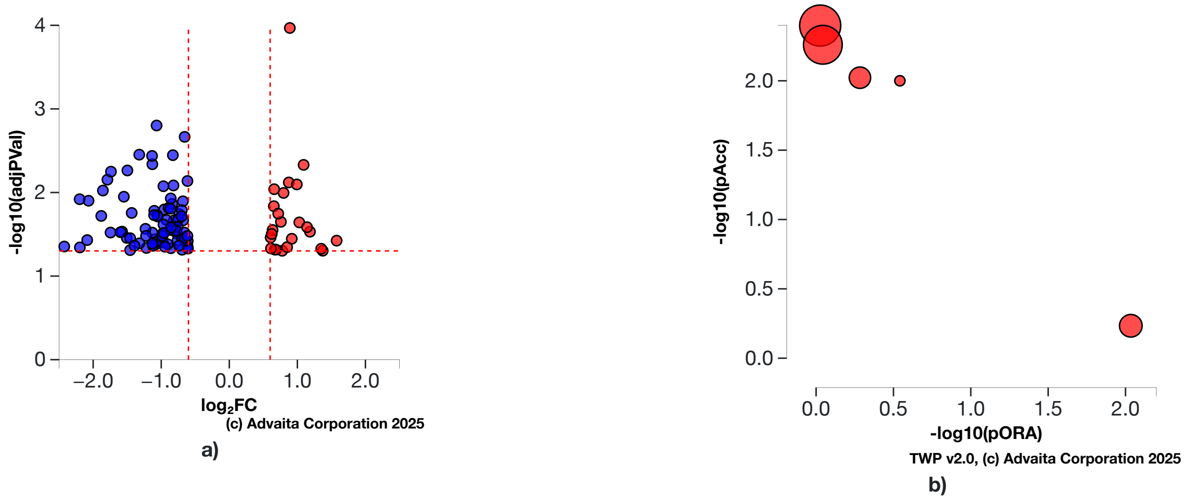

Fig. 1.1: a) Volcano plot: *All **107** significantly differentially expressed (DE) genes are represented in terms of their measured expression change (x-axis) and the significance of the change (y-axis). The significance is represented in terms of the negative log (base 10) of the p-value, so that more significant genes are plotted higher on the y-axis. The dotted lines represent the thresholds used to select the DE genes: **0.6** for expression change and **0.05** for significance. The up-regulated genes (positive log fold change) are shown in red, while the down-regulated genes are blue.* **b) Pathways perturbation vs over-representation:** *The top 5 pathways are plotted in terms of the two types of evidence computed by iPathwayGuide: over-representation on the x-axis (pORA) and the total pathway accumulation on the y-axis (pAcc). Each pathway is represented by a single dot, with significant pathways shown in red, non-significant in black, and the size of each dot is proportional to the size of the pathway it represents. Both p-values are shown in terms of their negative log (base 10) values.*

2. Pathway Analysis

2.1. Methods

iPathwayGuide scores pathways using the Impact Analysis method (Draghici *et al*., 2007; Tarca *et al*., 2009, Khatri *et al*., 2007). Impact analysis uses two types of evidence: i) the over-representation of differentially expressed (DE) genes in a given pathway and ii) the perturbation of that pathway computed by propagating the measured expression changes across the pathway topology. These aspects are captured by two independent probability values, pORA and pAcc, that are then combined in a unique pathway-specific p-value. The underlying pathway topologies, comprised of genes and their directional interactions, are obtained from the KEGG database (Kanehisa *et al*., 2000; Kanehisa *et al*., 2010; Kanehisa *et al*., 2012; Kanehisa *et al*., 2014).

The first probability, pORA, expresses the probability of observing the number of DE genes in a given pathway that is greater than or equal to the number observed, by random chance (Draghici *et al*., 2003; Draghici 2011). Let us consider there are *N* genes measured in the experiment, with *M* of these on the given pathway. Based on the user-defined a priori selection of DE genes, *K* out of *M* genes were found to be differentially expressed. The probability of observing exactly *x* differentially expressed genes on the given pathway is computed based on the hypergeometric distribution:

$$(1) \qquad P(X=x|N,M,K) = \frac{\binom{M}{x}\binom{N-M}{K-x}}{\binom{N}{K}}$$

Because the hypergeometric distribution is discrete, the probability of observing fewer than *x* genes on the given pathway just by chance can be calculated by summing the probabilities of randomly observing *0*, *1*, *2*, ..., up to *x-1* DE genes on the pathway:

$$(2) \qquad p_u(x-1) = P(X=1)+P(X=2)+\ldots+P(X=x-1) = \sum_{i=0}^{x-1} \frac{\binom{M}{i}\binom{N-M}{K-i}}{\binom{N}{K}}$$

iPathwayGuide calculates the probability of randomly observing a number of DE genes on the given pathway that is greater than or equal to the number of DE genes obtained from data, by computing the over-representation p-value: $pORA = p_o(x) = 1 - p_u(x-1)$:

$$(3) \qquad p_o(x) = 1 - \sum_{i=0}^{x-1} \frac{\binom{M}{i}\binom{N-M}{K-i}}{\binom{N}{K}}$$

The second probability, pAcc, is calculated based on the amount of total accumulation measured in each pathway. A perturbation factor is computed for each gene on the pathway using:

$$(4) \qquad PF(g) = \alpha(g) \cdot \triangle E(g) + \sum_{u \in US_g} \beta_{ug} \frac{PF(u)}{N_{ds}(u)}$$

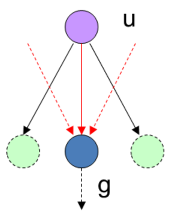

In Equation 4, *PF(g)* is the perturbation factor for gene *g*, the term *ΔE(g)* represents the signed normalized measured expression change of gene *g*, and *α(g)* is a priori weight based on the type of the gene. The last term is the sum of the perturbation factors of all genes *u*, directly upstream of the target gene *g*, normalized by the number of downstream genes of each such gene $N_{ds}(u)$. The value of β_{ug} quantifies the strength of the interaction between genes *g* and *u*. The sign of *β* represents the type of interaction: positive for activation-like signals, and negative for inhibition-like signals. Subsequently, iPathwayGuide calculates the accumulation at the level of each gene, *Acc(g)*, as the difference between the perturbation factor *PF(g)* and the observed log fold-change:

$$(5) \qquad Acc(g_i) = PF(g_i) - \triangle E(g_i)$$

All perturbation accumulations are computed at the same time by solving the system of linear equations resulting from combining Equation 4 for all genes on a given pathway. Once all gene perturbation accumulations are computed, iPathwayGuide computes the total accumulation of the pathway as the sum of all absolute accumulations of the genes in a given pathway. The significance of obtaining a total accumulation (pAcc) at least as large as observed, just by chance, is assessed through bootstrap analysis.

The two types of evidence, pORA and pAcc, are combined into an overall pathway score by calculating a p-value using Fisher's method. This p-value is then corrected for multiple comparisons using false dicovery rate (FDR) and Bonferroni corrections. Bonferroni is simpler and more conservative of the two (Bonferroni, 1935; Bonferroni, 1936). It reduces the false discovery rate by imposing a stringent threshold on each comparison adjusted for the total number of comparisons. The FDR correction has more power, but only controls the family-wise false positives rate (Benjamini and Hochberg, 1995; Benjamini and Yekutieli, 2001).

2.2. Results

Table 2.2.1: Top pathways and their associated p-values

Pathway name	Pathway Id	p-value	p-value (FDR)	p-value (Bonferroni)
Type II diabetes mellitus	04930	**0.020**	0.490	1.000
Alzheimer disease	05010	**0.025**	0.490	1.000
Melanogenesis	04916	**0.031**	0.490	1.000
Human papillomavirus infection	05165	**0.031**	0.490	1.000
HIF-1 signaling pathway	04066	**0.034**	0.490	1.000

* the p-value corresponding to the pathway was computed using only over-representation analysis.

Type II diabetes mellitus (KEGG: 04930)

Insulin resistance is strongly associated with type II diabetes. "Diabetogenic" factors including FFA, TNFalpha and cellular stress induce insulin resistance through inhibition of IRS1 functions. Serine/threonine phosphorylation, interaction with SOCS, regulation of the expression, modification of the cellular localization, and degradation represent the molecular mechanisms stimulated by them. Various kinases (ERK, JNK, IKKbeta, PKCzeta, PKCtheta and mTOR) are involved in this process. The development of type II diabetes requires impaired beta-cell function. Chronic hyperglycemia has been shown to induce multiple defects in beta-cells. Hyperglycemia has been proposed to lead to large amounts of reactive oxygen species (ROS) in beta-cells, with subsequent damage to cellular components including PDX-1. Loss of PDX-1, a critical regulator of insulin promoter activity, has also been proposed as an important mechanism leading to beta-cell dysfunction. Although there is little doubt as to the importance of genetic factors in type II diabetes, genetic analysis is difficult due to complex interaction among multiple susceptibility genes and between genetic and environmental factors. Genetic studies have therefore given very diverse results. Kir6.2 and IRS are two of the candidate genes. It is known that Kir6.2 and IRS play central roles in insulin secretion and insulin signal transmission, respectively.

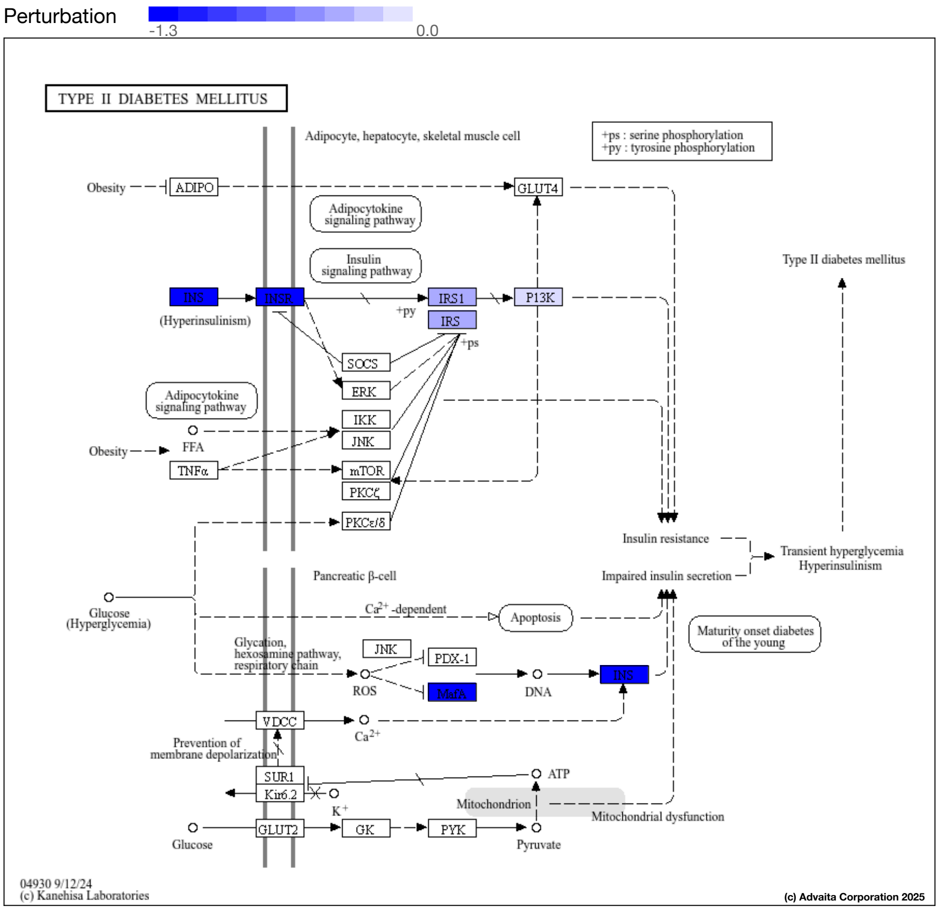

Fig. 2.2.1: Type II diabetes mellitus (KEGG: 04930): *The pathway diagram is overlayed with the computed perturbation of each gene. The perturbation accounts both for the gene's measured fold change and for the accumulated perturbation propagated from any upstream genes (accumulation). The highest negative perturbation is shown in dark blue, while the highest positive perturbation in dark red. The legend describes the values on the gradient. Note: For legibility, one gene may be represented in multiple places in the diagram and one box may represent multiple genes in the same gene family. A gene is highlighted in all locations it occurs in the diagram. For each gene family, the color corresponding to the gene with the highest absolute perturbation is displayed.*

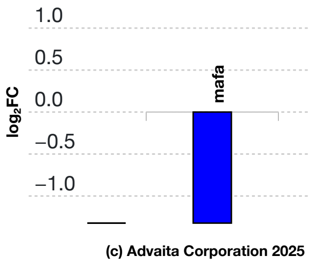

Fig. 2.2.2: Gene measured expression bar plot: *All the differentially expressed genes in Type II diabetes mellitus (KEGG: 04930) are ranked based on their absolute value of log fold change. Upregulated genes are shown in red, downregulated genes are shown in blue. The box and whisker plot on the left summarizes the distribution of all the differentially expressed genes in this pathway. The box represents the 1st quartile, the median and the 3rd quartile, while the outliers are represented by circles.*

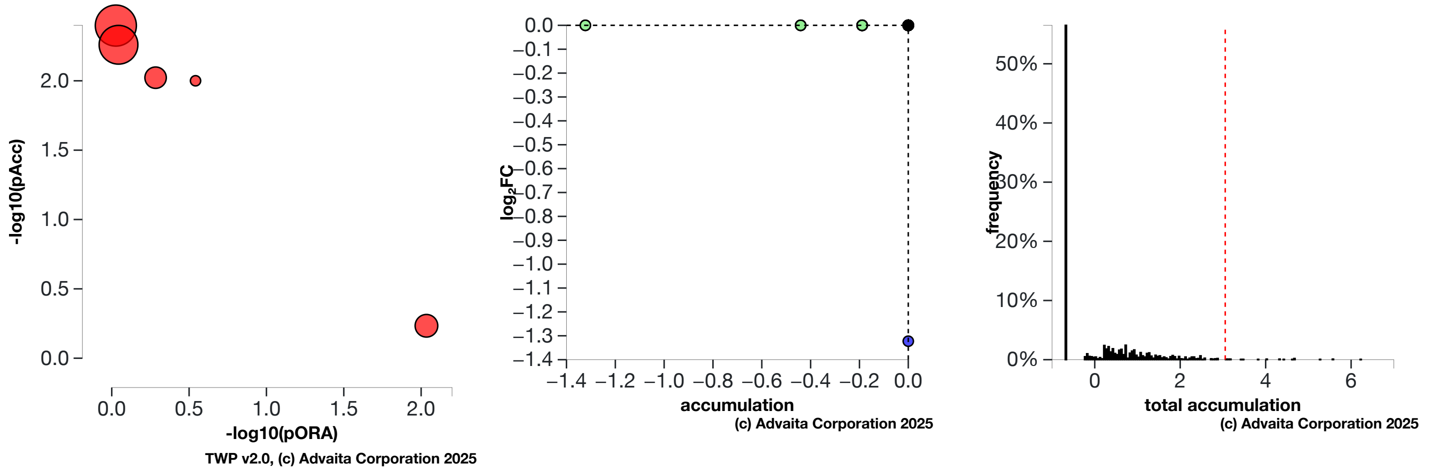

Fig. 2.2.3: a) Perturbation vs over-representation: *Type II diabetes mellitus (KEGG: 04930) (yellow) is shown, using negative log of the accumulation and over-representation p-values, along with the other most significant pathways. Pathways in red are significant based on the combined uncorrected p-values, whereas the ones in black are non-significant (where applicable).* **b) Gene measured expression vs accumulation:** *All the genes from this pathway are represented in terms of their measured fold change (y-axis) and accumulation (x-axis). Accumulation is the perturbation received by the gene from any upstream genes. Genes in blue had only measured fold change.Genes in green had only accumulation.The remaining genes that were not measured and had no accumulation are shown in black.* **c) Bootstrap diagram:** *The perturbation p-value is computed using bootstrap analysis. Bootstrapping assesses the probability of observing a sum of all absolute gene accumulation total accumulation at least as extreme as the computed one just by chance. A null distribution (gray bars) is computed through an iterative process that is repeated 2000 times. At each iteration, a number of genes equal to the number of differentially expressed genes in this pathway is randomly assigned anywhere in the pathway and the total accumulation is recomputed. The red line indicates the observed total accumulation of genes in the given pathway in relation to the distribution of expected values. The perturbation p-value is more significant the further away from the mean it is.*

Alzheimer disease (KEGG: 05010)

Alzheimer disease (AD) is a chronic disorder that slowly destroys neurons and causes serious cognitive disability. AD is associated with senile plaques and neurofibrillary tangles (NFTs). Amyloid-beta (Abeta), a major component of senile plaques, has various pathological effects on cell and organelle function. To date genetic studies have revealed four genes that may be linked to autosomal dominant or familial early onset AD (FAD). These four genes include: amyloid precursor protein (APP), presenilin 1 (PS1), presenilin 2 (PS2) and apolipoprotein E (ApoE). All mutations associated with APP and PS proteins can lead to an increase in the production of Abeta peptides, specfically the more amyloidogenic form, Abeta42. It was proposed that Abeta form Ca2+ permeable pores and bind to and modulate multiple synaptic proteins, including NMDAR, mGluR5 and VGCC, leading to the overfilling of neurons with calcium ions. Consequently, cellular Ca2+ disruptions will lead to neuronal apoptosis, autophagy deficits, mitochondrial abnormality, defective neurotransmission, impaired synaptic plasticity and neurodegeneration in AD. FAD-linked PS1 mutation downregulates the unfolded protein response and leads to vulnerability to ER stress.

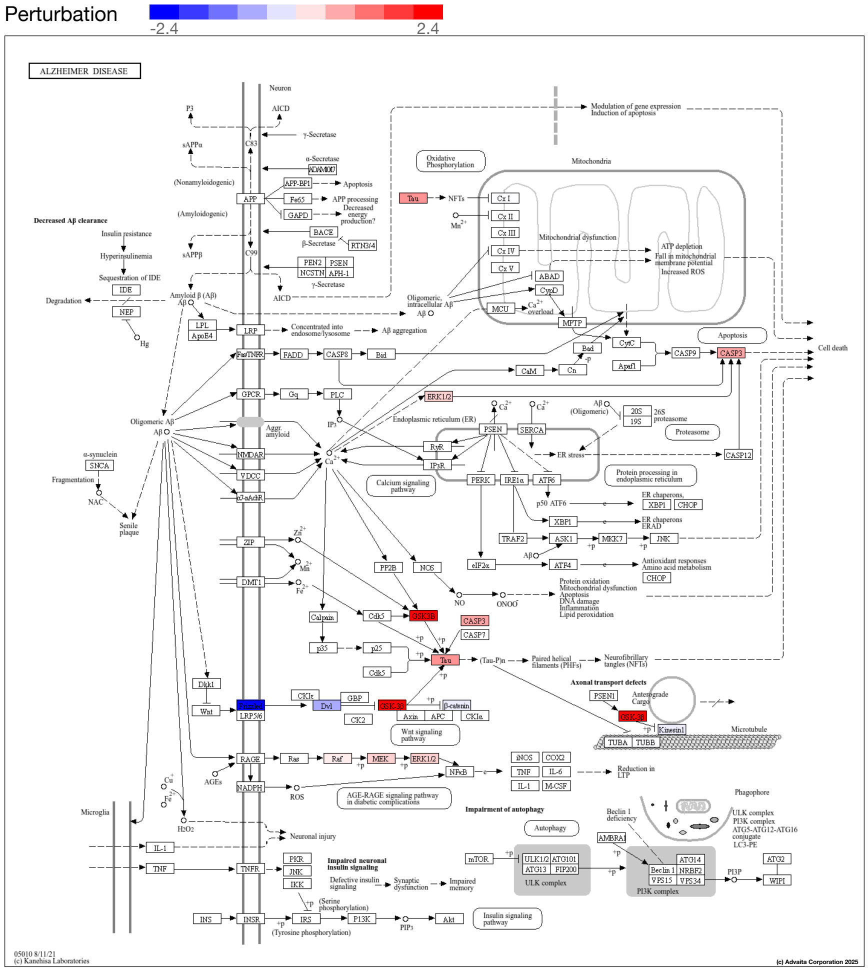

Fig. 2.2.4: Alzheimer disease (KEGG: 05010): *The pathway diagram is overlayed with the computed perturbation of each gene. The perturbation accounts both for the gene's measured fold change and for the accumulated perturbation propagated from any upstream genes (accumulation). The highest negative perturbation is shown in dark blue, while the highest positive perturbation in dark red. The legend describes the values on the gradient. Note: For legibility, one gene may be represented in multiple places in the diagram and one box may represent multiple genes in the same gene family. A gene is highlighted in all locations it occurs in the diagram. For each gene family, the color corresponding to the gene with the highest absolute perturbation is displayed.*

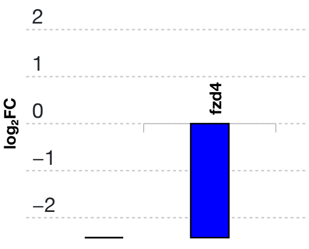

(c) Advaita Corporation 2025

Fig. 2.2.5: Gene measured expression bar plot: *All the differentially expressed genes in Alzheimer disease (KEGG: 05010) are ranked based on their absolute value of log fold change. Upregulated genes are shown in red, downregulated genes are shown in blue. The box and whisker plot on the left summarizes the distribution of all the differentially expressed genes in this pathway. The box represents the 1st quartile, the median and the 3rd quartile, while the outliers are represented by circles.*

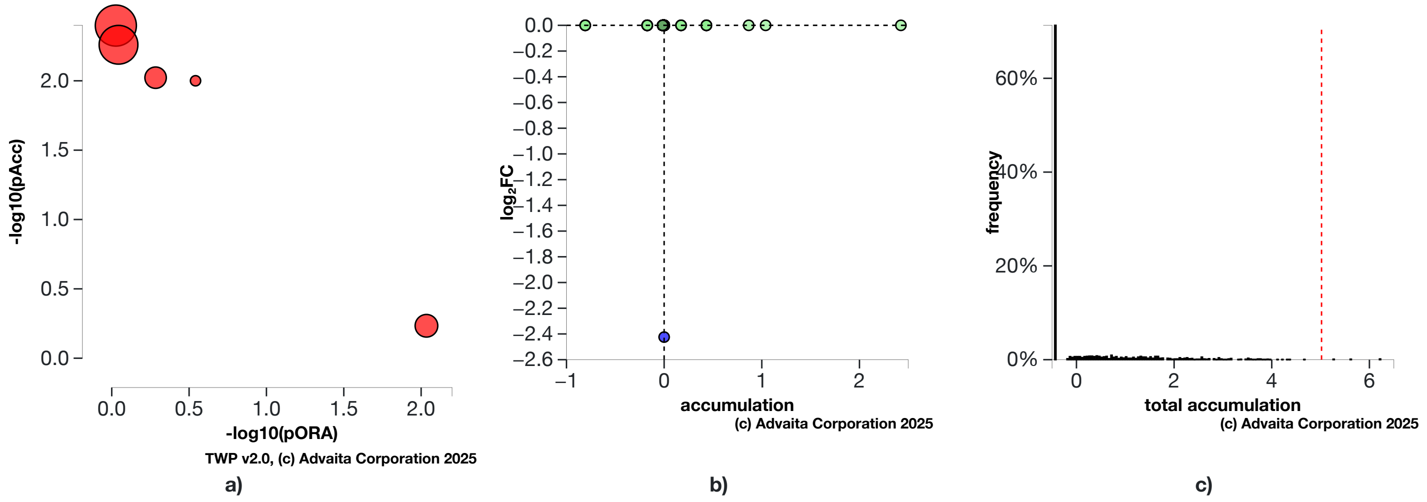

Fig. 2.2.6: a) Perturbation vs over-representation: *Alzheimer disease (KEGG: 05010) (yellow) is shown, using negative log of the accumulation and over-representation p-values, along with the other most significant pathways. Pathways in red are significant based on the combined uncorrected p-values, whereas the ones in black are non-significant (where applicable).* **b) Gene measured expression vs accumulation:** *All the genes from this pathway are represented in terms of their measured fold change (y-axis) and accumulation (x-axis). Accumulation is the perturbation received by the gene from any upstream genes. Genes in blue had only measured fold change.Genes in green had only accumulation.The remaining genes that were not measured and had no accumulation are shown in black.* **c) Bootstrap diagram:** *The perturbation p-value is computed using bootstrap analysis. Bootstrapping assesses the probability of observing a sum of all absolute gene accumulation total accumulation at least as extreme as the computed one just by chance. A null distribution (gray bars) is computed through an iterative process that is repeated 2000 times. At each iteration, a number of genes equal to the number of differentially expressed genes in this pathway is randomly assigned anywhere in the pathway and the total accumulation is recomputed. The red line indicates the observed total accumulation of genes in the given pathway in relation to the distribution of expected values. The perturbation p-value is more significant the further away from the mean it is.*

Melanogenesis (KEGG: 04916)

Cutaneous melanin pigment plays a critical role in camouflage, mimicry, social communication, and protection against harmful effects of solar radiation. Melanogenesis is under complex regulatory control by multiple agents. The most important positive regulator of melanogenesis is the MC1 receptor with its ligands melanocortic peptides. MC1R activates the cyclic AMP (cAMP) response-element binding protein (CREB). Increased expression of MITF and its activation by phosphorylation (P) stimulate the transcription of tyrosinase (TYR), tyrosinase-related protein 1 (TYRP1), and dopachrome tautomerase (DCT), which produce melanin. Melanin synthesis takes place within specialized intracellular organelles named melanosomes. Melanin-containing melanosomes then move from the perinuclear region to the dendrite tips and are transferred to keratinocytes by a still not well-characterized mechanism.

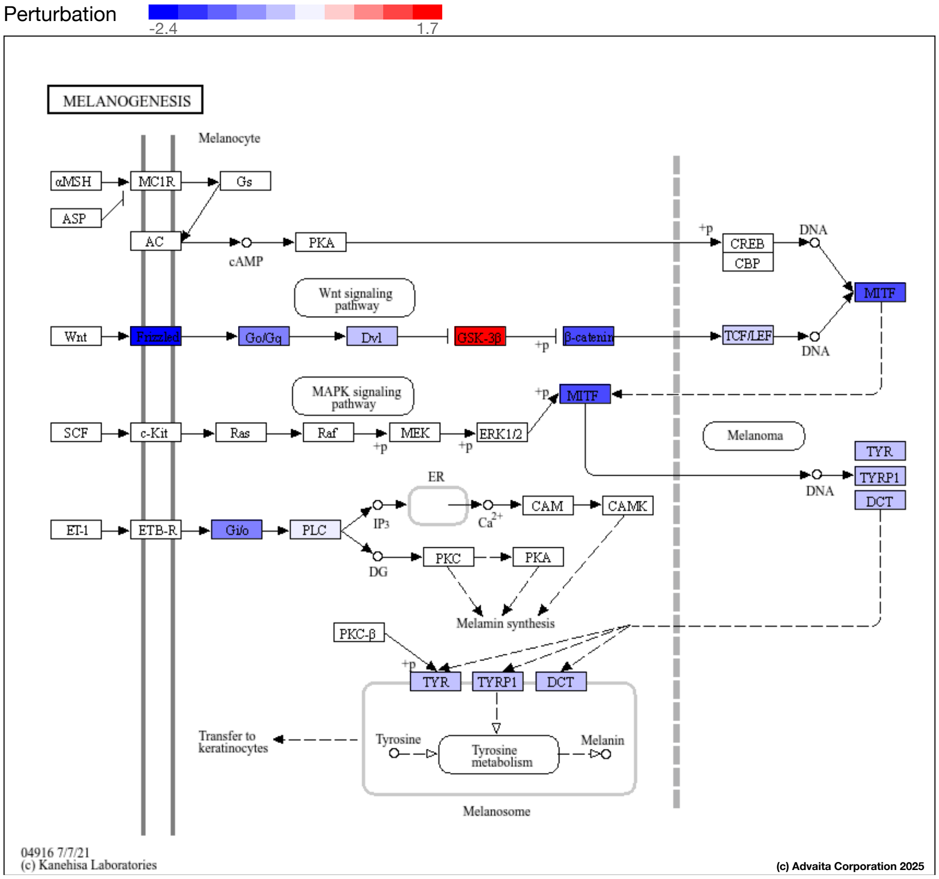

Fig. 2.2.7: Melanogenesis (KEGG: 04916): *The pathway diagram is overlayed with the computed perturbation of each gene. The perturbation accounts both for the gene's measured fold change and for the accumulated perturbation propagated from any upstream genes (accumulation). The highest negative perturbation is shown in dark blue, while the highest positive perturbation in dark red. The legend describes the values on the gradient. Note: For legibility, one gene may be represented in multiple places in the diagram and one box may represent multiple genes in the same gene family. A gene is highlighted in all locations it occurs in the diagram. For each gene family, the color corresponding to the gene with the highest absolute perturbation is displayed.*

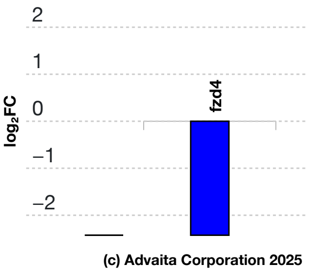

Fig. 2.2.8: Gene measured expression bar plot: *All the differentially expressed genes in Melanogenesis (KEGG: 04916) are ranked based on their absolute value of log fold change. Upregulated genes are shown in red, downregulated genes are shown in blue. The box and whisker plot on the left summarizes the distribution of all the differentially expressed genes in this pathway. The box represents the 1st quartile, the median and the 3rd quartile, while the outliers are represented by circles.*

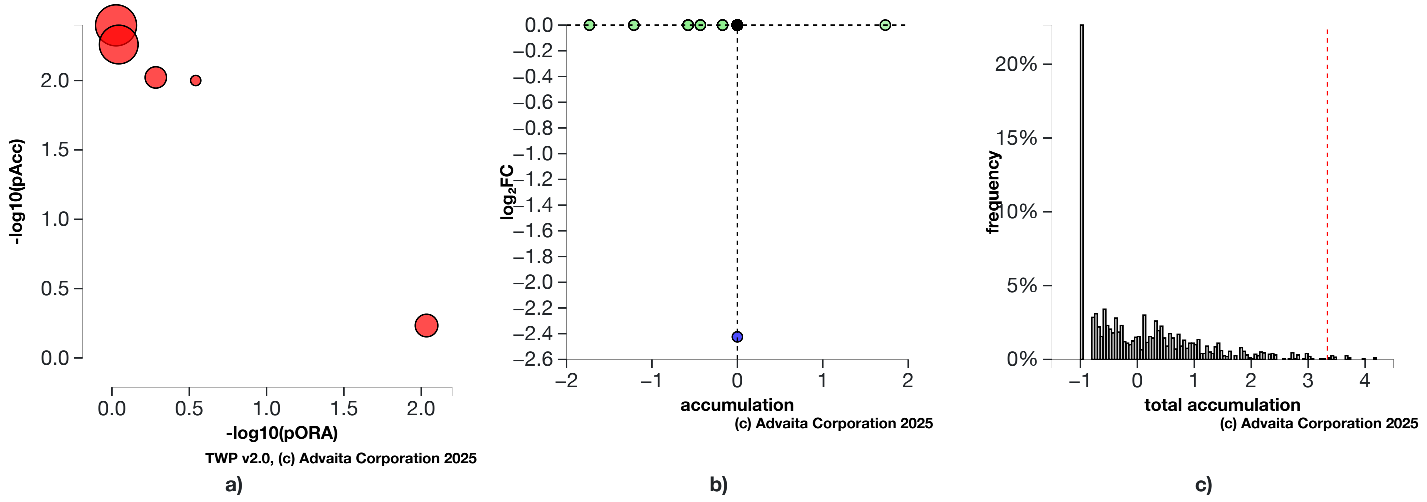

Fig. 2.2.9: a) Perturbation vs over-representation: *Melanogenesis (KEGG: 04916) (yellow) is shown, using negative log of the accumulation and over-representation p-values, along with the other most significant pathways. Pathways in red are significant based on the combined uncorrected p-values, whereas the ones in black are non-significant (where applicable).* **b) Gene measured expression vs accumulation:** *All the genes from this pathway are represented in terms of their measured fold change (y-axis) and accumulation (x-axis). Accumulation is the perturbation received by the gene from any upstream genes. Genes in blue had only measured fold change.Genes in green had only accumulation.The remaining genes that were not measured and had no accumulation are shown in black.* **c) Bootstrap diagram:** *The perturbation p-value is computed using bootstrap analysis. Bootstrapping assesses the probability of observing a sum of all absolute gene accumulation total accumulation at least as extreme as the computed one just by chance. A null distribution (gray bars) is computed through an iterative process that is repeated 2000 times. At each iteration, a number of genes equal to the number of differentially expressed genes in this pathway is randomly assigned anywhere in the pathway and the total accumulation is recomputed. The red line indicates the observed total accumulation of genes in the given pathway in relation to the distribution of expected values. The perturbation p-value is more significant the further away from the mean it is.*

Human papillomavirus infection (KEGG: 05165)

Human papillomavirus (HPV) is a non-enveloped, double-stranded DNA virus. HPV infect mucoal and cutaneous epithelium resulting in several types of pathologies, most notably, cervical cancer. All types of HPV share a common genomic structure and encode eight proteins: E1, E2, E4, E5, E6, and E7 (early) and L1 and L2 (late). It has been demonstrated that E1 and E2 are involved in viral transcription and replication. The functions of the E4 protein is not yet fully understood. E5, E6, and E7 act as oncoproteins. E5 inhibits the V-ATPase, prolonging EGFR signaling and thereby promoting cell proliferation. The expression of E6 and E7 not only inhibits the tumor suppressors p53 and Rb, but also alters additional signalling pathways. Among these pathways, PI3K/Akt signalling cascade plays a very important role in HPV-induced carcinogenesis. The L1 and L2 proteins form icosahedral capsids for progeny virion generation.

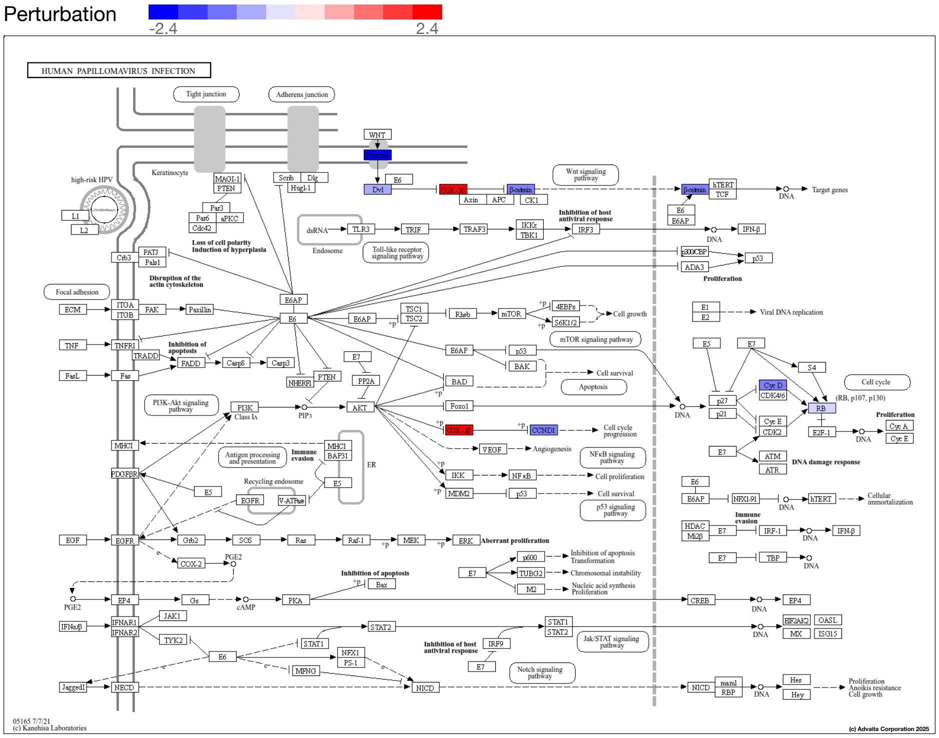

Fig. 2.2.10: Human papillomavirus infection (KEGG: 05165): *The pathway diagram is overlayed with the computed perturbation of each gene. The perturbation accounts both for the gene's measured fold change and for the accumulated perturbation propagated from any upstream genes (accumulation). The highest negative perturbation is shown in dark blue, while the highest positive perturbation in dark red. The legend describes the values on the gradient. Note: For legibility, one gene may be represented in multiple places in the diagram and one box may represent multiple genes in the same gene family. A gene is highlighted in all locations it occurs in the diagram. For each gene family, the color corresponding to the gene with the highest absolute perturbation is displayed.*

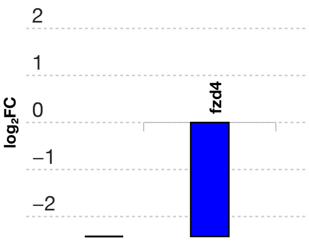

(c) Advaita Corporation 2025

Fig. 2.2.11: Gene measured expression bar plot: *All the differentially expressed genes in Human papillomavirus infection (KEGG: 05165) are ranked based on their absolute value of log fold change. Upregulated genes are shown in red, downregulated genes are shown in blue. The box and whisker plot on the left summarizes the distribution of all the differentially expressed genes in this pathway. The box represents the 1st quartile, the median and the 3rd quartile, while the outliers are represented by circles.*

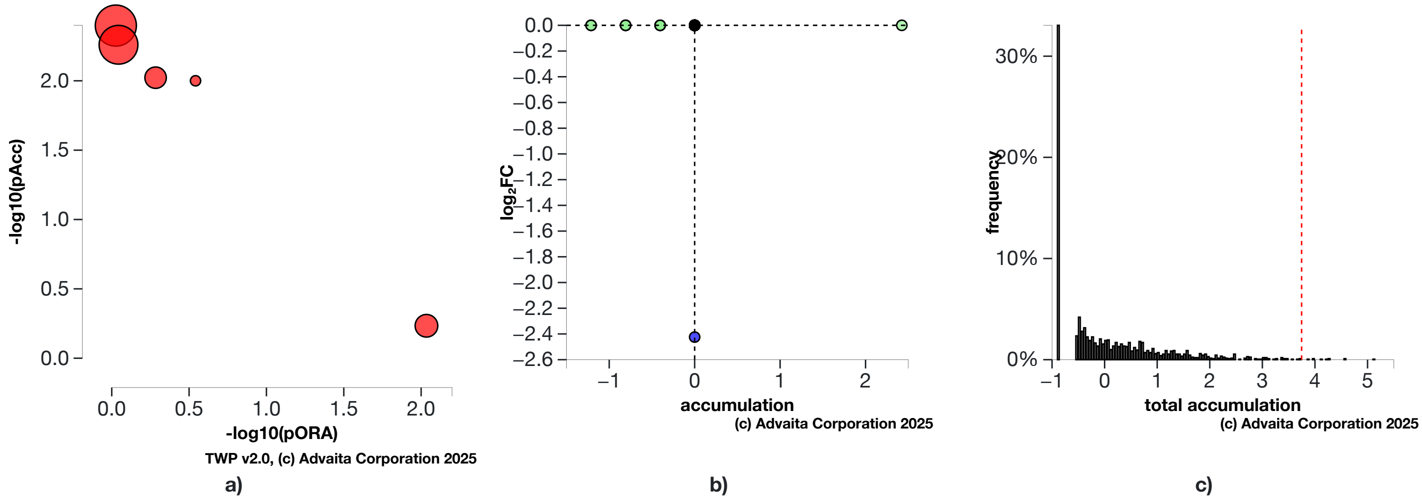

Fig. 2.2.12: a) Perturbation vs over-representation: *Human papillomavirus infection (KEGG: 05165) (yellow) is shown, using negative log of the accumulation and over-representation p-values, along with the other most significant pathways. Pathways in red are significant based on the combined uncorrected p-values, whereas the ones in black are non-significant (where applicable).* **b) Gene measured expression vs accumulation:** *All the genes from this pathway are represented in terms of their measured fold change (y-axis) and accumulation (x-axis). Accumulation is the perturbation received by the gene from any upstream genes. Genes in blue had only measured fold change.Genes in green had only accumulation.The remaining genes that were not measured and had no accumulation are shown in black.* **c) Bootstrap diagram:** *The perturbation p-value is computed using bootstrap analysis. Bootstrapping assesses the probability of observing a sum of all absolute gene accumulation total accumulation at least as extreme as the computed one just by chance. A null distribution (gray bars) is computed through an iterative process that is repeated 2000 times. At each iteration, a number of genes equal to the number of differentially expressed genes in this pathway is randomly assigned anywhere in the pathway and the total accumulation is recomputed. The red line indicates the observed total accumulation of genes in the given pathway in relation to the distribution of expected values. The perturbation p-value is more significant the further away from the mean it is.*

HIF-1 signaling pathway (KEGG: 04066)

Hypoxia-inducible factor 1 (HIF-1) is a transcription factor that functions as a master regulator of oxygen homeostasis. It consists of two subunits: an inducibly-expressed HIF-1alpha subunit and a constitutively-expressed HIF-1beta subunit. Under normoxia, HIF-1 alpha undergoes hydroxylation at specific prolyl residues which leads to an immediate ubiquitination and subsequent proteasomal degradation of the subunit. In contrast, under hypoxia, HIF-1 alpha subunit becomes stable and interacts with coactivators such as p300/CBP to modulate its transcriptional activity. Eventually, HIF-1 acts as a master regulator of numerous hypoxia-inducible genes under hypoxic conditions. The target genes of HIF-1 encode proteins that increase O2 delivery and mediate adaptive responses to O2 deprivation. Despite its name, HIF-1 is induced not only in response to reduced oxygen availability but also by other stimulants, such as nitric oxide, or various growth factors.

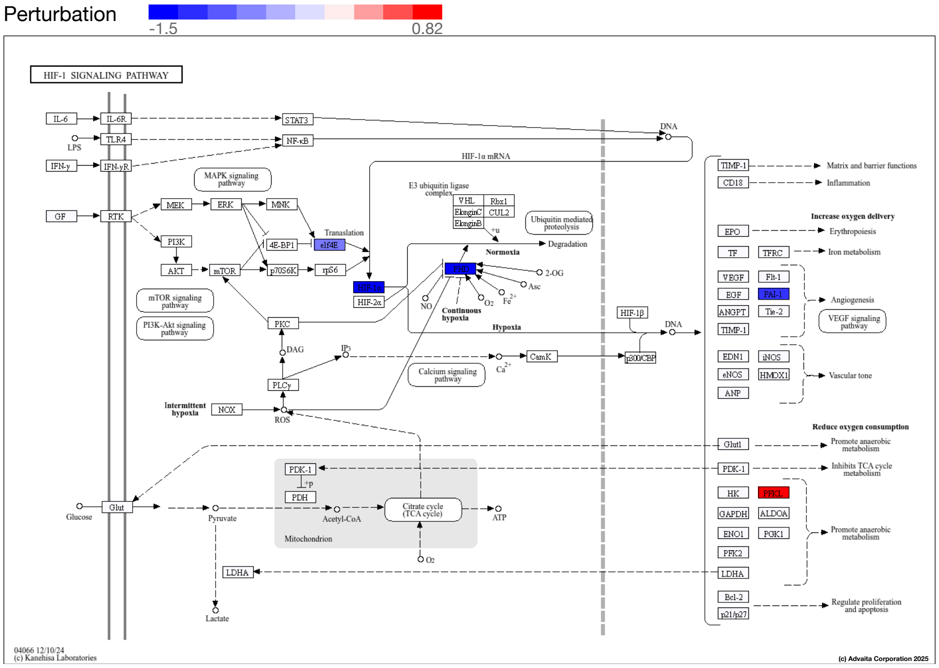

Fig. 2.2.13: HIF-1 signaling pathway (KEGG: 04066): *The pathway diagram is overlayed with the computed perturbation of each gene. The perturbation accounts both for the gene's measured fold change and for the accumulated perturbation propagated from any upstream genes (accumulation). The highest negative perturbation is shown in dark blue, while the highest positive perturbation in dark red. The legend describes the values on the gradient. Note: For legibility, one gene may be represented in multiple places in the diagram and one box may represent multiple genes in the same gene family. A gene is highlighted in all locations it occurs in the diagram. For each gene family, the color corresponding to the gene with the highest absolute perturbation is displayed.*

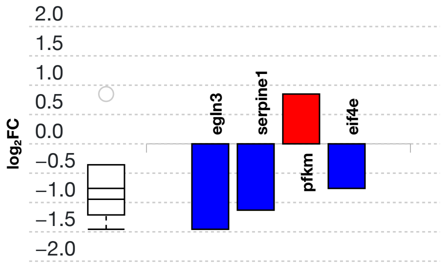

(c) Advaita Corporation 2025

Fig. 2.2.14: Gene measured expression bar plot: *All the differentially expressed genes in HIF-1 signaling pathway (KEGG: 04066) are ranked based on their absolute value of log fold change. Upregulated genes are shown in red, downregulated genes are shown in blue. The box and whisker plot on the left summarizes the distribution of all the differentially expressed genes in this pathway. The box represents the 1st quartile, the median and the 3rd quartile, while the outliers are represented by circles.*

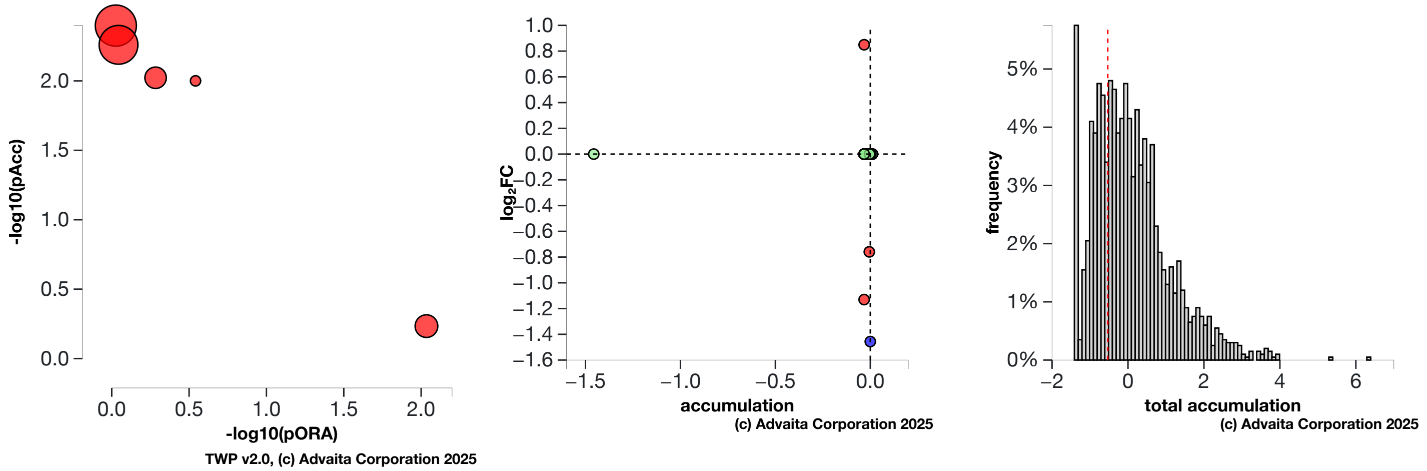

a) b) c)

Fig. 2.2.15: a) Perturbation vs over-representation: *HIF-1 signaling pathway (KEGG: 04066) (yellow) is shown, using negative log of the accumulation and over-representation p-values, along with the other most significant pathways. Pathways in red are significant based on the combined uncorrected p-values, whereas the ones in black are non-significant (where applicable).* **b) Gene measured expression vs accumulation:** *All the genes from this pathway are represented in terms of their measured fold change (y-axis) and accumulation (x-axis). Accumulation is the perturbation received by the gene from any upstream genes. Genes displayed in red had both accumulation and measured fold change.Genes in blue had only measured fold change.Genes in green had only accumulation.The remaining genes that were not measured and had no accumulation are shown in black.* **c) Bootstrap diagram:** *The perturbation p-value is computed using bootstrap analysis. Bootstrapping assesses the probability of observing a sum of all absolute gene accumulation total accumulation at least as extreme as the computed one just by chance. A null distribution (gray bars) is computed through an iterative process that is repeated 2000 times. At each iteration, a number of genes equal to the number of differentially expressed genes in this pathway is randomly assigned anywhere in the pathway and the total accumulation is recomputed. The red line indicates the observed total accumulation of genes in the given pathway in relation to the distribution of expected values. The perturbation p-value is more significant the further away from the mean it is.*

3. Gene Ontology Analysis

3.1. Methods

For each Gene Ontology (GO) term (Ashburner *et al*., 2002; Gene Ontology Consortium, 2004), the number of differentially expressed (DE) genes annotated to the term is compared to the number of DE genes expected just by chance. iPathwayGuide uses an over-representation approach to compute the statistical significance of observing at least the given number of DE genes. The p-value is computed using the hypergeometric distribution as described for pORA in the Pathway Analysis section. This p-value is corrected for multiple comparisons using FDR and Bonferroni.

The classical enrichment method used above considers all GO terms to be independent. By definition, all genes annotated to a GO term are also annotated to its ancestors. Because of this, the enrichment approach counts each gene multiple times by propagating it through the GO hierarchy from the most specific term the gene is associated with, all the way to the root of the ontology. This introduces redundancy in the analysis and reports many general and non-informative terms as significant. To overcome this limitation, iPathwayGuide allows users to use two more sophisticated pruning methods: *high-specificity pruning* and *smallest common denominator pruning*. The **high-specificity** pruning method *identifies the most specific GO terms* that are significantly associated with the set of DE genes. Let us consider, BP1 = “induction of apoptosis by intracellular signals” and BP2 = “induction of apoptosis by extracellular signals,” which are two of the children of BP3 = “induction of apoptosis.” If enough DE genes are associated with BP1 and BP2, the high-specificity pruning will report them as significant. The **smallest common denominator** pruning method *identifies the GO terms that best encapsulate the set of DE genes*, at times consolidating significance of two or more specific terms into their common parent. In the example above, this pruning method might report BP3 as significant because it is the most specific biological term that would include all DE genes that make both BP1 and BP2 significant.

3.2. Biological Processes results

Table 3.2.1: Top identified biological processes. Only the top scoring biological process for each pruning type is described below the table.

Pruning Type: None				Pruning Type: High-specificity		Pruning Type: Smallest Common Denominator	
**GO Term**	**p-value**	**p-value (FDR)**	**p-value (Bonferroni)**	**GO Term**	**p-value**	**GO Term**	**p-value**
dentinogenesis	8.800e-4	0.577	1.000	dentinogenesis	1.000	dentinogenesis	1.000
lipid homeostasis	0.001	0.577	1.000	lipid homeostasis	1.000	lipid homeostasis	1.000
homotypic cell-cell adhesion	0.004	0.577	1.000	homotypic cell-cell adhesion	1.000	homotypic cell-cell adhesion	1.000
regulation of monocyte chemotaxis	0.005	0.577	1.000	regulation of monocyte chemotaxis	1.000	regulation of monocyte chemotaxis	1.000
regulation of lysosomal protein catabolic process	0.005	0.577	1.000	regulation of lysosomal protein catabolic process	1.000	regulation of lysosomal protein catabolic process	1.000

dentinogenesis (GO:0097187)

The process whose specific outcome is the formation of dentin, the mineralized tissue that constitutes the major bulk of teeth. Dentin may be one of three types: primary dentin, secondary dentin, and tertiary dentin. In this experiment, the algorithm identified **2** differentially expressed gene(s) out of ALL **5** gene(s).

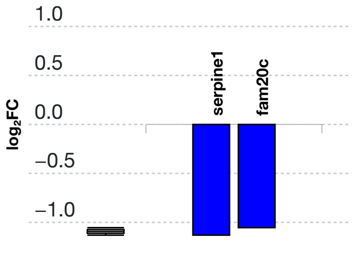

Fig. 3.2.1: Gene measured expression bar plot: *All the differentially expressed genes that are annotated to dentinogenesis are ranked based on their absolute value of log fold change. Upregulated genes are shown in red, downregulated genes are shown in blue. The box and whisker plot on the left summarizes the distribution of all the differentially expressed genes that are annotated to this GO term. The box represents the 1st quartile, the median and the 3rd quartile, while the outliers are represented by circles.*

3.3. Molecular Functions results

Table 3.3.1: Top identified molecular functions. Only the top scoring molecular function for each pruning type is described below the table.

Pruning Type: None				Pruning Type: High-specificity		Pruning Type: Smallest Common Denominator	
GO Term	p-value	p-value (FDR)	p-value (Bonferroni)	GO Term	p-value	GO Term	p-value
BMP binding	**2.100e-4**	0.097	0.097	BMP binding	0.097	BMP binding	0.097
cytokine binding	**4.600e-4**	0.106	0.212	amine binding	0.351	amine binding	0.339
amine binding	**0.002**	0.223	1.000	cholesterol binding	0.351	cholesterol binding	0.339
steroid binding	**0.006**	0.223	1.000	beta-1,4-mannosylglycoprotein 4-beta-N-acetylglucosaminyltransferase activity	0.351	beta-1,4-mannosylglycoprotein 4-beta-N-acetylglucosaminyltransferase activity	0.339
cholesterol binding	**0.007**	0.223	1.000	eukaryotic initiation factor 4G binding	0.351	eukaryotic initiation factor 4G binding	0.339

BMP binding (GO:0036122)

Binding to a member of the bone morphogenetic protein (BMP) family. In this experiment, the algorithm identified **3** differentially expressed gene(s) out of ALL **13** gene(s).

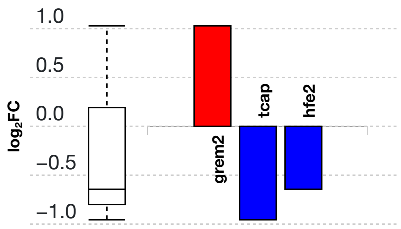

(c) Advaita Corporation 2025

Fig. 3.3.2: Gene measured expression bar plot: *All the differentially expressed genes that are annotated to BMP binding are ranked based on their absolute value of log fold change. Upregulated genes are shown in red, downregulated genes are shown in blue. The box and whisker plot on the left summarizes the distribution of all the differentially expressed genes that are annotated to this GO term. The box represents the 1st quartile, the median and the 3rd quartile, while the outliers are represented by circles.*

3.4. Cellular Components results

Table 3.4.1: Top identified cellular components. Only the top scoring cellular component for each pruning type is described below the table.

Pruning Type: None				Pruning Type: High-specificity		Pruning Type: Smallest Common Denominator	
**GO Term**	**p-value**	**p-value (FDR)**	**p-value (Bonferroni)**	**GO Term**	**p-value**	**GO Term**	**p-value**
presynaptic active zone cytoplasmic component	0.007	0.515	1.000	presynaptic active zone cytoplasmic component	0.802	presynaptic active zone cytoplasmic component	0.788
collagen type VIII trimer	0.009	0.515	1.000	collagen type VIII trimer	0.802	collagen type VIII trimer	0.788
PCSK9-LDLR complex	0.009	0.515	1.000	PCSK9-LDLR complex	0.802	PCSK9-LDLR complex	0.788
intraciliary transport particle	0.015	0.515	1.000	transcription factor TFIIIB complex	0.802	intraciliary transport particle	0.788
transcription factor TFIIIB complex	0.019	0.515	1.000	BLOC-3 complex	0.802	transcription factor TFIIIB complex	0.788

presynaptic active zone cytoplasmic component (GO:0098831)

A specialized region below the presynaptic membrane, characterized by electron-dense material, a specialized cytoskeletal matrix and accumulated (associated) synaptic vesicles. In this experiment, the algorithm identified **2** differentially expressed gene(s) out of ALL **14** gene(s).

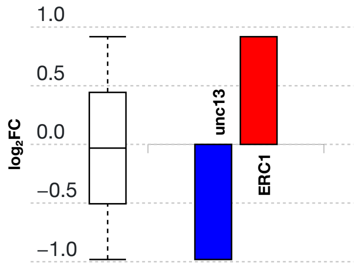

Fig. 3.4.3: Gene measured expression bar plot: *All the differentially expressed genes that are annotated to presynaptic active zone cytoplasmic component are ranked based on their absolute value of log fold change. Upregulated genes are shown in red, downregulated genes are shown in blue. The box and whisker plot on the left summarizes the distribution of all the differentially expressed genes that are annotated to this GO term. The box represents the 1st quartile, the median and the 3rd quartile, while the outliers are represented by circles.*

4. Advaita DB

4.1. Methods

For each Gene Set (GO) term (Ashburner *et al*., 2002; Gene Ontology Consortium, 2004), the number of differentially expressed (DE) genes to the term is compared to the number of DE genes expected just by chance. iPathwayGuide uses an over-representation approach to compute the statistical significance of observing at least the given number of DE genes. The p-value is computed using the hypergeometric distribution as described for pORA in the Pathway Analysis section. This p-value is corrected for multiple comparisons using FDR and Bonferroni.

The classical enrichment method used above considers all gene sets to be independent. By definition, all genes annotated to a GO term are also annotated to its ancestors. Because of this, the enrichment approach counts each gene multiple times by propagating it through the GO hierarchy from the most specific term the gene is associated with, all the way to the root of the ontology. This introduces redundancy in the analysis and reports many general and non-informative terms as significant. To overcome this limitation, iPathwayGuide allows users to use two more sophisticated pruning methods: *high-specificity pruning* and *smallest common denominator pruning*. The **high-specificity** pruning method *identifies the most specific gene sets* that are significantly associated with the set of DE genes. Let us consider, BP1 = “induction of apoptosis by intracellular signals” and BP2 = “induction of apoptosis by extracellular signals,” which are two of the children of BP3 = “induction of apoptosis.” If enough DE genes are associated with BP1 and BP2, the high-specificity pruning will report them as significant. The **smallest common denominator** pruning method *identifies the gene sets that best encapsulate the set of DE genes*, at times consolidating significance of two or more specific terms into their common parent. In the example above, this pruning method might report BP3 as significant because it is the most specific biological term that would include all DE genes that make both BP1 and BP2 significant.

4.2. Organ signatures

Table 4.2.1: Top identified Organ signatures gene sets. Only the top scoring Organ signatures gene sets are described below the table.

Gene Set	p-value	p-value (FDR)	p-value (Bonferroni)
MINOR SALIVARY GLAND	0.03042	0.06083	0.06083
HEART	0.2568	0.2568	0.5137

MINOR SALIVARY GLAND (12617)

The salivary glands in mammals are exocrine glands that produce saliva through a system of ducts. Humans have three paired major salivary glands (parotid, submandibular, and sublingual), as well as hundreds of minor salivary glands. Salivary glands can be classified as serous, mucous, or seromucous (mixed). In this experiment, the algorithm identified **1** differentially expressed gene(s) out of ALL **2** gene(s).

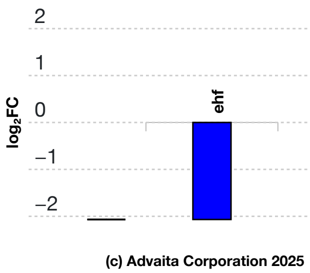

Fig. 4.2.1: Gene measured expression bar plot: *All the differentially expressed genes that are annotated to MINOR SALIVARY GLAND are ranked based on their absolute value of log fold change. The plot is limited to the top 5 genes out of a total of 1 differentially expressed genes. Upregulated genes are shown in red, downregulated genes are shown in blue. The box and whisker plot on the left summarizes the distribution of all the differentially expressed genes that are annotated to this gene set. The box represents the 1st quartile, the median and the 3rd quartile, while the outliers are represented by circles.*

HEART (12613)

Heart is a hollow muscular organ that pumps the blood through the circulatory system by rhythmic contraction and dilation. In vertebrates there may be up to four chambers (as in humans), with two atria and two ventricles. In this experiment, the algorithm identified **1** differentially expressed gene(s) out of ALL **18** gene(s).

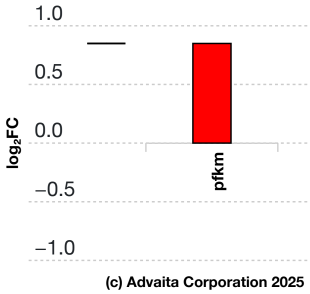

Fig. 4.2.2: Gene measured expression bar plot: *All the differentially expressed genes that are annotated to HEART are ranked based on their absolute value of log fold change. The plot is limited to the top 5 genes out of a total of 1 differentially expressed genes. Upregulated genes are shown in red, downregulated genes are shown in blue. The box and whisker plot on the left summarizes the distribution of all the differentially expressed genes that are annotated to this gene set. The box represents the 1st quartile, the median and the 3rd quartile, while the outliers are represented by circles.*

4.3. Toxicity

Table 4.3.1: Top identified Toxicity gene sets. Only the top scoring Toxicity gene sets are described below the table.

Gene Set	p-value	p-value (FDR)	p-value (Bonferroni)
Liver Toxicity	0.0001265	0.0001265	0.0001265

Liver Toxicity (12646)

A collection of genes highly related to liver toxicity In this experiment, the algorithm identified **2** differentially expressed gene(s) out of ALL **98** gene(s).

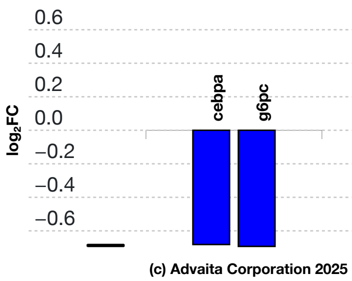

Fig. 4.3.1: Gene measured expression bar plot: *All the differentially expressed genes that are annotated to Liver Toxicity are ranked based on their absolute value of log fold change. The plot is limited to the top 5 genes out of a total of 2 differentially expressed genes. Upregulated genes are shown in red, downregulated genes are shown in blue. The box and whisker plot on the left summarizes the distribution of all the differentially expressed genes that are annotated to this gene set. The box represents the 1st quartile, the median and the 3rd quartile, while the outliers are represented by circles.*

5. Gene Ontology

5.1. Methods

For each Gene Set (GO) term (Ashburner *et al*., 2002; Gene Ontology Consortium, 2004), the number of differentially expressed (DE) genes to the term is compared to the number of DE genes expected just by chance. iPathwayGuide uses an over-representation approach to compute the statistical significance of observing at least the given number of DE genes. The p-value is computed using the hypergeometric distribution as described for pORA in the Pathway Analysis section. This p-value is corrected for multiple comparisons using FDR and Bonferroni.

The classical enrichment method used above considers all gene sets to be independent. By definition, all genes annotated to a GO term are also annotated to its ancestors. Because of this, the enrichment approach counts each gene multiple times by propagating it through the GO hierarchy from the most specific term the gene is associated with, all the way to the root of the ontology. This introduces redundancy in the analysis and reports many general and non-informative terms as significant. To overcome this limitation, iPathwayGuide allows users to use two more sophisticated pruning methods: *high-specificity pruning* and *smallest common denominator pruning*. The **high-specificity** pruning method *identifies the most specific gene sets* that are significantly associated with the set of DE genes. Let us consider, BP1 = “induction of apoptosis by intracellular signals” and BP2 = “induction of apoptosis by extracellular signals,” which are two of the children of BP3 = “induction of apoptosis.” If enough DE genes are associated with BP1 and BP2, the high-specificity pruning will report them as significant. The **smallest common denominator** pruning method *identifies the gene sets that best encapsulate the set of DE genes*, at times consolidating significance of two or more specific terms into their common parent. In the example above, this pruning method might report BP3 as significant because it is the most specific biological term that would include all DE genes that make both BP1 and BP2 significant.

5.2. Biological Processes

Table 5.2.1: Top identified Biological Processes gene sets. Only the top scoring Biological Processes gene sets are described below the table.

Gene Set	p-value	p-value (FDR)	p-value (Bonferroni)

5.3. Cellular Components

Table 5.3.1: Top identified Cellular Components gene sets. Only the top scoring Cellular Components gene sets are described below the table.

Gene Set	p-value	p-value (FDR)	p-value (Bonferroni)

5.4. Molecular Functions

Table 5.4.1: Top identified Molecular Functions gene sets. Only the top scoring Molecular Functions gene sets are described below the table.

Gene Set	p-value	p-value (FDR)	p-value (Bonferroni)

6. MsigDB

6.1. Methods

For each Gene Set (GO) term (Ashburner *et al*., 2002; Gene Ontology Consortium, 2004), the number of differentially expressed (DE) genes to the term is compared to the number of DE genes expected just by chance. iPathwayGuide uses an over-representation approach to compute the statistical

significance of observing at least the given number of DE genes. The p-value is computed using the hypergeometric distribution as described for pORA in the Pathway Analysis section. This p-value is corrected for multiple comparisons using FDR and Bonferroni.

The classical enrichment method used above considers all gene sets to be independent. By definition, all genes annotated to a GO term are also annotated to its ancestors. Because of this, the enrichment approach counts each gene multiple times by propagating it through the GO hierarchy from the most specific term the gene is associated with, all the way to the root of the ontology. This introduces redundancy in the analysis and reports many general and non-informative terms as significant. To overcome this limitation, iPathwayGuide allows users to use two more sophisticated pruning methods: *high-specificity pruning* and *smallest common denominator pruning*. The **high-specificity** pruning method *identifies the most specific gene sets* that are significantly associated with the set of DE genes. Let us consider, BP1 = “induction of apoptosis by intracellular signals” and BP2 = “induction of apoptosis by extracellular signals,” which are two of the children of BP3 = “induction of apoptosis.” If enough DE genes are associated with BP1 and BP2, the high-specificity pruning will report them as significant. The **smallest common denominator** pruning method *identifies the gene sets that best encapsulate the set of DE genes*, at times consolidating significance of two or more specific terms into their common parent. In the example above, this pruning method might report BP3 as significant because it is the most specific biological term that would include all DE genes that make both BP1 and BP2 significant.

6.2. Cell types

Table 6.2.1: Top identified Cell types gene sets. Only the top scoring Cell types gene sets are described below the table.

Gene Set	p-value	p-value (FDR)	p-value (Bonferroni)
FAN EMBRYONIC CTX BIG GROUPS BRAIN IMMUNE	0.00007071	0.03182	0.03182
DESCARTES FETAL EYE CORNEAL AND CONJUNCTIVAL EPITHELIAL CELLS	0.0001534	0.03453	0.06905
HE LIM SUN FETAL LUNG C2 PLATELET CELL	0.0004544	0.06817	0.2045
ZHENG CORD BLOOD C6 HSC MULTIPOTENT PROGENITOR	0.001135	0.1277	0.5107
HE LIM SUN FETAL LUNG C3 DEFINITIVE RETICULOCYTE	0.001866	0.1543	0.8397

FAN EMBRYONIC CTX BIG GROUPS BRAIN IMMUNE (11771)

In this experiment, the algorithm identified **6** differentially expressed gene(s) out of ALL **148** gene(s).

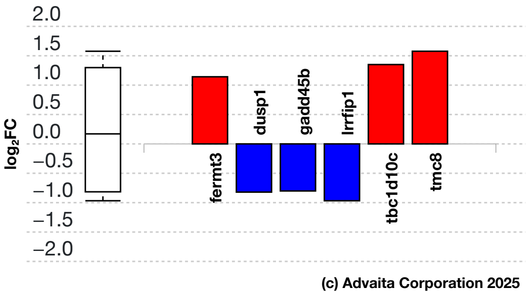

Fig. 6.2.1: Gene measured expression bar plot: *All the differentially expressed genes that are annotated to FAN EMBRYONIC CTX BIG GROUPS BRAIN IMMUNE are ranked based on their absolute value of log fold change. The plot is limited to the top 5 genes out of a total of 6 differentially expressed genes. Upregulated genes are shown in red, downregulated genes are shown in blue. The box and whisker plot on the left summarizes the distribution of all the differentially expressed genes that are annotated to this gene set. The box represents the 1st quartile, the median and the 3rd quartile, while the outliers are represented by circles.*

DESCARTES FETAL EYE CORNEAL AND CONJUNCTIVAL EPITHELIAL CELLS (12212)

Marker genes curated from the annotated cluster as represented in the Descartes Human Gene Expression During Development database In this experiment, the algorithm identified **7** differentially expressed gene(s) out of ALL **244** gene(s).

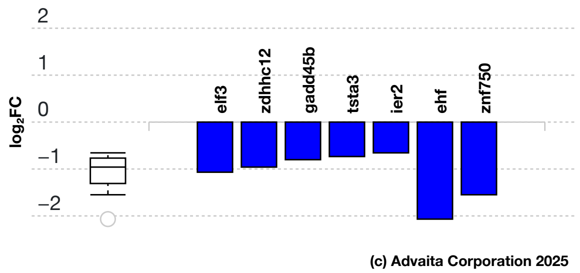

Fig. 6.2.2: Gene measured expression bar plot: *All the differentially expressed genes that are annotated to DESCARTES FETAL EYE CORNEAL AND CONJUNCTIVAL EPITHELIAL CELLS are ranked based on their absolute value of log fold change. The plot is limited to the top 5 genes out of a total of 7 differentially expressed genes. Upregulated genes are shown in red, downregulated genes are shown in blue. The box and whisker plot on the left summarizes the distribution of all the differentially expressed genes that are annotated to this gene set. The box represents the 1st quartile, the median and the 3rd quartile, while the outliers are represented by circles.*

HE LIM SUN FETAL LUNG C2 PLATELET CELL (12536)

Platelet In this experiment, the algorithm identified **8** differentially expressed gene(s) out of ALL **384** gene(s).

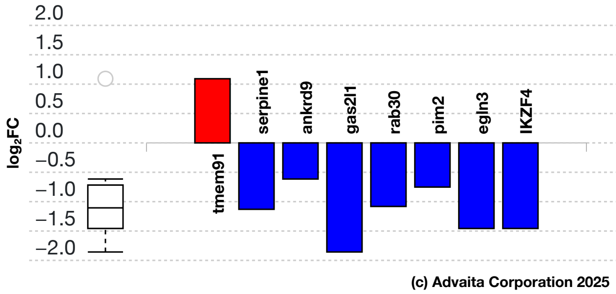

Fig. 6.2.3: Gene measured expression bar plot: *All the differentially expressed genes that are annotated to HE LIM SUN FETAL LUNG C2 PLATELET CELL are ranked based on their absolute value of log fold change. The plot is limited to the top 5 genes out of a total of 8 differentially expressed genes. Upregulated genes are shown in red, downregulated genes are shown in blue. The box and whisker plot on the left summarizes the distribution of all the differentially expressed genes that are annotated to this gene set. The box represents the 1st quartile, the median and the 3rd quartile, while the outliers are represented by circles.*

ZHENG CORD BLOOD C6 HSC MULTIPOTENT PROGENITOR (11961)

In this experiment, the algorithm identified **4** differentially expressed gene(s) out of ALL **97** gene(s).

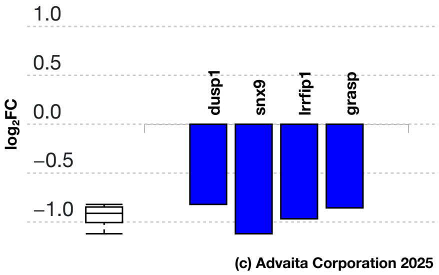

Fig. 6.2.4: Gene measured expression bar plot: *All the differentially expressed genes that are annotated to ZHENG CORD BLOOD C6 HSC MULTIPOTENT PROGENITOR are ranked based on their absolute value of log fold change. The plot is limited to the top 5 genes out of a total of 4 differentially expressed genes. Upregulated genes are shown in red, downregulated genes are shown in blue. The box and whisker plot on the left summarizes the distribution of all the differentially expressed genes that are annotated to this gene set. The box represents the 1st quartile, the median and the 3rd quartile, while the outliers are represented by circles.*

HE LIM SUN FETAL LUNG C3 DEFINITIVE RETICULOCYTE (12550)

Definitive reticulocyte In this experiment, the algorithm identified **4** differentially expressed gene(s) out of ALL **111** gene(s).

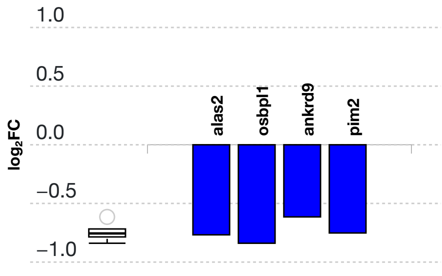

Fig. 6.2.5: Gene measured expression bar plot: *All the differentially expressed genes that are annotated to HE LIM SUN FETAL LUNG C3 DEFINITIVE RETICULOCYTE are ranked based on their absolute value of log fold change. The plot is limited to the top 5 genes out of a total of 4 differentially expressed genes. Upregulated genes are shown in red, downregulated genes are shown in blue. The box and whisker plot on the left summarizes the distribution of all the differentially expressed genes that are annotated to this gene set. The box represents the 1st quartile, the median and the 3rd quartile, while the outliers are represented by circles.*

6.3. Hallmark signatures

Table 6.3.1: Top identified Hallmark signatures gene sets. Only the top scoring Hallmark signatures gene sets are described below the table.

Gene Set	p-value	p-value (FDR)	p-value (Bonferroni)
HALLMARK TNFA SIGNALING VIA NFKB	0.001981	0.0614	0.0614
HALLMARK SPERMATOGENESIS	0.06332	0.6215	1
HALLMARK UV RESPONSE DN	0.07388	0.6215	1
HALLMARK IL2 STAT5 SIGNALING	0.1532	0.6215	1
HALLMARK GLYCOLYSIS	0.1548	0.6215	1

HALLMARK TNFA SIGNALING VIA NFKB (6309)

Genes regulated by NF kB in response to TNF GeneID 7124 In this experiment, the algorithm identified **6** differentially expressed gene(s) out of ALL **200** gene(s).

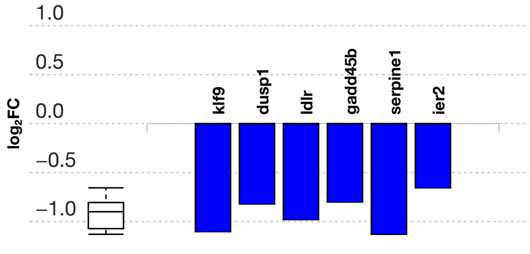

Fig. 6.3.1: Gene measured expression bar plot: *All the differentially expressed genes that are annotated to HALLMARK TNFA SIGNALING VIA NFKB are ranked based on their absolute value of log fold change. The plot is limited to the top 5 genes out of a total of 6 differentially expressed genes. Upregulated genes are shown in red, downregulated genes are shown in blue. The box and whisker plot on the left summarizes the distribution of all the differentially expressed genes that are annotated to this gene set. The box represents the 1st quartile, the median and the 3rd quartile, while the outliers are represented by circles.*

HALLMARK SPERMATOGENESIS (6355)

Genes up regulated during production of male gametes sperm as in spermatogenesis In this experiment, the algorithm identified **3** differentially expressed gene(s) out of ALL **135** gene(s).

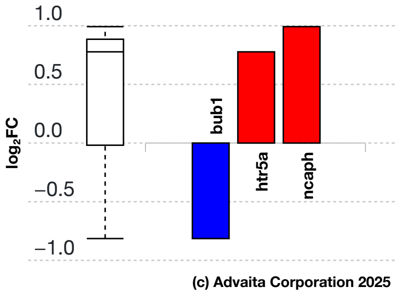

Fig. 6.3.2: Gene measured expression bar plot: *All the differentially expressed genes that are annotated to HALLMARK SPERMATOGENESIS are ranked based on their absolute value of log fold change. The plot is limited to the top 5 genes out of a total of 3 differentially expressed genes. Upregulated genes are shown in red, downregulated genes are shown in blue. The box and whisker plot on the left summarizes the distribution of all the differentially expressed genes that are annotated to this gene set. The box represents the 1st quartile, the median and the 3rd quartile, while the outliers are represented by circles.*

HALLMARK UV RESPONSE DN (6347)

Genes down regulated in response to ultraviolet UV radiation In this experiment, the algorithm identified **3** differentially expressed gene(s) out of ALL **144** gene(s).

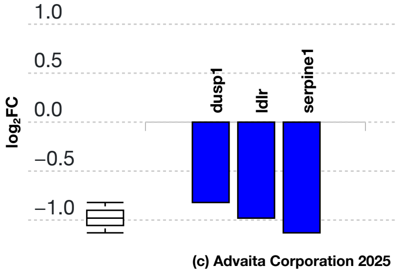

Fig. 6.3.3: Gene measured expression bar plot: *All the differentially expressed genes that are annotated to HALLMARK UV RESPONSE DN are ranked based on their absolute value of log fold change. The plot is limited to the top 5 genes out of a total of 3 differentially expressed genes. Upregulated genes are shown in red, downregulated genes are shown in blue. The box and whisker plot on the left summarizes the distribution of all the differentially expressed genes that are annotated to this gene set. The box represents the 1st quartile, the median and the 3rd quartile, while the outliers are represented by circles.*

HALLMARK IL2 STAT5 SIGNALING (6351)

Genes up regulated by STAT5 in response to IL2 stimulation In this experiment, the algorithm identified **3** differentially expressed gene(s) out of ALL **199** gene(s).

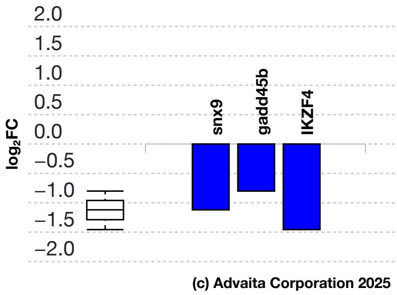

Fig. 6.3.4: Gene measured expression bar plot: *All the differentially expressed genes that are annotated to HALLMARK IL2 STAT5 SIGNALING are ranked based on their absolute value of log fold change. The plot is limited to the top 5 genes out of a total of 3 differentially expressed genes. Upregulated genes are shown in red, downregulated genes are shown in blue. The box and whisker plot on the left summarizes the distribution of all the differentially expressed genes that are annotated to this gene set. The box represents the 1st quartile, the median and the 3rd quartile, while the outliers are represented by circles.*

HALLMARK GLYCOLYSIS (6343)

Genes encoding proteins involved in glycolysis and gluconeogenesis In this experiment, the algorithm identified **3** differentially expressed gene(s) out of ALL **200** gene(s).

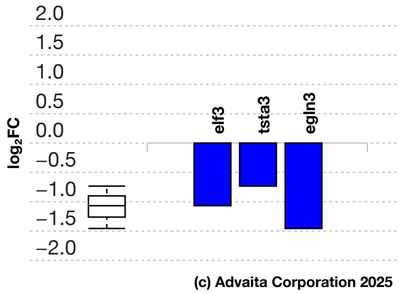

Fig. 6.3.5: Gene measured expression bar plot: *All the differentially expressed genes that are annotated to HALLMARK GLYCOLYSIS are ranked based on their absolute value of log fold change. The plot is limited to the top 5 genes out of a total of 3 differentially expressed genes. Upregulated genes are shown in red, downregulated genes are shown in blue. The box and whisker plot on the left summarizes the distribution of all the differentially expressed genes that are annotated to this gene set. The box represents the 1st quartile, the median and the 3rd quartile, while the outliers are represented by circles.*

6.4. Immune signatures

Table 6.4.1: Top identified Immune signatures gene sets. Only the top scoring Immune signatures gene sets are described below the table.

Gene Set	p-value	p-value (FDR)	p-value (Bonferroni)
GSE42724 MEMORY VS B1 BCELL DN	0.0004498	0.2436	1
GSE17721 LPS VS POLYIC 4H BMDC UP	0.0004872	0.2436	1
GSE22589 SIV VS HIV AND SIV INFECTED DC DN	0.0004872	0.2436	1
GSE23308 WT VS MINERALCORTICOID REC KO MACROPHAGE DN	0.0004872	0.2436	1
GSE39152 SPLEEN CD103 NEG VS BRAIN CD103 POS MEMORY CD8 TCELL DN	0.0004872	0.2436	1

GSE42724 MEMORY VS B1 BCELL DN (11638)

Genes down regulated in B lymphocytes memory versus B1 In this experiment, the algorithm identified **6** differentially expressed gene(s) out of ALL **197** gene(s).

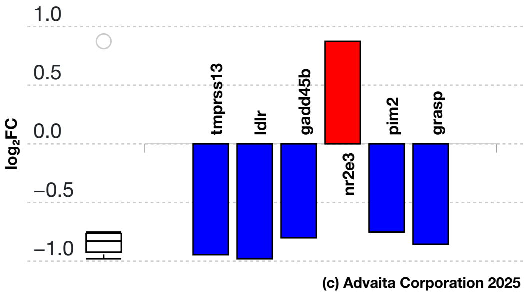

Fig. 6.4.1: Gene measured expression bar plot: *All the differentially expressed genes that are annotated to GSE42724 MEMORY VS B1 BCELL DN are ranked based on their absolute value of log fold change. The plot is limited to the top 5 genes out of a total of 6 differentially expressed genes. Upregulated genes are shown in red, downregulated genes are shown in blue. The box and whisker plot on the left summarizes the distribution of all the differentially expressed genes that are annotated to this gene set. The box represents the 1st quartile, the median and the 3rd quartile, while the outliers are represented by circles.*

GSE17721 LPS VS POLYIC 4H BMDC UP (7244)

Genes up regulated in comparison of dendritic cells DC stimulated with LPS TLR4 agonist at 4 h versus DC cells stimulated with poly I C TLR3 agonist at 4 h In this experiment, the algorithm identified **6** differentially expressed gene(s) out of ALL **200** gene(s).

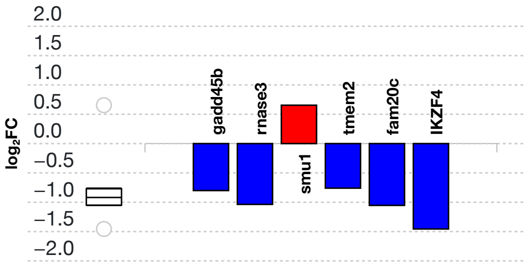

Fig. 6.4.2: Gene measured expression bar plot: *All the differentially expressed genes that are annotated to GSE17721 LPS VS POLYIC 4H BMDC UP are ranked based on their absolute value of log fold change. The plot is limited to the top 5 genes out of a total of 6 differentially expressed genes. Upregulated genes are shown in red, downregulated genes are shown in blue. The box and whisker plot on the left summarizes the distribution of all the differentially expressed genes that are annotated to this gene set. The box represents the 1st quartile, the median and the 3rd quartile, while the outliers are represented by circles.*

GSE22589 SIV VS HIV AND SIV INFECTED DC DN (10275)

Genes down regulated in monocyte derived dendritic cells infected by SIV versus HIV and SIV In this experiment, the algorithm identified **6** differentially expressed gene(s) out of ALL **200** gene(s).

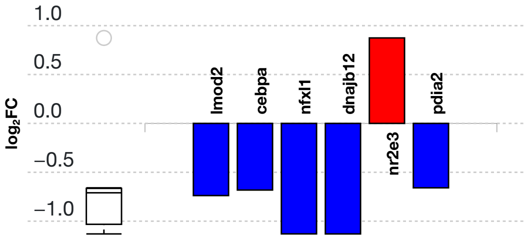

Fig. 6.4.3: Gene measured expression bar plot: *All the differentially expressed genes that are annotated to GSE22589 SIV VS HIV AND SIV INFECTED DC DN are ranked based on their absolute value of log fold change. The plot is limited to the top 5 genes out of a total of 6 differentially expressed genes. Upregulated genes are shown in red, downregulated genes are shown in blue. The box and whisker plot on the left summarizes the distribution of all the differentially expressed genes that are annotated to this gene set. The box represents the 1st quartile, the median and the 3rd quartile, while the outliers are represented by circles.*

GSE23308 WT VS MINERALCORTICOID REC KO MACROPHAGE DN (10232)

Genes down regulated in macrophages wildtype versus NR3C2 GeneID 4306 knockout In this experiment, the algorithm identified **6** differentially expressed gene(s) out of ALL **200** gene(s).

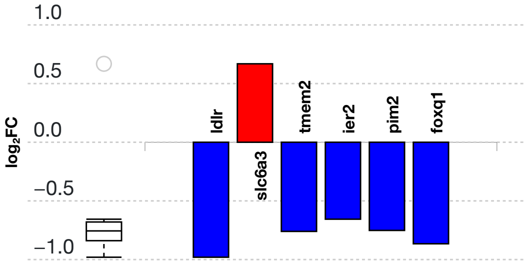

Fig. 6.4.4: Gene measured expression bar plot: *All the differentially expressed genes that are annotated to GSE23308 WT VS MINERALCORTICOID REC KO MACROPHAGE DN are ranked based on their absolute value of log fold change. The plot is limited to the top 5 genes out of a total of 6 differentially expressed genes. Upregulated genes are shown in red, downregulated genes are shown in blue. The box and whisker plot on the left summarizes the distribution of all the differentially expressed genes that are annotated to this gene set. The box represents the 1st quartile, the median and the 3rd quartile, while the outliers are represented by circles.*

GSE39152 SPLEEN CD103 NEG VS BRAIN CD103 POS MEMORY CD8 TCELL DN (11129)

Genes down regulated in memory CD8 T cells ITGAE GeneID 3682 from spleen versus ITGAE GeneID 3682 from brain In this experiment, the algorithm identified **6** differentially expressed gene(s) out of ALL **200** gene(s).

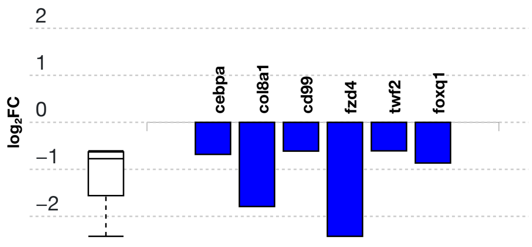

Fig. 6.4.5: Gene measured expression bar plot: *All the differentially expressed genes that are annotated to GSE39152 SPLEEN CD103 NEG VS BRAIN CD103 POS MEMORY CD8 TCELL DN are ranked based on their absolute value of log fold change. The plot is limited to the top 5 genes out of a total of 6 differentially expressed genes. Upregulated genes are shown in red, downregulated genes are shown in blue. The box and whisker plot on the left summarizes the distribution of all the differentially expressed genes that are annotated to this gene set. The box represents the 1st quartile, the median and the 3rd quartile, while the outliers are represented by circles.*

6.5. Oncogenic signatures

Table 6.5.1: Top identified Oncogenic signatures gene sets. Only the top scoring Oncogenic signatures gene sets are described below the table.

Gene Set	p-value	p-value (FDR)	p-value (Bonferroni)
CORDENONSI YAP CONSERVED SIGNATURE	0.005299	0.4718	0.673
NRL DN.V1 UP	0.01035	0.4718	1
CRX NRL DN.V1 UP	0.01115	0.4718	1
CAHOY ASTROGLIAL	0.02425	0.5139	1
AKT UP MTOR DN.V1 DN	0.02794	0.5139	1

CORDENONSI YAP CONSERVED SIGNATURE (6525)

YAP conserved signature In this experiment, the algorithm identified **3** differentially expressed gene(s) out of ALL **57** gene(s).

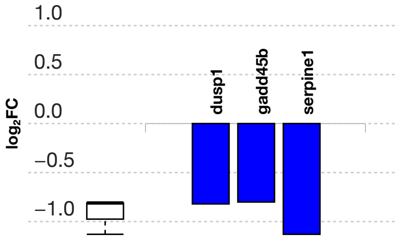

Fig. 6.5.1: Gene measured expression bar plot: *All the differentially expressed genes that are annotated to CORDENONSI YAP CONSERVED SIGNATURE are ranked based on their absolute value of log fold change. The plot is limited to the top 5 genes out of a total of 3 differentially expressed genes. Upregulated genes are shown in red, downregulated genes are shown in blue. The box and whisker plot on the left summarizes the distribution of all the differentially expressed genes that are annotated to this gene set. The box represents the 1st quartile, the median and the 3rd quartile, while the outliers are represented by circles.*

NRL DN.V1 UP (6478)

Genes up regulated in retina cells from NRL Gene ID 4901 knockout mice In this experiment, the algorithm identified **4** differentially expressed gene(s) out of ALL **137** gene(s).

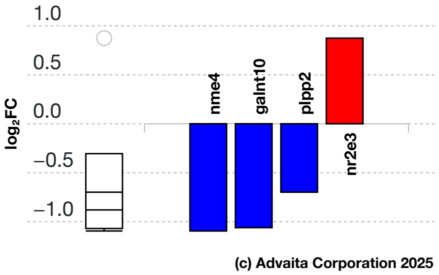

Fig. 6.5.2: Gene measured expression bar plot: *All the differentially expressed genes that are annotated to NRL DN.V1 UP are ranked based on their absolute value of log fold change. The plot is limited to the top 5 genes out of a total of 4 differentially expressed genes. Upregulated genes are shown in red, downregulated genes are shown in blue. The box and whisker plot on the left summarizes the distribution of all the differentially expressed genes that are annotated to this gene set. The box represents the 1st quartile, the median and the 3rd quartile, while the outliers are represented by circles.*

CRX NRL DN.V1 UP (6476)

Genes up regulated in retina cells from CRX and NRL Gene ID 1406 4901 double knockout mice In this experiment, the algorithm identified **4** differentially expressed gene(s) out of ALL **140** gene(s).

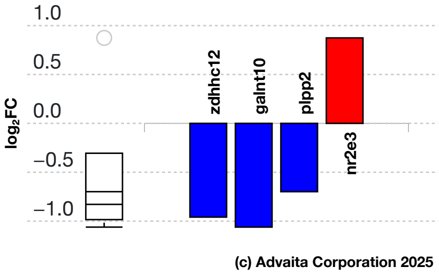

Fig. 6.5.3: Gene measured expression bar plot: *All the differentially expressed genes that are annotated to CRX NRL DN.V1 UP are ranked based on their absolute value of log fold change. The plot is limited to the top 5 genes out of a total of 4 differentially expressed genes. Upregulated genes are shown in red, downregulated genes are shown in blue. The box and whisker plot on the left summarizes the distribution of all the differentially expressed genes that are annotated to this gene set. The box represents the 1st quartile, the median and the 3rd quartile, while the outliers are represented by circles.*

CAHOY ASTROGLIAL (6486)

Genes up regulated in astrogia cells In this experiment, the algorithm identified **3** differentially expressed gene(s) out of ALL **100** gene(s).

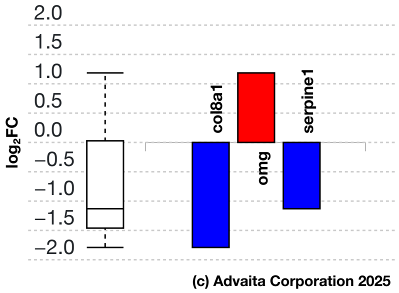

Fig. 6.5.4: Gene measured expression bar plot: *All the differentially expressed genes that are annotated to CAHOY ASTROGLIAL are ranked based on their absolute value of log fold change. The plot is limited to the top 5 genes out of a total of 3 differentially expressed genes. Upregulated genes are shown in red, downregulated genes are shown in blue. The box and whisker plot on the left summarizes the distribution of all the differentially expressed genes that are annotated to this gene set. The box represents the 1st quartile, the median and the 3rd quartile, while the outliers are represented by circles.*

AKT UP MTOR DN.V1 DN (6383)

Genes down regulated by everolimus PubChem 6442177 in mouse prostate tissue transgenically expressing human AKT1 gene Gene ID 207 vs untreated controls In this experiment, the algorithm identified **4** differentially expressed gene(s) out of ALL **185** gene(s).

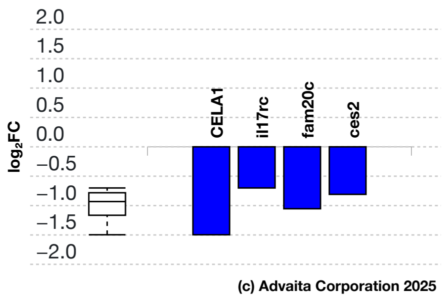

Fig. 6.5.5: Gene measured expression bar plot: *All the differentially expressed genes that are annotated to AKT UP MTOR DN.V1 DN are ranked based on their absolute value of log fold change. The plot is limited to the top 5 genes out of a total of 4 differentially expressed genes. Upregulated genes are shown in red, downregulated genes are shown in blue. The box and whisker plot on the left summarizes the distribution of all the differentially expressed genes that are annotated to this gene set. The box represents the 1st quartile, the median and the 3rd quartile, while the outliers are represented by circles.*

7. Predicted Upstream Regulator Analysis - miRNAs

7.1. Methods

The prediction of active miRNAs (Friedman *et al*., 2009; Lewis *et al*., 2005) is based on enrichment of differentially downregulated target genes of the miRNAs. In general, miRNAs have an inhibitory effect on their targets. Therefore, for any given miRNA the method computes the ratio between the number of differentially downregulated targets and all differentially expressed targets, and compares it to the ratio of all downwardly expressed targets to all targets. Overall, iPathwayGuide calculates the probability of observing at least the number of differentially downregulated target genes for a given miRNA just by chance. This p-value is computed using the hypergeometric distribution as described for pORA in the Pathway Analysis section.

7.2. Results

Table 7.2.1: Top identified miRNAs

miRNA Name	p-value	p-value (FDR)	p-value (Bonferroni)
hsa-miR-124-3p	**3.573e-4**	0.099	0.108
hsa-miR-144-3p	**0.002**	0.099	0.622
hsa-miR-101-3p	**0.003**	0.099	0.943
hsa-miR-9-5p	**0.004**	0.099	1.000
hsa-miR-17-5p	**0.004**	0.099	1.000

hsa-miR-124-3p (MIMAT0000422)

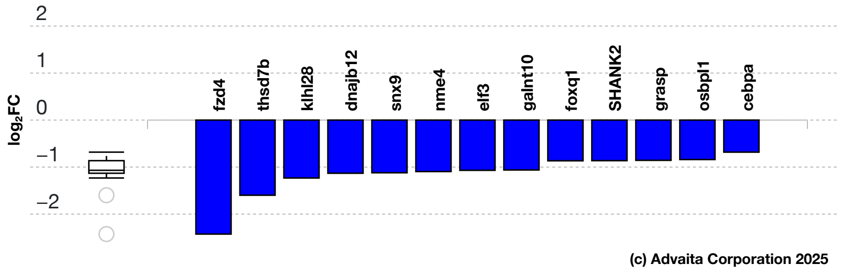

Fig. 7.2.1: Gene measured expression bar plot: *All the differentially expressed genes that are targeted by hsa-miR-124-3p are ranked based on their measured expression change (most downregulated to upregulated). The downregulated genes are shown in blue, and the upregulated ones are shown in red (where applicable). Out of all the differentially expressed target genes, 13 were found to be downregulated. The box and whisker plot on the left summarizes the distribution of all the differentially expressed genes targeted by this miRNA. The box represents the 1st quartile, the median and the 3rd quartile, while the outliers are represented by circles.*

hsa-miR-144-3p (MIMAT0000436)

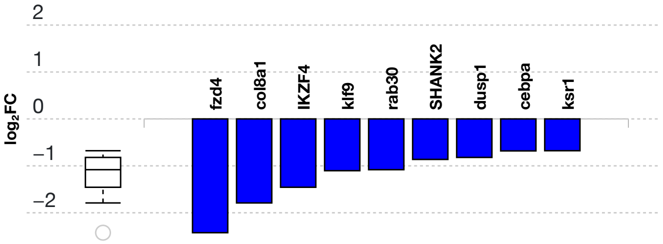

(c) Advaita Corporation 2025

Fig. 7.2.2: Gene measured expression bar plot: *All the differentially expressed genes that are targeted by hsa-miR-144-3p are ranked based on their measured expression change (most downregulated to upregulated). The downregulated genes are shown in blue, and the upregulated ones are shown in red (where applicable). Out of all the differentially expressed target genes, 9 were found to be downregulated. The box and whisker plot on the left summarizes the distribution of all the differentially expressed genes targeted by this miRNA. The box represents the 1st quartile, the median and the 3rd quartile, while the outliers are represented by circles.*

hsa-miR-101-3p (MIMAT0000099)

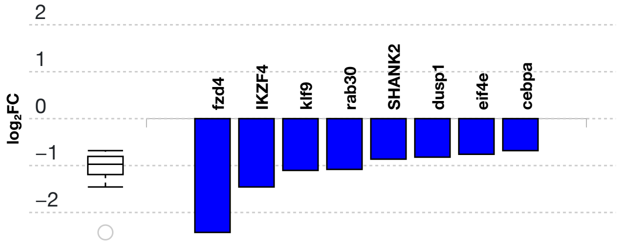

(c) Advaita Corporation 2025

Fig. 7.2.3: Gene measured expression bar plot: *All the differentially expressed genes that are targeted by hsa-miR-101-3p are ranked based on their measured expression change (most downregulated to upregulated). The downregulated genes are shown in blue, and the upregulated ones are shown in red (where applicable). Out of all the differentially expressed target genes, 8 were found to be downregulated. The box and whisker plot on the left summarizes the distribution of all the differentially expressed genes targeted by this miRNA. The box represents the 1st quartile, the median and the 3rd quartile, while the outliers are represented by circles.*

hsa-miR-9-5p (MIMAT0000441)

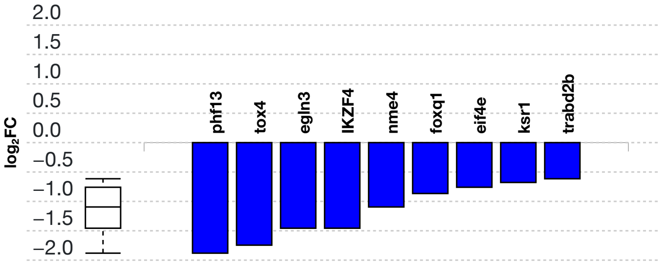

(c) Advaita Corporation 2025

Fig. 7.2.4: Gene measured expression bar plot: *All the differentially expressed genes that are targeted by hsa-miR-9-5p are ranked based on their measured expression change (most downregulated to upregulated). The downregulated genes are shown in blue, and the upregulated ones are shown in red (where applicable). Out of all the differentially expressed target genes, 9 were found to be downregulated. The box and whisker plot on the left summarizes the distribution of all the differentially expressed genes targeted by this miRNA. The box represents the 1st quartile, the median and the 3rd quartile, while the outliers are represented by circles.*

hsa-miR-17-5p (MIMAT0000070)

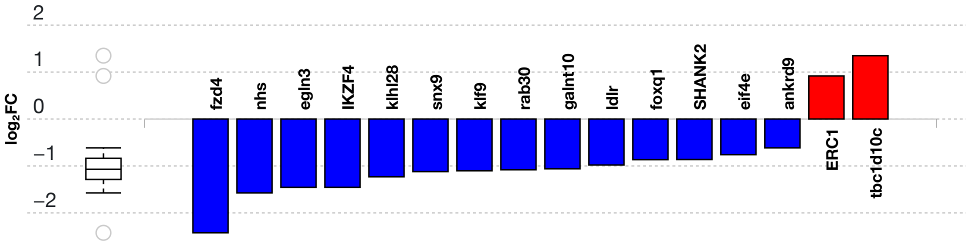

(c) Advaita Corporation 2025

Fig. 7.2.5: Gene measured expression bar plot: *All the differentially expressed genes that are targeted by hsa-miR-17-5p are ranked based on their measured expression change (most downregulated to upregulated). The downregulated genes are shown in blue, and the upregulated ones are shown in red (where applicable). Out of all the differentially expressed target genes, 14 were found to be downregulated. The box and whisker plot on the left summarizes the distribution of all the differentially expressed genes targeted by this miRNA. The box represents the 1st quartile, the median and the 3rd quartile, while the outliers are represented by circles.*

8. Predicted Upstream Regulator Analysis - Genes

8.1. Methods

The prediction of upstream regulators is based on two types of information: i) the enrichment of differentially expressed genes from the experiment and ii) a network of regulatory interactions from our proprietary knowledge base (see the report information for details). The network is a directed graph in which the nodes represent genes, and the edges represent regulatory interactions between two genes. A signed edge in this graph consists of a source gene, a target gene, and a sign to indicate the type of signal: activation (+) or inhibition (-). To create the network, the analysis selects only those edges observed in the literature with at least a medium confidence (evidence score greater than or equal to 400). The analysis considers two hypotheses:

HA. The upstream regulator is **activated** in the condition studied.
HI. The upstream regulator is **inhibited** in the condition studied.

The analysis divides the set of all the genes obtained from NCBI Gene database into several subsets based on the measurements in the experiment and the definitions shown in **Figure 5.1.1** and **Figure 5.1.2**. Let the sign of a measured DE gene be the sign of the log fold change value: (+) for up-regulated genes and (-) for down-regulated genes. A gene is a target gene if it corresponds to a node in the network that has at least one incoming edge. We define a *consistent gene* as a target DE gene such that the sign of the gene is consistent both with the type of the signal **and** with the hypothesis considered. Formally, by definition, a target DE gene *g* is consistent with Hypothesis HA if and only if an incoming edge *e* exists such that *sign(g) = sign(e)*. In other words, this describes the situation when the upstream regulator is predicted as activated, the signal is activation and the target DE gene is up-regulated, or the signal is inhibition and the target DE gene is down-regulated (see panel A in **Figure 5.1.1**). A target DE gene *g* is consistent with Hypothesis HI if and only if an incoming edge *e* exists such that *sign(g) ≠ sign(e)*. This second case captures the situation in which the upstream regulator is inhibited, the signal is inhibition and the target DE gene is up-regulated, or the signal is activation and the target DE gene is down-regulated (see panel B in **Figure 5.1.1**).

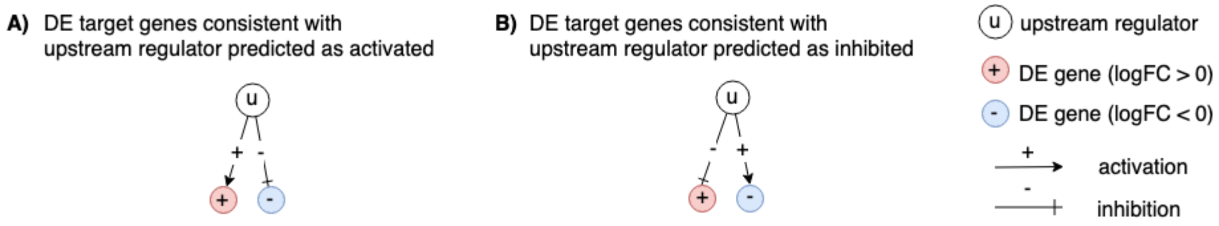

Fig. 8.1.1: Target genes consistent with the hypothesis considered: *In panel A, the signs of the DE genes match the signs of their respective incoming edges, increasing the likelihood that the upstream regulator u is activated. In panel B, the signs of the DE genes are opposite to the signs of their edges, increasing the likelihood that the upstream regulator u is inhibited.*

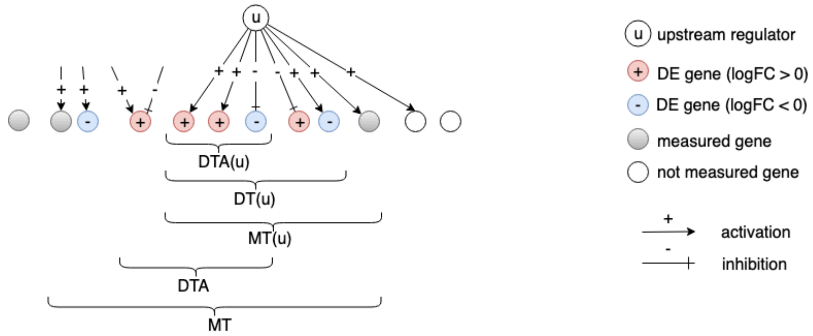

Fig. 8.1.2: *The set of all genes includes the set of measured genes that are also targets in the network, or Measured Targets (MT). We define the subset of "DE Targets consistent with the first hypothesis that the upstream regulators are Activated", DTA. For a selected upstream regulator u, we have the set of "Measured Targets of u" MT(u), "Differentially expressed Targets downstream of u" DT(u), and the set of "DE targets consistent with the hypothesis HA that u is Activated" DTA(u). The equivalent graphic for the hypothesis HI associated with DTI and DTI(u) is not shown.*

Upstream regulators Z-score

For both research hypotheses, the analysis computes a Z-score for each upstream regulator *z(u)* by iterating over the genes in DT(u) and their incoming edges *in(g)*. We can then compute the p-value corresponding to the z-score P_z as the one-tailed area under the probability density function for a normal distribution, N(0,1).

Upstream regulators predicted as activated

Here, the research hypothesis considers the upstream regulator as activated. For each upstream regulator *u*, the number of consistent DE genes downstream of *u*, DTA(u) is compared to the number of measured target genes expected to be both consistent and DE just by chance. iPathwayGuide uses an over-representation approach to compute the statistical significance of observing at least the given number of consistent DE genes. The p-value P_{act} is computed using the hypergeometric distribution (Draghici *et al*., 2003, Draghici 2011).

After computing a p-value for both types of evidence, P_z and P_{act}, we need to combine these two probabilities into one global probability value, P_G that is used to rank the upstream regulators and test the research hypothesis that the upstream regulators are predicted as activated in the condition studied. Since only a positive z-score indicates that the upstream regulator is predicted as activated, we only combine p-values for a positive z-score. Moreover, to avoid introducing false positives, only P_z for significant z-scores ($z \geq 2$) are combined. The analysis uses the standard Fisher's method to combine p-values into one test statistic (Fisher 1925).

Upstream regulators predicted as inhibited

In parallel with upstream regulators predicted as activated, we use P_{inh} and P_z to predict upstream regulators that are inhibited. Here, the research hypothesis states that the upstream regulators are inhibited in the conditions studied. For each upstream regulator *u*, the number of consistent DE genes downstream of *u*, DTI(u) is compared to the number of measured target genes expected to be both consistent and DE just by chance. Using the Fisher's method as above, the analysis combines P_{inh} and P_z, where P_z is considered only for significant negative z-scores ($z \leq -2$).

8.2. Results: upstream regulators predicted as activated

Upstream Regulator (u)	DTA(u)	DT(u)	p-value	p-value (FDR)	p-value (Bonferroni)
RFK	1	1	**0.005**	0.397	1.000
TGIF2	1	1	**0.005**	0.397	1.000
NACC1	1	1	**0.005**	0.397	1.000
APOF	1	1	**0.010**	0.397	1.000
PDE4B	1	1	**0.010**	0.397	1.000

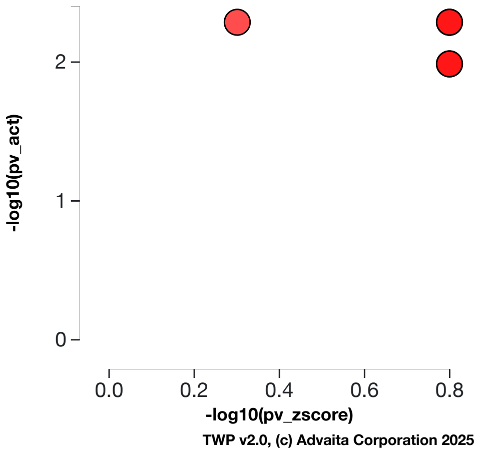

Table 8.2.1: Top upstream regulators predicted as activated. *For each upstream regulator u, the table shows the number of DE targets supporting the hypothesis that the regulator is activated DTA(u) the total number of DE genes downstream of u DT(u), the combined raw p-value, and the p-value corrected for multiple comparisons.* **Fig. 8.2.1: A two-way plot showing the top five upstream regulators predicted as activated.** *Dots representing upstream regulators are positioned using P_{zscore} on the horizontal axis, and using P_{act} on the vertical axis. P_{act} is the p-value based on the number of DE targets consistent with the typeof the incoming signal and with the selected hypothesis type. Upstream regulators with a significant combined p-value are shown in red. The size of each dot represents the number of consistent DE genes for that regulator.*

RFK (riboflavin kinase)

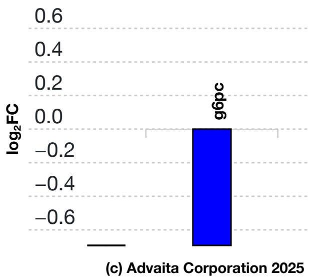

Fig. 8.2.3: Gene measured expression bar plot: *All the consistent differentially expressed genes that are targeted by RFK are ranked based on their absolute value of log fold change. Upregulated genes are shown in red, downregulated genes are shown in blue. The box and whisker plot on the left summarizes the distribution of all the consistent differentially expressed genes targeted by this upstream regulator. The box shows the 1st quartile, the median and the 3rd quartile, while the outliers are represented by circles.*

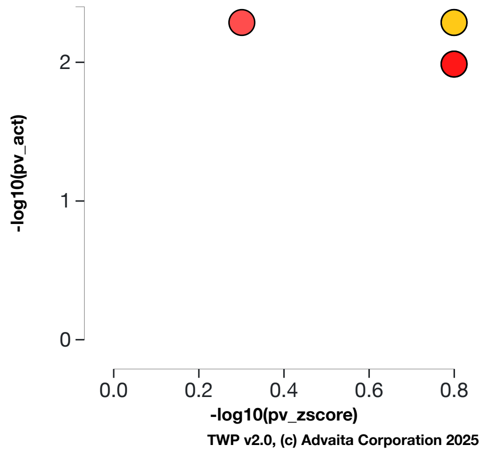

Fig. 8.2.4: Activation p-value vs zscore p-value: *RFK, riboflavin kinase, (yellow) is shown, using negative log of the activation and zscore p-values, along with the other most significant upstream regulators. The size of the dot represents the relative number of consistent DE genes, which for selected upstream regulator is 1.*

TGIF2 (TGFB induced factor homeobox 2)

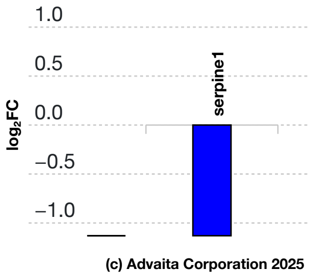

Fig. 8.2.5: Gene measured expression bar plot: *All the consistent differentially expressed genes that are targeted by TGIF2 are ranked based on their absolute value of log fold change. Upregulated genes are shown in red, downregulated genes are shown in blue. The box and whisker plot on the left summarizes the distribution of all the consistent differentially expressed genes targeted by this upstream regulator. The box shows the 1st quartile, the median and the 3rd quartile, while the outliers are represented by circles.*

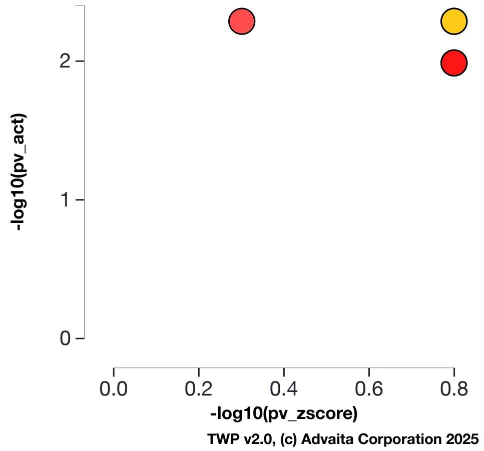

Fig. 8.2.6: Activation p-value vs zscore p-value: *TGIF2, TGFB induced factor homeobox 2, (yellow) is shown, using negative log of the activation and zscore p-values, along with the other most significant upstream regulators. The size of the dot represents the relative number of consistent DE genes, which for selected upstream regulator is 1.*

NACC1 (nucleus accumbens associated 1)

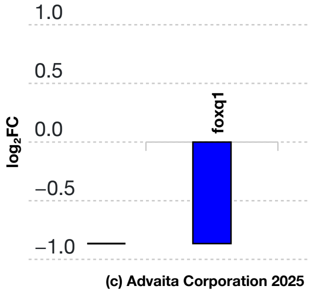

Fig. 8.2.7: Gene measured expression bar plot: *All the consistent differentially expressed genes that are targeted by NACC1 are ranked based on their absolute value of log fold change. Upregulated genes are shown in red, downregulated genes are shown in blue. The box and whisker plot on the left summarizes the distribution of all the consistent differentially expressed genes targeted by this upstream regulator. The box shows the 1st quartile, the median and the 3rd quartile, while the outliers are represented by circles.*

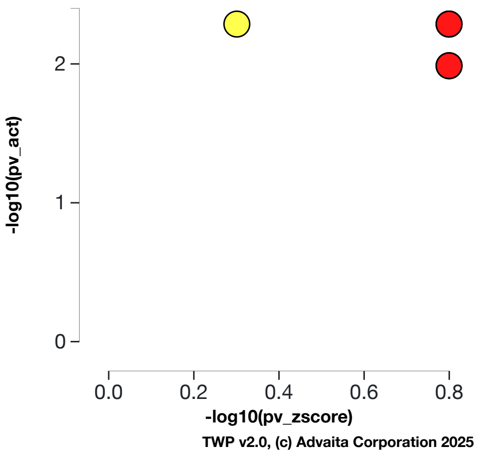

Fig. 8.2.8: Activation p-value vs zscore p-value: *NACC1, nucleus accumbens associated 1, (yellow) is shown, using negative log of the activation and zscore p-values, along with the other most significant upstream regulators. The size of the dot represents the relative number of consistent DE genes, which for selected upstream regulator is 1.*

APOF (apolipoprotein F)

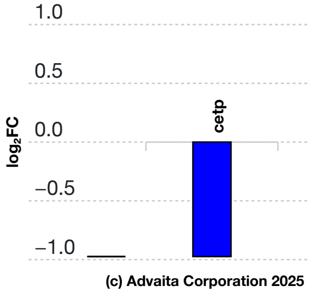

Fig. 8.2.9: Gene measured expression bar plot: *All the consistent differentially expressed genes that are targeted by APOF are ranked based on their absolute value of log fold change. Upregulated genes are shown in red, downregulated genes are shown in blue. The box and whisker plot on the left summarizes the distribution of all the consistent differentially expressed genes targeted by this upstream regulator. The box shows the 1st quartile, the median and the 3rd quartile, while the outliers are represented by circles.*

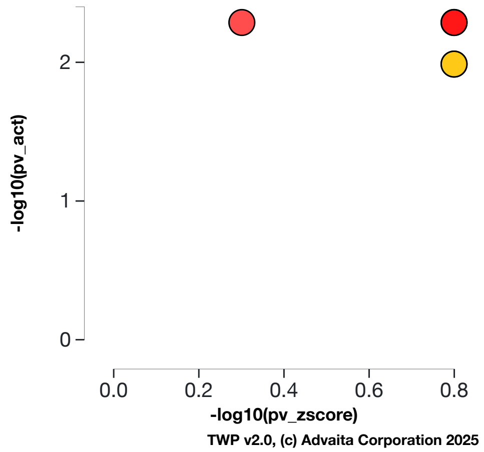

Fig. 8.2.10: Activation p-value vs zscore p-value: *APOF, apolipoprotein F, (yellow) is shown, using negative log of the activation and zscore p-values, along with the other most significant upstream regulators. The size of the dot represents the relative number of consistent DE genes, which for selected upstream regulator is 1.*

PDE4B (phosphodiesterase 4B)

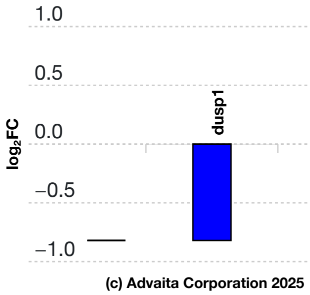

Fig. 8.2.11: Gene measured expression bar plot: *All the consistent differentially expressed genes that are targeted by PDE4B are ranked based on their absolute value of log fold change. Upregulated genes are shown in red, downregulated genes are shown in blue. The box and whisker plot on the left summarizes the distribution of all the consistent differentially expressed genes targeted by this upstream regulator. The box shows the 1st quartile, the median and the 3rd quartile, while the outliers are represented by circles.*

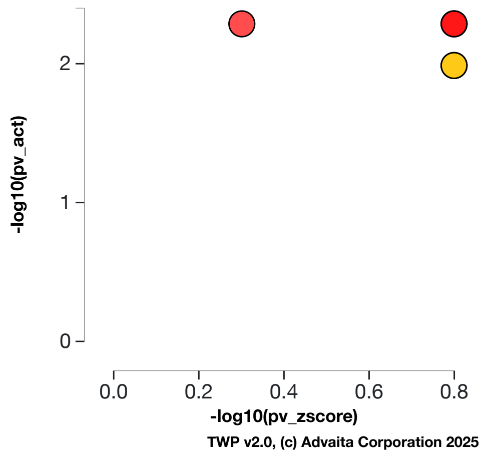

Fig. 8.2.12: Activation p-value vs zscore p-value: *PDE4B, phosphodiesterase 4B, (yellow) is shown, using negative log of the activation and zscore p-values, along with the other most significant upstream regulators. The size of the dot represents the relative number of consistent DE genes, which for selected upstream regulator is 1.*

8.3. Results: upstream regulators predicted as inhibited

Upstream Regulator (u)	DTI(u)	DT(u)	p-value	p-value (FDR)	p-value (Bonferroni)
EPAS1	2	2	0.002	0.167	0.664
HIF1A	3	3	0.002	0.167	0.830
GFPT2	1	1	0.008	0.167	1.000
RAB11FIP2	1	1	0.008	0.167	1.000
NACC1	1	1	0.008	0.167	1.000

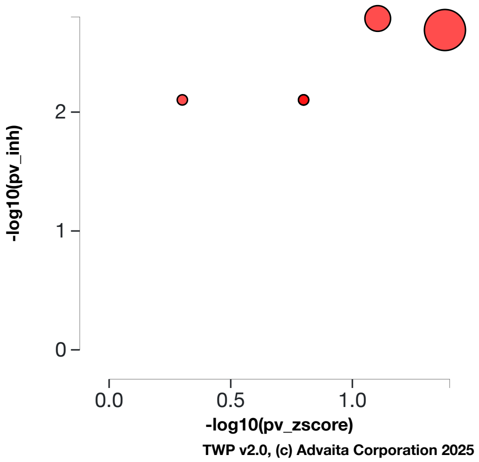

Table 8.3.1: Top upstream regulators predicted as inhibited. *For each upstream regulator u, the table shows the number of DE targets supporting the hypothesis that the regulator is inhibited DTI(u) the total number of DE genes downstream of u DT(u), the combined raw p-value, and the p-value corrected for multiple comparisons.* **Fig. 8.3.1: A two-way plot showing the top five upstream regulators predicted as inhibited.** *Dots representing upstream regulators are positioned using P_{zscore} on the horizontal axis, and using P_{inh} on the vertical axis. P_{inh} is the p-value based on the number of DE targets consistent with the typeof the incoming signal and with the selected hypothesis type. Upstream regulators with a significant combined p-value are shown in red. The size of each dot represents the number of consistent DE genes for that regulator.*

EPAS1 (endothelial PAS domain protein 1)

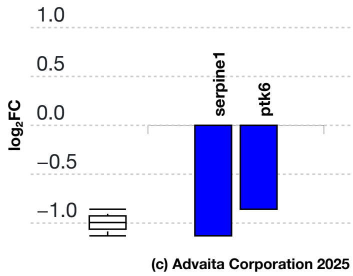

Fig. 8.3.13: Gene measured expression bar plot: *All the consistent differentially expressed genes that are targeted by EPAS1 are ranked based on their absolute value of log fold change. Upregulated genes are shown in red, downregulated genes are shown in blue. The box and whisker plot on the left summarizes the distribution of all the consistent differentially expressed genes targeted by this upstream regulator. The box shows the 1st quartile, the median and the 3rd quartile, while the outliers are represented by circles.*

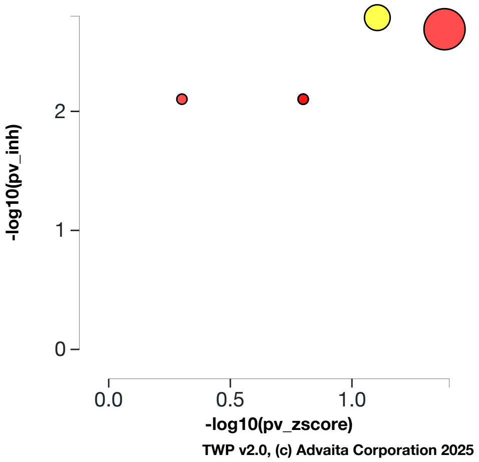

Fig. 8.3.14: Inhibition p-value vs zscore p-value: *EPAS1, endothelial PAS domain protein 1, (yellow) is shown, using negative log of the inhibition and zscore p-values, along with the other most significant upstream regulators. The size of the dot represents the relative number of consistent DE genes, which for selected upstream regulator is 2.*

HIF1A (hypoxia inducible factor 1 subunit alpha)

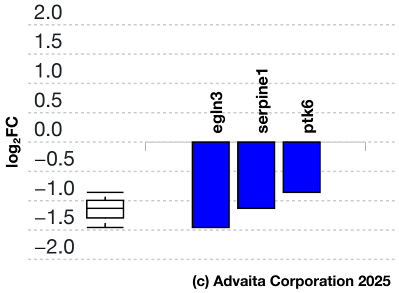

Fig. 8.3.15: Gene measured expression bar plot: *All the consistent differentially expressed genes that are targeted by HIF1A are ranked based on their absolute value of log fold change. Upregulated genes are shown in red, downregulated genes are shown in blue. The box and whisker plot on the left summarizes the distribution of all the consistent differentially expressed genes targeted by this upstream regulator. The box shows the 1st quartile, the median and the 3rd quartile, while the outliers are represented by circles.*

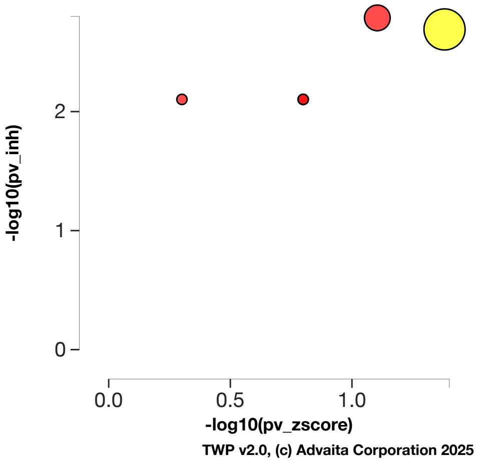

Fig. 8.3.16: Inhibition p-value vs zscore p-value: *HIF1A, hypoxia inducible factor 1 subunit alpha, (yellow) is shown, using negative log of the inhibition and zscore p-values, along with the other most significant upstream regulators. The size of the dot represents the relative number of consistent DE genes, which for selected upstream regulator is 3.*

GFPT2 (glutamine-fructose-6-phosphate transaminase 2)

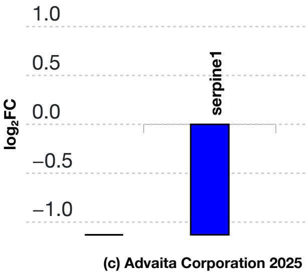

Fig. 8.3.17: Gene measured expression bar plot: *All the consistent differentially expressed genes that are targeted by GFPT2 are ranked based on their absolute value of log fold change. Upregulated genes are shown in red, downregulated genes are shown in blue. The box and whisker plot on the left summarizes the distribution of all the consistent differentially expressed genes targeted by this upstream regulator. The box shows the 1st quartile, the median and the 3rd quartile, while the outliers are represented by circles.*

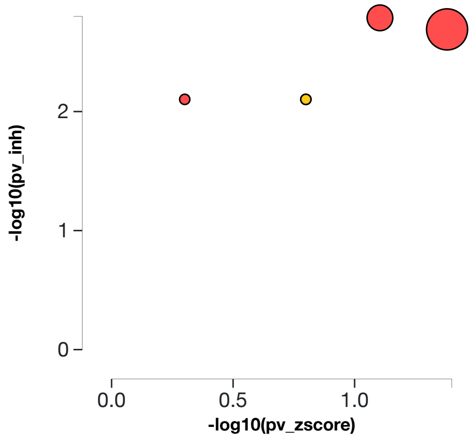

TWP v2.0, (c) Advaita Corporation 2025

Fig. 8.3.18: Inhibition p-value vs zscore p-value: *GFPT2, glutamine-fructose-6-phosphate transaminase 2, (yellow) is shown, using negative log of the inhibition and zscore p-values, along with the other most significant upstream regulators. The size of the dot represents the relative number of consistent DE genes, which for selected upstream regulator is 1.*

RAB11FIP2 (RAB11 family interacting protein 2)

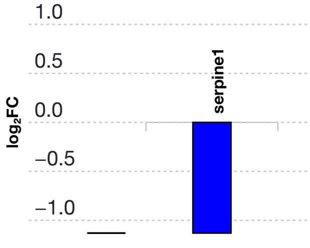

(c) Advaita Corporation 2025

Fig. 8.3.19: Gene measured expression bar plot: *All the consistent differentially expressed genes that are targeted by RAB11FIP2 are ranked based on their absolute value of log fold change. Upregulated genes are shown in red, downregulated genes are shown in blue. The box and whisker plot on the left summarizes the distribution of all the consistent differentially expressed genes targeted by this upstream regulator. The box shows the 1st quartile, the median and the 3rd quartile, while the outliers are represented by circles.*

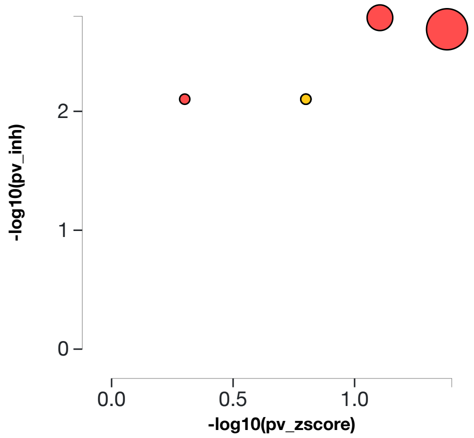

TWP v2.0, (c) Advaita Corporation 2025

Fig. 8.3.20: Inhibition p-value vs zscore p-value: *RAB11FIP2, RAB11 family interacting protein 2, (yellow) is shown, using negative log of the inhibition and zscore p-values, along with the other most significant upstream regulators. The size of the dot represents the relative number of consistent DE genes, which for selected upstream regulator is 1.*

NACC1 (nucleus accumbens associated 1)

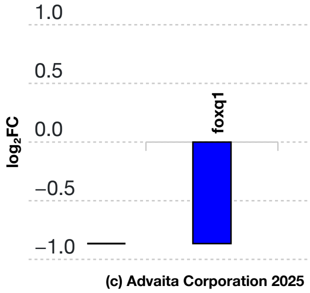

Fig. 8.3.21: Gene measured expression bar plot: *All the consistent differentially expressed genes that are targeted by NACC1 are ranked based on their absolute value of log fold change. Upregulated genes are shown in red, downregulated genes are shown in blue. The box and whisker plot on the left summarizes the distribution of all the consistent differentially expressed genes targeted by this upstream regulator. The box shows the 1st quartile, the median and the 3rd quartile, while the outliers are represented by circles.*

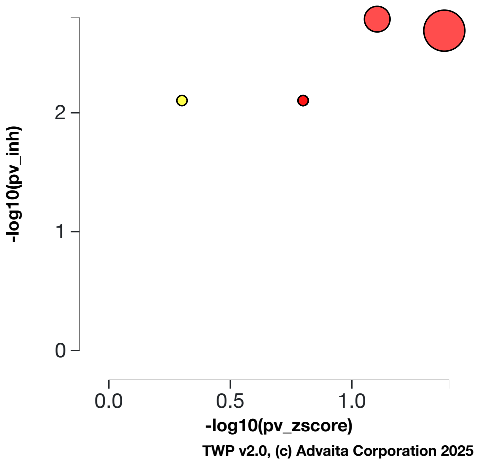

Fig. 8.3.22: Inhibition p-value vs zscore p-value: *NACC1, nucleus accumbens associated 1, (yellow) is shown, using negative log of the inhibition and zscore p-values, along with the other most significant upstream regulators. The size of the dot represents the relative number of consistent DE genes, which for selected upstream regulator is 1.*

9. Predicted Upstream Regulator Analysis – Chemicals, Drugs, Toxicants (CDTs)

9.1. Methods

The prediction of upstream Chemicals, Drugs, Toxicants (CDTs) is based on two types of information: i) the enrichment of differentially expressed genes from the experiment and ii) a network of interactions from the Advaita Knowledge Base (AKB v18.1). The network is a directed graph in which the source node represents either a chemical substance or compound (e.g. zinc), a drug (e.g. aspirin), or a toxicant (e.g. tobacco smoke). The generic abbreviation CDT will be used henceforth to designate any of these. The edges represent known effects that these CDTs have on various genes. A signed edge in this graph consists of a source CDT, a target gene, and a sign to indicate the type of effect: activation (+) or inhibition (-). The analysis considers two hypotheses:

HP. The upstream chemical, drug or toxicant is **present (or overly abundant)** in the condition studied.
HA. The upstream chemical, drug or toxicant is **absent (or insufficient)** in the condition studied.

The analysis divides the set of all the genes from AKB into several subsets based on the measurements in the experiment and the definitions shown in **Figure 6.1.1** and **Figure 6.1.2**. Let the sign of a measured DE gene be the sign of the log fold change value: (+) for up-regulated genes and (-) for down-regulated genes. A gene is a target gene if it corresponds to a node in the network that has at least one incoming edge. We define a *consistent gene* as a target DE gene such that the sign of the gene is consistent both with the type of the signal **and** with the hypothesis considered. Formally, by definition, a target DE gene *g* is consistent with Hypothesis HP if and only if an incoming edge *e* exists such that *sign(g) = sign(e)*. In other words, this describes the situation when the CDT is predicted as present, the signal is activation and the target DE gene is up-regulated, or the signal is inhibition and the target DE gene is down-regulated (see panel A in **Figure 6.1.1**). A target DE gene *g* is consistent with Hypothesis HA if and only if an incoming edge *e* exists such that *sign(g) ≠ sign(e)*. This second case captures the situation in which the CDT is absent (or insufficient), the signal is inhibition and the target DE gene is up-regulated, or the signal is activation and the target DE gene is down-regulated (see panel B in **Figure 6.1.1**).

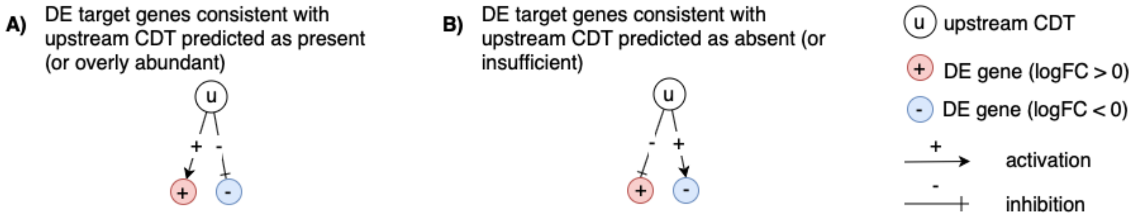

Fig. 9.1.1: Target genes consistent with the hypothesis considered: *In panel A, the signs of the DE genes match the signs of their respective incoming edges, increasing the likelihood that the CDT u is present. In panel B, the signs of the DE genes are opposite to the signs of their edges, increasing the likelihood that the CDT u is absent.*

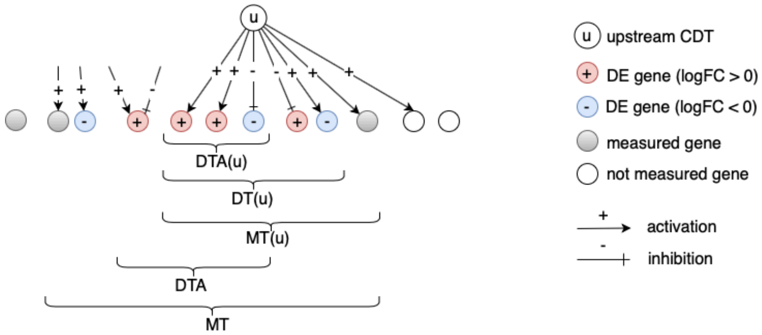

Fig. 9.1.2: *The set of all genes includes the set of measured genes that are also targets in the network, or Measured Targets (MT). We define the subset of "DE Targets consistent with the first hypothesis that the CDTs are Present (or overly abundant)", DTA. For a selected upstream CDT u, we have the set of "Measured Targets of u" MT(u), "Differentially expressed Targets downstream of u" DT(u), and the set of "DE targets consistent with the hypothesis HP that u is Present" DTA(u). The equivalent graphic for the hypothesis HA associated with DTI and DTI(u) is not shown.*

Z-score

For both research hypotheses, the analysis computes a Z-score for each CDT *z(u)* by iterating over the genes in DT(u) and their incoming edges *in(g)*. We can then compute the p-value corresponding to the z-score P_z as the one-tailed area under the probability density function for a normal distribution, N(0,1).

Upstream CDTs predicted as present (or overly abundant)

Here, the research hypothesis considers presence of the CDT. This hypothesis is useful when investigating whether the given phenotype has been impacted by the presence of a given chemical, drug or toxicant (e.g. tobacco smoke, dioxin, etc.). For each CDT *u*, the number of consistent DE genes downstream of *u*, DTA(u) is compared to the number of measured target genes expected to be both consistent and DE just by chance. iPathwayGuide uses an over-representation approach to compute the statistical significance of observing at least the given number of consistent DE genes. The p-value P_{pres} is computed using the hypergeometric distribution (Draghici *et al*., 2003, Draghici 2011).

After computing a p-value for both types of evidence, P_z and P_{pres}, we combine these two probabilities into one global probability value, P_G that is used to rank the upstream regulators and test the research hypothesis that the upstream CDTs are predicted as present in the condition studied. The analysis uses the standard Fisher's method to combine p-values into one test statistic (Fisher 1925).

Upstream CDTs predicted as absent (or insufficient)

In parallel with upstream CDTs predicted as present, we use P_{abs} and P_z to predict upstream CDTs that are absent. This hypothesis is relevant when investigating whether the given phenotype has been impacted by the lack of a given chemical that is necessary for the well-functioning of the organism or cell (e.g. a vitamin deficiency, iron deficiency, etc.). Here, the research hypothesis states that the upstream CDT are insufficient in the condition studied. For each upstream CDT *u*, the number of consistent DE genes downstream of *u*, DTI(u) is compared to the number of measured target genes expected to be both consistent and DE just by chance. Using the Fisher's method as above, the analysis combines P_{abs} and P_z, where P_z is considered only for significant negative z-scores ($z \leq -2$).

9.2. Results: upstream CDTs predicted as present (or overly abundant)

CDT (u)	DTA(u)	DT(u)	p-value	p-value (FDR)	p-value (Bonferroni)
Smoke	36	47	0.004	0.963	1.000
Phenformin	8	11	0.005	0.963	1.000
3-((6-(2-methoxyphenyl)pyrimidin-4-yl)amino)phenyl)methane sulfonamide	7	7	0.006	0.963	1.000
SEA 0400	1	1	0.009	0.963	1.000
bis(quercetinato)oxovanadium(IV)	1	1	0.009	0.963	1.000

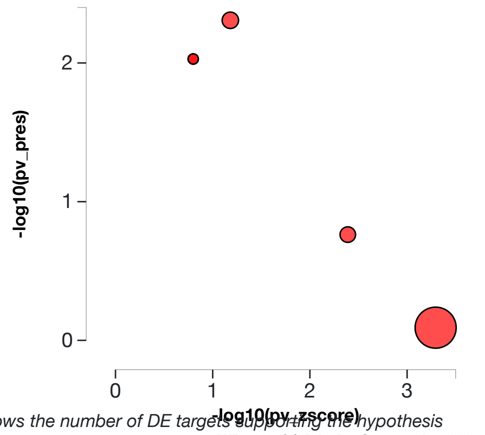

Table 9.2.1: Top upstream CDTs predicted as present (or overly abundant). *For each upstream CDT u, the table shows the number of DE targets supporting the hypothesis that the CDT is present DTA(u) the total number of DE genes downstream of u DT(u), the combined raw p-value, and the p-value corrected for multiple comparisons.* **Fig. 9.2.1: A two-way plot showing the top five upstream CDTs predicted as present (or overly abundant).** *Dots representing upstream CDTs are positioned using P_{zscore} on the horizontal axis, and using P_{pres} on the vertical axis. P_{pres} is the p-value based on the number of DE targets consistent with the typeof the incoming signal and with the selected hypothesis type. Upstream CDTs with a significant combined p-value are shown in red. The size of each dot represents the relative number of consistent DE genes for that CDT.*

Smoke

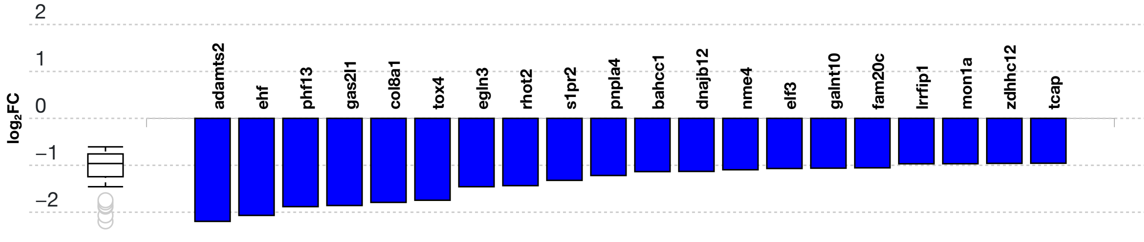

Fig. 9.2.3: Consistent DE target genes measured expression bar plot: *All the consistent differentially expressed genes that are targeted by Smoke are ranked based on their absolute value of log fold change. The plot is limited to the top 20 genes out of a total of 36 consistent differentially expressed target genes. Upregulated genes are shown in red, downregulated genes are shown in blue. The box and whisker plot on the left summarizes the distribution of all the consistent differentially expressed genes targeted by this upstream regulator. The box shows the 1st quartile, the median and the 3rd quartile, while any outliers are represented by circles.*

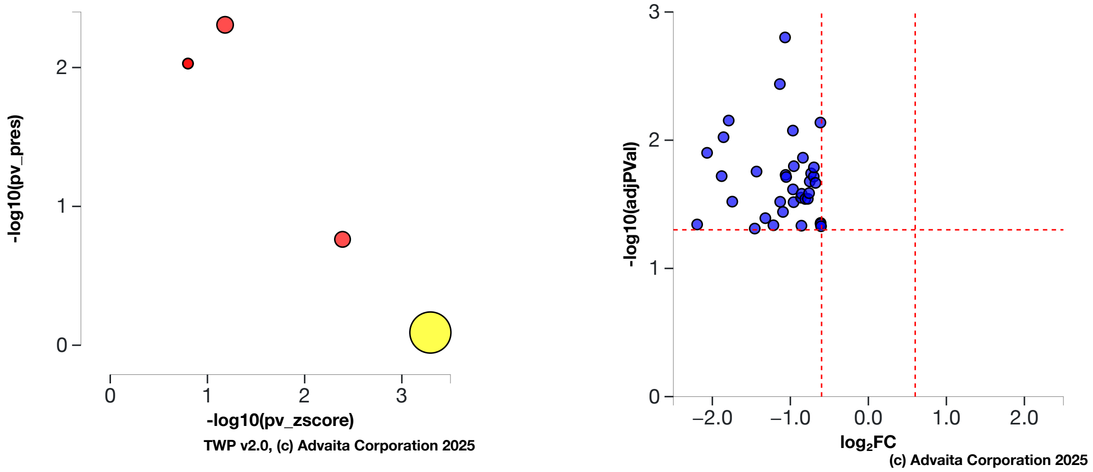

a) b)

Fig. 9.2.4: a) Present (overly abundant) p-value vs zscore p-value: *The significance of Smoke is plotted on two axes, with negative log of P_z on x-axis and negative log of P_{pres} on y-axis. The size of the dot represents the relative number of consistent DE genes, which for selected upstream regulator is 36.* **b) Volcano plot:** *There are* ***36*** *DE genes that are targets of Smoke consistent with the hypothesis that Smoke is present (overly abundant) The target genes are represented in terms of their measured expression change (x-axis) and the significance of the change (y-axis). The significance is represented in terms of the negative log (base 10) of the p-value, so that more significant genes are plotted higher on the y-axis. The dotted lines represent the thresholds used to select the DE genes:* ***0.6*** *for expression change and* ***0.05*** *for significance.*

Phenformin

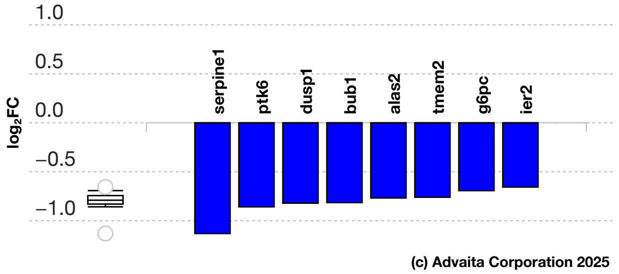

Fig. 9.2.5: Consistent DE target genes measured expression bar plot: *All the consistent differentially expressed genes that are targeted by Phenformin are ranked based on their absolute value of log fold change. The box and whisker plot on the left summarizes the distribution of all the consistent differentially expressed genes targeted by this upstream regulator. The box shows the 1st quartile, the median and the 3rd quartile, while any outliers are represented by circles.*

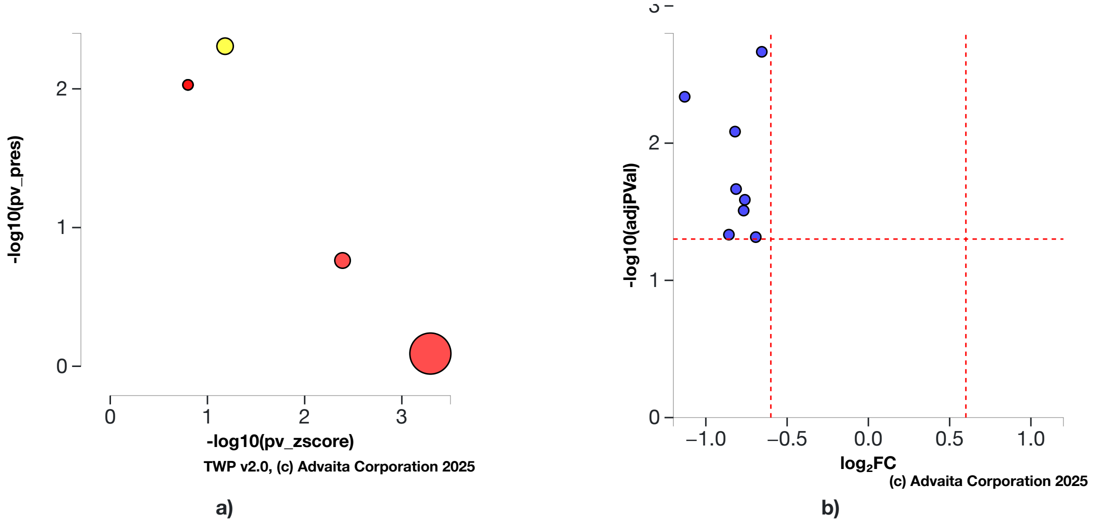

Fig. 9.2.6: a) Present (overly abundant) p-value vs zscore p-value: *The significance of Phenformin is plotted on two axes, with negative log of P_z on x-axis and negative log of P_{pres} on y-axis. The size of the dot represents the relative number of consistent DE genes, which for selected upstream regulator is 8.* **b) Volcano plot:** *There are **8** DE genes that are targets of Phenformin consistent with the hypothesis that Phenformin is present (overly abundant) The target genes are represented in terms of their measured expression change (x-axis) and the significance of the change (y-axis). The significance is represented in terms of the negative log (base 10) of the p-value, so that more significant genes are plotted higher on the y-axis. The dotted lines represent the thresholds used to select the DE genes: **0.6** for expression change and **0.05** for significance.*

3-((6-(2-methoxyphenyl)pyrimidin-4-yl)amino)phenyl)methane sulfonamide

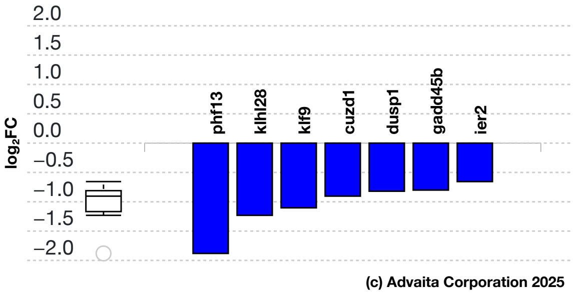

Fig. 9.2.7: Consistent DE target genes measured expression bar plot: *All the consistent differentially expressed genes that are targeted by 3-((6-(2-methoxyphenyl)pyrimidin-4-yl)amino)phenyl)methane sulfonamide are ranked based on their absolute value of log fold change. The box and whisker plot on the left summarizes the distribution of all the consistent differentially expressed genes targeted by this upstream regulator. The box shows the 1st quartile, the median and the 3rd quartile, while any outliers are represented by circles.*

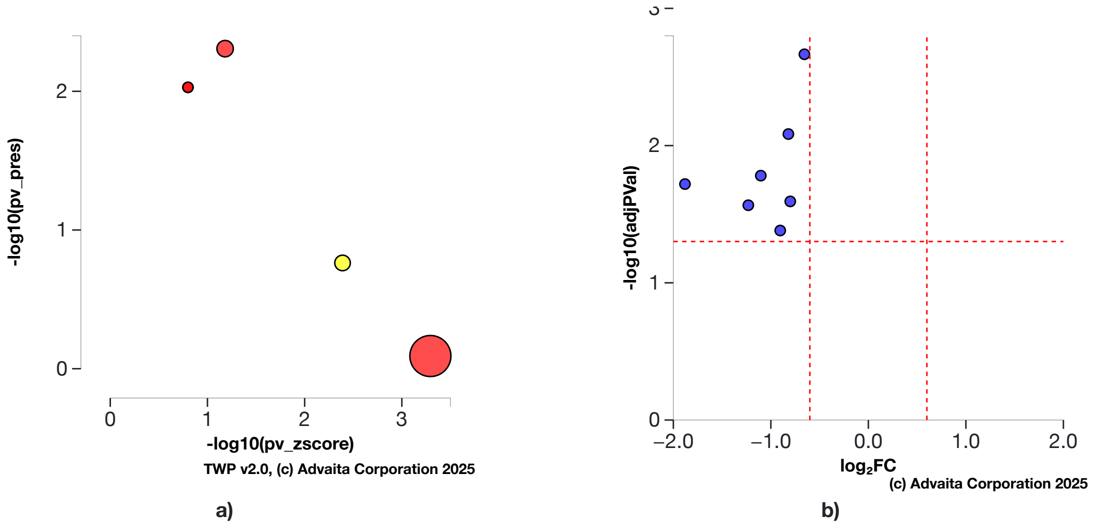

Fig. 9.2.8: a) Present (overly abundant) p-value vs zscore p-value: *The significance of 3-((6-(2-methoxyphenyl)pyrimidin-4-yl)amino)phenyl)methane sulfonamide is plotted on two axes, with negative log of P_z on x-axis and negative log of P_{pres} on y-axis. The size of the dot represents the relative number of consistent DE genes, which for selected upstream regulator is 7.* **b) Volcano plot:** *There are **7** DE genes that are targets of 3-((6-(2-methoxyphenyl)pyrimidin-4-yl)amino)phenyl)methane sulfonamide consistent with the hypothesis that 3-((6-(2-methoxyphenyl)pyrimidin-4-yl)amino)phenyl)methane sulfonamide is present (overly abundant) The target genes are represented in terms of their measured expression change (x-axis) and the significance of the change (y-axis). The significance is represented in terms of the negative log (base 10) of the p-value, so that more significant genes are plotted higher on the y-axis. The dotted lines represent the thresholds used to select the DE genes:* ***0.6*** *for expression change and* ***0.05*** *for significance.*

SEA 0400

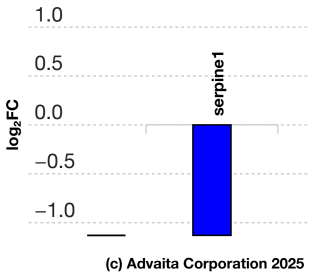

Fig. 9.2.9: Consistent DE target genes measured expression bar plot: *All the consistent differentially expressed genes that are targeted by SEA 0400 are ranked based on their absolute value of log fold change. The box and whisker plot on the left summarizes the distribution of all the consistent differentially expressed genes targeted by this upstream regulator. The box shows the 1st quartile, the median and the 3rd quartile, while any outliers are represented by circles.*

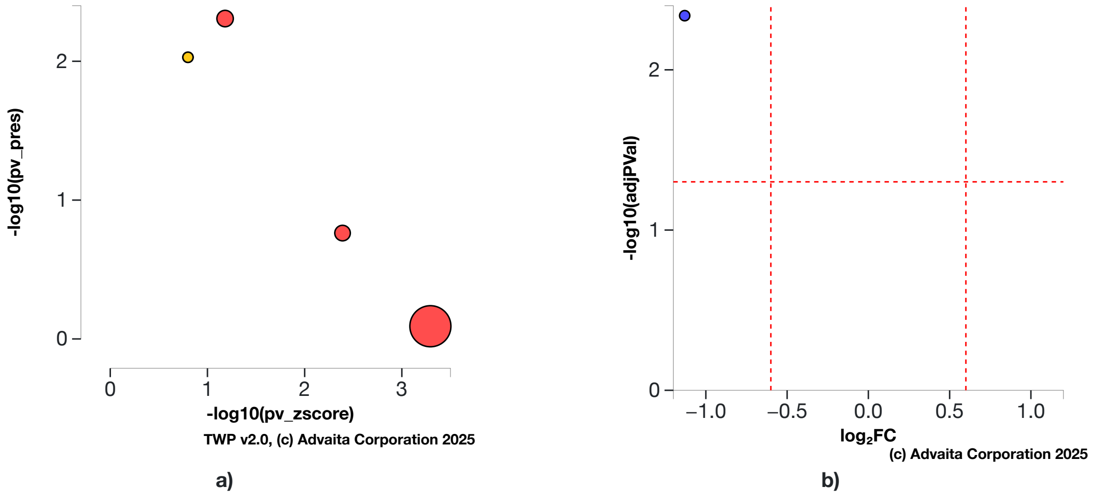

Fig. 9.2.10: a) Present (overly abundant) p-value vs zscore p-value: *The significance of SEA 0400 is plotted on two axes, with negative log of P_z on x-axis and negative log of P_{pres} on y-axis. The size of the dot represents the relative number of consistent DE genes, which for selected upstream regulator is 1.* **b) Volcano plot:** *There are **1** DE genes that are targets of SEA 0400 consistent with the hypothesis that SEA 0400 is present (overly abundant) The target genes are represented in terms of their measured expression change (x-axis) and the significance of the change (y-axis). The significance is represented in terms of the negative log (base 10) of the p-value, so that more significant genes are plotted higher on the y-axis. The dotted lines represent the thresholds used to select the DE genes:* ***0.6*** *for expression change and* ***0.05*** *for significance.*

bis(quercetinato)oxovanadium(IV)

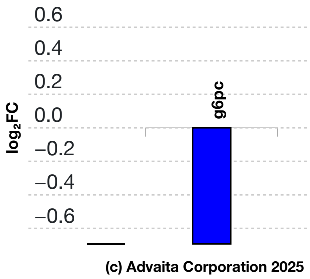

Fig. 9.2.11: Consistent DE target genes measured expression bar plot: *All the consistent differentially expressed genes that are targeted by bis(quercetinato)oxovanadium(IV) are ranked based on their absolute value of log fold change. The box and whisker plot on the left summarizes the distribution of all the consistent differentially expressed genes targeted by this upstream regulator. The box shows the 1st quartile, the median and the 3rd quartile, while any outliers are represented by circles.*

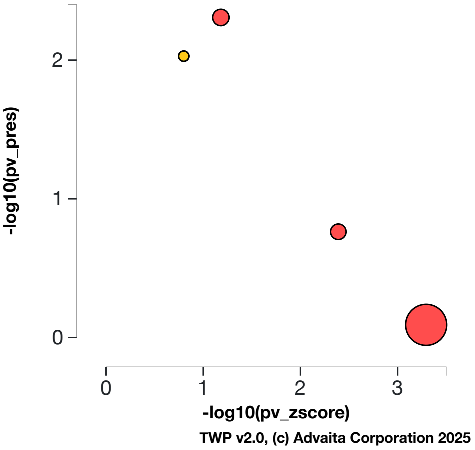

a)

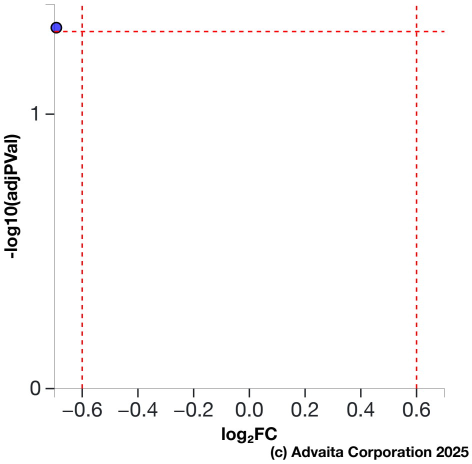

b)

Fig. 9.2.12: a) Present (overly abundant) p-value vs zscore p-value: *The significance of bis(quercetinato)oxovanadium(IV) is plotted on two axes, with negative log of P_z on x-axis and negative log of P_{pres} on y-axis. The size of the dot represents the relative number of consistent DE genes, which for selected upstream regulator is 1.* **b) Volcano plot:** *There are **1** DE genes that are targets of bis(quercetinato)oxovanadium(IV) consistent with the hypothesis that bis(quercetinato)oxovanadium(IV) is present (overly abundant) The target genes are represented in terms of their measured expression change (x-axis) and the significance of the change (y-axis). The significance is represented in terms of the negative log (base 10) of the p-value, so that more significant genes are plotted higher on the y-axis. The dotted lines represent the thresholds used to select the DE genes: **0.6** for expression change and **0.05** for significance.*

9.3. Results: upstream CDTs predicted as absent (or insufficient)

CDT (u)	DTI(u)	DT(u)	p-value	p-value (FDR)	p-value (Bonferroni)
perfluoroheptanesulfonic acid	3	3	7.871e-6	0.009	0.009
Nevirapine	5	5	4.267e-4	0.249	0.501
entinostat	18	20	6.353e-4	0.249	0.746
afimoxifene	6	6	0.001	0.354	1.000
Indomethacin	14	15	0.002	0.428	1.000

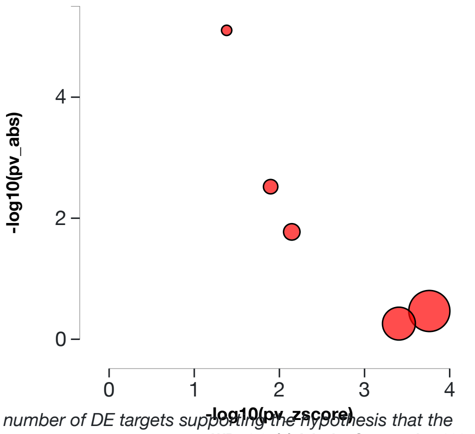

Table 9.3.1: Top upstream CDTs predicted as absent (or insufficient). *For each upstream CDT u, the table shows the number of DE targets supporting the hypothesis that the CDT is absent DTI(u) the total number of DE genes downstream of u DT(u), the combined raw p-value, and the p-value corrected for multiple comparisons.*

Fig. 9.3.1: A two-way plot showing the top five upstream CDTs predicted as absent (or insufficient). *Dots representing upstream CDTs are positioned using P_{zscore} on the horizontal axis, and using P_{abs} on the vertical axis. P_{abs} is the p-value based on the number of DE targets consistent with the typeof the incoming signal and with the selected hypothesis type. Upstream CDTs with a significant combined p-value are shown in red. The size of each dot represents the relative number of consistent DE genes for that CDT.*

perfluoroheptanesulfonic acid

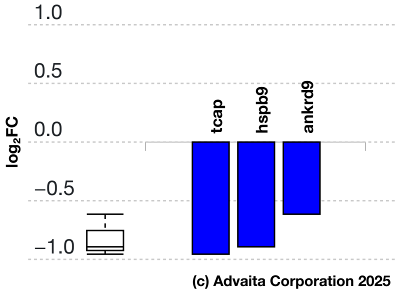

Fig. 9.3.13: Consistent DE target genes measured expression bar plot: *All the consistent differentially expressed genes that are targeted by perfluoroheptanesulfonic acid are ranked based on their absolute value of log fold change. The box and whisker plot on the left summarizes the distribution of all the consistent differentially expressed genes targeted by this upstream regulator. The box shows the 1st quartile, the median and the 3rd quartile, while any outliers are represented by circles.*

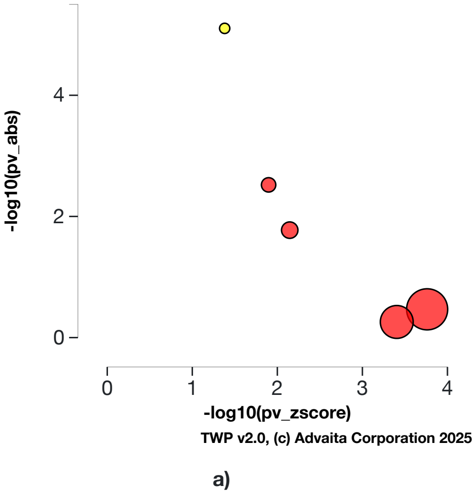

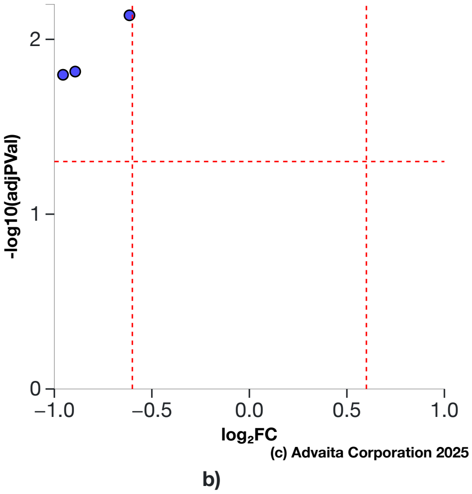

Fig. 9.3.14: a) Absent (or insufficient) p-value vs zscore p-value: *The significance of perfluoroheptanesulfonic acid is plotted on two axes, with negative log of P_z on x-axis and negative log of P_{abs} on y-axis. The size of the dot represents the relative number of consistent DE genes, which for selected upstream regulator is 3.* **b) Volcano plot:** *There are **3** DE genes that are targets of perfluoroheptanesulfonic acid consistent with the hypothesis that perfluoroheptanesulfonic acid is absent (or insufficient) The target genes are represented in terms of their measured expression change (x-axis) and the significance of the change (y-axis). The significance is represented in terms of the negative log (base 10) of the p-value, so that more significant genes are plotted higher on the y-axis. The dotted lines represent the thresholds used to select the DE genes: **0.6** for expression change and **0.05** for significance.*

Nevirapine

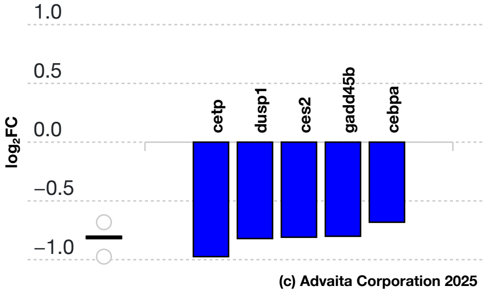

Fig. 9.3.15: Consistent DE target genes measured expression bar plot: *All the consistent differentially expressed genes that are targeted by Nevirapine are ranked based on their absolute value of log fold change. The box and whisker plot on the left summarizes the distribution of all the consistent differentially expressed genes targeted by this upstream regulator. The box shows the 1st quartile, the median and the 3rd quartile, while any outliers are represented by circles.*

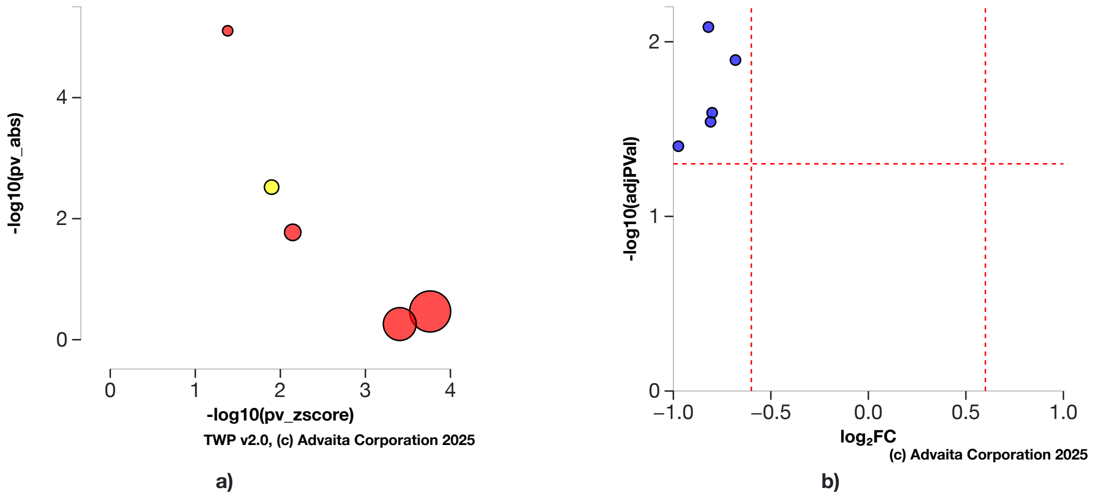

Fig. 9.3.16: a) Absent (or insufficient) p-value vs zscore p-value: *The significance of Nevirapine is plotted on two axes, with negative log of P_z on x-axis and negative log of P_{abs} on y-axis. The size of the dot represents the relative number of consistent DE genes, which for selected upstream regulator is 5.* **b) Volcano plot:** *There are* ***5*** *DE genes that are targets of Nevirapine consistent with the hypothesis that Nevirapine is absent (or insufficient) The target genes are represented in terms of their measured expression change (x-axis) and the significance of the change (y-axis). The significance is represented in terms of the negative log (base 10) of the p-value, so that more significant genes are plotted higher on the y-axis. The dotted lines represent the thresholds used to select the DE genes:* ***0.6*** *for expression change and* ***0.05*** *for significance.*

entinostat

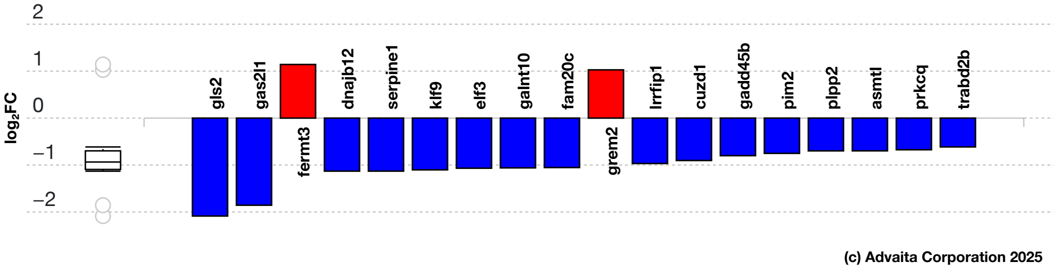

Fig. 9.3.17: Consistent DE target genes measured expression bar plot: *All the consistent differentially expressed genes that are targeted by entinostat are ranked based on their absolute value of log fold change. The box and whisker plot on the left summarizes the distribution of all the consistent differentially expressed genes targeted by this upstream regulator. The box shows the 1st quartile, the median and the 3rd quartile, while any outliers are represented by circles.*

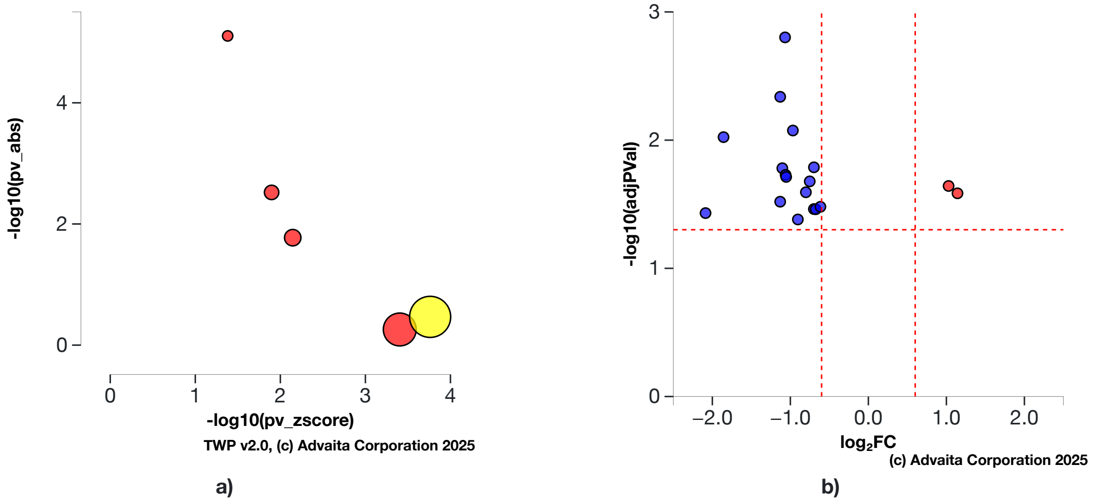

Fig. 9.3.18: a) Absent (or insufficient) p-value vs zscore p-value: *The significance of entinostat is plotted on two axes, with negative log of P_z on x-axis and negative log of P_{abs} on y-axis. The size of the dot represents the relative number of consistent DE genes, which for selected upstream regulator is 18.* **b) Volcano plot:** *There are* ***18*** *DE genes that are targets of entinostat consistent with the hypothesis that entinostat is absent (or insufficient) The target genes are represented in terms of their measured expression change (x-axis) and the significance of the change (y-axis). The significance is represented in terms of the negative log (base 10) of the p-value, so that more significant genes are plotted higher on the y-axis. The dotted lines represent the thresholds used to select the DE genes:* ***0.6*** *for expression change and* ***0.05*** *for significance.*

afimoxifene

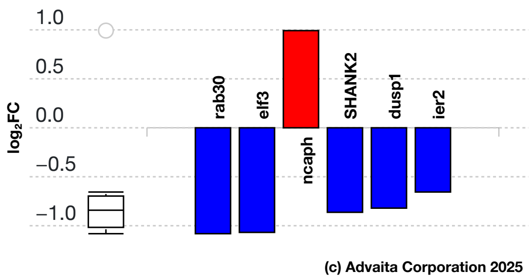

Fig. 9.3.19: Consistent DE target genes measured expression bar plot: *All the consistent differentially expressed genes that are targeted by afimoxifene are ranked based on their absolute value of log fold change. The box and whisker plot on the left summarizes the distribution of all the consistent differentially expressed genes targeted by this upstream regulator. The box shows the 1st quartile, the median and the 3rd quartile, while any outliers are represented by circles.*

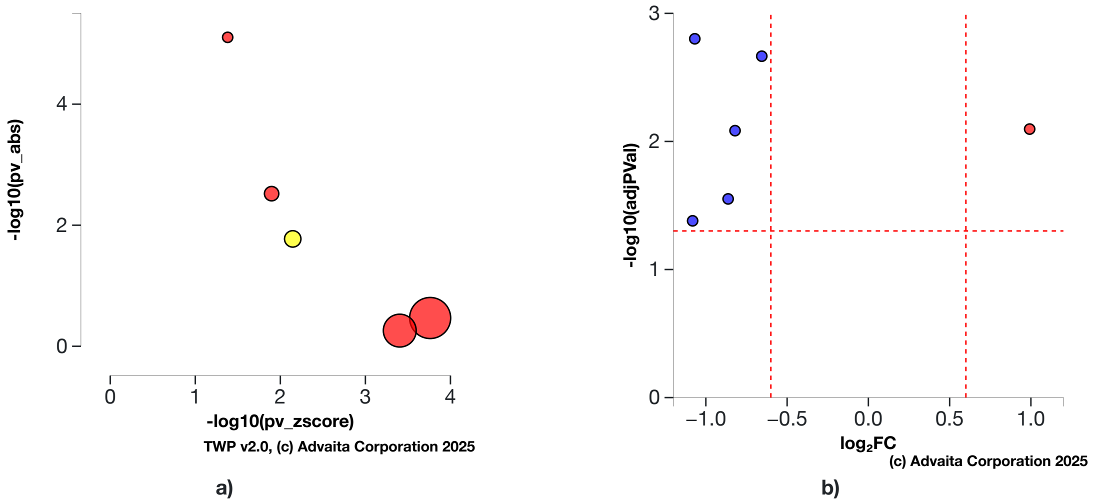

Fig. 9.3.20: a) Absent (or insufficient) p-value vs zscore p-value: *The significance of afimoxifene is plotted on two axes, with negative log of P_z on x-axis and negative log of P_{abs} on y-axis. The size of the dot represents the relative number of consistent DE genes, which for selected upstream regulator is 6.* **b) Volcano plot:** *There are* ***6*** *DE genes that are targets of afimoxifene consistent with the hypothesis that afimoxifene is absent (or insufficient) The target genes are represented in terms of their measured expression change (x-axis) and the significance of the change (y-axis). The significance is represented in terms of the negative log (base 10) of the p-value, so that more significant genes are plotted higher on the y-axis. The dotted lines represent the thresholds used to select the DE genes:* ***0.6*** *for expression change and* ***0.05*** *for significance.*

Indomethacin

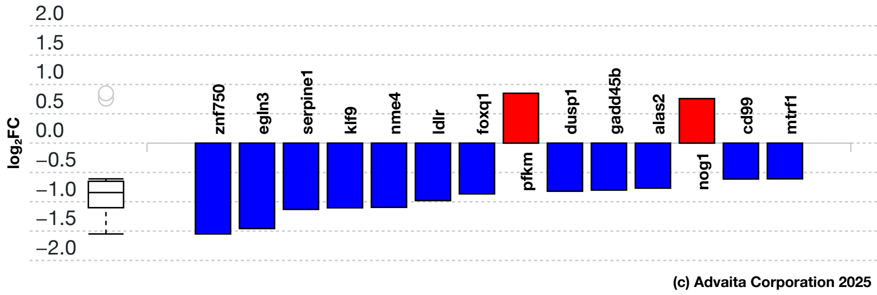

Fig. 9.3.21: Consistent DE target genes measured expression bar plot: *All the consistent differentially expressed genes that are targeted by Indomethacin are ranked based on their absolute value of log fold change. The box and whisker plot on the left summarizes the distribution of all the consistent differentially expressed genes targeted by this upstream regulator. The box shows the 1st quartile, the median and the 3rd quartile, while any outliers are represented by circles.*

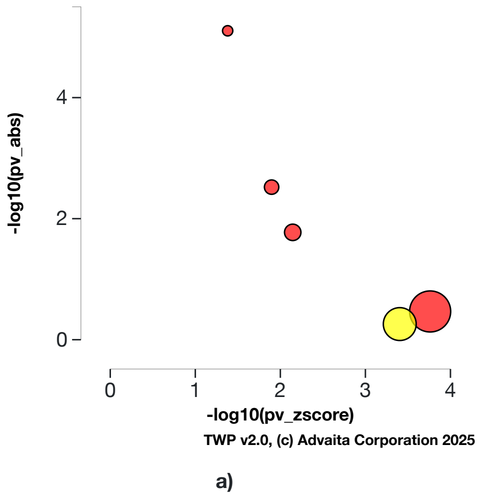

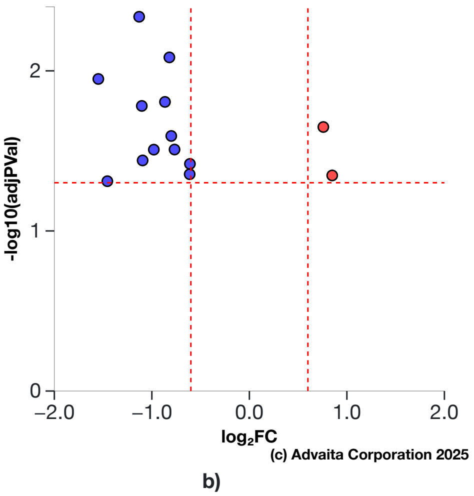

Fig. 9.3.22: a) Absent (or insufficient) p-value vs zscore p-value: *The significance of Indomethacin is plotted on two axes, with negative log of P_z on x-axis and negative log of P_{abs} on y-axis. The size of the dot represents the relative number of consistent DE genes, which for selected upstream regulator is 14.* **b) Volcano plot:** *There are **14** DE genes that are targets of Indomethacin consistent with the hypothesis that Indomethacin is absent (or insufficient) The target genes are represented in terms of their measured expression change (x-axis) and the significance of the change (y-axis). The significance is represented in terms of the negative log (base 10) of the p-value, so that more significant genes are plotted higher on the y-axis. The dotted lines represent the thresholds used to select the DE genes: **0.6** for expression change and **0.05** for significance.*

10. Disease Analysis

10.1. Methods

For each disease, the number of differentially expressed (DE) genes annotated to a disease term is compared to the number of DE genes expected just by chance. iPathwayGuide uses an over-representation approach to compute the statistical significance of observing at least the given number of DE genes. The p-value is computed using the hypergeometric distribution as described for pORA in the Pathway Analysis section. This p-value is corrected for multiple comparisons using FDR and Bonferroni.

10.2. Results

Table 10.2.1: Top identified diseases

Disease Name	p-value	p-value (FDR)	p-value (Bonferroni)
Hyperlipoproteinemia type IIa; LDL receptor disorder	0.005	0.062	0.925
Atherosclerosis	0.007	0.062	1.000
Hyperlipidemia	0.008	0.062	1.000
Seborrhea-like dermatitis with psoriasiform element	0.009	0.062	1.000
Raine syndrome; Lethal osteosclerotic bone dysplasia	0.009	0.062	1.000

Hyperlipoproteinemia type IIa; LDL receptor disorder (H01383)

Familial hypercholesterolemia is characterized by severely elevated low-density lipoprotein (LDL) cholesterol, xanthomas, and the development of premature cardiovascular disease. Hyperlipoproteinemia type IIa is an autosomal dominant disorder caused by mutations in the LDL receptor. The LDL receptor gene consists of a number of distinct functional domains such as signal sequence, ligand binding, and so on. There are more than 1600 mutations in the LDLR gene that can cause familial hypercholesterolemia, accounting for up to 95% of all cases. In this experiment, the algorithm identified **2** differentially expressed genes out of **12** genes associated with the disease.

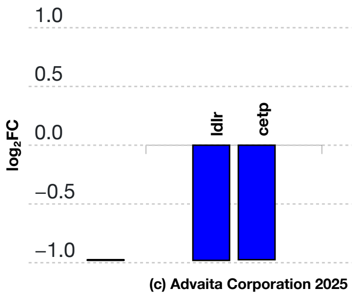

Fig. 10.2.1: Gene measured expression bar plot: *All the differentially expressed genes that are annotated to Hyperlipoproteinemia type IIa; LDL receptor disorder are ranked based on their absolute value of log fold change. Upregulated genes are shown in red, downregulated genes are shown in blue. The box plot on the left summarizes the distribution of all the differentially expressed genes that are annotated to this disease. The box represents the 1st quartile, the median and the 3rd quartile, while the outliers are represented by circles.*

Atherosclerosis (H02505)

Atherosclerosis is a chronic inflammatory disease marked by a narrowing of the arteries from lipid-rich plaques present within the walls of arterial blood vessels. It represents the root cause of the majority of cardiovascular diseases (CVDs) and their complications, including conditions such as coronary artery disease, myocardial infarction and stroke. Atherosclerosis develops as a result of the interactions of various genetic and environmental factors. Elevated cholesterol and LDLcholesterol (LDL-C) levels are the main risk factors that associated with the formation of atherosclerotic plaques and the development of atherosclerosis. In this experiment, the algorithm identified **3** differentially expressed genes out of **41** genes associated with the disease.

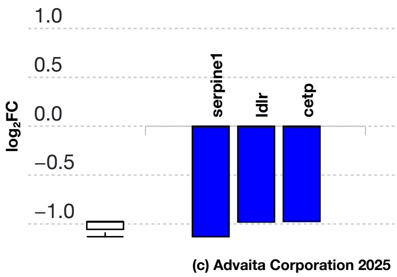

Fig. 10.2.2: Gene measured expression bar plot: *All the differentially expressed genes that are annotated to Atherosclerosis are ranked based on their absolute value of log fold change. Upregulated genes are shown in red, downregulated genes are shown in blue. The box plot on the left summarizes the distribution of all the differentially expressed genes that are annotated to this disease. The box represents the 1st quartile, the median and the 3rd quartile, while the outliers are represented by circles.*

Hyperlipidemia (H01635)

Dyslipidemia is a condition characterized by either an increase or decrease in concentration of lipids in the blood. Hyperlipidemia, which refers to an increase in cholesterol, triglyceride (TG), or both, is the most common form of dyslipidemia. Hyperlipidemias can be classified as familial (also called primary) caused by an inherited gene mutation, or acquired (also called secondary) when resulting from underlying disorders that lead to alterations in plasma lipid and lipoprotein metabolism. The causes of acquired hyperlipidemia include dietary, alcohol intake, oral contraceptives, diabetes mellitus, and pharmacological agents (e.g., retinoic acid derivatives, steroids, and beta-blockers). Familial hyperlipidemias are classified according to the Fredrickson classification (hyperlipoproteinemia types I to V) which is based on lipoprotein analyses by electrophoresis or ultracentrifugation. It was later adopted by the World Health Organization (WHO). Hyperlipidemias are also classified according to which types of lipids are elevated. Hypercholesterolemia, hypertriglyceridemia, and combined hyperlipidemia refer to elevations involving the major cholesterol-rich lipoproteins (LDL), triglyceride-rich lipoproteins (VLDL), and both, respectively. In this experiment, the algorithm identified **2** differentially expressed genes out of **15** genes associated with the disease.

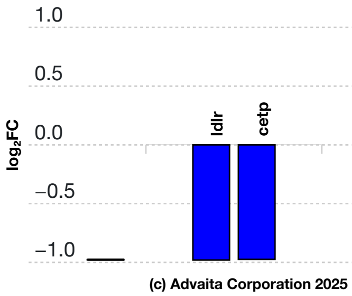

Fig. 10.2.3: Gene measured expression bar plot: *All the differentially expressed genes that are annotated to Hyperlipidemia are ranked based on their absolute value of log fold change. Upregulated genes are shown in red, downregulated genes are shown in blue. The box plot on the left summarizes the distribution of all the differentially expressed genes that are annotated to this disease. The box represents the 1st quartile, the median and the 3rd quartile, while the outliers are represented by circles.*

Seborrhea-like dermatitis with psoriasiform element (H00795)

Seborrhea-like dermatosis with psoriasiform elements is a chronic dermatosis characterized by common dandruffs and eczematous or psoriasiform plaques. Enhanced keratinocyte proliferation and dermal infiltration of inflammatory cells are observed in the disease. Mutations in ZNF750, which encodes a putative C2H2 zinc finger protein, have been reported. In this experiment, the algorithm identified **1** differentially expressed genes out of **1** genes associated with the disease.

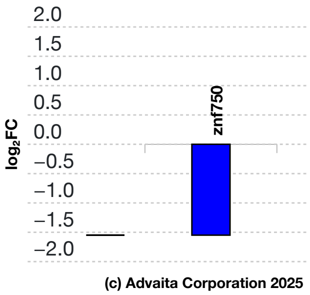

Fig. 10.2.4: Gene measured expression bar plot: *All the differentially expressed genes that are annotated to Seborrhea-like dermatitis with psoriasiform element are ranked based on their absolute value of log fold change. Upregulated genes are shown in red, downregulated genes are shown in blue. The box plot on the left summarizes the distribution of all the differentially expressed genes that are annotated to this disease. The box represents the 1st quartile, the median and the 3rd quartile, while the outliers are represented by circles.*

Raine syndrome; Lethal osteosclerotic bone dysplasia (H00968)

Raine syndrome or lethal osteosclerotic bone dysplasia is an autosomal recessive disorder characterized by generalized osteosclerosis and characteristic facial dysmorphism. Death in the neonatal period is due to respiratory failure. Radiographic studies have shown generalized increase in the density of all bones and the basal structures of the skull, which leads to the characteristic facial features including microcephaly, proptosis, hypoplastic nose and midface, small jaw, and cleft palate. In this experiment, the algorithm identified **1** differentially expressed genes out of **1** genes associated with the disease.

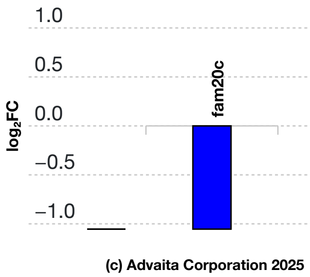

Fig. 10.2.5: Gene measured expression bar plot: *All the differentially expressed genes that are annotated to Raine syndrome; Lethal osteosclerotic bone dysplasia are ranked based on their absolute value of log fold change. Upregulated genes are shown in red, downregulated genes are shown in blue. The box plot on the left summarizes the distribution of all the differentially expressed genes that are annotated to this disease. The box represents the 1st quartile, the median and the 3rd quartile, while the outliers are represented by circles.*

11. References

- Agarwal V, Bell GW, Nam J, Bartel DP. Predicting effective microRNA target sites in mammalian mRNAs. eLife, 4:e05005 (2015).
- Alexa, A., Rahnenfuehrer, J., Lengauer,T.: Improved scoring of functional groups from gene expression data by decorrelating GO graph structure. Bioinformatics 22(13): 1600-1607 (2006).
- Ashburner, M., Ball, C.A., Blake, J.A., Botstein, D., Butler, H., Cherry, J.M., Davis, A.P., Dolinski, K., Dwight, S.S., Eppig, J.T., Harris, M.A., Hill, D.P., Issel-Tarver, L., Kasarskis, A., Lewis, S., Matese, J.C., Richardson, J.E., Ringwald, M., Rubin, G.M., Sherlock, G.: The Gene Ontology Consortium. Gene ontology: Tool for the unification of biology. Nature Genetics 25(1): 25-9 (2000).
- Ashburner, M., Lewis, S.: On Ontologies for Biologists: The Gene Ontology - Untangling the web: 'In Silico' simulation of biological processes: Novartis Found Symp, 247:66-80; discussion 80-3, 84-90: 244-52 (2002).
- Benjamini, Y. and Hochberg, Y.: Controlling the false discovery rate: A practical and powerful approach to multiple testing. Journal of the Royal Statistical Society B, 57(1):289-300, (1995).
- Benjamini, Y. and Yekutieli, D.: The control of the false discovery rate in multiple testing under dependency. Annals of Statistics, 29(4):1165-1188, (2001).
- Bonferroni, C. E.: Il calcolo delle assicurazioni su gruppi di teste, chapter "Studi in Onore del Professore Salvatore Ortu Carboni", pages 13-60, Rome, (1935).
- Bonferroni, C. E.: Teoria statistica delle classi e calcolo delle probabilita. Pubblicazioni del Istituto Superiore di Scienze Economiche e Commerciali di Firenze, 8:3-62, (1936).
- Camon, E., Magrane, M., Barrell, D., Lee, V., Dimmer, E., Maslen, J., Binns, D., Harte, N., Lopez, R., Apweiler, R.: The Gene Ontology Annotation (GOA) database: sharing knowledge in Uniprot with Gene Ontology. Nucleic Acids Research, 32(Database issue), D262-D266 (2004).
- Davis AP, Grondin CJ, Johnson RJ, Sciaky D, McMorran R, Wiegers J, Wiegers TC, Mattingly CJ, The Comparative Toxicogenomics Database: update 2019, Nucleic Acids Research, 47(D1): D948-D954 (2019).
- Draghici, S., Khatri, P., Martins, R.P., Ostermeier, G.C. and Krawetz, S.A.: Global functional profiling of gene expression. Genomics, 81(2), pp.98-104 (2003).
- Draghici, S., Khatri, P., Bhavsar, P., Shah, A., Krawetz, S., Tainsky, M.A.: Onto-Tools, The toolkit of the modern biologist: Onto-Express, Onto-Compare, Onto-Design and Onto-Translate. Nucleic Acids Research, 31(13): 3775-81 (2003).
- Draghici, S., Khatri, P., Tarca, A.L., Amin, K., Done, A., Voichita, C., Georgescu, C., Romero, R.: A systems biology approach for pathway level analysis. Genome Research, 17(10): 1537-45 (2007).
- Draghici, S.: Statistics and Data Analysis for Microarrays Using R and Bioconductor, second edition. Chapman and Hall/CRC (2011).
- Friedman, R.C., Farh, K.K., Burge, C.B., Bartel, D.P.: Most mammalian mRNAs are conserved targets of microRNAs. Genome Research, 19: 92-105 (2009).

- Garcia, D.M., Baek, D., Shin, C., Bell, G.W., Grimson, A., Bartel, D.P.: Weak seed-pairing stability and high target-site abundance decrease the proficiency of lsy-6 and other miRNAs. Nature Structural & Molecular Biology, 18: 1139-1146 (2011).
- Gene Ontology Consortium. Creating the Gene Ontology Resource: Design and Implementation. Genome Research 11: 1425-1433 (2001).
- Gene Ontology Consortium. The Gene Ontology (GO) database and informatics resource. Nucleic Acids Research 32 (suppl 1): D258-D261 (2004).
- Griffiths-Jones S.: The microRNA Registry. Nucleic Acids Research 32:D109-D111 (2004).
- Griffiths-Jones S., Grocock R.J., van Dongen S., Bateman A., Enright A.J.: miRBase: microRNA sequences, targets and gene nomenclature. Nucleic Acids Research 34:D140-D144 (2006).
- Griffiths-Jones S., Saini H.K., van Dongen S., Enright A.J.: miRBase: tools for microRNA genomics. Nucleic Acids Research 36:D154-D158 (2008).
- Grimson, A., Farh, K.K., Johnston, W.K., Garrett-Engele, P., Lim, L.P., Bartel, D.P.: MicroRNA targeting specificity in mammals: Determinants beyond seed pairing. Molecular Cell, 27: 91-105 (2007).
- Fisher R. A.: Statistical methods for research workers. Oliver & Boyd, Edinburgh, (1925).
- Kanehisa, M., Goto, S.: KEGG: Kyoto Encyclopedia of Genes and Genomes. Nucleic Acids Research 28: 27-30 (2000).
- Kanehisa, M., Goto, S., Kawashima, S., and Nakaya, A.: The KEGG databases at GenomeNet. Nucleic Acids Research 30: 42-46 (2002).
- Kanehisa, M., Goto, S., Kawashima, S., Okuno, Y., and Hattori, M.; The KEGG resources for deciphering the genome. Nucleic Acids Research 32: D277-D280 (2004).
- Kanehisa, M., Araki, M., Goto, S., Hattori, M., Hirakawa, M., Itoh, M., Katayama, T., Kawashima, S., Okuda, S., Tokimatsu, T., and Yamanishi, Y.: KEGG for linking genomes to life and the environment. Nucleic Acids Research 36: D480-D484 (2008).
- Kanehisa, M., Goto, S., Furumichi, M., Tanabe, M., Hirakawa, M.: KEGG for representation and analysis of molecular networks involving diseases and drugs. Nucleic Acids Research 38: D355-D360 (2010).
- Kanehisa, M., Goto, S., Sato, Y., Furumichi, M., Tanabe, M.: KEGG for integration and interpretation of large-scale molecular datasets. Nucleic Acids Research 40: D109-D114 (2012).
- Kanehisa, M., Goto, S., Sato, Y., Kawashima, M., Furumichi, M., and Tanabe, M.; Data, information, knowledge and principle: back to metabolism in KEGG. Nucleic Acids Research 42: D199-D205 (2014).
- Khatri, P., Draghici, S., Tarca, A.D., Hassan, S.S., Romero, R.: A system biology approach for the steady-state analysis of gene signaling networks. Lecture Notes in Computer Science (LNCS) 4756, pp 32-41 (2007).
- Kozomara A., Griffiths-Jones S.: miRBase: integrating microRNA annotation and deep-sequencing data. Nucleic Acids Research 39:D152-D157 (2011).
- Kozomara A., Griffiths-Jones S.: miRBase: annotating high confidence microRNAs using deep sequencing data. Nucleic Acids Research 42:D68-D73 (2014).
- Lewis, B.P., Burge, C.B., Bartel, D.P.: Conserved seed pairing, often flanked by adenosines, indicates that thousands of human genes are microRNA targets. Cell, 120(1):15-20 (2005).
- Nam J, Rissland OS, Koppstein D, Abreu-Goodger C, Jan CH, Agarwal V, Yildirim MA, Rodriguez A, Bartel DP. Global analyses of the effect of different cellular contexts on microRNA targeting.Molecular Cell, 53:1031-43 (2014).
- Rhee, S.Y., Wood, V., Dolinski, K., Draghici, S.: Use and misuse of the gene ontology annotations. Nature Reviews Genetics 9(4):509-515 (2008).
- Szklarczyk, D., Morris, J.H., Cook, H., *et al*. The STRING database in 2017: quality-controlled protein-protein association networks, made broadly accessible. Nucleic Acids Research 45(D1):D362-D368 (2017).
- Tarca, A.L., Draghici, S., Khatri, P., Hassan, S., Mittal, P., Kim, J.S., Kim, C.J., Kusanovic, J.P., Romero, R.: A novel Signaling Pathway Impact Analysis (SPIA). Bioinformatics 25(1), 75-82 (2009).
