# Supplementary material for: Transcriptome and Behavioral Assessment in Larval Zebrafish (Danio rerio) Following Exposure to Perfluorononanoic Acid (PFNA)
Source: Genes (Basel). 2026 May 7;17(5):558. doi: 10.3390/genes17050558 (PMC13206344; doi:10.3390/genes17050558)
Supplement: Supplementary file 1 [file genes-17-00558-s001.zip › PFNA 10 Summary Report.pdf]

iPathwayGuide

iPathwayGuide

| | |
|---|---|
| **Title:** | PFNA 10 |
| **Description:** | File: Differential_expression_analysis_table high.txt |
| **Organism:** | Homo sapiens (9606) |
| **Contrast** | PFNA 10 vs. Control - mRNA (RNA-seq) |
| **Creation time:** | 09-30-2025 09:29 AM |

# 1. Introduction

In this experiment, **336** differentially expressed (DE) genes were identified out of a total of **11,595** genes with measured expression. These were identified using thresholds defined by the user. In this experiment, the user chose a threshold of **0.05** for statistical significance (p-value) and a log fold change of expression with absolute value of at least **1.2**. These data were analyzed in the context of pathways obtained from the Kyoto Encyclopedia of Genes and Genomes (KEGG) database (Release 113.0+/01-01, Jan 25) (Kanehisa *et al*., 2000; Kanehisa *et al*., 2002), gene ontologies from the Gene Ontology Consortium database (2024-Sep20) (Ashburner *et al*., 2000; Gene Ontology Consortium, 2001), miRNAs from the miRBase (MIRBASE Version:Version22.1,10/18) and TARGETSCAN (Targetscan version: Mouse:8.0, Human:8.0) databases (Agarwal *et al*., 2015; Nam *et al*., 2014; Griffiths-Jones et al., 2008; Kozomara and Griffiths-Jones, 2014; Friedman *et al*., 2009; Grimson *et al*., 2007), network of regulatory relations from BioGRID: Biological General Repository for Interaction Datasets v4.4.233. April. 25th, 2024 (Szklarczyk *et al*., 2017), chemicals/drugs/toxicants from the Comparative Toxicogenomics Database January 7, 2025 (17601) (Davis *et al*., 2019), and diseases from the KEGG database (Release 113.0+/01-01, Jan 25) (Kanehisa *et al*., 2000; Kanehisa *et al*., 2002). In summary, **15** pathways were found to be significantly impacted. In addition, **871** Gene Ontology (GO) terms, **0** miRNAs , **86** gene upstream regulators, **156** chemical upstream regulators and **96** diseases were found to be significantly enriched before the correction for multiple comparisons.

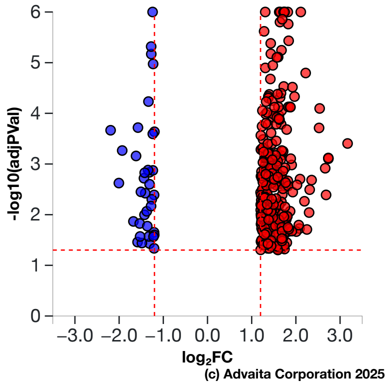


a)

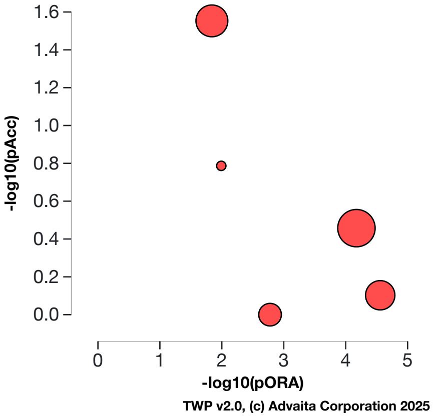


b)

**Fig. 1.1: a) Volcano plot:** *All **336** significantly differentially expressed (DE) genes are represented in terms of their measured expression change (x-axis) and the significance of the change (y-axis). The significance is represented in terms of the negative log (base 10) of the p-value, so that more significant genes are plotted higher on the y-axis. The dotted lines represent the thresholds used to select the DE genes: **1.2** for expression change and **0.05** for significance. The up-regulated genes (positive log fold change) are shown in red, while the down-regulated genes are blue.* **b) Pathways perturbation vs over-representation:** *The top 5 pathways are plotted in terms of the two types of evidence computed by iPathwayGuide: over-representation on the x-axis (pORA) and the total pathway accumulation on the y-axis (pAcc). Each pathway is represented by a single dot, with significant pathways shown in red, non-significant in black, and the size of each dot is proportional to the size of the pathway it represents. Both p-values are shown in terms of their negative log (base 10) values.*

# 2. Pathway Analysis

## 2.1. Methods

iPathwayGuide scores pathways using the Impact Analysis method (Draghici *et al*., 2007; Tarca *et al*., 2009, Khatri *et al*., 2007). Impact analysis uses two types of evidence: i) the over-representation of differentially expressed (DE) genes in a given pathway and ii) the perturbation of that pathway computed by propagating the measured expression changes across the pathway topology. These aspects are captured by two independent probability values, pORA and pAcc, that are then combined in a unique pathway-specific p-value. The underlying pathway topologies, comprised of genes and their directional interactions, are obtained from the KEGG database (Kanehisa *et al*., 2000; Kanehisa *et al*., 2010; Kanehisa *et al*., 2012; Kanehisa *et al*., 2014).

The first probability, pORA, expresses the probability of observing the number of DE genes in a given pathway that is greater than or equal to the number observed, by random chance (Draghici *et al*., 2003; Draghici 2011). Let us consider there are *N* genes measured in the experiment, with *M* of these on the given pathway. Based on the user-defined a priori selection of DE genes, *K* out of *M* genes were found to be differentially expressed. The probability of observing exactly *x* differentially expressed genes on the given pathway is computed based on the hypergeometric distribution:

Because the hypergeometric distribution is discrete, the probability of observing fewer than *x* genes on the given pathway just by chance can be calculated by summing the probabilities of randomly observing *0*, *1*, *2*, *...*, up to *x-1* DE genes on the pathway:

iPathwayGuide calculates the probability of randomly observing a number of DE genes on the given pathway that is greater than or equal to the number of DE genes obtained from data, by computing the over-representation p-value: $pORA = p_o(x) = 1 - p_u(x-1)$:

The second probability, pAcc, is calculated based on the amount of total accumulation measured in each pathway. A perturbation factor is computed for each gene on the pathway using:

In Equation 4, *PF(g)* is the perturbation factor for gene *g*, the term *ΔE(g)* represents the signed normalized measured expression change of gene *g*, and *α(g)* is a priori weight based on the type of the gene. The last term is the sum of the perturbation factors of all genes *u*, directly upstream of the target gene *g*, normalized by the number of downstream genes of each such gene $N_{ds}(u)$. The value of $\beta_{ug}$ quantifies the strength of the interaction between genes *g* and *u*. The sign of *β* represents the type of interaction: positive for activation-like signals, and negative for inhibition-like signals. Subsequently, iPathwayGuide calculates the accumulation at the level of each gene, *Acc(g)*, as the difference between the perturbation factor *PF(g)* and the observed log fold-change:

All perturbation accumulations are computed at the same time by solving the system of linear equations resulting from combining Equation 4 for all genes on a given pathway. Once all gene perturbation accumulations are computed, iPathwayGuide computes the total accumulation of the pathway as the sum of all absolute accumulations of the genes in a given pathway. The significance of obtaining a total accumulation (pAcc) at least as large as observed, just by chance, is assessed through bootstrap analysis.

The two types of evidence, pORA and pAcc, are combined into an overall pathway score by calculating a p-value using Fisher's method. This p-value is then corrected for multiple comparisons using false dicovery rate (FDR) and Bonferroni corrections. Bonferroni is simpler and more conservative of the two (Bonferroni, 1935; Bonferroni, 1936). It reduces the false discovery rate by imposing a stringent threshold on each comparison adjusted for the total number of comparisons. The FDR correction has more power, but only controls the family-wise false positives rate (Benjamini and Hochberg, 1995; Benjamini and Yekutieli, 2001).

# 2.2. Results

**Table 2.2.1: Top pathways and their associated p-values**

| Pathway name | Pathway Id | p-value | p-value (FDR) | p-value (Bonferroni) |
|---|---|---|---|---|
| Glutamatergic synapse | 04724 | 2.566e-4 | 0.032 | 0.060 |
| Axon guidance | 04360 | 2.729e-4 | 0.032 | 0.063 |
| Natural killer cell mediated cytotoxicity | 04650 | 0.004 | 0.276 | 0.827 |
| Synaptic vesicle cycle * | 04721 | 0.007 | 0.391 | 1.000 |
| African trypanosomiasis | 05143 | 0.012 | 0.456 | 1.000 |

* the p-value corresponding to the pathway was computed using only over-representation analysis.

## Glutamatergic synapse (KEGG: 04724)

Glutamate is the major excitatory neurotransmitter in the mammalian central nervous system(CNS). Glutamate is packaged into synaptic vesicles in the presynaptic terminal. Once released into the synaptic cleft, glutamate acts on postsynaptic ionotropic glutamate receptors (iGluRs) to mediate fast excitatory synaptic transmission. Glutamate can also act on metabotropic glutamate receptors (mGluRs) and exert a variety of modulatory effects through their coupling to G proteins and the subsequent recruitment of second messenger systems. Presynaptically localized Group II and Group III mGluRs are thought to represent the classical inhibitory autoreceptor mechanism that suppresses excess glutamate release. After its action on these receptors, glutamate can be removed from the synaptic cleft by EAATs located either on the presynaptic terminal, neighboring glial cells, or the postsynaptic neuron. In glia, glutamate is converted to glutamine, which is then transported back to the presynaptic terminal and converted back to glutamate.

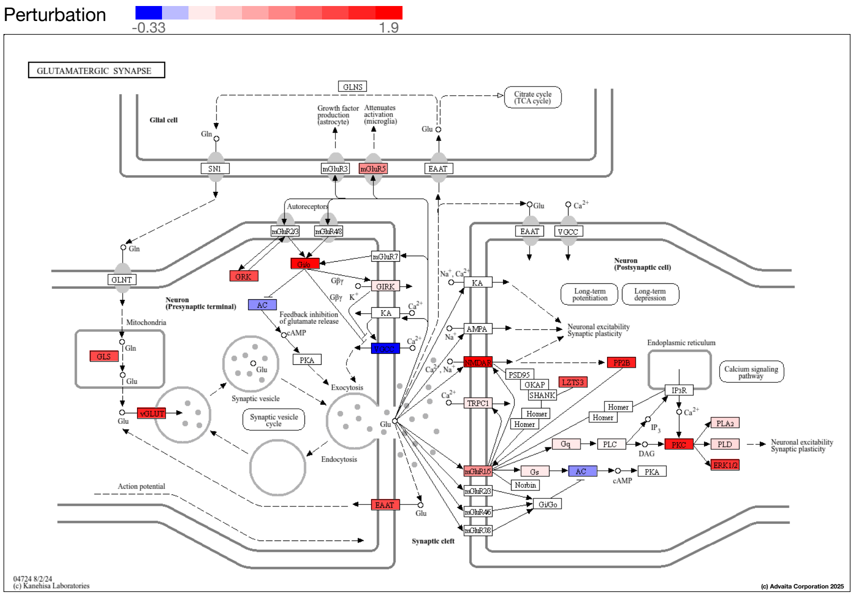


**Fig. 2.2.1: Glutamatergic synapse (KEGG: 04724):** *The pathway diagram is overlayed with the computed perturbation of each gene. The perturbation accounts both for the gene's measured fold change and for the accumulated perturbation propagated from any upstream genes (accumulation). The highest negative perturbation is shown in dark blue, while the highest positive perturbation in dark red. The legend describes the values on the gradient. Note: For legibility, one gene may be represented in multiple places in the diagram and one box may represent multiple genes in the same gene family. A gene is highlighted in all locations it occurs in the diagram. For each gene family, the color corresponding to the gene with the highest absolute perturbation is displayed.*

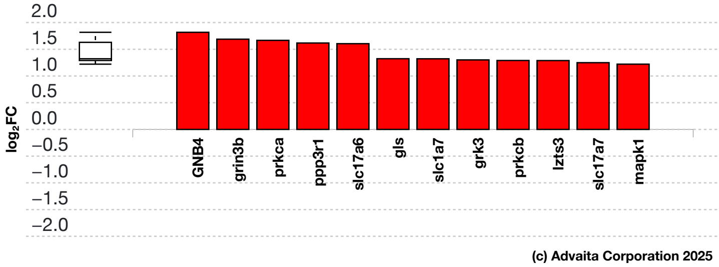


**Fig. 2.2.2: Gene measured expression bar plot:** *All the differentially expressed genes in Glutamatergic synapse (KEGG: 04724) are ranked based on their absolute value of log fold change. Upregulated genes are shown in red, downregulated genes are shown in blue. The box and whisker plot on the left summarizes the distribution of all the differentially expressed genes in this pathway. The box represents the 1st quartile, the median and the 3rd quartile, while the outliers are represented by circles.*

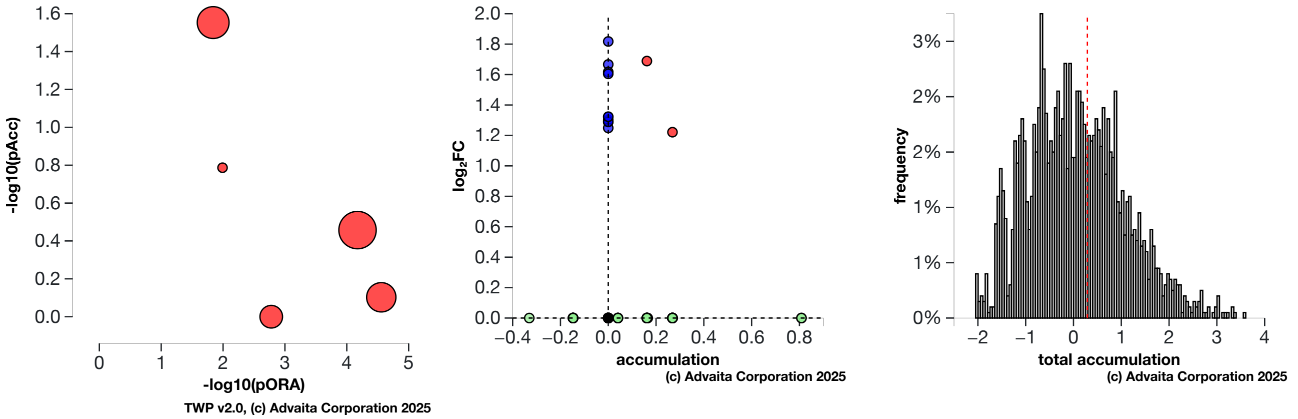


a) b) c)

**Fig. 2.2.3: a) Perturbation vs over-representation:** *Glutamatergic synapse (KEGG: 04724) (yellow) is shown, using negative log of the accumulation and over-representation p-values, along with the other most significant pathways. Pathways in red are significant based on the combined uncorrected p-values, whereas the ones in black are non-significant (where applicable).* **b) Gene measured expression vs accumulation:** *All the genes from this pathway are represented in terms of their measured fold change (y-axis) and accumulation (x-axis). Accumulation is the perturbation received by the gene from any upstream genes. Genes displayed in red had both accumulation and measured fold change.Genes in blue had only measured fold change.Genes in green had only accumulation.The remaining genes that were not measured and had no accumulation are shown in black.* **c) Bootstrap diagram:** *The perturbation p-value is computed using bootstrap analysis. Bootstrapping assesses the probability of observing a sum of all absolute gene accumulation total accumulation at least as extreme as the computed one just by chance. A null distribution (gray bars) is computed through an iterative process that is repeated 2000 times. At each iteration, a number of genes equal to the number of differentially expressed genes in this pathway is randomly assigned anywhere in the pathway and the total accumulation is recomputed. The red line indicates the observed total accumulation of genes in the given pathway in relation to the distribution of expected values. The perturbation p-value is more significant the further away from the mean it is.*

## Axon guidance (KEGG: 04360)

Axon guidance represents a key stage in the formation of neuronal network. Axons are guided by a variety of guidance factors, such as netrins, ephrins, Slits, and semaphorins. These guidance cues are read by growth cone receptors, and signal transduction pathways downstream of these receptors converge onto the Rho GTPases to elicit changes in cytoskeletal organization that determine which way the growth cone will turn.

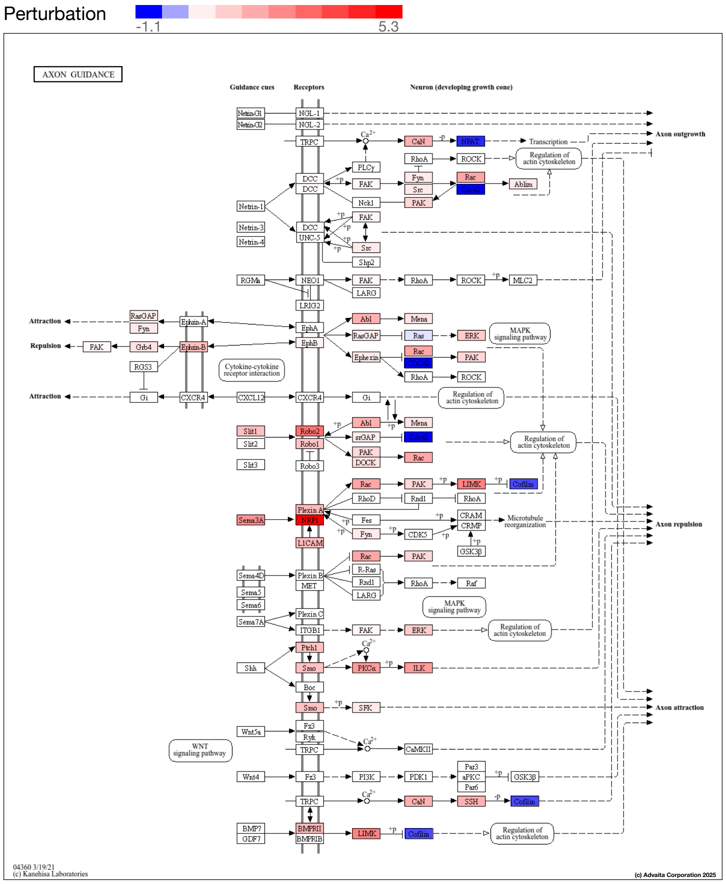


**Fig. 2.2.4: Axon guidance (KEGG: 04360):** *The pathway diagram is overlayed with the computed perturbation of each gene. The perturbation accounts both for the gene's measured fold change and for the accumulated perturbation propagated from any upstream genes (accumulation). The highest negative perturbation is shown in dark blue, while the highest positive perturbation in dark red. The legend describes the values on the gradient. Note: For legibility, one gene may be represented in multiple places in the diagram and one box may represent multiple genes in the same gene family. A gene is highlighted in all locations it occurs in the diagram. For each gene family, the color corresponding to the gene with the highest absolute perturbation is displayed.*

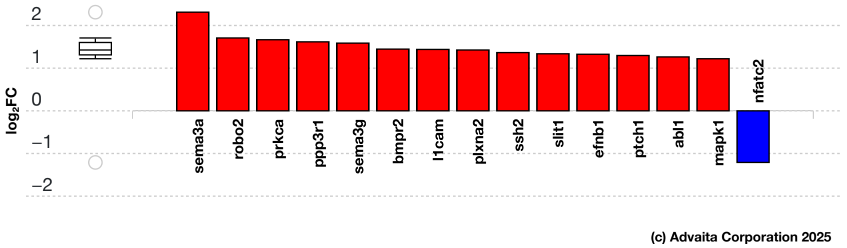


**Fig. 2.2.5: Gene measured expression bar plot:** *All the differentially expressed genes in Axon guidance (KEGG: 04360) are ranked based on their absolute value of log fold change. Upregulated genes are shown in red, downregulated genes are shown in blue. The box and whisker plot on the left summarizes the distribution of all the differentially expressed genes in this pathway. The box represents the 1st quartile, the median and the 3rd quartile, while the outliers are represented by circles.*

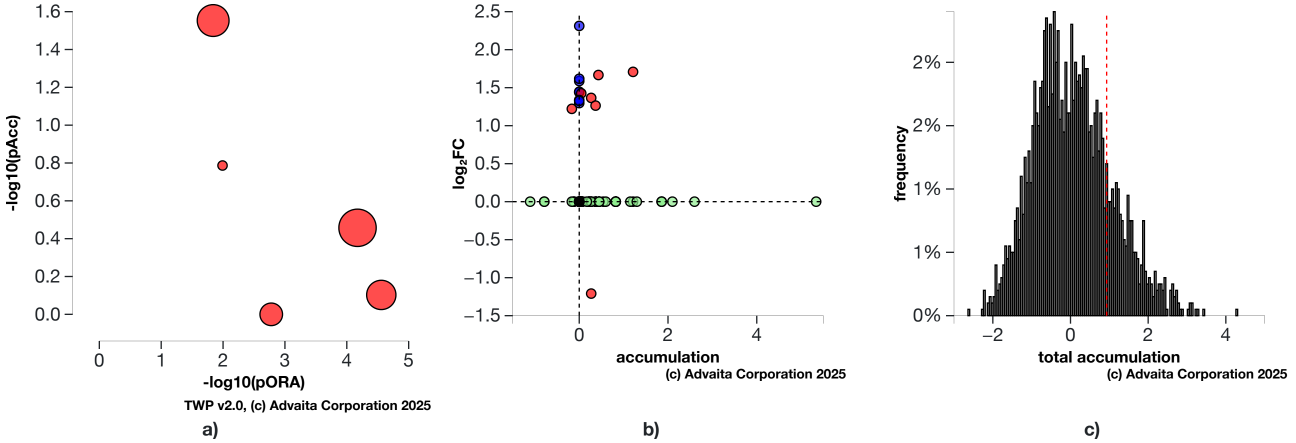


**Fig. 2.2.6: a) Perturbation vs over-representation:** *Axon guidance (KEGG: 04360) (yellow) is shown, using negative log of the accumulation and over-representation p-values, along with the other most significant pathways. Pathways in red are significant based on the combined uncorrected p-values, whereas the ones in black are non-significant (where applicable).* **b) Gene measured expression vs accumulation:** *All the genes from this pathway are represented in terms of their measured fold change (y-axis) and accumulation (x-axis). Accumulation is the perturbation received by the gene from any upstream genes. Genes displayed in red had both accumulation and measured fold change.Genes in blue had only measured fold change.Genes in green had only accumulation.The remaining genes that were not measured and had no accumulation are shown in black.* **c) Bootstrap diagram:** *The perturbation p-value is computed using bootstrap analysis. Bootstrapping assesses the probability of observing a sum of all absolute gene accumulation total accumulation at least as extreme as the computed one just by chance. A null distribution (gray bars) is computed through an iterative process that is repeated 2000 times. At each iteration, a number of genes equal to the number of differentially expressed genes in this pathway is randomly assigned anywhere in the pathway and the total accumulation is recomputed. The red line indicates the observed total accumulation of genes in the given pathway in relation to the distribution of expected values. The perturbation p-value is more significant the further away from the mean it is.*

## Natural killer cell mediated cytotoxicity (KEGG: 04650)

Natural killer (NK) cells are lymphocytes of the innate immune system that are involved in early defenses against both allogeneic (nonself) cells and autologous cells undergoing various forms of stress, such as infection with viruses, bacteria, or parasites or malignant transformation. Although NK cells do not express classical antigen receptors of the immunoglobulin gene family, such as the antibodies produced by B cells or the T cell receptor expressed by T cells, they are equipped with various receptors whose engagement allows them to discriminate between target and nontarget cells. Activating receptors bind ligands on the target cell surface and trigger NK cell activation and target cell lysis. However Inhibitory receptors recognize MHC class I molecules (HLA) and inhibit killing by NK cells by overruling the actions of the activating receptors. This inhibitory signal is lost when the target cells do not express MHC class I and perhaps also in cells infected with virus, which might inhibit MHC class I exprssion or alter its conformation. The mechanism of NK cell killing is the same as that used by the cytotoxic T cells generated in an adaptive immune response; cytotoxic granules are released onto the surface of the bound target cell, and the effector proteins they contain penetrate the cell membrane and induce programmed cell death.

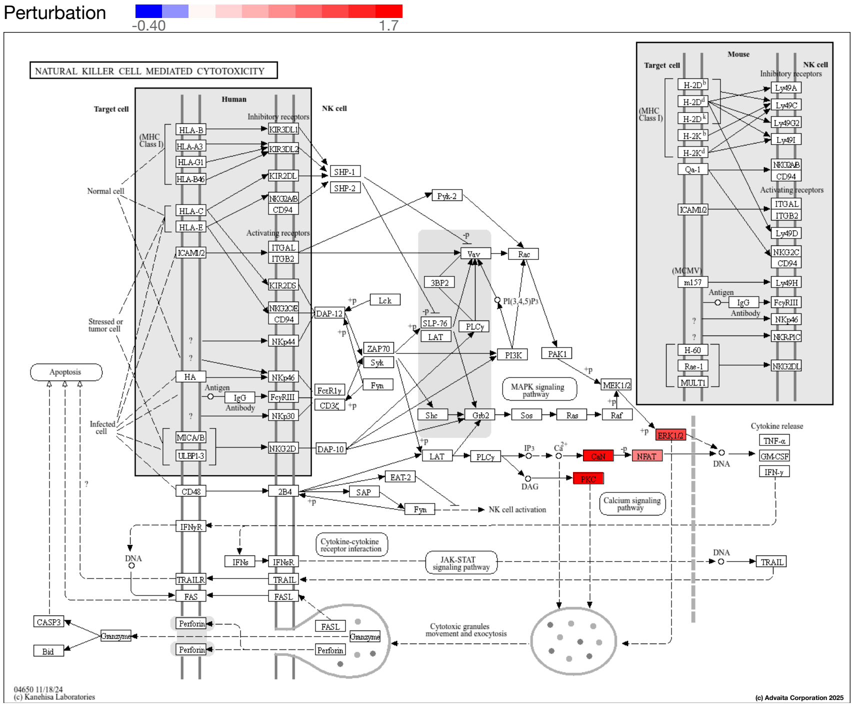


**Fig. 2.2.7: Natural killer cell mediated cytotoxicity (KEGG: 04650):** *The pathway diagram is overlayed with the computed perturbation of each gene. The perturbation accounts both for the gene's measured fold change and for the accumulated perturbation propagated from any upstream genes (accumulation). The highest negative perturbation is shown in dark blue, while the highest positive perturbation in dark red. The legend describes the values on the gradient. Note: For legibility, one gene may be represented in multiple places in the diagram and one box may represent multiple genes in the same gene family. A gene is highlighted in all locations it occurs in the diagram. For each gene family, the color corresponding to the gene with the highest absolute perturbation is displayed.*

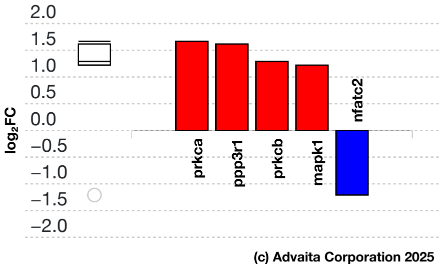


**Fig. 2.2.8: Gene measured expression bar plot:** *All the differentially expressed genes in Natural killer cell mediated cytotoxicity (KEGG: 04650) are ranked based on their absolute value of log fold change. Upregulated genes are shown in red, downregulated genes are shown in blue. The box and whisker plot on the left summarizes the distribution of all the differentially expressed genes in this pathway. The box represents the 1st quartile, the median and the 3rd quartile, while the outliers are represented by circles.*

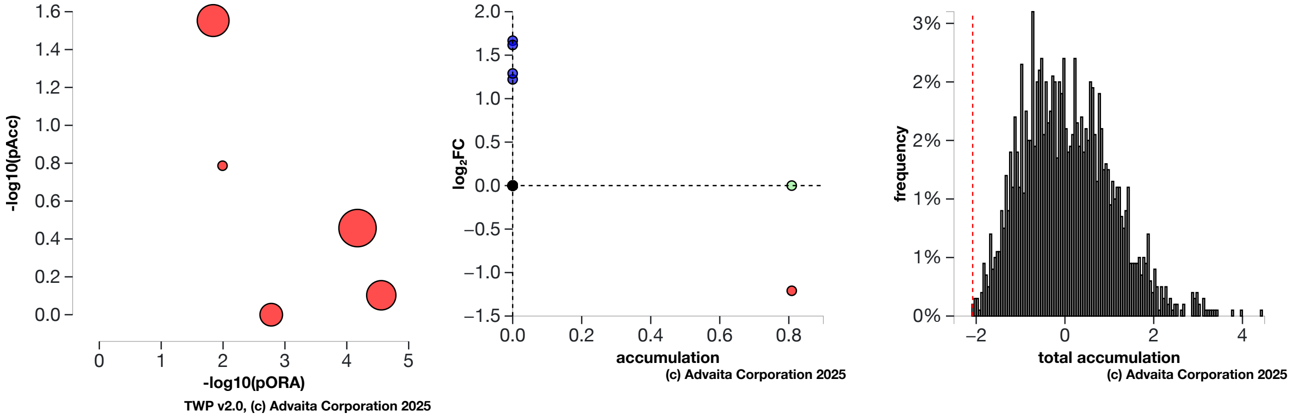


a) b) c)

**Fig. 2.2.9: a) Perturbation vs over-representation:** *Natural killer cell mediated cytotoxicity (KEGG: 04650) (yellow) is shown, using negative log of the accumulation and over-representation p-values, along with the other most significant pathways. Pathways in red are significant based on the combined uncorrected p-values, whereas the ones in black are non-significant (where applicable).* **b) Gene measured expression vs accumulation:** *All the genes from this pathway are represented in terms of their measured fold change (y-axis) and accumulation (x-axis). Accumulation is the perturbation received by the gene from any upstream genes. Genes displayed in red had both accumulation and measured fold change.Genes in blue had only measured fold change.Genes in green had only accumulation.The remaining genes that were not measured and had no accumulation are shown in black.* **c) Bootstrap diagram:** *The perturbation p-value is computed using bootstrap analysis. Bootstrapping assesses the probability of observing a sum of all absolute gene accumulation total accumulation at least as extreme as the computed one just by chance. A null distribution (gray bars) is computed through an iterative process that is repeated 2000 times. At each iteration, a number of genes equal to the number of differentially expressed genes in this pathway is randomly assigned anywhere in the pathway and the total accumulation is recomputed. The red line indicates the observed total accumulation of genes in the given pathway in relation to the distribution of expected values. The perturbation p-value is more significant the further away from the mean it is.*

## Synaptic vesicle cycle (KEGG: 04721)

Communication between neurons is mediated by the release of neurotransmitter from synaptic vesicles (SVs). At the nerve terminal, SVs cycle through repetitive episodes of exocytosis and endocytosis. SVs are filled with neurotransmitters by active transport. The loaded SVs are then docked at a specialized region of the presynaptic plasma membrane known as the active zone, where they undergo a priming reaction. Upon arrival of an action potential, Ca2+ enters through voltage-gated channels and neurotransmitter is released by exocytosis, usually in less than a millisecond. After fusion, the vesicle is retrieved by endocytosis and reloaded for another round of exocytosis.

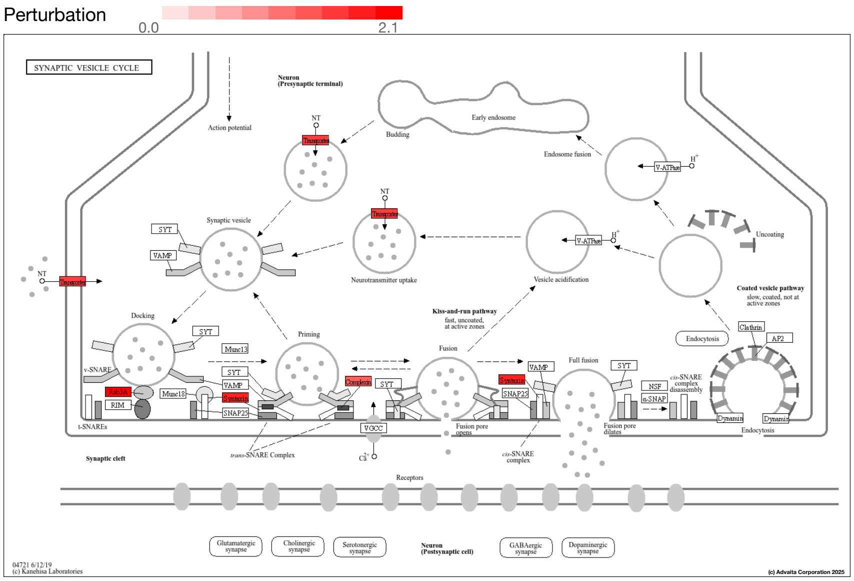


**Fig. 2.2.10: Synaptic vesicle cycle (KEGG: 04721):** *The pathway diagram is overlayed with the computed perturbation of each gene. The perturbation accounts both for the gene's measured fold change and for the accumulated perturbation propagated from any upstream genes (accumulation). The highest negative perturbation is shown in dark blue, while the highest positive perturbation in dark red. The legend describes the values on the gradient. Note: For legibility, one gene may be represented in multiple places in the diagram and one box may represent multiple genes in the same gene family. A gene is highlighted in all locations it occurs in the diagram. For each gene family, the color corresponding to the gene with the highest absolute perturbation is displayed.*

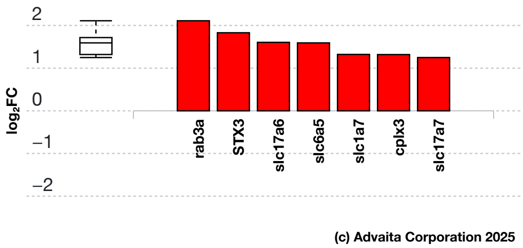


**Fig. 2.2.11: Gene measured expression bar plot:** *All the differentially expressed genes in Synaptic vesicle cycle (KEGG: 04721) are ranked based on their absolute value of log fold change. Upregulated genes are shown in red, downregulated genes are shown in blue. The box and whisker plot on the left summarizes the distribution of all the differentially expressed genes in this pathway. The box represents the 1st quartile, the median and the 3rd quartile, while the outliers are represented by circles.*

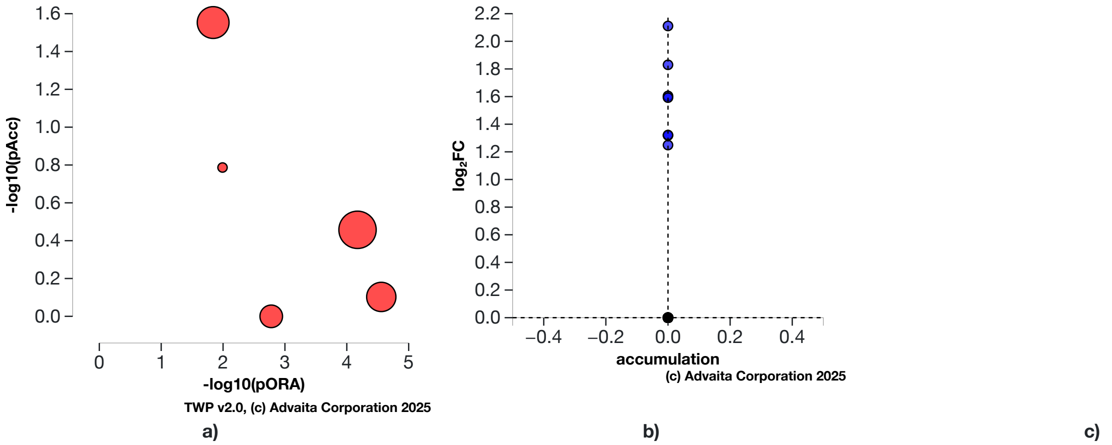


**Fig. 2.2.12: a) Perturbation vs over-representation:** *Synaptic vesicle cycle (KEGG: 04721) (yellow) is shown, using negative log of the accumulation and over-representation p-values, along with the other most significant pathways. Pathways in red are significant based on the combined uncorrected p-values, whereas the ones in black are non-significant (where applicable).* **b) Gene measured expression vs accumulation:** *All the genes from this pathway are represented in terms of their measured fold change (y-axis) and accumulation (x-axis). Accumulation is the perturbation received by the gene from any upstream genes. Genes in blue had only measured fold change.The remaining genes that were not measured and had no accumulation are shown in black.* **c) Bootstrap diagram:** *This pathway was not suitable for impact analysis. No perturbation p-value was computed for it.*

## African trypanosomiasis (KEGG: 05143)

Trypanosoma brucei, the parasite responsible for African trypanosomiasis (sleeping sickness), are spread by the tsetse fly in sub-Saharan Africa. The parasites are able to pass through the blood-brain barrier and cause neurological damage by inducing cytokines like TNF alpha, IFN gamma, and IL1. These cytokines and other metabolites such as nitric oxide and somnogenic prostaglandin D2 disturb circadian rhythms in patients with African trypanosomiasis.

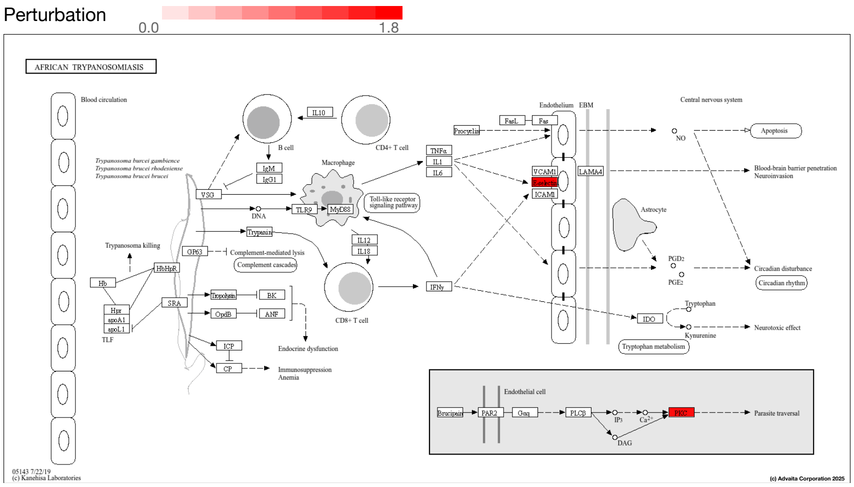


**Fig. 2.2.13: African trypanosomiasis (KEGG: 05143):** *The pathway diagram is overlayed with the computed perturbation of each gene. The perturbation accounts both for the gene's measured fold change and for the accumulated perturbation propagated from any upstream genes (accumulation). The highest negative perturbation is shown in dark blue, while the highest positive perturbation in dark red. The legend describes the values on the gradient. Note: For legibility, one gene may be represented in multiple places in the diagram and one box may represent multiple genes in the same gene family. A gene is highlighted in all locations it occurs in the diagram. For each gene family, the color corresponding to the gene with the highest absolute perturbation is displayed.*

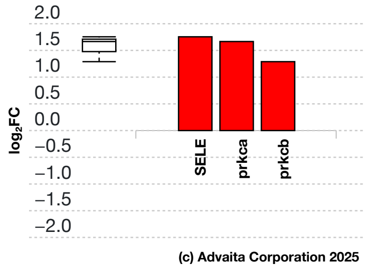


**Fig. 2.2.14: Gene measured expression bar plot:** *All the differentially expressed genes in African trypanosomiasis (KEGG: 05143) are ranked based on their absolute value of log fold change. Upregulated genes are shown in red, downregulated genes are shown in blue. The box and whisker plot on the left summarizes the distribution of all the differentially expressed genes in this pathway. The box represents the 1st quartile, the median and the 3rd quartile, while the outliers are represented by circles.*

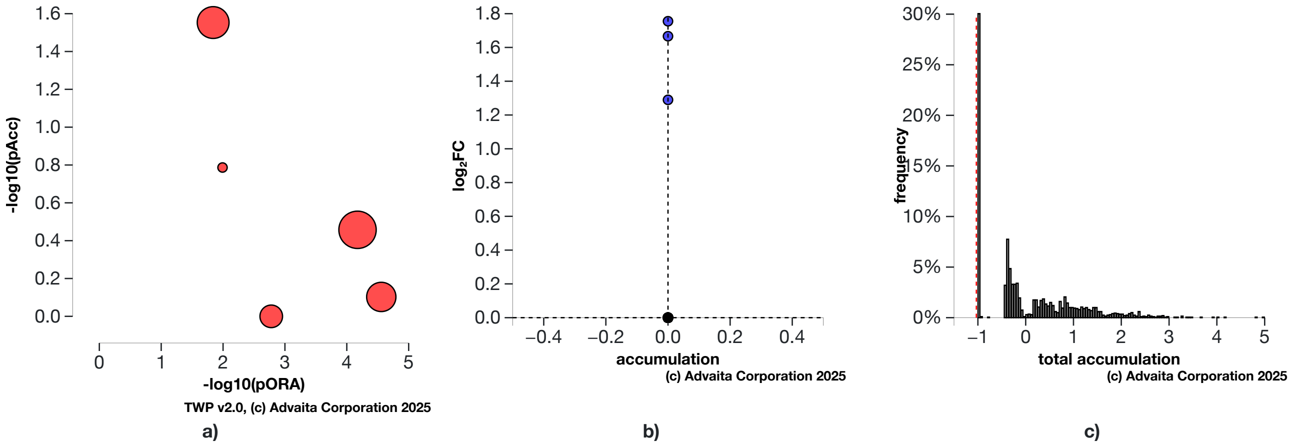


**Fig. 2.2.15: a) Perturbation vs over-representation:** *African trypanosomiasis (KEGG: 05143) (yellow) is shown, using negative log of the accumulation and over-representation p-values, along with the other most significant pathways. Pathways in red are significant based on the combined uncorrected p-values, whereas the ones in black are non-significant (where applicable).* **b) Gene measured expression vs accumulation:** *All the genes from this pathway are represented in terms of their measured fold change (y-axis) and accumulation (x-axis). Accumulation is the perturbation received by the gene from any upstream genes. Genes in blue had only measured fold change.The remaining genes that were not measured and had no accumulation are shown in black.* **c) Bootstrap diagram:** *The perturbation p-value is computed using bootstrap analysis. Bootstrapping assesses the probability of observing a sum of all absolute gene accumulation total accumulation at least as extreme as the computed one just by chance. A null distribution (gray bars) is computed through an iterative process that is repeated 2000 times. At each iteration, a number of genes equal to the number of differentially expressed genes in this pathway is randomly assigned anywhere in the pathway and the total accumulation is recomputed. The red line indicates the observed total accumulation of genes in the given pathway in relation to the distribution of expected values. The perturbation p-value is more significant the further away from the mean it is.*

# 3. Gene Ontology Analysis

## 3.1. Methods

For each Gene Ontology (GO) term (Ashburner *et al*., 2002; Gene Ontology Consortium, 2004), the number of differentially expressed (DE) genes annotated to the term is compared to the number of DE genes expected just by chance. iPathwayGuide uses an over-representation approach to compute the statistical significance of observing at least the given number of DE genes. The p-value is computed using the hypergeometric distribution as described for pORA in the Pathway Analysis section. This p-value is corrected for multiple comparisons using FDR and Bonferroni.

The classical enrichment method used above considers all GO terms to be independent. By definition, all genes annotated to a GO term are also annotated to its ancestors. Because of this, the enrichment approach counts each gene multiple times by propagating it through the GO hierarchy from the most specific term the gene is associated with, all the way to the root of the ontology. This introduces redundancy in the analysis and reports many general and non-informative terms as significant. To overcome this limitation, iPathwayGuide allows users to use two more sophisticated pruning methods: *high-specificity pruning* and *smallest common denominator pruning*. The **high-specificity** pruning method *identifies the most specific GO terms* that are significantly associated with the set of DE genes. Let us consider, BP1 = “induction of apoptosis by intracellular signals” and BP2 = “induction of apoptosis by extracellular signals,” which are two of the children of BP3 = “induction of apoptosis.” If enough DE genes are associated with BP1 and BP2, the high-specificity pruning will report them as significant. The **smallest common denominator** pruning method *identifies the GO terms that best encapsulate the set of DE genes*, at times consolidating significance of two or more specific terms into their common parent. In the example above, this pruning method might report BP3 as significant because it is the most specific biological term that would include all DE genes that make both BP1 and BP2 significant.

# 3.2. Biological Processes results

**Table 3.2.1: Top identified biological processes. Only the top scoring biological process for each pruning type is described below the table.**

| Pruning Type: None | | | | Pruning Type: High-specificity | | Pruning Type: Smallest Common Denominator | |
|---|---|---|---|---|---|---|---|
| GO Term | p-value | p-value (FDR) | p-value (Bonferroni) | GO Term | p-value | GO Term | p-value |
| cell communication | 7.200e-10 | 2.499e-6 | 3.271e-6 | adult locomotory behavior | 0.150 | regulation of synapse structure or activity | 0.045 |
| signaling | 1.100e-9 | 2.499e-6 | 4.997e-6 | synaptic signaling | 0.729 | synapse assembly | 0.045 |
| synaptic signaling | 3.200e-9 | 4.846e-6 | 1.454e-5 | chemical synaptic transmission | 0.729 | adult locomotory behavior | 0.050 |
| regulation of cell communication | 6.600e-9 | 7.496e-6 | 2.998e-5 | nervous system development | 0.729 | synaptic vesicle exocytosis | 0.159 |
| regulation of signaling | 1.300e-8 | 1.181e-5 | 5.906e-5 | neurogenesis | 0.729 | regulation of axon extension | 0.159 |

## cell communication (GO:0007154)

Any process that mediates interactions between a cell and its surroundings. Encompasses interactions such as signaling or attachment between one cell and another cell, between a cell and an extracellular matrix, or between a cell and any other aspect of its environment. In this experiment, the algorithm identified **169** differentially expressed gene(s) out of ALL **4,178** gene(s).

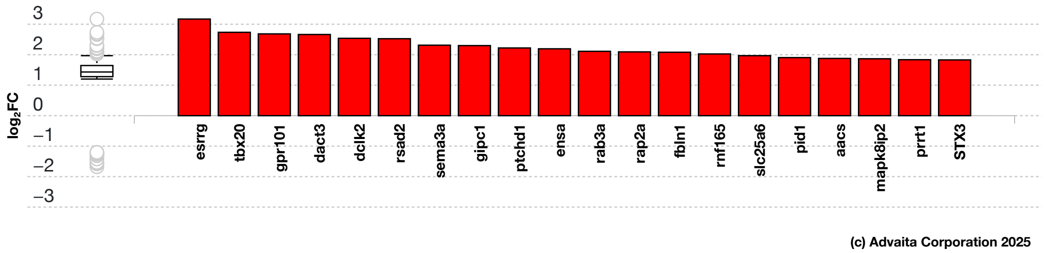


**Fig. 3.2.1: Gene measured expression bar plot:** *All the differentially expressed genes that are annotated to cell communication are ranked based on their absolute value of log fold change. The plot is limited to the top 20 genes out of a total of 169 differentially expressed genes. Upregulated genes are shown in red, downregulated genes are shown in blue. The box and whisker plot on the left summarizes the distribution of all the differentially expressed genes that are annotated to this GO term. The box represents the 1st quartile, the median and the 3rd quartile, while the outliers are represented by circles.*

## adult locomotory behavior (GO:0008344)

Locomotory behavior in a fully developed and mature organism. In this experiment, the algorithm identified **10** differentially expressed gene(s) out of ALL **73** gene(s).

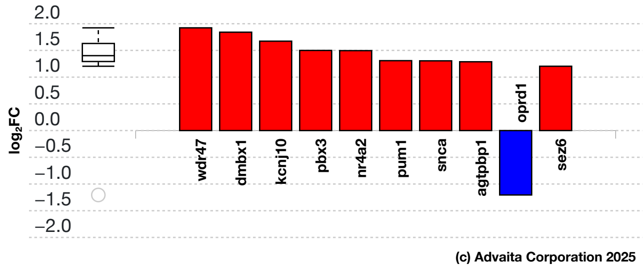


**Fig. 3.2.2: Gene measured expression bar plot:** *All the differentially expressed genes that are annotated to adult locomotory behavior are ranked based on their absolute value of log fold change. Upregulated genes are shown in red, downregulated genes are shown in blue. The box and whisker plot on the left summarizes the distribution of all the differentially expressed genes that are annotated to this GO term. The box represents the 1st quartile, the median and the 3rd quartile, while the outliers are represented by circles.*

## regulation of synapse structure or activity (GO:0050803)

Any process that modulates the physical form or the activity of a synapse, the junction between a neuron and a target (neuron, muscle, or secretory cell). In this experiment, the algorithm identified **23** differentially expressed gene(s) out of ALL **266** gene(s).

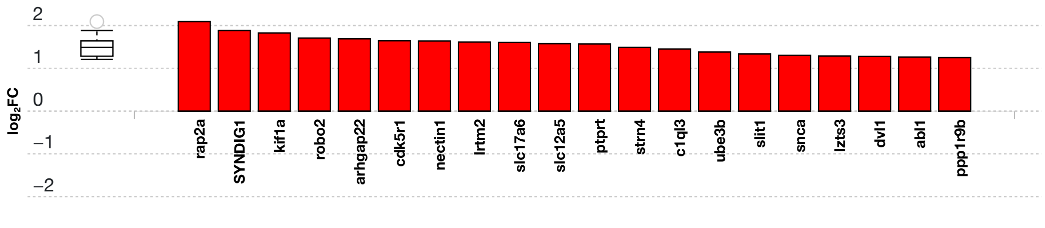


**Fig. 3.2.3: Gene measured expression bar plot:** *All the differentially expressed genes that are annotated to regulation of synapse structure or activity are ranked based on their absolute value of log fold change. The plot is limited to the top 20 genes out of a total of 23 differentially expressed genes. Upregulated genes are shown in red, downregulated genes are shown in blue. The box and whisker plot on the left summarizes the distribution of all the differentially expressed genes that are annotated to this GO term. The box represents the 1st quartile, the median and the 3rd quartile, while the outliers are represented by circles.*

# 3.3. Molecular Functions results

**Table 3.3.1: Top identified molecular functions. Only the top scoring molecular function for each pruning type is described below the table.**

| Pruning Type: None | | | | Pruning Type: High-specificity | | Pruning Type: Smallest Common Denominator | |
|---|---|---|---|---|---|---|---|
| **GO Term** | **p-value** | **p-value (FDR)** | **p-value (Bonferroni)** | **GO Term** | **p-value** | **GO Term** | **p-value** |
| signaling receptor activity | **3.700e-5** | **0.016** | **0.032** | sialic acid binding | 0.230 | phosphatase binding | **0.105** |
| molecular transducer activity | **3.700e-5** | **0.016** | **0.032** | calcium,diacylglycerol-dependent serine/threonine kinase activity | 0.230 | sialic acid binding | **0.173** |
| transmembrane signaling receptor activity | **1.000e-4** | **0.026** | 0.087 | histone H3T6 kinase activity | 0.230 | calcium,diacylglycerol-dependent serine/threonine kinase activity | **0.173** |
| phosphatase binding | **1.200e-4** | **0.026** | 0.105 | delta-catenin binding | 0.255 | histone H3T6 kinase activity | 0.173 |
| sialic acid binding | **4.200e-4** | 0.069 | 0.367 | small GTPase binding | 0.255 | protein phosphatase regulator activity | 0.175 |

## signaling receptor activity (GO:0038023)

Receiving a signal and transmitting it in the cell to initiate a change in cell activity. A signal is a physical entity or change in state that is used to transfer information in order to trigger a response. In this experiment, the algorithm identified **34** differentially expressed gene(s) out of ALL **578** gene(s).

**Fig. 3.3.4: Gene measured expression bar plot:** *All the differentially expressed genes that are annotated to signaling receptor activity are ranked based on their absolute value of log fold change. The plot is limited to the top 20 genes out of a total of 34 differentially expressed genes. Upregulated genes are shown in red, downregulated genes are shown in blue. The box and whisker plot on the left summarizes the distribution of all the differentially expressed genes that are annotated to this GO term. The box represents the 1st quartile, the median and the 3rd quartile, while the outliers are represented by circles.*

## sialic acid binding (GO:0033691)

Binding to a sialic acid, a N- or O- substituted derivative of neuraminic acid, a nine carbon monosaccharide. Sialic acids often occur in polysaccharides, glycoproteins, and glycolipids in animals and bacteria. In this experiment, the algorithm identified **3** differentially expressed gene(s) out of ALL **6** gene(s).

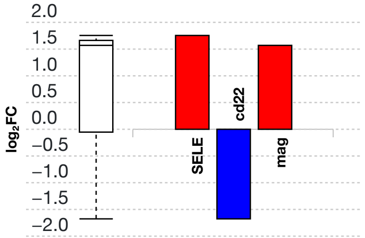


**Fig. 3.3.5: Gene measured expression bar plot:** *All the differentially expressed genes that are annotated to sialic acid binding are ranked based on their absolute value of log fold change. Upregulated genes are shown in red, downregulated genes are shown in blue. The box and whisker plot on the left summarizes the distribution of all the differentially expressed genes that are annotated to this GO term. The box represents the 1st quartile, the median and the 3rd quartile, while the outliers are represented by circles.*

## phosphatase binding (GO:0019902)

Binding to a phosphatase. In this experiment, the algorithm identified **14** differentially expressed gene(s) out of ALL **155** gene(s).

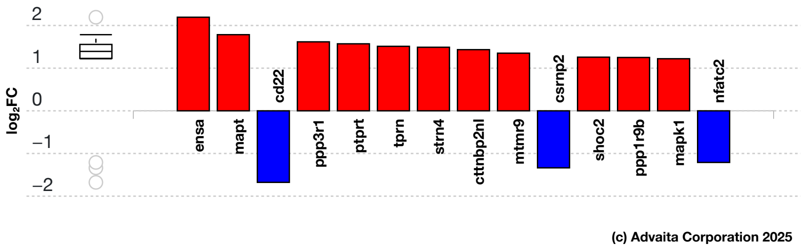


**Fig. 3.3.6: Gene measured expression bar plot:** *All the differentially expressed genes that are annotated to phosphatase binding are ranked based on their absolute value of log fold change. Upregulated genes are shown in red, downregulated genes are shown in blue. The box and whisker plot on the left summarizes the distribution of all the differentially expressed genes that are annotated to this GO term. The box represents the 1st quartile, the median and the 3rd quartile, while the outliers are represented by circles.*

## 3.4. Cellular Components results

**Table 3.4.1: Top identified cellular components. Only the top scoring cellular component for each pruning type is described below the table.**

| Pruning Type: None | | | | Pruning Type: High-specificity | | Pruning Type: Smallest Common Denominator | |
|---|---|---|---|---|---|---|---|
| **GO Term** | **p-value** | **p-value (FDR)** | **p-value (Bonferroni)** | **GO Term** | **p-value** | **GO Term** | **p-value** |
| synapse | **8.000e-14** | **2.056e-11** | **4.112e-11** | synaptic vesicle membrane | 8.224e-4 | synapse | **5.140e-6** |
| cell projection | **8.500e-14** | **2.056e-11** | **4.369e-11** | dendritic spine | 0.004 | axon | **5.140e-6** |
| neuron projection | **1.200e-13** | **2.056e-11** | **6.168e-11** | growth cone | 0.004 | dendrite | **8.395e-6** |
| plasma membrane bounded cell projection | **3.200e-12** | **4.112e-10** | **1.645e-9** | axon terminus | 0.035 | plasma membrane region | 5.011e-5 |
| plasma membrane region | **4.000e-11** | **4.112e-9** | **2.056e-8** | plasma membrane | 0.093 | cell body | 0.007 |

### synapse (GO:0045202)

The junction between an axon of one neuron and a dendrite of another neuron, a muscle fiber or a glial cell. As the axon approaches the synapse it enlarges into a specialized structure, the presynaptic terminal bouton, which contains mitochondria and synaptic vesicles. At the tip of the terminal bouton is the presynaptic membrane; facing it, and separated from it by a minute cleft (the synaptic cleft) is a specialized area of membrane on the receiving cell, known as the postsynaptic membrane. In response to the arrival of nerve impulses, the presynaptic terminal bouton secretes molecules of neurotransmitters into the synaptic cleft. These diffuse across the cleft and transmit the signal to the postsynaptic membrane. In this experiment, the algorithm identified **87** differentially expressed gene(s) out of ALL **1,350** gene(s).

**Fig. 3.4.7: Gene measured expression bar plot:** *All the differentially expressed genes that are annotated to synapse are ranked based on their absolute value of log fold change. The plot is limited to the top 20 genes out of a total of 87 differentially expressed genes. Upregulated genes are shown in red, downregulated genes are shown in blue. The box and whisker plot on the left summarizes the distribution of all the differentially expressed genes that are annotated to this GO term. The box represents the 1st quartile, the median and the 3rd quartile, while the outliers are represented by circles.*

### synaptic vesicle membrane (GO:0030672)

The lipid bilayer surrounding a synaptic vesicle. In this experiment, the algorithm identified **15** differentially expressed gene(s) out of ALL **120** gene(s).

**Fig. 3.4.8: Gene measured expression bar plot:** *All the differentially expressed genes that are annotated to synaptic vesicle membrane are ranked based on their absolute value of log fold change. The plot is limited to the top 20 genes out of a total of 15 differentially expressed genes. Upregulated genes are shown in red, downregulated genes are shown in blue. The box and whisker plot on the left summarizes the distribution of all the differentially expressed genes that are annotated to this GO term. The box represents the 1st quartile, the median and the 3rd quartile, while the outliers are represented by circles.*

# 4. Predicted Upstream Regulator Analysis - miRNAs

## 4.1. Methods

The prediction of active miRNAs (Friedman *et al*., 2009; Lewis *et al*., 2005) is based on enrichment of differentially downregulated target genes of the miRNAs. In general, miRNAs have an inhibitory effect on their targets. Therefore, for any given miRNA the method computes the ratio between the number of differentially downregulated targets and all differentially expressed targets, and compares it to the ratio of all downwardly expressed targets to all targets. Overall, iPathwayGuide calculates the probability of observing at least the number of differentially downregulated target genes for a given miRNA just by chance. This p-value is computed using the hypergeometric distribution as described for pORA in the Pathway Analysis section.

# 4.2. Results

**Table 4.2.1: Top identified miRNAs**

| miRNA Name | p-value | p-value (FDR) | p-value (Bonferroni) |
|---|---|---|---|
| hsa-miR-127-3p | 0.789 | 1.000 | 1.000 |
| hsa-miR-122-5p | 0.866 | 1.000 | 1.000 |
| hsa-miR-100-5p | 0.897 | 1.000 | 1.000 |
| hsa-miR-99a-5p | 0.897 | 1.000 | 1.000 |
| hsa-miR-99b-5p | 0.897 | 1.000 | 1.000 |

## hsa-miR-127-3p (MIMAT0000446)

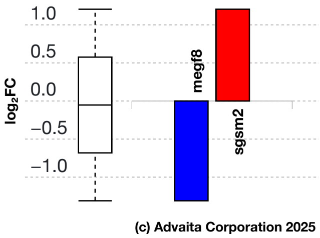


**Fig. 4.2.1: Gene measured expression bar plot:** *All the differentially expressed genes that are targeted by hsa-miR-127-3p are ranked based on their measured expression change (most downregulated to upregulated). The downregulated genes are shown in blue, and the upregulated ones are shown in red (where applicable). Out of all the differentially expressed target genes, 1 were found to be downregulated. The box and whisker plot on the left summarizes the distribution of all the differentially expressed genes targeted by this miRNA. The box represents the 1st quartile, the median and the 3rd quartile, while the outliers are represented by circles.*

## hsa-miR-122-5p (MIMAT0000421)

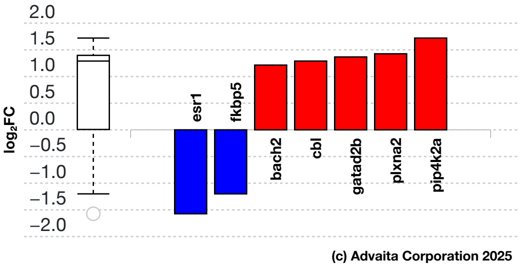


**Fig. 4.2.2: Gene measured expression bar plot:** *All the differentially expressed genes that are targeted by hsa-miR-122-5p are ranked based on their measured expression change (most downregulated to upregulated). The downregulated genes are shown in blue, and the upregulated ones are shown in red (where applicable). Out of all the differentially expressed target genes, 2 were found to be downregulated. The box and whisker plot on the left summarizes the distribution of all the differentially expressed genes targeted by this miRNA. The box represents the 1st quartile, the median and the 3rd quartile, while the outliers are represented by circles.*

## hsa-miR-100-5p (MIMAT0000098)

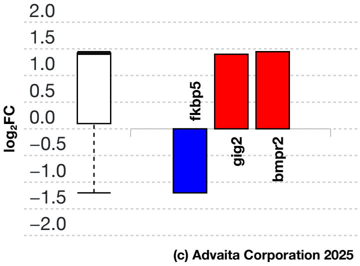


**Fig. 4.2.3: Gene measured expression bar plot:** *All the differentially expressed genes that are targeted by hsa-miR-100-5p are ranked based on their measured expression change (most downregulated to upregulated). The downregulated genes are shown in blue, and the upregulated ones are shown in red (where applicable). Out of all the differentially expressed target genes, 1 were found to be downregulated. The box and whisker plot on the left summarizes the distribution of all the differentially expressed genes targeted by this miRNA. The box represents the 1st quartile, the median and the 3rd quartile, while the outliers are represented by circles.*

## hsa-miR-99a-5p (MIMAT0000097)

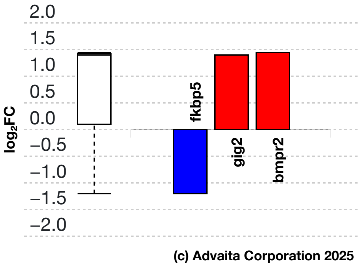


**Fig. 4.2.4: Gene measured expression bar plot:** *All the differentially expressed genes that are targeted by hsa-miR-99a-5p are ranked based on their measured expression change (most downregulated to upregulated). The downregulated genes are shown in blue, and the upregulated ones are shown in red (where applicable). Out of all the differentially expressed target genes, 1 were found to be downregulated. The box and whisker plot on the left summarizes the distribution of all the differentially expressed genes targeted by this miRNA. The box represents the 1st quartile, the median and the 3rd quartile, while the outliers are represented by circles.*

## hsa-miR-99b-5p (MIMAT0000689)

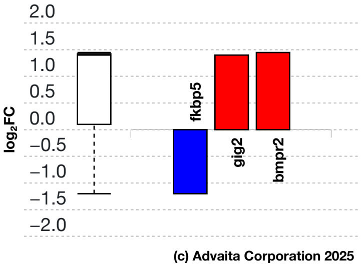


**Fig. 4.2.5: Gene measured expression bar plot:** *All the differentially expressed genes that are targeted by hsa-miR-99b-5p are ranked based on their measured expression change (most downregulated to upregulated). The downregulated genes are shown in blue, and the upregulated ones are shown in red (where applicable). Out of all the differentially expressed target genes, 1 were found to be downregulated. The box and whisker plot on the left summarizes the distribution of all the differentially expressed genes targeted by this miRNA. The box represents the 1st quartile, the median and the 3rd quartile, while the outliers are represented by circles.*

# 5. Predicted Upstream Regulator Analysis - Genes

## 5.1. Methods

The prediction of upstream regulators is based on two types of information: i) the enrichment of differentially expressed genes from the experiment and ii) a network of regulatory interactions from our proprietary knowledge base (see the report information for details). The network is a directed graph in which the nodes represent genes, and the edges represent regulatory interactions between two genes. A signed edge in this graph consists of a source gene, a target gene, and a sign to indicate the type of signal: activation (+) or inhibition (-). To create the network, the analysis selects only those edges observed in the literature with at least a medium confidence (evidence score greater than or equal to 400). The analysis considers two hypotheses:

HA. The upstream regulator is **activated** in the condition studied.
HI. The upstream regulator is **inhibited** in the condition studied.

The analysis divides the set of all the genes obtained from NCBI Gene database into several subsets based on the measurements in the experiment and the definitions shown in **Figure 5.1.1** and **Figure 5.1.2**. Let the sign of a measured DE gene be the sign of the log fold change value: (+) for up-regulated genes and (-) for down-regulated genes. A gene is a target gene if it corresponds to a node in the network that has at least one incoming edge. We define a *consistent gene* as a target DE gene such that the sign of the gene is consistent both with the type of the signal **and** with the hypothesis considered. Formally, by definition, a target DE gene *g* is consistent with Hypothesis HA if and only if an incoming edge *e* exists such that *sign(g) = sign(e)*. In other words, this describes the situation when the upstream regulator is predicted as activated, the signal is activation and the target DE gene is up-regulated, or the signal is inhibition and the target DE gene is down-regulated (see panel A in **Figure 5.1.1**). A target DE gene *g* is consistent with Hypothesis HI if and only if an incoming edge *e* exists such that *sign(g) ≠ sign(e)*. This second case captures the situation in which the upstream regulator is inhibited, the signal is inhibition and the target DE gene is up-regulated, or the signal is activation and the target DE gene is down-regulated (see panel B in **Figure 5.1.1**).

**Fig. 5.1.1: Target genes consistent with the hypothesis considered:** *In panel A, the signs of the DE genes match the signs of their respective incoming edges, increasing the likelihood that the upstream regulator u is activated. In panel B, the signs of the DE genes are opposite to the signs of their edges, increasing the likelihood that the upstream regulator u is inhibited.*

**Fig. 5.1.2:** *The set of all genes includes the set of measured genes that are also targets in the network, or Measured Targets (MT). We define the subset of "DE Targets consistent with the first hypothesis that the upstream regulators are Activated", DTA. For a selected upstream regulator u, we have the set of "Measured Targets of u" MT(u), "Differentially expressed Targets downstream of u" DT(u), and the set of "DE targets consistent with the hypothesis HA that u is Activated" DTA(u). The equivalent graphic for the hypothesis HI associated with DTI and DTI(u) is not shown.*

## Upstream regulators Z-score

For both research hypotheses, the analysis computes a Z-score for each upstream regulator *z(u)* by iterating over the genes in DT(u) and their incoming edges *in(g)*. We can then compute the p-value corresponding to the z-score $P_z$ as the one-tailed area under the probability density function for a normal distribution, N(0,1).

## Upstream regulators predicted as activated

Here, the research hypothesis considers the upstream regulator as activated. For each upstream regulator *u*, the number of consistent DE genes downstream of *u*, DTA(u) is compared to the number of measured target genes expected to be both consistent and DE just by chance. iPathwayGuide uses an over-representation approach to compute the statistical significance of observing at least the given number of consistent DE genes. The p-value $P_{act}$ is computed using the hypergeometric distribution (Draghici *et al*., 2003, Draghici 2011).

After computing a p-value for both types of evidence, $P_z$ and $P_{act}$, we need to combine these two probabilities into one global probability value, $P_G$ that is used to rank the upstream regulators and test the research hypothesis that the upstream regulators are predicted as activated in the condition studied. Since only a positive z-score indicates that the upstream regulator is predicted as activated, we only combine p-values for a positive z-score. Moreover, to avoid introducing false positives, only $P_z$ for significant z-scores ( $z \geq 2$ ) are combined. The analysis uses the standard Fisher's method to combine p-values into one test statistic (Fisher 1925).

## Upstream regulators predicted as inhibited

In parallel with upstream regulators predicted as activated, we use $P_{inh}$ and $P_z$ to predict upstream regulators that are inhibited. Here, the research hypothesis states that the upstream regulators are inhibited in the conditions studied. For each upstream regulator *u*, the number of consistent DE genes downstream of *u*, DTI(u) is compared to the number of measured target genes expected to be both consistent and DE just by chance. Using the Fisher's method as above, the analysis combines $P_{inh}$ and $P_z$, where $P_z$ is considered only for significant negative z-scores ( $z \leq -2$).

# 5.2. Results: upstream regulators predicted as activated

| Upstream Regulator (u) | DTA(u) | DT(u) | p-value | p-value (FDR) | p-value (Bonferroni) |
|---|---|---|---|---|---|
| NRG2 | 2 | 2 | 0.001 | 0.242 | 0.484 |
| NRG3 | 2 | 2 | 0.001 | 0.242 | 0.484 |
| GNRH1 | 3 | 3 | 0.004 | 0.358 | 1.000 |
| CREB1 | 4 | 4 | 0.005 | 0.358 | 1.000 |
| UNC13B | 2 | 2 | 0.020 | 0.358 | 1.000 |

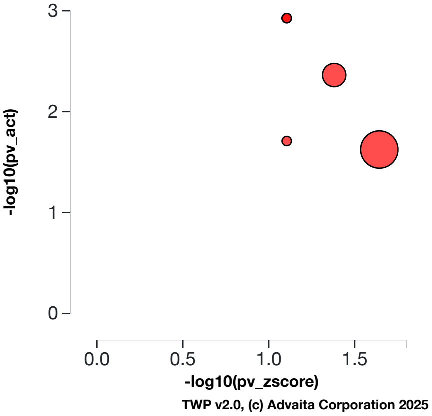


**Table 5.2.1: Top upstream regulators predicted as activated.** *For each upstream regulator u, the table shows the number of DE targets supporting the hypothesis that the regulator is activated DTA(u) the total number of DE genes downstream of u DT(u), the combined raw p-value, and the p-value corrected for multiple comparisons.* **Fig. 5.2.1: A two-way plot showing the top five upstream regulators predicted as activated.** *Dots representing upstream regulators are positioned using $P_{zscore}$ on the horizontal axis, and using $P_{act}$ on the vertical axis. $P_{act}$ is the p-value based on the number of DE targets consistent with the typeof the incoming signal and with the selected hypothesis type. Upstream regulators with a significant combined p-value are shown in red. The size of each dot represents the number of consistent DE genes for that regulator.*

## NRG2 (neuregulin 2)

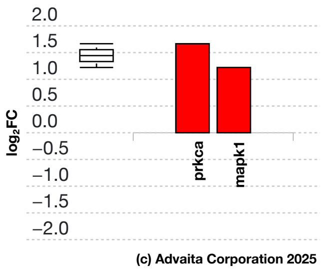


**Fig. 5.2.3: Gene measured expression bar plot:** *All the consistent differentially expressed genes that are targeted by NRG2 are ranked based on their absolute value of log fold change. Upregulated genes are shown in red, downregulated genes are shown in blue. The box and whisker plot on the left summarizes the distribution of all the consistent differentially expressed genes targeted by this upstream regulator. The box shows the 1st quartile, the median and the 3rd quartile, while the outliers are represented by circles.*

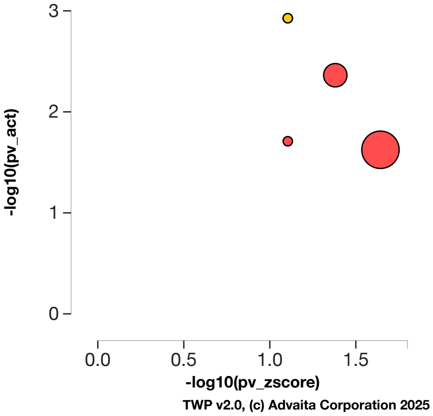


**Fig. 5.2.4: Activation p-value vs zscore p-value:** *NRG2, neuregulin 2, (yellow) is shown, using negative log of the activation and zscore p-values, along with the other most significant upstream regulators. The size of the dot represents the relative number of consistent DE genes, which for selected upstream regulator is 2.*

## NRG3 (neuregulin 3)

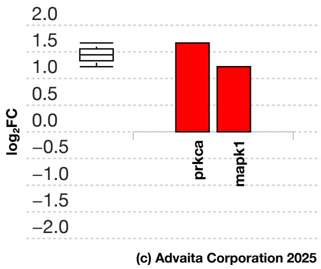


**Fig. 5.2.5: Gene measured expression bar plot:** *All the consistent differentially expressed genes that are targeted by NRG3 are ranked based on their absolute value of log fold change. Upregulated genes are shown in red, downregulated genes are shown in blue. The box and whisker plot on the left summarizes the distribution of all the consistent differentially expressed genes targeted by this upstream regulator. The box shows the 1st quartile, the median and the 3rd quartile, while the outliers are represented by circles.*

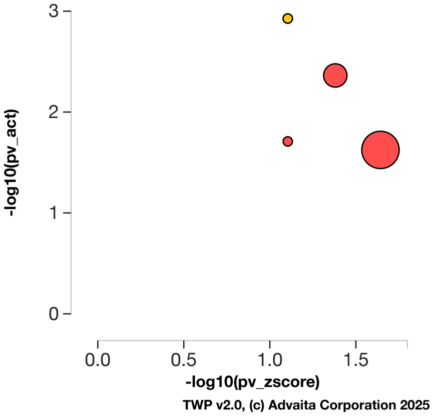


**Fig. 5.2.6: Activation p-value vs zscore p-value:** *NRG3, neuregulin 3, (yellow) is shown, using negative log of the activation and zscore p-values, along with the other most significant upstream regulators. The size of the dot represents the relative number of consistent DE genes, which for selected upstream regulator is 2.*

## GNRH1 (gonadotropin releasing hormone 1)

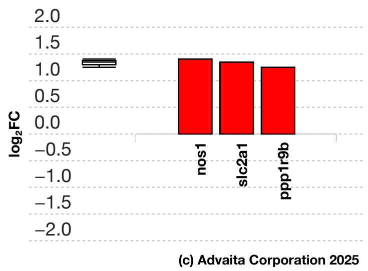


**Fig. 5.2.7: Gene measured expression bar plot:** *All the consistent differentially expressed genes that are targeted by GNRH1 are ranked based on their absolute value of log fold change. Upregulated genes are shown in red, downregulated genes are shown in blue. The box and whisker plot on the left summarizes the distribution of all the consistent differentially expressed genes targeted by this upstream regulator. The box shows the 1st quartile, the median and the 3rd quartile, while the outliers are represented by circles.*

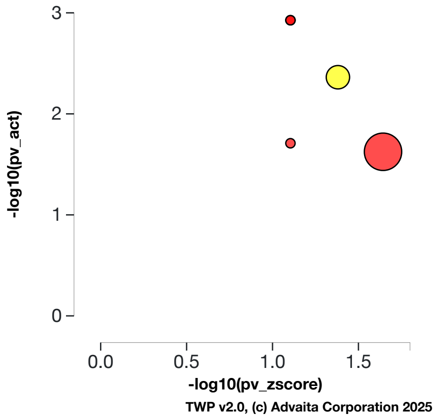


**Fig. 5.2.8: Activation p-value vs zscore p-value:** *GNRH1, gonadotropin releasing hormone 1, (yellow) is shown, using negative log of the activation and zscore p-values, along with the other most significant upstream regulators. The size of the dot represents the relative number of consistent DE genes, which for selected upstream regulator is 3.*

## CREB1 (cAMP responsive element binding protein 1)

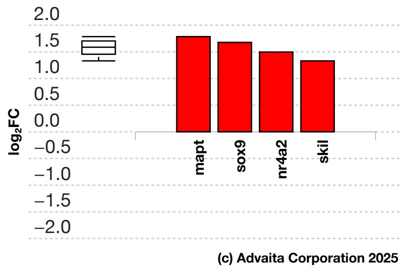


**Fig. 5.2.9: Gene measured expression bar plot:** *All the consistent differentially expressed genes that are targeted by CREB1 are ranked based on their absolute value of log fold change. Upregulated genes are shown in red, downregulated genes are shown in blue. The box and whisker plot on the left summarizes the distribution of all the consistent differentially expressed genes targeted by this upstream regulator. The box shows the 1st quartile, the median and the 3rd quartile, while the outliers are represented by circles.*

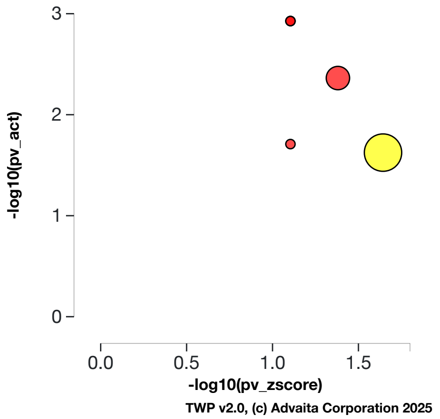


**Fig. 5.2.10: Activation p-value vs zscore p-value:** *CREB1, cAMP responsive element binding protein 1, (yellow) is shown, using negative log of the activation and zscore p-values, along with the other most significant upstream regulators. The size of the dot represents the relative number of consistent DE genes, which for selected upstream regulator is 4.*

## UNC13B (unc-13 homolog B)

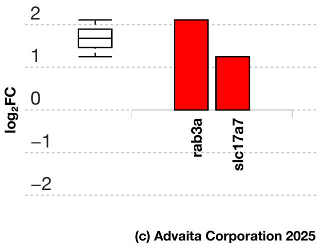


**Fig. 5.2.11: Gene measured expression bar plot:** *All the consistent differentially expressed genes that are targeted by UNC13B are ranked based on their absolute value of log fold change. Upregulated genes are shown in red, downregulated genes are shown in blue. The box and whisker plot on the left summarizes the distribution of all the consistent differentially expressed genes targeted by this upstream regulator. The box shows the 1st quartile, the median and the 3rd quartile, while the outliers are represented by circles.*

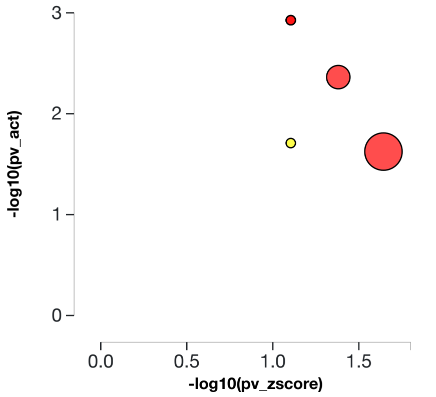


**Fig. 5.2.12: Activation p-value vs zscore p-value:** *UNC13B, unc-13 homolog B, (yellow) is shown, using negative log of the activation and zscore p-values, along with the other most significant upstream regulators. The size of the dot represents the relative number of consistent DE genes, which for selected upstream regulator is 2.*

# 5.3. Results: upstream regulators predicted as inhibited

| Upstream Regulator (u) | DTI(u) | DT(u) | p-value | p-value (FDR) | p-value (Bonferroni) |
|---|---|---|---|---|---|
| DDX41 | 2 | 2 | 0.003 | 0.437 | 1.000 |
| NLK | 2 | 2 | 0.012 | 0.437 | 1.000 |
| HMGN1 | 1 | 1 | 0.016 | 0.437 | 1.000 |
| LGALS8 | 1 | 1 | 0.016 | 0.437 | 1.000 |
| PKD1 | 1 | 1 | 0.016 | 0.437 | 1.000 |

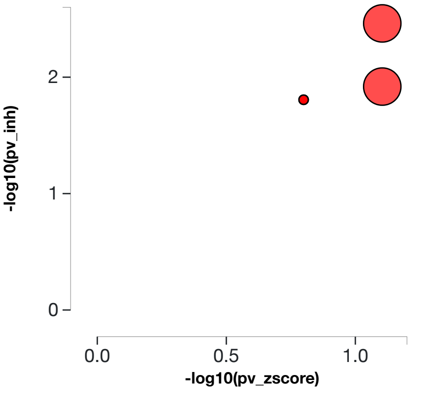


**Table 5.3.1: Top upstream regulators predicted as inhibited.** *For each upstream regulator u, the table shows the number of DE targets supporting the hypothesis that the regulator is inhibited DTI(u) the total number of DE genes downstream of u DT(u), the combined raw p-value, and the p-value corrected for multiple comparisons.* **Fig. 5.3.1: A two-way plot showing the top five upstream regulators predicted as inhibited.** *Dots representing upstream regulators are positioned using $P_{zscore}$ on the horizontal axis, and using $P_{inh}$ on the vertical axis. $P_{inh}$ is the p-value based on the number of DE targets consistent with the typeof the incoming signal and with the selected hypothesis type. Upstream regulators with a significant combined p-value are shown in red. The size of each dot represents the number of consistent DE genes for that regulator.*

## DDX41 (DEAD-box helicase 41)

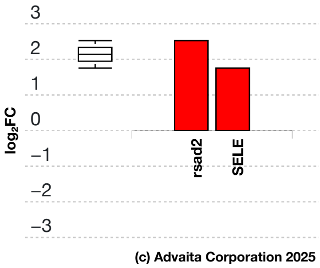


**Fig. 5.3.13: Gene measured expression bar plot:** *All the consistent differentially expressed genes that are targeted by DDX41 are ranked based on their absolute value of log fold change. Upregulated genes are shown in red, downregulated genes are shown in blue. The box and whisker plot on the left summarizes the distribution of all the consistent differentially expressed genes targeted by this upstream regulator. The box shows the 1st quartile, the median and the 3rd quartile, while the outliers are represented by circles.*

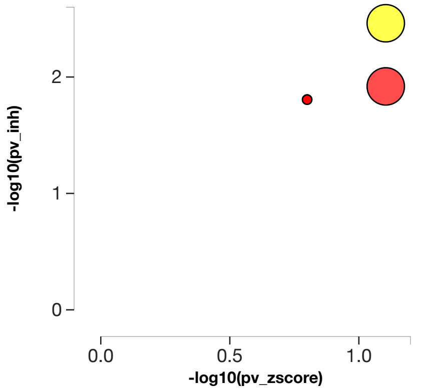


TWP v2.0, (c) Advaita Corporation 2025

**Fig. 5.3.14: Inhibition p-value vs zscore p-value:** *DDX41, DEAD-box helicase 41, (yellow) is shown, using negative log of the inhibition and zscore p-values, along with the other most significant upstream regulators. The size of the dot represents the relative number of consistent DE genes, which for selected upstream regulator is 2.*

## NLK (nemo like kinase)

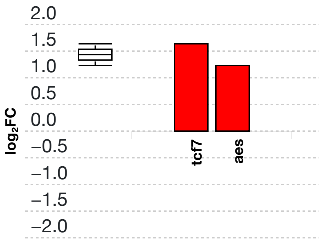


(c) Advaita Corporation 2025

**Fig. 5.3.15: Gene measured expression bar plot:** *All the consistent differentially expressed genes that are targeted by NLK are ranked based on their absolute value of log fold change. Upregulated genes are shown in red, downregulated genes are shown in blue. The box and whisker plot on the left summarizes the distribution of all the consistent differentially expressed genes targeted by this upstream regulator. The box shows the 1st quartile, the median and the 3rd quartile, while the outliers are represented by circles.*

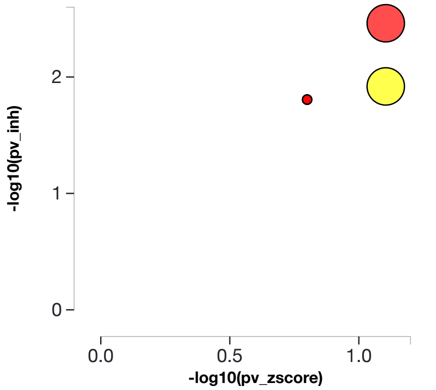


TWP v2.0, (c) Advaita Corporation 2025

**Fig. 5.3.16: Inhibition p-value vs zscore p-value:** *NLK, nemo like kinase, (yellow) is shown, using negative log of the inhibition and zscore p-values, along with the other most significant upstream regulators. The size of the dot represents the relative number of consistent DE genes, which for selected upstream regulator is 2.*

## HMGN1 (high mobility group nucleosome binding domain 1)

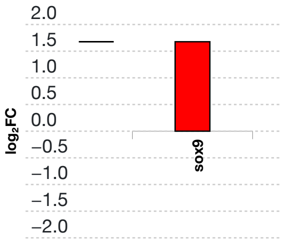


(c) Advaita Corporation 2025

**Fig. 5.3.17: Gene measured expression bar plot:** *All the consistent differentially expressed genes that are targeted by HMGN1 are ranked based on their absolute value of log fold change. Upregulated genes are shown in red, downregulated genes are shown in blue. The box and whisker plot on the left summarizes the distribution of all the consistent differentially expressed genes targeted by this upstream regulator. The box shows the 1st quartile, the median and the 3rd quartile, while the outliers are represented by circles.*

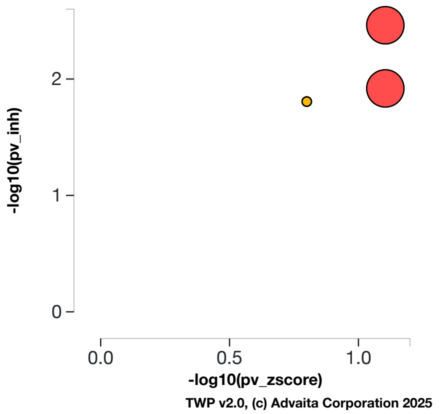


**Fig. 5.3.18: Inhibition p-value vs zscore p-value:** *HMGN1, high mobility group nucleosome binding domain 1, (yellow) is shown, using negative log of the inhibition and zscore p-values, along with the other most significant upstream regulators. The size of the dot represents the relative number of consistent DE genes, which for selected upstream regulator is 1.*

## LGALS8 (galectin 8)

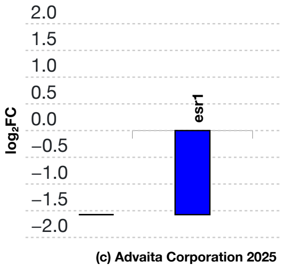


**Fig. 5.3.19: Gene measured expression bar plot:** *All the consistent differentially expressed genes that are targeted by LGALS8 are ranked based on their absolute value of log fold change. Upregulated genes are shown in red, downregulated genes are shown in blue. The box and whisker plot on the left summarizes the distribution of all the consistent differentially expressed genes targeted by this upstream regulator. The box shows the 1st quartile, the median and the 3rd quartile, while the outliers are represented by circles.*

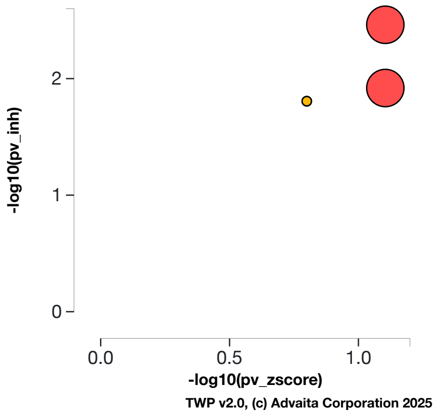


**Fig. 5.3.20: Inhibition p-value vs zscore p-value:** *LGALS8, galectin 8, (yellow) is shown, using negative log of the inhibition and zscore p-values, along with the other most significant upstream regulators. The size of the dot represents the relative number of consistent DE genes, which for selected upstream regulator is 1.*

## PKD1 (polycystin 1, transient receptor potential channel interacting)

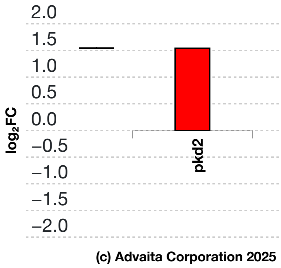


**Fig. 5.3.21: Gene measured expression bar plot:** *All the consistent differentially expressed genes that are targeted by PKD1 are ranked based on their absolute value of log fold change. Upregulated genes are shown in red, downregulated genes are shown in blue. The box and whisker plot on the left summarizes the distribution of all the consistent differentially expressed genes targeted by this upstream regulator. The box shows the 1st quartile, the median and the 3rd quartile, while the outliers are represented by circles.*

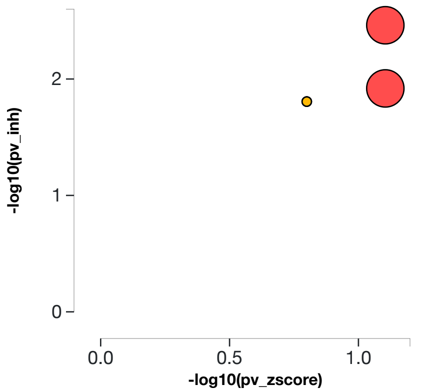


**Fig. 5.3.22: Inhibition p-value vs zscore p-value:** *PKD1, polycystin 1, transient receptor potential channel interacting, (yellow) is shown, using negative log of the inhibition and zscore p-values, along with the other most significant upstream regulators. The size of the dot represents the relative number of consistent DE genes, which for selected upstream regulator is 1.*

# 6. Predicted Upstream Regulator Analysis – Chemicals, Drugs, Toxicants (CDTs)

## 6.1. Methods

The prediction of upstream Chemicals, Drugs, Toxicants (CDTs) is based on two types of information: i) the enrichment of differentially expressed genes from the experiment and ii) a network of interactions from the Advaita Knowledge Base (AKB v18.1). The network is a directed graph in which the source node represents either a chemical substance or compound (e.g. zinc), a drug (e.g. aspirin), or a toxicant (e.g. tobacco smoke). The generic abbreviation CDT will be used henceforth to designate any of these. The edges represent known effects that these CDTs have on various genes. A signed edge in this graph consists of a source CDT, a target gene, and a sign to indicate the type of effect: activation (+) or inhibition (-). The analysis considers two hypotheses:

HP. The upstream chemical, drug or toxicant is **present (or overly abundant)** in the condition studied.
HA. The upstream chemical, drug or toxicant is **absent (or insufficient)** in the condition studied.

The analysis divides the set of all the genes from AKB into several subsets based on the measurements in the experiment and the definitions shown in **Figure 6.1.1** and **Figure 6.1.2**. Let the sign of a measured DE gene be the sign of the log fold change value: (+) for up-regulated genes and (-) for down-regulated genes. A gene is a target gene if it corresponds to a node in the network that has at least one incoming edge. We define a *consistent gene* as a target DE gene such that the sign of the gene is consistent both with the type of the signal **and** with the hypothesis considered. Formally, by definition, a target DE gene *g* is consistent with Hypothesis HP if and only if an incoming edge *e* exists such that *sign(g) = sign(e)*. In other words, this describes the situation when the CDT is predicted as present, the signal is activation and the target DE gene is up-regulated, or the signal is inhibition and the target DE gene is down-regulated (see panel A in **Figure 6.1.1**). A target DE gene *g* is consistent with Hypothesis HA if and only if an incoming edge *e* exists such that *sign(g) ≠ sign(e)*. This second case captures the situation in which the CDT is absent (or insufficient), the signal is inhibition and the target DE gene is up-regulated, or the signal is activation and the target DE gene is down-regulated (see panel B in **Figure 6.1.1**).

**Fig. 6.1.1: Target genes consistent with the hypothesis considered:** *In panel A, the signs of the DE genes match the signs of their respective incoming edges, increasing the likelihood that the CDT u is present. In panel B, the signs of the DE genes are opposite to the signs of their edges, increasing the likelihood that the CDT u is absent.*

**Fig. 6.1.2:** *The set of all genes includes the set of measured genes that are also targets in the network, or Measured Targets (MT). We define the subset of "DE Targets consistent with the first hypothesis that the CDTs are Present (or overly abundant)", DTA. For a selected upstream CDT u, we have the set of "Measured Targets of u" MT(u), "Differentially*

*expressed Targets downstream of u" DT(u), and the set of "DE targets consistent with the hypothesis HP that u is Present" DTA(u). The equivalent graphic for the hypothesis HA associated with DTI and DTI(u) is not shown.*

### Z-score

For both research hypotheses, the analysis computes a Z-score for each CDT *z(u)* by iterating over the genes in DT(u) and their incoming edges *in(g)*. We can then compute the p-value corresponding to the z-score $P_z$ as the one-tailed area under the probability density function for a normal distribution, N(0,1).

### Upstream CDTs predicted as present (or overly abundant)

Here, the research hypothesis considers presence of the CDT. This hypothesis is useful when investigating whether the given phenotype has been impacted by the presence of a given chemical, drug or toxicant (e.g. tobacco smoke, dioxin, etc.). For each CDT *u*, the number of consistent DE genes downstream of *u*, DTA(u) is compared to the number of measured target genes expected to be both consistent and DE just by chance. iPathwayGuide uses an over-representation approach to compute the statistical significance of observing at least the given number of consistent DE genes. The p-value $P_{pres}$ is computed using the hypergeometric distribution (Draghici *et al*., 2003, Draghici 2011).

After computing a p-value for both types of evidence, $P_z$ and $P_{pres}$, we combine these two probabilities into one global probability value, $P_G$ that is used to rank the upstream regulators and test the research hypothesis that the upstream CDTs are predicted as present in the condition studied. The analysis uses the standard Fisher's method to combine p-values into one test statistic (Fisher 1925).

### Upstream CDTs predicted as absent (or insufficient)

In parallel with upstream CDTs predicted as present, we use $P_{abs}$ and $P_z$ to predict upstream CDTs that are absent. This hypothesis is relevant when investigating whether the given phenotype has been impacted by the lack of a given chemical that is necessary for the well-functioning of the organism or cell (e.g. a vitamin deficiency, iron deficiency, etc.). Here, the research hypothesis states that the upstream CDT are insufficient in the condition studied. For each upstream CDT *u*, the number of consistent DE genes downstream of *u*, DTI(u) is compared to the number of measured target genes expected to be both consistent and DE just by chance. Using the Fisher's method as above, the analysis combines $P_{abs}$ and $P_z$, where $P_z$ is considered only for significant negative z-scores ( $z \leq -2$).

## 6.2. Results: upstream CDTs predicted as present (or overly abundant)

| CDT (u) | DTA(u) | DT(u) | p-value | p-value (FDR) | p-value (Bonferroni) |
|---|---|---|---|---|---|
| Dietary Sucrose | 25 | 29 | **4.225e-6** | **0.004** | **0.007** |
| decamethrin | 134 | 174 | **3.896e-9** | **6.553e-6** | **6.553e-6** |
| Ethanol | 76 | 92 | **1.461e-5** | **0.006** | **0.025** |
| fenvalerate | 35 | 42 | **1.568e-5** | **0.006** | **0.026** |
| propionaldehyde | 34 | 40 | **1.804e-5** | **0.006** | **0.030** |

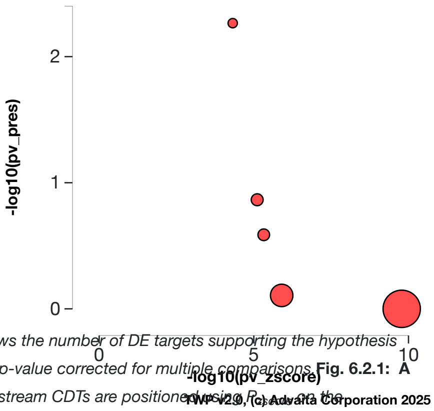


**Table 6.2.1: Top upstream CDTs predicted as present (or overly abundant).** *For each upstream CDT u, the table shows the number of DE targets supporting the hypothesis that the CDT is present DTA(u) the total number of DE genes downstream of u DT(u), the combined raw p-value, and the p-value corrected for multiple comparisons.* **Fig. 6.2.1: A two-way plot showing the top five upstream CDTs predicted as present (or overly abundant).** *Dots representing upstream CDTs are positioned using $P_z$ on the horizontal axis, and using $P_{pres}$ on the vertical axis. $P_{pres}$ is the p-value based on the number of DE targets consistent with the typeof the incoming signal and with the selected hypothesis type. Upstream CDTs with a significant combined p-value are shown in red. The size of each dot represents the relative number of consistent DE genes for that CDT.*

### Dietary Sucrose

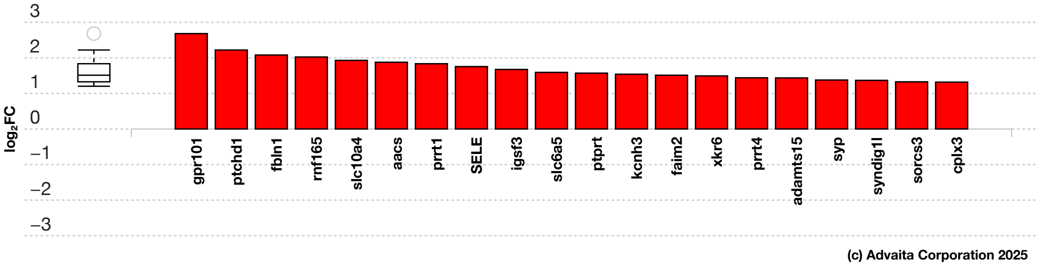


**Fig. 6.2.3: Consistent DE target genes measured expression bar plot:** *All the consistent differentially expressed genes that are targeted by Dietary Sucrose are ranked based on their absolute value of log fold change. The plot is limited to the top 20 genes out of a total of 25 consistent differentially expressed target genes. Upregulated genes are shown in red, downregulated genes are shown in blue. The box and whisker plot on the left summarizes the distribution of all the consistent differentially expressed genes targeted by this upstream regulator. The box shows the 1st quartile, the median and the 3rd quartile, while any outliers are represented by circles.*

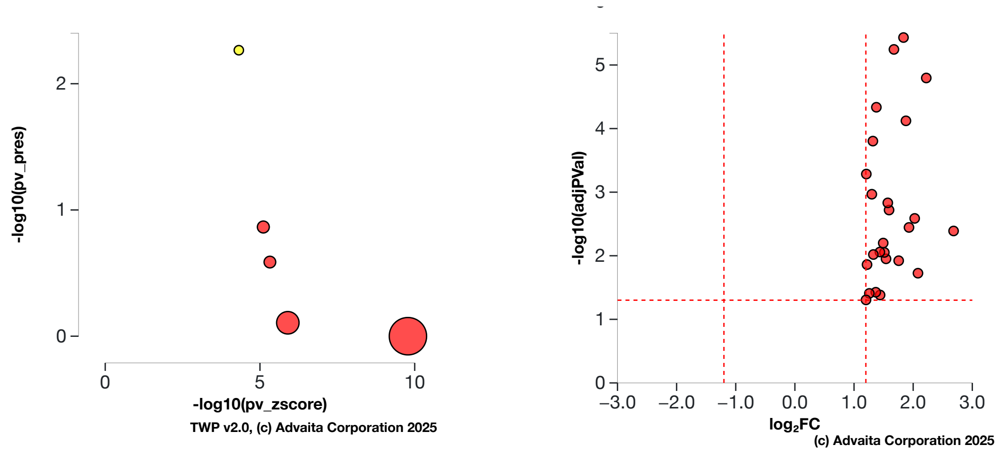


a) b)

**Fig. 6.2.4: a) Present (overly abundant) p-value vs zscore p-value:** *The significance of Dietary Sucrose is plotted on two axes, with negative log of $P_z$ on x-axis and negative log of $P_{pres}$ on y-axis. The size of the dot represents the relative number of consistent DE genes, which for selected upstream regulator is 25.* **b) Volcano plot:** *There are **25** DE genes that are targets of Dietary Sucrose consistent with the hypothesis that Dietary Sucrose is present (overly abundant) The target genes are represented in terms of their measured expression change (x-axis) and the significance of the change (y-axis). The significance is represented in terms of the negative log (base 10) of the p-value, so that more significant genes are plotted higher on the y-axis. The dotted lines represent the thresholds used to select the DE genes: **1.2** for expression change and **0.05** for significance.*

## decamethrin

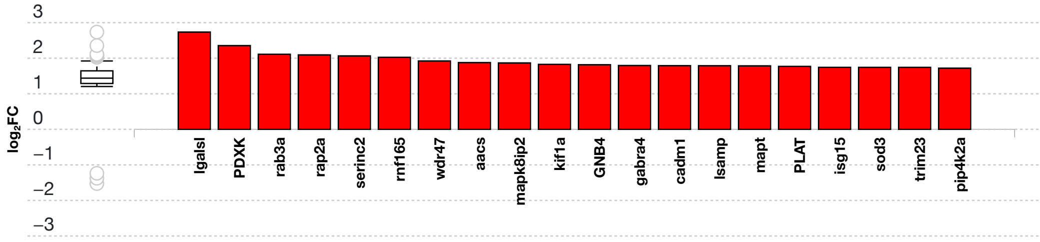


**Fig. 6.2.5: Consistent DE target genes measured expression bar plot:** *All the consistent differentially expressed genes that are targeted by decamethrin are ranked based on their absolute value of log fold change. The plot is limited to the top 20 genes out of a total of 134 consistent differentially expressed target genes. Upregulated genes are shown in red, downregulated genes are shown in blue. The box and whisker plot on the left summarizes the distribution of all the consistent differentially expressed genes targeted by this upstream regulator. The box shows the 1st quartile, the median and the 3rd quartile, while any outliers are represented by circles.*

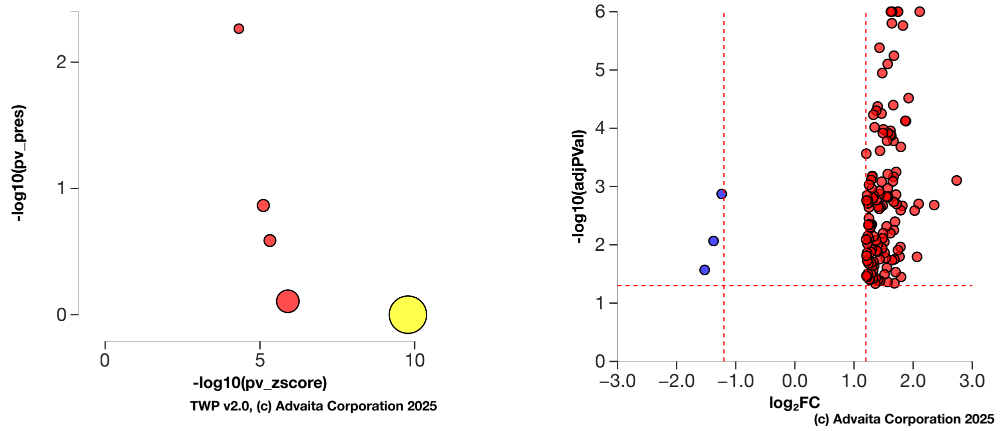


a) b)

**Fig. 6.2.6: a) Present (overly abundant) p-value vs zscore p-value:** *The significance of decamethrin is plotted on two axes, with negative log of $P_z$ on x-axis and negative log of $P_{pres}$ on y-axis. The size of the dot represents the relative number of consistent DE genes, which for selected upstream regulator is 134.* **b) Volcano plot:** *There are **134** DE genes that are targets of decamethrin consistent with the hypothesis that decamethrin is present (overly abundant) The target genes are represented in terms of their measured expression change (x-axis) and the significance of the change (y-axis). The significance is represented in terms of the negative log (base 10) of the p-value, so that more significant genes are plotted higher on the y-axis. The dotted lines represent the thresholds used to select the DE genes: **1.2** for expression change and **0.05** for significance.*

## Ethanol

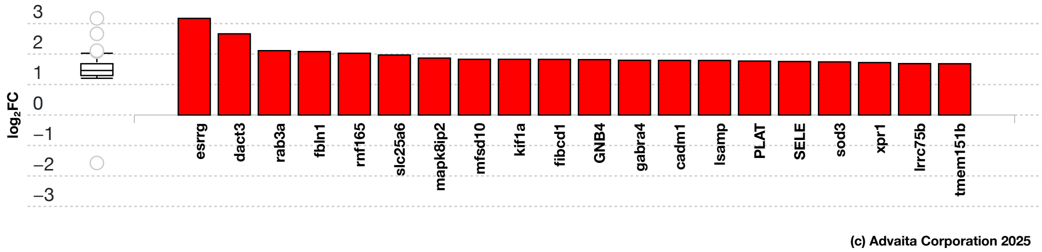


**Fig. 6.2.7: Consistent DE target genes measured expression bar plot:** *All the consistent differentially expressed genes that are targeted by Ethanol are ranked based on their absolute value of log fold change. The plot is limited to the top 20 genes out of a total of 76 consistent differentially expressed target genes. Upregulated genes are shown in red, downregulated genes are shown in blue. The box and whisker plot on the left summarizes the distribution of all the consistent differentially expressed genes targeted by this upstream regulator. The box shows the 1st quartile, the median and the 3rd quartile, while any outliers are represented by circles.*

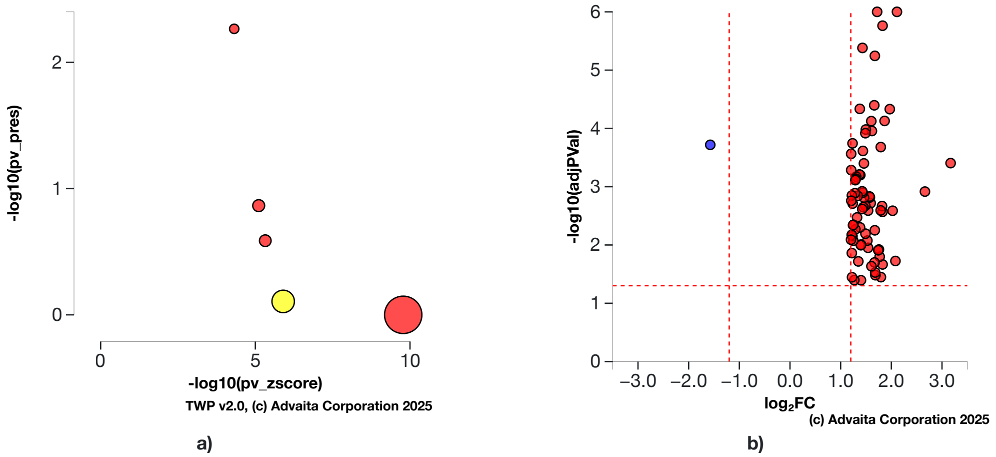


**Fig. 6.2.8: a) Present (overly abundant) p-value vs zscore p-value:** *The significance of Ethanol is plotted on two axes, with negative log of $P_z$ on x-axis and negative log of $P_{pres}$ on y-axis. The size of the dot represents the relative number of consistent DE genes, which for selected upstream regulator is 76.* **b) Volcano plot:** *There are* ***76*** *DE genes that are targets of Ethanol consistent with the hypothesis that Ethanol is present (overly abundant) The target genes are represented in terms of their measured expression change (x-axis) and the significance of the change (y-axis). The significance is represented in terms of the negative log (base 10) of the p-value, so that more significant genes are plotted higher on the y-axis. The dotted lines represent the thresholds used to select the DE genes:* ***1.2*** *for expression change and* ***0.05*** *for significance.*

## fenvalerate

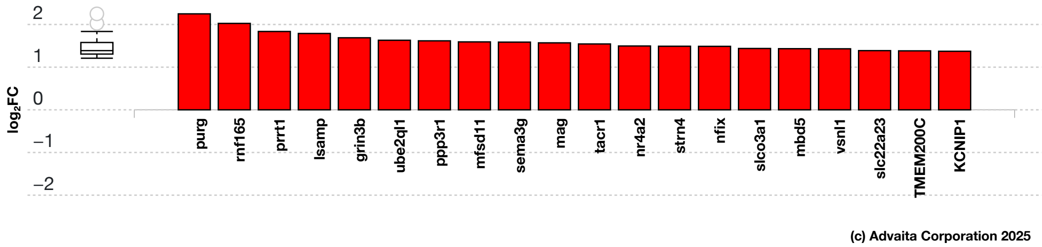


**Fig. 6.2.9: Consistent DE target genes measured expression bar plot:** *All the consistent differentially expressed genes that are targeted by fenvalerate are ranked based on their absolute value of log fold change. The plot is limited to the top 20 genes out of a total of 35 consistent differentially expressed target genes. Upregulated genes are shown in red, downregulated genes are shown in blue. The box and whisker plot on the left summarizes the distribution of all the consistent differentially expressed genes targeted by this upstream regulator. The box shows the 1st quartile, the median and the 3rd quartile, while any outliers are represented by circles.*

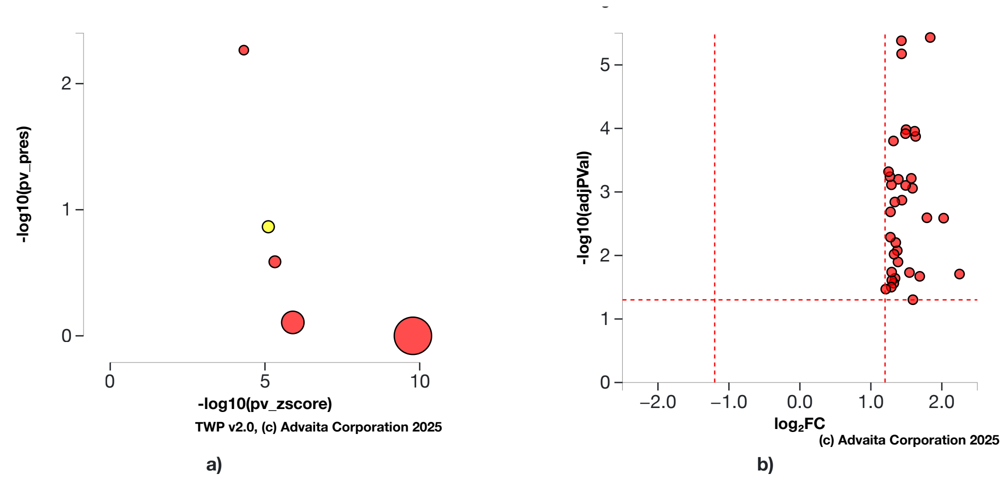


**Fig. 6.2.10: a) Present (overly abundant) p-value vs zscore p-value:** *The significance of fenvalerate is plotted on two axes, with negative log of $P_z$ on x-axis and negative log of $P_{pres}$ on y-axis. The size of the dot represents the relative number of consistent DE genes, which for selected upstream regulator is 35.* **b) Volcano plot:** *There are* ***35*** *DE genes that are targets of fenvalerate consistent with the hypothesis that fenvalerate is present (overly abundant) The target genes are represented in terms of their measured expression change (x-axis) and the significance of the change (y-axis). The significance is represented in terms of the negative log (base 10) of the p-value, so that more significant genes are plotted higher on the y-axis. The dotted lines represent the thresholds used to select the DE genes:* ***1.2*** *for expression change and* ***0.05*** *for significance.*

## propionaldehyde

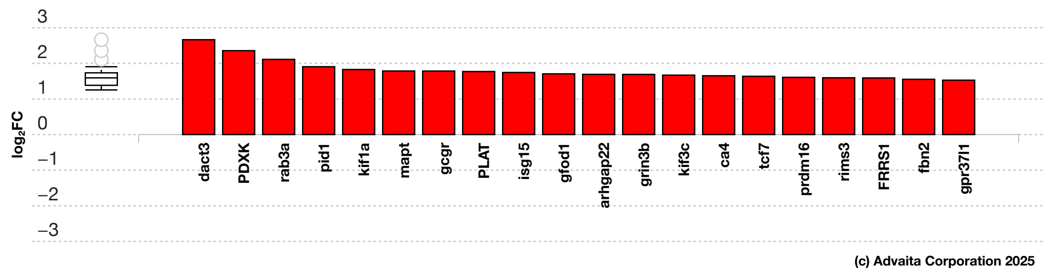


**Fig. 6.2.11: Consistent DE target genes measured expression bar plot:** *All the consistent differentially expressed genes that are targeted by propionaldehyde are ranked based on their absolute value of log fold change. The plot is limited to the top 20 genes out of a total of 34 consistent differentially expressed target genes. Upregulated genes are shown in red, downregulated genes are shown in blue. The box and whisker plot on the left summarizes the distribution of all the consistent differentially expressed genes targeted by this upstream regulator. The box shows the 1st quartile, the median and the 3rd quartile, while any outliers are represented by circles.*

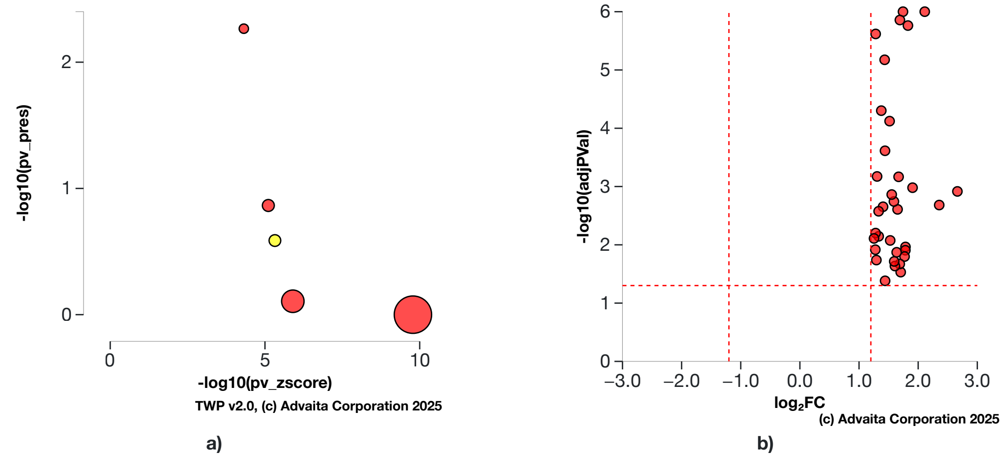


**Fig. 6.2.12: a) Present (overly abundant) p-value vs zscore p-value:** *The significance of propionaldehyde is plotted on two axes, with negative log of $P_z$ on x-axis and negative log of $P_{pres}$ on y-axis. The size of the dot represents the relative number of consistent DE genes, which for selected upstream regulator is 34.* **b) Volcano plot:** *There are* ***34*** *DE genes that are targets of propionaldehyde consistent with the hypothesis that propionaldehyde is present (overly abundant) The target genes are represented in terms of their measured expression change (x-axis) and the significance of the change (y-axis). The significance is represented in terms of the negative log (base 10) of the p-value, so that more significant genes are plotted higher on the y-axis. The dotted lines represent the thresholds used to select the DE genes:* ***1.2*** *for expression change and* ***0.05*** *for significance.*

## 6.3. Results: upstream CDTs predicted as absent (or insufficient)

| CDT (u) | DTI(u) | DT(u) | p-value | p-value (FDR) | p-value (Bonferroni) |
|---|---|---|---|---|---|
| Ivermectin | 62 | 69 | **4.587e-10** | **3.858e-7** | **7.715e-7** |
| 2,3,7,8-tetrachlorodibenzofuran | 50 | 59 | **8.035e-8** | **4.505e-5** | **1.351e-4** |
| Doxorubicin | 122 | 142 | **6.184e-12** | **1.040e-8** | **1.040e-8** |
| 2',3,3',4',5-pentachloro-4-hydroxybiphenyl | 47 | 53 | **1.532e-7** | **6.269e-5** | **2.577e-4** |
| Smoke | 87 | 112 | **1.864e-7** | **6.269e-5** | **3.135e-4** |

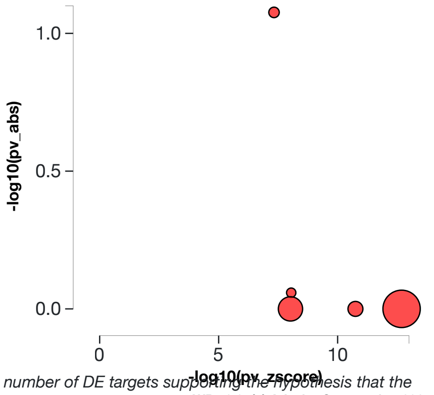


**Table 6.3.1: Top upstream CDTs predicted as absent (or insufficient).** *For each upstream CDT u, the table shows the number of DE targets supporting the hypothesis that the CDT is absent DTI(u) the total number of DE genes downstream of u DT(u), the combined raw p-value, and the p-value corrected for multiple comparisons.* **Fig. 6.3.1: A two-way plot showing the top five upstream CDTs predicted as absent (or insufficient).** *Dots representing upstream CDTs are positioned using $P_{zscore}$ on the horizontal axis, and using $P_{abs}$ on the vertical axis. $P_{abs}$ is the p-value based on the number of DE targets consistent with the typeof the incoming signal and with the selected hypothesis type. Upstream CDTs with a significant combined p-value are shown in red. The size of each dot represents the relative number of consistent DE genes for that CDT.*

### Ivermectin

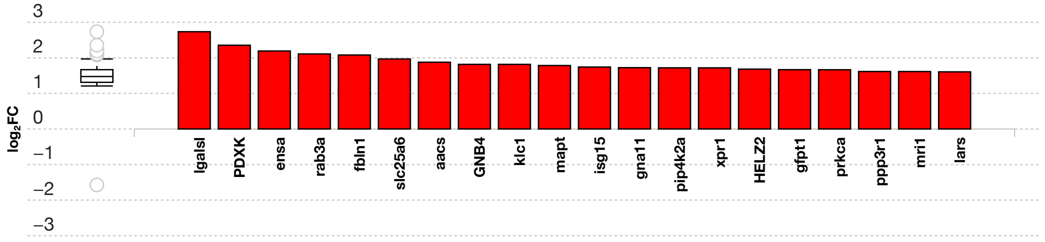


**Fig. 6.3.13: Consistent DE target genes measured expression bar plot:** *All the consistent differentially expressed genes that are targeted by Ivermectin are ranked based on their absolute value of log fold change. The plot is limited to the top 20 genes out of a total of 62 consistent differentially expressed target genes. Upregulated genes are shown in red, downregulated genes are shown in blue. The box and whisker plot on the left summarizes the distribution of all the consistent differentially expressed genes targeted by this upstream regulator. The box shows the 1st quartile, the median and the 3rd quartile, while any outliers are represented by circles.*

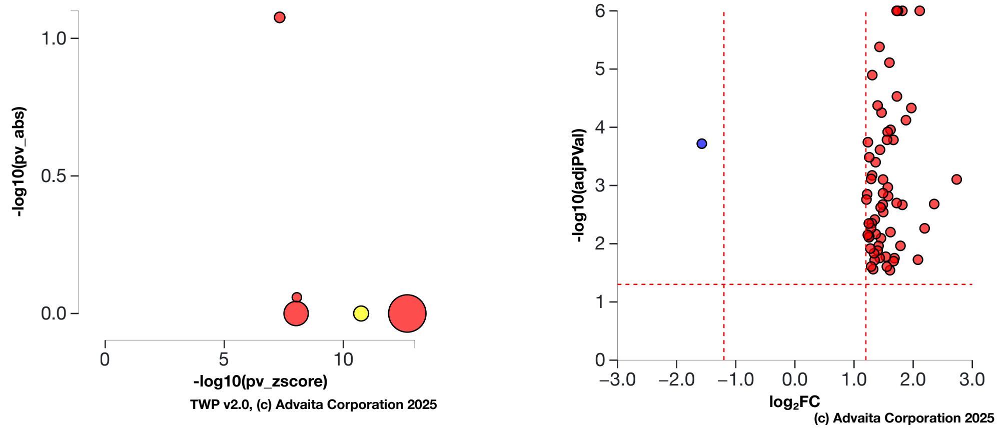


**Fig. 6.3.14: a) Absent (or insufficient) p-value vs zscore p-value:** *The significance of Ivermectin is plotted on two axes, with negative log of $P_z$ on x-axis and negative log of $P_{abs}$ on y-axis. The size of the dot represents the relative number of consistent DE genes, which for selected upstream regulator is 62.* **b) Volcano plot:** *There are* ***62*** *DE genes that are targets of Ivermectin consistent with the hypothesis that Ivermectin is absent (or insufficient) The target genes are represented in terms of their measured expression change (x-axis) and the significance of the change (y-axis). The significance is represented in terms of the negative log (base 10) of the p-value, so that more significant genes are plotted higher on the y-axis. The dotted lines represent the thresholds used to select the DE genes:* ***1.2*** *for expression change and* ***0.05*** *for significance.*

## 2,3,7,8-tetrachlorodibenzofuran

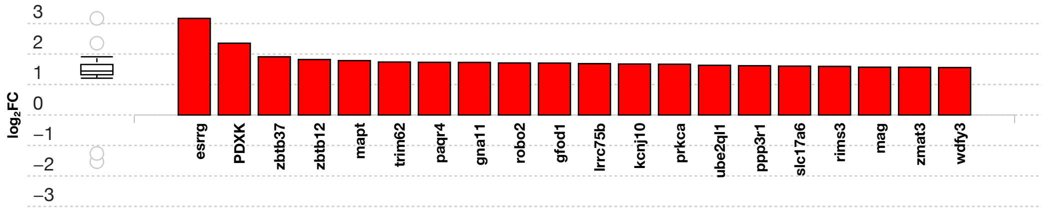


**Fig. 6.3.15: Consistent DE target genes measured expression bar plot:** *All the consistent differentially expressed genes that are targeted by 2,3,7,8-tetrachlorodibenzofuran are ranked based on their absolute value of log fold change. The plot is limited to the top 20 genes out of a total of 50 consistent differentially expressed target genes. Upregulated genes are shown in red, downregulated genes are shown in blue. The box and whisker plot on the left summarizes the distribution of all the consistent differentially expressed genes targeted by this upstream regulator. The box shows the 1st quartile, the median and the 3rd quartile, while any outliers are represented by circles.*

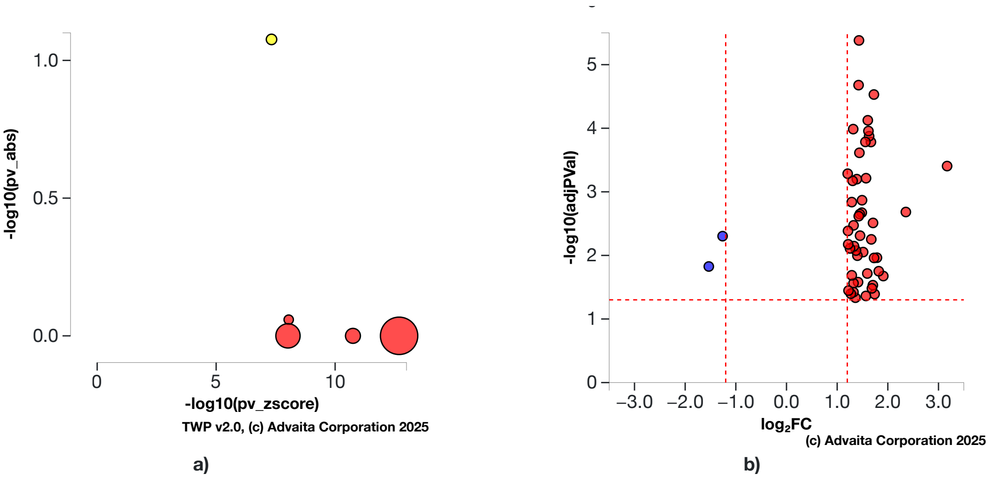


**Fig. 6.3.16: a) Absent (or insufficient) p-value vs zscore p-value:** *The significance of 2,3,7,8-tetrachlorodibenzofuran is plotted on two axes, with negative log of $P_z$ on x-axis and negative log of $P_{abs}$ on y-axis. The size of the dot represents the relative number of consistent DE genes, which for selected upstream regulator is 50.* **b) Volcano plot:** *There are **50** DE genes that are targets of 2,3,7,8-tetrachlorodibenzofuran consistent with the hypothesis that 2,3,7,8-tetrachlorodibenzofuran is absent (or insufficient) The target genes are represented in terms of their measured expression change (x-axis) and the significance of the change (y-axis). The significance is represented in terms of the negative log (base 10) of the p-value, so that more significant genes are plotted higher on the y-axis. The dotted lines represent the thresholds used to select the DE genes: **1.2** for expression change and **0.05** for significance.*

## Doxorubicin

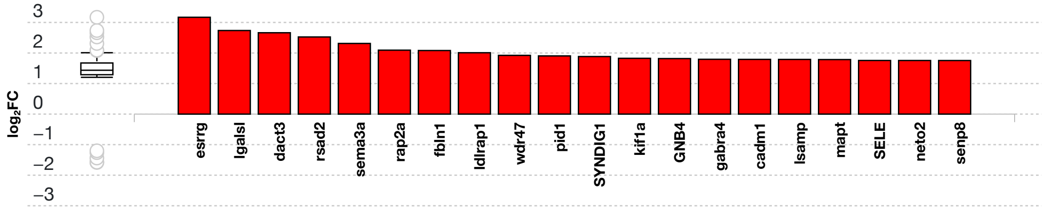


**Fig. 6.3.17: Consistent DE target genes measured expression bar plot:** *All the consistent differentially expressed genes that are targeted by Doxorubicin are ranked based on their absolute value of log fold change. The plot is limited to the top 20 genes out of a total of 122 consistent differentially expressed target genes. Upregulated genes are shown in red, downregulated genes are shown in blue. The box and whisker plot on the left summarizes the distribution of all the consistent differentially expressed genes targeted by this upstream regulator. The box shows the 1st quartile, the median and the 3rd quartile, while any outliers are represented by circles.*

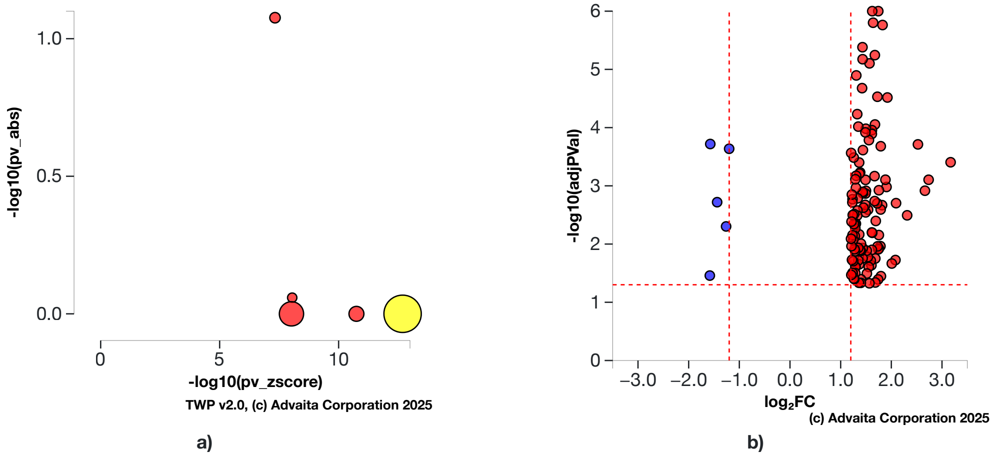


**Fig. 6.3.18: a) Absent (or insufficient) p-value vs zscore p-value:** *The significance of Doxorubicin is plotted on two axes, with negative log of $P_z$ on x-axis and negative log of $P_{abs}$ on y-axis. The size of the dot represents the relative number of consistent DE genes, which for selected upstream regulator is 122.* **b) Volcano plot:** *There are* ***122*** *DE genes that are targets of Doxorubicin consistent with the hypothesis that Doxorubicin is absent (or insufficient) The target genes are represented in terms of their measured expression change (x-axis) and the significance of the change (y-axis). The significance is represented in terms of the negative log (base 10) of the p-value, so that more significant genes are plotted higher on the y-axis. The dotted lines represent the thresholds used to select the DE genes:* ***1.2*** *for expression change and* ***0.05*** *for significance.*

## 2',3,3',4',5-pentachloro-4-hydroxybiphenyl

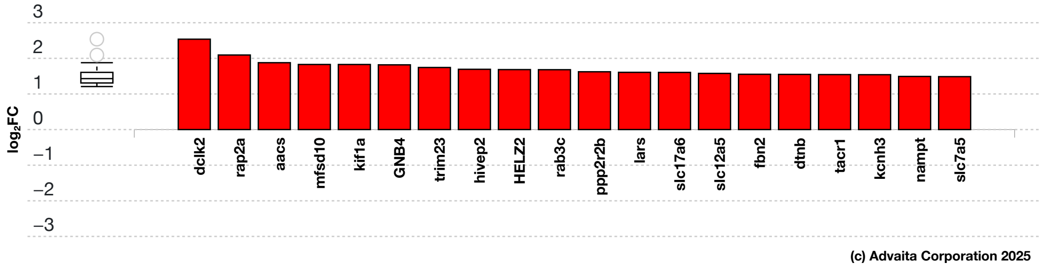


**Fig. 6.3.19: Consistent DE target genes measured expression bar plot:** *All the consistent differentially expressed genes that are targeted by 2',3,3',4',5-pentachloro-4-hydroxybiphenyl are ranked based on their absolute value of log fold change. The plot is limited to the top 20 genes out of a total of 47 consistent differentially expressed target genes. Upregulated genes are shown in red, downregulated genes are shown in blue. The box and whisker plot on the left summarizes the distribution of all the consistent differentially expressed genes targeted by this upstream regulator. The box shows the 1st quartile, the median and the 3rd quartile, while any outliers are represented by circles.*

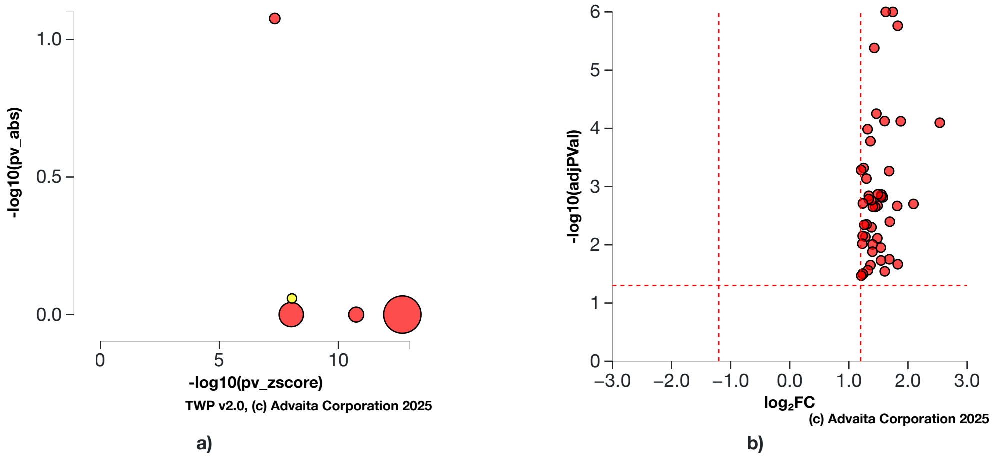


**Fig. 6.3.20: a) Absent (or insufficient) p-value vs zscore p-value:** *The significance of 2',3,3',4',5-pentachloro-4-hydroxybiphenyl is plotted on two axes, with negative log of $P_z$ on x-axis and negative log of $P_{abs}$ on y-axis. The size of the dot represents the relative number of consistent DE genes, which for selected upstream regulator is 47.* **b) Volcano plot:** *There are* ***47*** *DE genes that are targets of 2',3,3',4',5-pentachloro-4-hydroxybiphenyl consistent with the hypothesis that 2',3,3',4',5-pentachloro-4-hydroxybiphenyl is absent (or insufficient) The target genes are represented in terms of their measured expression change (x-axis) and the significance of the change (y-axis). The significance is represented in terms of the negative log (base 10) of the p-value, so that more significant genes are plotted higher on the y-axis. The dotted lines represent the thresholds used to select the DE genes:* ***1.2*** *for expression change and* ***0.05*** *for significance.*

Smoke

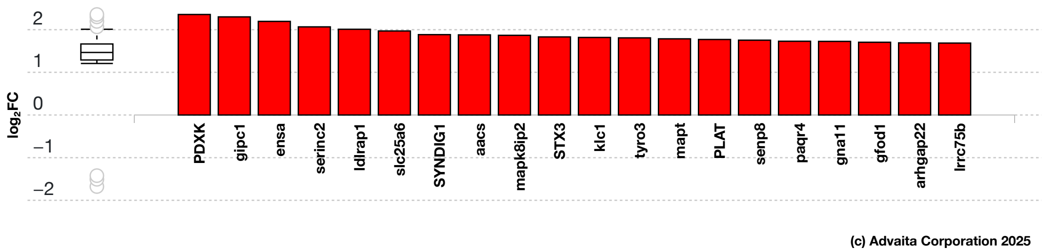


**Fig. 6.3.21: Consistent DE target genes measured expression bar plot:** *All the consistent differentially expressed genes that are targeted by Smoke are ranked based on their absolute value of log fold change. The plot is limited to the top 20 genes out of a total of 87 consistent differentially expressed target genes. Upregulated genes are shown in red, downregulated genes are shown in blue. The box and whisker plot on the left summarizes the distribution of all the consistent differentially expressed genes targeted by this upstream regulator. The box shows the 1st quartile, the median and the 3rd quartile, while any outliers are represented by circles.*

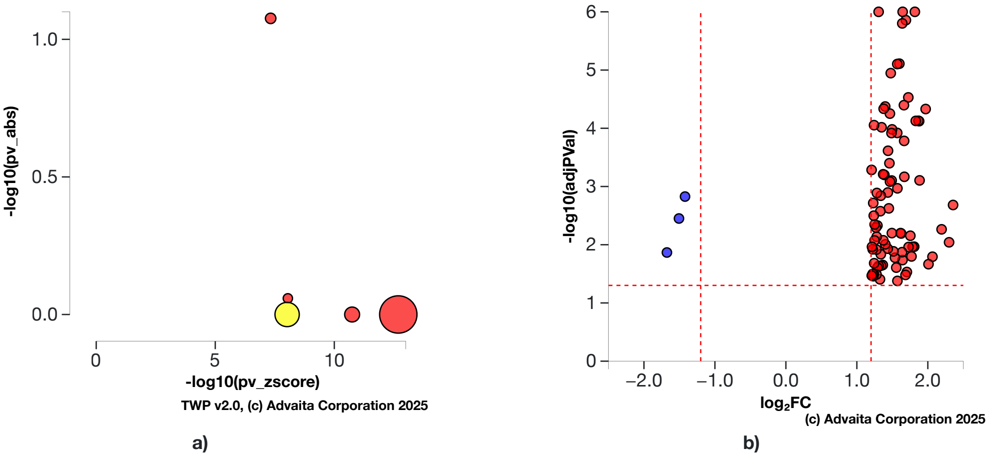


**Fig. 6.3.22: a) Absent (or insufficient) p-value vs zscore p-value:** *The significance of Smoke is plotted on two axes, with negative log of $P_z$ on x-axis and negative log of $P_{abs}$ on y-axis. The size of the dot represents the relative number of consistent DE genes, which for selected upstream regulator is 87.* **b) Volcano plot:** *There are **87** DE genes that are targets of Smoke consistent with the hypothesis that Smoke is absent (or insufficient) The target genes are represented in terms of their measured expression change (x-axis) and the significance of the change (y-axis). The significance is represented in terms of the negative log (base 10) of the p-value, so that more significant genes are plotted higher on the y-axis. The dotted lines represent the thresholds used to select the DE genes: **1.2** for expression change and **0.05** for significance.*

# 7. Disease Analysis

## 7.1. Methods

For each disease, the number of differentially expressed (DE) genes annotated to a disease term is compared to the number of DE genes expected just by chance. iPathwayGuide uses an over-representation approach to compute the statistical significance of observing at least the given number of DE genes. The p-value is computed using the hypergeometric distribution as described for pORA in the Pathway Analysis section. This p-value is corrected for multiple comparisons using FDR and Bonferroni.

## 7.2. Results

**Table 7.2.1: Top identified diseases**

| Disease Name | p-value | p-value (FDR) | p-value (Bonferroni) |
|---|---|---|---|
| Aphasia | 8.373e-4 | 0.145 | 0.373 |
| Nerve Degeneration | 0.002 | 0.145 | 0.693 |
| Ataxia | 0.002 | 0.145 | 0.848 |
| Sotos syndrome | 0.002 | 0.145 | 1.000 |
| Myocardial Ischemia | 0.005 | 0.145 | 1.000 |

## Aphasia (MESH:D001037)

In this experiment, the algorithm identified **2** differentially expressed genes out of **2** genes associated with the disease.

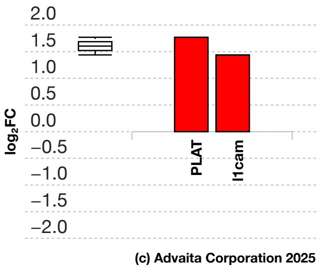


**Fig. 7.2.1: Gene measured expression bar plot:** *All the differentially expressed genes that are annotated to Aphasia are ranked based on their absolute value of log fold change. Upregulated genes are shown in red, downregulated genes are shown in blue. The box plot on the left summarizes the distribution of all the differentially expressed genes that are annotated to this disease. The box represents the 1st quartile, the median and the 3rd quartile, while the outliers are represented by circles.*

## Nerve Degeneration (MESH:D009410)

In this experiment, the algorithm identified **8** differentially expressed genes out of **76** genes associated with the disease.

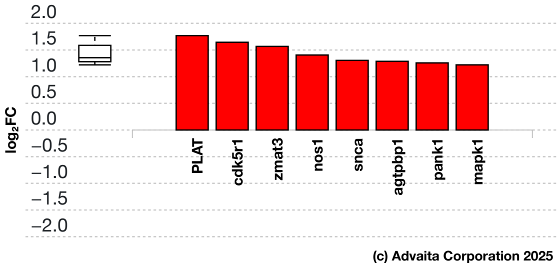


**Fig. 7.2.2: Gene measured expression bar plot:** *All the differentially expressed genes that are annotated to Nerve Degeneration are ranked based on their absolute value of log fold change. Upregulated genes are shown in red, downregulated genes are shown in blue. The box plot on the left summarizes the distribution of all the differentially expressed genes that are annotated to this disease. The box represents the 1st quartile, the median and the 3rd quartile, while the outliers are represented by circles.*

## Ataxia (MESH:D001259)

In this experiment, the algorithm identified **4** differentially expressed genes out of **19** genes associated with the disease.

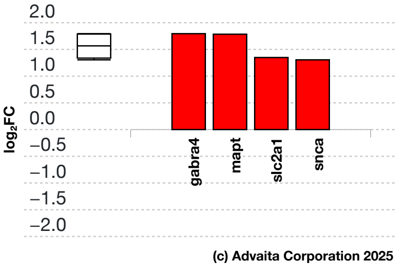


**Fig. 7.2.3: Gene measured expression bar plot:** *All the differentially expressed genes that are annotated to Ataxia are ranked based on their absolute value of log fold change. Upregulated genes are shown in red, downregulated genes are shown in blue. The box plot on the left summarizes the distribution of all the differentially expressed genes that are annotated to this disease. The box represents the 1st quartile, the median and the 3rd quartile, while the outliers are represented by circles.*

## Sotos syndrome (H00718)

Overgrowth syndromes are a heterogeneous group of disorders resulting from the dysfunction of various processes involving cell proliferation, cell growth, or apoptosis. Within this group, Sotos syndrome (SOTOS) is characterised by overgrowth, multiple congenital anomalies, and developmental delay. It has been reported that the majority of patients with typical Sotos and Weaver syndrome have intragenic mutations or deletions of NSD1, and thus, represent allelic disorders. In this experiment, the algorithm identified **2** differentially expressed genes out of **3** genes associated with the disease.

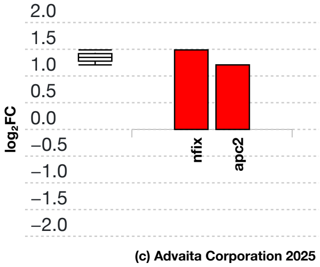


**Fig. 7.2.4: Gene measured expression bar plot:** *All the differentially expressed genes that are annotated to Sotos syndrome are ranked based on their absolute value of log fold change. Upregulated genes are shown in red, downregulated genes are shown in blue. The box plot on the left summarizes the distribution of all the differentially expressed genes that are annotated to this disease. The box represents the 1st quartile, the median and the 3rd quartile, while the outliers are represented by circles.*

### Myocardial Ischemia (MESH:D017202)

In this experiment, the algorithm identified **10** differentially expressed genes out of **132** genes associated with the disease.

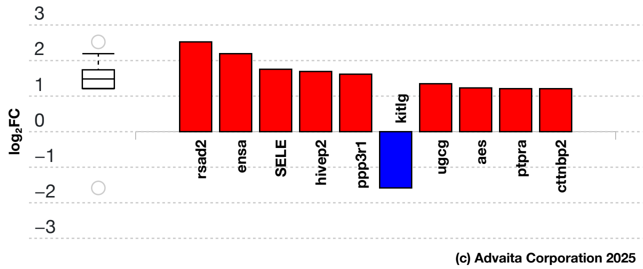


**Fig. 7.2.5: Gene measured expression bar plot:** *All the differentially expressed genes that are annotated to Myocardial Ischemia are ranked based on their absolute value of log fold change. Upregulated genes are shown in red, downregulated genes are shown in blue. The box plot on the left summarizes the distribution of all the differentially expressed genes that are annotated to this disease. The box represents the 1st quartile, the median and the 3rd quartile, while the outliers are represented by circles.*

# 8. References


- Agarwal V, Bell GW, Nam J, Bartel DP. Predicting effective microRNA target sites in mammalian mRNAs. eLife, 4:e05005 (2015).
- Alexa, A., Rahnenfuehrer, J., Lengauer,T.: Improved scoring of functional groups from gene expression data by decorrelating GO graph structure. Bioinformatics 22(13): 1600-1607 (2006).
- Ashburner, M., Ball, C.A., Blake, J.A., Botstein, D., Butler, H., Cherry, J.M., Davis, A.P., Dolinski, K., Dwight, S.S., Eppig, J.T., Harris, M.A., Hill, D.P., Issel-Tarver, L., Kasarskis, A., Lewis, S., Matese, J.C., Richardson, J.E., Ringwald, M., Rubin, G.M., Sherlock, G.: The Gene Ontology Consortium. Gene ontology: Tool for the unification of biology. Nature Genetics 25(1): 25-9 (2000).
- Ashburner, M., Lewis, S.: On Ontologies for Biologists: The Gene Ontology - Untangling the web: 'In Silico' simulation of biological processes: Novartis Found Symp, 247:66-80; discussion 80-3, 84-90: 244-52 (2002).
- Benjamini, Y. and Hochberg, Y.: Controlling the false discovery rate: A practical and powerful approach to multiple testing. Journal of the Royal Statistical Society B, 57(1):289-300, (1995).
- Benjamini, Y. and Yekutieli, D.: The control of the false discovery rate in multiple testing under dependency. Annals of Statistics, 29(4):1165-1188, (2001).
- Bonferroni, C. E.: Il calcolo delle assicurazioni su gruppi di teste, chapter "Studi in Onore del Professore Salvatore Ortu Carboni", pages 13-60, Rome, (1935).
- Bonferroni, C. E.: Teoria statistica delle classi e calcolo delle probabilita. Pubblicazioni del Istituto Superiore di Scienze Economiche e Commerciali di Firenze, 8:3-62, (1936).
- Camon, E., Magrane, M., Barrell, D., Lee, V., Dimmer, E., Maslen, J., Binns, D., Harte, N., Lopez, R., Apweiler, R.: The Gene Ontology Annotation (GOA) database: sharing knowledge in Uniprot with Gene Ontology. Nucleic Acids Research, 32(Database issue), D262-D266 (2004).
- Davis AP, Grondin CJ, Johnson RJ, Sciaky D, McMorran R, Wiegers J, Wiegers TC, Mattingly CJ, The Comparative Toxicogenomics Database: update 2019, Nucleic Acids Research, 47(D1): D948-D954 (2019).
- Draghici, S., Khatri, P., Martins, R.P., Ostermeier, G.C. and Krawetz, S.A.: Global functional profiling of gene expression. Genomics, 81(2), pp.98-104 (2003).
- Draghici, S., Khatri, P., Bhavsar, P., Shah, A., Krawetz, S., Tainsky, M.A.: Onto-Tools, The toolkit of the modern biologist: Onto-Express, Onto-Compare, Onto-Design and Onto-Translate. Nucleic Acids Research, 31(13): 3775-81 (2003).
- Draghici, S., Khatri, P., Tarca, A.L., Amin, K., Done, A., Voichita, C., Georgescu, C., Romero, R.: A systems biology approach for pathway level analysis. Genome Research, 17(10): 1537-45 (2007).
- Draghici, S.: Statistics and Data Analysis for Microarrays Using R and Bioconductor, second edition. Chapman and Hall/CRC (2011).
- Friedman, R.C., Farh, K.K., Burge, C.B., Bartel, D.P.: Most mammalian mRNAs are conserved targets of microRNAs. Genome Research, 19: 92-105 (2009).
- Garcia, D.M., Baek, D., Shin, C., Bell, G.W., Grimson, A., Bartel, D.P.: Weak seed-pairing stability and high target-site abundance decrease the proficiency of lsy-6 and other miRNAs. Nature Structural & Molecular Biology, 18: 1139-1146 (2011).
- Gene Ontology Consortium. Creating the Gene Ontology Resource: Design and Implementation. Genome Research 11: 1425-1433 (2001).
- Gene Ontology Consortium. The Gene Ontology (GO) database and informatics resource. Nucleic Acids Research 32 (suppl 1): D258-D261 (2004).
- Griffiths-Jones S.: The microRNA Registry. Nucleic Acids Research 32:D109-D111 (2004).
- Griffiths-Jones S., Grocock R.J., van Dongen S., Bateman A., Enright A.J.: miRBase: microRNA sequences, targets and gene nomenclature. Nucleic Acids Research 34:D140-D144 (2006).

- Griffiths-Jones S., Saini H.K., van Dongen S., Enright A.J.: miRBase: tools for microRNA genomics. Nucleic Acids Research 36:D154-D158 (2008).
- Grimson, A., Farh, K.K., Johnston, W.K., Garrett-Engele, P., Lim, L.P., Bartel, D.P.: MicroRNA targeting specificity in mammals: Determinants beyond seed pairing. Molecular Cell, 27: 91-105 (2007).
- Fisher R. A.: Statistical methods for research workers. Oliver & Boyd, Edinburgh, (1925).
- Kanehisa, M., Goto, S.: KEGG: Kyoto Encyclopedia of Genes and Genomes. Nucleic Acids Research 28: 27-30 (2000).
- Kanehisa, M., Goto, S., Kawashima, S., and Nakaya, A.: The KEGG databases at GenomeNet. Nucleic Acids Research 30: 42-46 (2002).
- Kanehisa, M., Goto, S., Kawashima, S., Okuno, Y., and Hattori, M.; The KEGG resources for deciphering the genome. Nucleic Acids Research 32: D277-D280 (2004).
- Kanehisa, M., Araki, M., Goto, S., Hattori, M., Hirakawa, M., Itoh, M., Katayama, T., Kawashima, S., Okuda, S., Tokimatsu, T., and Yamanishi, Y.: KEGG for linking genomes to life and the environment. Nucleic Acids Research 36: D480-D484 (2008).
- Kanehisa, M., Goto, S., Furumichi, M., Tanabe, M., Hirakawa, M.: KEGG for representation and analysis of molecular networks involving diseases and drugs. Nucleic Acids Research 38: D355-D360 (2010).
- Kanehisa, M., Goto, S., Sato, Y., Furumichi, M., Tanabe, M.: KEGG for integration and interpretation of large-scale molecular datasets. Nucleic Acids Research 40: D109-D114 (2012).
- Kanehisa, M., Goto, S., Sato, Y., Kawashima, M., Furumichi, M., and Tanabe, M.; Data, information, knowledge and principle: back to metabolism in KEGG. Nucleic Acids Research 42: D199-D205 (2014).
- Khatri, P., Draghici, S., Tarca, A.D., Hassan, S.S., Romero, R.: A system biology approach for the steady-state analysis of gene signaling networks. Lecture Notes in Computer Science (LNCS) 4756, pp 32-41 (2007).
- Kozomara A., Griffiths-Jones S.: miRBase: integrating microRNA annotation and deep-sequencing data. Nucleic Acids Research 39:D152-D157 (2011).
- Kozomara A., Griffiths-Jones S.: miRBase: annotating high confidence microRNAs using deep sequencing data. Nucleic Acids Research 42:D68-D73 (2014).
- Lewis, B.P., Burge, C.B., Bartel, D.P.: Conserved seed pairing, often flanked by adenosines, indicates that thousands of human genes are microRNA targets. Cell, 120(1):15-20 (2005).
- Nam J, Rissland OS, Koppstein D, Abreu-Goodger C, Jan CH, Agarwal V, Yildirim MA, Rodriguez A, Bartel DP. Global analyses of the effect of different cellular contexts on microRNA targeting.Molecular Cell, 53:1031-43 (2014).
- Rhee, S.Y., Wood, V., Dolinski, K., Draghici, S.: Use and misuse of the gene ontology annotations. Nature Reviews Genetics 9(4):509-515 (2008).
- Szklarczyk, D., Morris, J.H., Cook, H., *et al*. The STRING database in 2017: quality-controlled protein-protein association networks, made broadly accessible. Nucleic Acids Research 45(D1):D362-D368 (2017).
- Tarca, A.L., Draghici, S., Khatri, P., Hassan, S., Mittal, P., Kim, J.S., Kim, C.J., Kusanovic, J.P., Romero, R.: A novel Signaling Pathway Impact Analysis (SPIA). Bioinformatics 25(1), 75-82 (2009).
