# Supplementary material for: Transcriptome and Behavioral Assessment in Larval Zebrafish (Danio rerio) Following Exposure to Perfluorononanoic Acid (PFNA)
Source: Genes (Basel). 2026 May 7;17(5):558. doi: 10.3390/genes17050558 (PMC13206344; doi:10.3390/genes17050558)
Supplement: Supplementary file 1 [file genes-17-00558-s001.zip › QC-Report/L1_QC_report.html]

AZENTA NGS Sample QC Report


# AZENTA NGS Sample QC Report

## 1. Sample Information

|  |  |
| --- | --- |
| Customer | Chris Martyniuk |
| Email | cmartyn@ufl.edu |
| Quote Number | 30-1177363563 |
| Configuration | Illumina 2x150bp |
| Sample | L1 |

## 2. Sample Sequencing QC Summary

Raw data quality was evaluated with FastQC.

### 2.1 Per base quality score distribution

Figure 2.1.1 shows an overview of the range of quality scores at each base across all the reads. The x-axis represents the position in the read. The y-axis represents the quality score.

Figure 2.1.1 Base quality score distribution

### 2.2.1 Reads quality score distribution

Figure 2.2 shows the percentage of reads that have a given quality score. The x-axis represents the quality score. The y-axis represents the percentage of reads that have an equal or higher quality score.

Figure 2.2.1 Reads quality score distribution

### 2.3.1 Reads GC content distribution

Figure 2.3 shows the distribution of average GC content across all the reads. The x-axis represents the GC content percentage. The y-axis represents the percentage of reads with the given GC content.

Figure 2.3.1 Reads GC content distribution

- Azenta Life Sciences
- Next Generation Sequencing
- Email: ngs@azenta.com
- Phone: 908-222-0711 ext 1
