# Supplementary material for: Transcriptome and Behavioral Assessment in Larval Zebrafish (Danio rerio) Following Exposure to Perfluorononanoic Acid (PFNA)
Source: Genes (Basel). 2026 May 7;17(5):558. doi: 10.3390/genes17050558 (PMC13206344; doi:10.3390/genes17050558)
Supplement: Supplementary file 1 [file genes-17-00558-s001.zip › RNASeq_report.html]

AZENTA RNA-Seq Analysis Report


# AZENTA RNA-Seq Analysis Report

## 1. Project Summary

|  |  |
| --- | --- |
| Customer | Chris Martyniuk |
| Email | cmartyn@ufl.edu |
| Sample Type | zebrafish RNA |
| Quote Number | 30-1177363563 |
| Configuration | Illumina 2x150bp |

## 2. Description of Workflow

### 2.1 RNA-seq library preparation workflow

Figure 2.2.1 mRNA sequencing via polyA selection

### 2.2 Bioinformatics analysis workflow

Figure 2.2.2 Bioinformatics analysis workflow. (Standard Package)

## 3. Analysis

### 3.1 Sample sequencing statistics

### 3.2 Overall sequencing statistics

### 3.3 Mapping sequence reads to the reference genome

Sequence reads were trimmed to remove possible adapter sequences and nucleotides with poor quality using Trimmomatic v.0.36. The trimmed reads were mapped to the Danio rerio GRCz10.89 reference genome available on ENSEMBL using the STAR aligner v.2.5.2b. The STAR aligner is a splice aligner that detects splice junctions and incorporates them to help align the entire read sequences. BAM files were generated as a result of this step. Below are the statistics of mapping the reads to the reference genome.

### 3.4 Extracting gene hit counts

Unique gene hit counts were calculated by using featureCounts from the Subread package v.1.5.2. The hit counts were summarized and reported using the gene\_id feature in the annotation file. Only unique reads that fell within exon regions were counted. If a strand-specific library preparation was performed, the reads were strand-specifically counted.

### 3.5 Differential gene expression analysis

After extraction of gene hit counts, the gene hit counts table was used for downstream differential expression analysis. Using DESeq2, a comparison of gene expression between the customer-defined groups of samples was performed. The Wald test was used to generate p-values and log2 fold changes. Genes with an adjusted p-value < 0.05 and absolute log2 fold change > 1 were called as differentially expressed genes for each comparison. Below are the results of the number of significantly differentially expressed genes for all comparisons provided.

### 3.6 Gene ontology analysis

A gene ontology analysis was performed on the statistically significant set of genes by implementing the software GeneSCF v.1.1-p2. The zfin GO list was used to cluster the set of genes based on their biological processes and determine their statistical significance. A list of genes clustered based on their gene ontologies was generated.

### 3.7 Splice variant expression analysis

To estimate the expression levels of alternatively spliced transcripts, the splice variant hit counts were extracted from the RNA-seq reads mapped to the genome. Differentially spliced genes were identified for groups with more than one sample by testing for significant differences in read counts on exons (and junctions) of the genes using DEXSeq. For groups with only one sample, the exon hit count tables were provided. The results of the splice variant expression analysis are included in separate reports, and provided in the zipped format as ‘differential\_splice\_variant\_expression.zip’.

## 4. FAQs

If you have any further questions or concerns, please consider examining our FAQ page located here.

## 5. Deliverables

- 00\_fastq: raw data
  - raw data in fastq format: *sample*\_R1\_001.fastq.gz *sample*\_R2\_001.fastq.gz
- bam: aligned data
  - alignment file in bam format: *sample*.bam
- hit-counts: hit count for all the genes
  - unique hit counts for each sample: *sample*.counts.txt
- DEG: differential gene expression analysis results
  - raw gene hit counts of all samples used in the comparison: counts/raw\_counts.csv
  - normalized gene hit counts of all samples used in the comparison: counts/normalized\_counts.csv
  - rlog transformed gene hit counts of all samples used in the comparison: counts/rlog\_trasnformed\_counts.csv
  - log2 fold change and (adjusted) p-value table: Differential\_expression\_analysis\_table.csv
  - log2 fold change and (adjusted) p-value table for significantly DE genes: Significant-DEGs.csv
- GO: GO enrichment analysis results
  - GO enrichment analsysis table: *comparison*\_GO\_analysis.csv
- differential\_splice\_variant\_expression: differential splicing anlysis results
  - Results generated by DEXSeq: DEXSeqReport in zipped format

- Azenta Life Sciences
- Next Generation Sequencing
- Email: ngs@azenta.com
- Phone: 908-222-0711 ext 1
