# Supplementary material for: Transcriptome and Behavioral Assessment in Larval Zebrafish (Danio rerio) Following Exposure to Perfluorononanoic Acid (PFNA)
Source: Genes (Basel). 2026 May 7;17(5):558. doi: 10.3390/genes17050558 (PMC13206344; doi:10.3390/genes17050558)
Supplement: Supplementary file 1 [file genes-17-00558-s001.zip › Suppl. Figures.pdf]

Supplemental Figures

Transcriptome and behavioral assessment in larval zebrafish (*Danio rerio*) following exposure to perfluorononanoic acid (PFNA)

Lev Avidan[1†], Cole D. English[1†], Katie A. McDonnell[1], Emma Ivantsova[1], Christopher J. Martyniuk[1,2*]

[1] Center for Environmental and Human Toxicology, Department of Physiological Sciences, College of Veterinary Medicine, University of Florida, Gainesville, Florida, 32611, USA
[2] UF Genetics Institute, Interdisciplinary Program in Biomedical Sciences Neuroscience
* Correspondence: cmartyn@ufl.edu
† Authors contributed equally to the study.

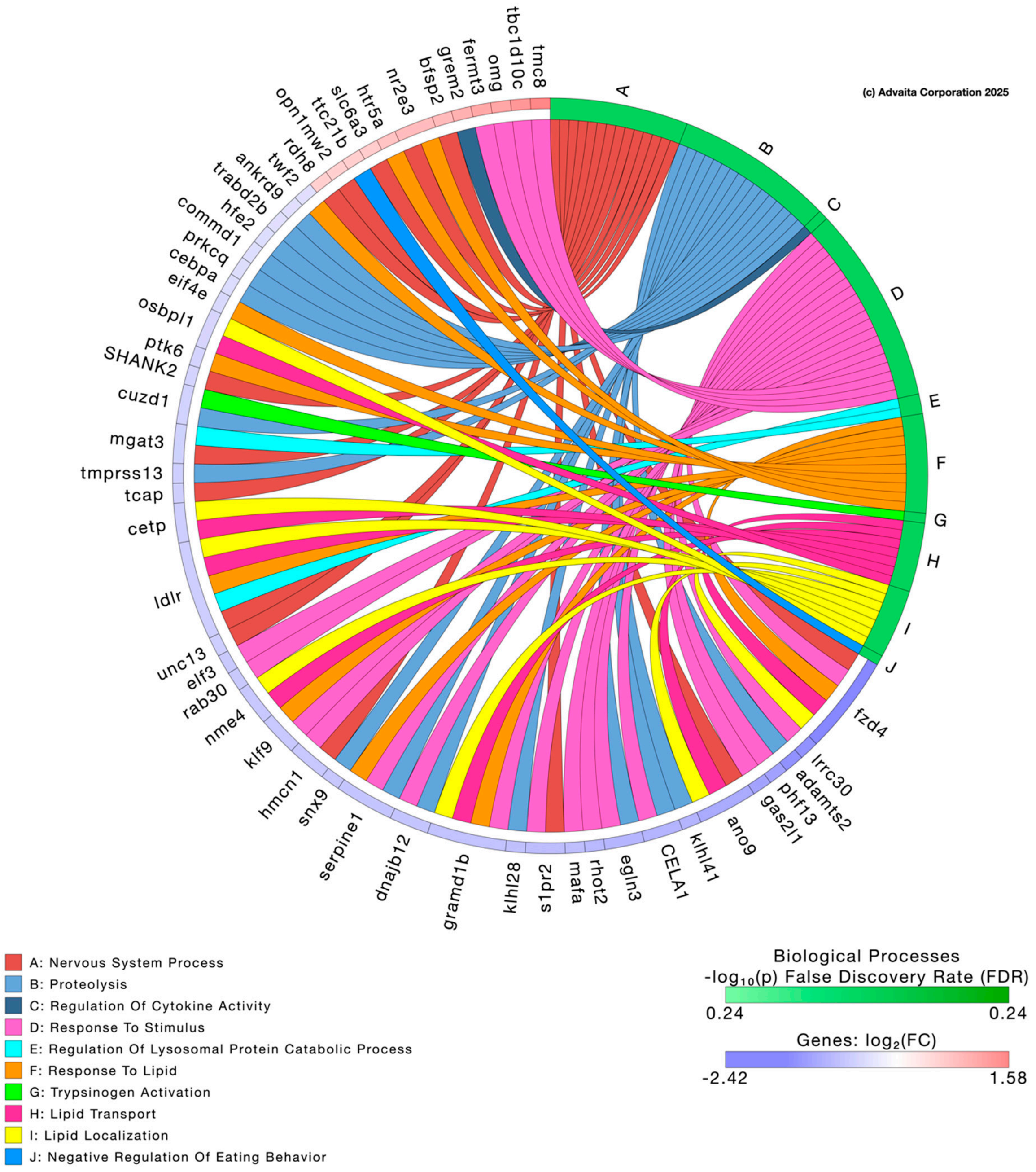

Figure S1. Biological processes (gene ontology) identified as enriched based on iPathway using a chord diagram (FDR < 0.05). The diagram depicts how each gene relates to the top biological pathways following 0.1 μg/L PFNA exposure.

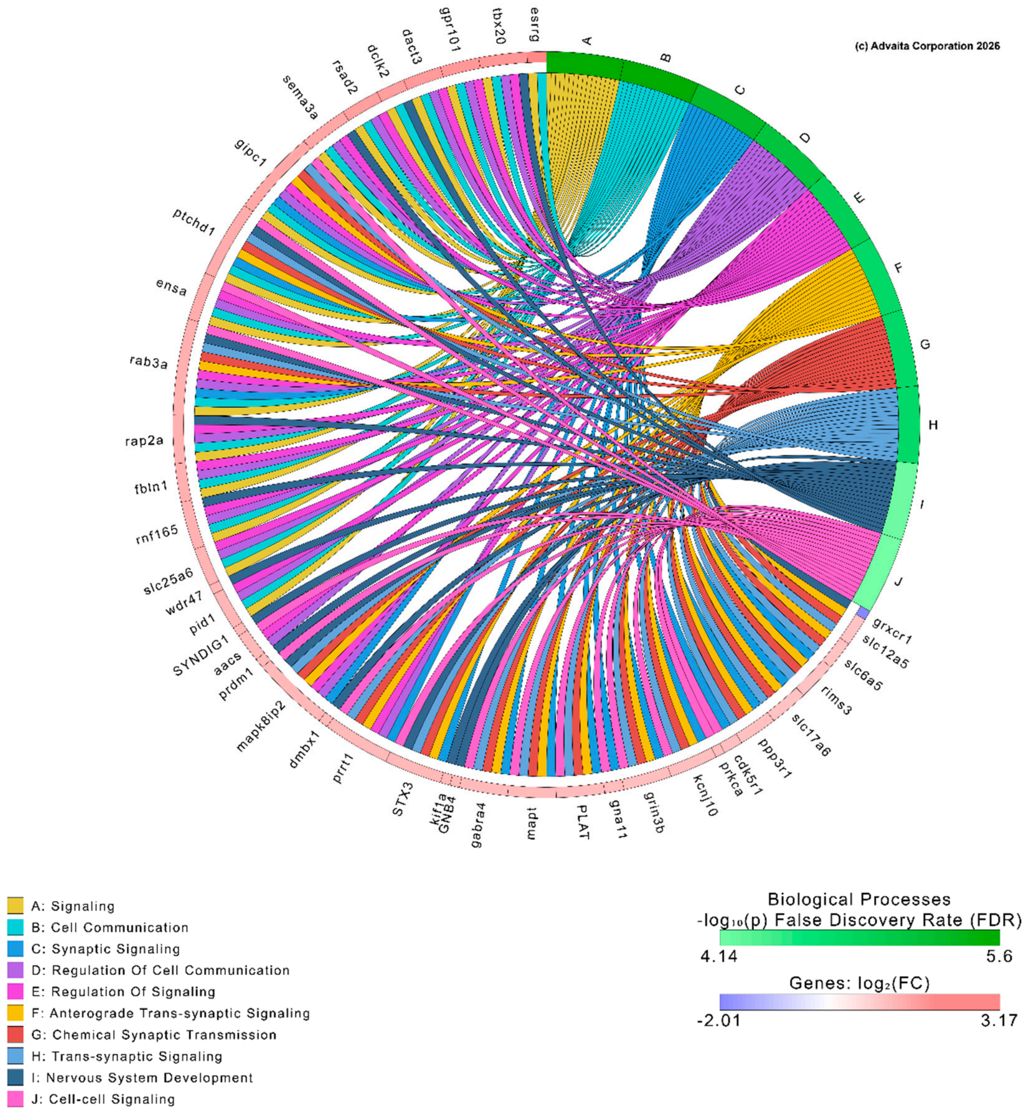

Figure S2. Biological processes (gene ontology) identified as enriched based on iPathway using a chord diagram (FDR < 0.05). The diagram depicts how each gene relates to the top biological pathways following 10 μg/L PFNA exposure.

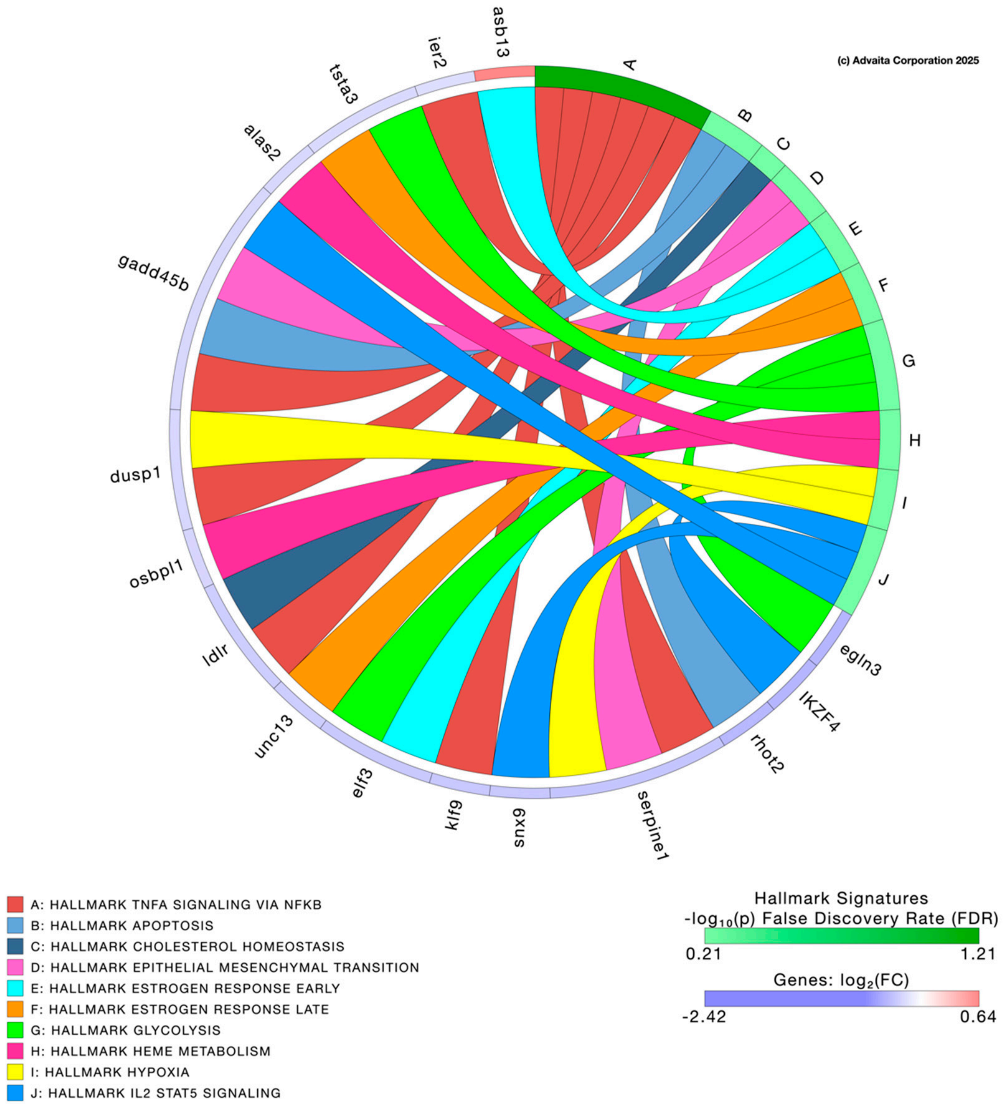

Figure S3. Chord diagram illustrates the relationships between differentially expressed genes and enriched Hallmark gene signatures following 0.1 μg/L PFNA exposure.

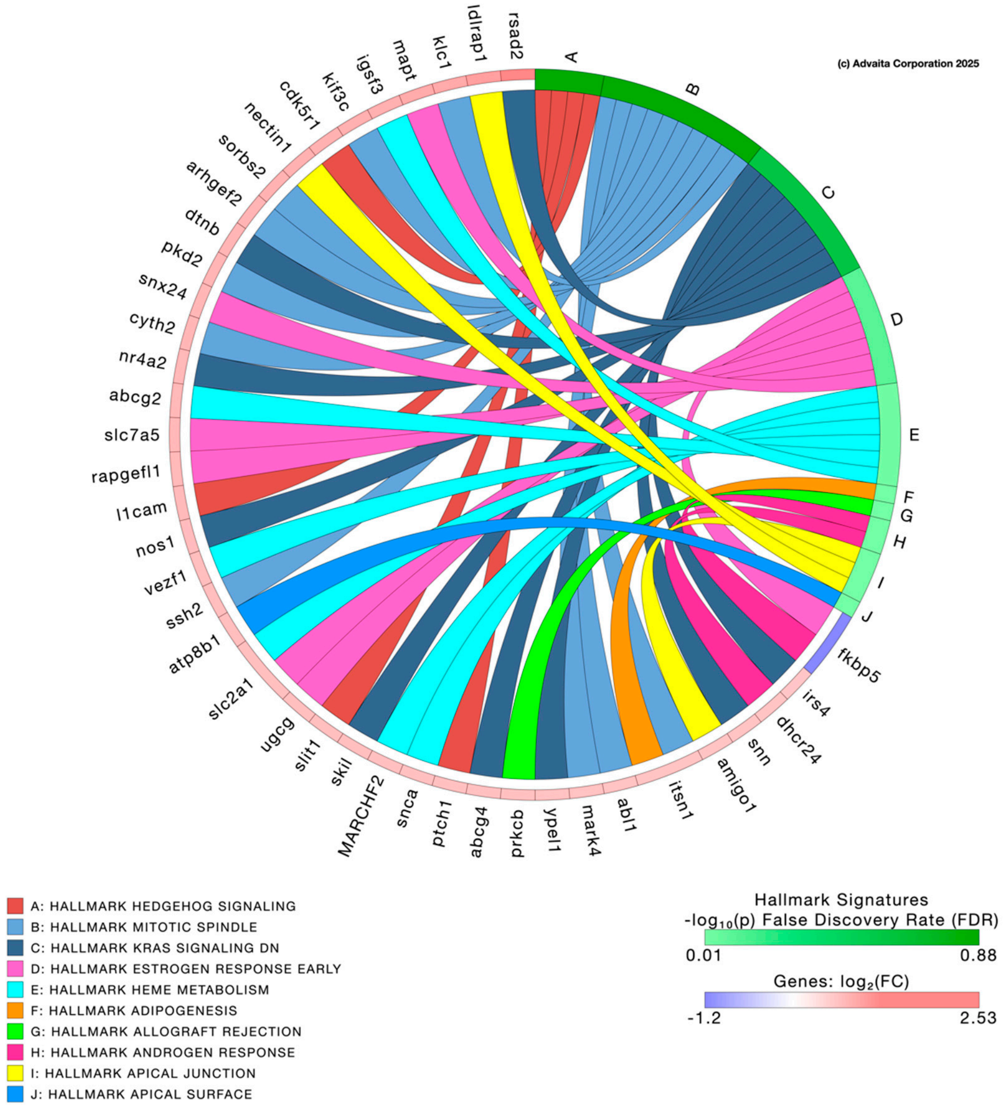

Figure S4. Chord diagram illustrates the relationships between differentially expressed genes and enriched Hallmark gene signatures following 10 µg/L PFNA exposure.
