# Supplementary material for: Transcriptome and Behavioral Assessment in Larval Zebrafish (Danio rerio) Following Exposure to Perfluorononanoic Acid (PFNA)
Source: Genes (Basel). 2026 May 7;17(5):558. doi: 10.3390/genes17050558 (PMC13206344; doi:10.3390/genes17050558)
Supplement: Supplementary file 1 [file genes-17-00558-s001.zip › Suppl. Methods.pdf]

*Supplemental Methods*

*Article*

# Transcriptome and behavioral assessment in larval zebrafish (*Danio rerio*) following exposure to perfluorononanoic acid (PFNA)

**Lev Avidan[1†], Cole D. English[1†], Katie A. McDonnell [1], Emma Ivantsova[1], Christopher J. Martyniuk[1,2*]**

, College of Veterinary Medicine, University of Florida, Gainesville, Florida, 32611, USA
[2] UF Genetics Institute, Interdisciplinary Program in Biomedical Sciences Neuroscience
* Correspondence: cmartyn@ufl.edu
† Authors contributed equally to the study.

*2.1 Chemical Preparation*

PFNA was purchased from Sigma-Aldrich (CAS no. 375-95-1, purity 97%). Stock solutions of 0.1 µg/L, 1 µg/L, 10 µg/L, 100 µg/L, and 1000 µg/L PFNA were prepared in 0.1% dimethyl sulfoxide (DMSO, CAS 67-68-5, Sigma-Aldrich, Inc., St. Louis, MO, USA) and added to embryo rearing media (ERM) containing the zebrafish embryos. Recipes for ERM were obtained from Westerfield [1] and reported in full in [2]. Exposure solutions were prepared daily to yield final concentrations of 0.1, 1, 10, 100, and 1000 µg/L PFNA.

*2.2. Husbandry and egg production of zebrafish*

Adult zebrafish (AB x Tübingen, *Danio rerio,* 6 months of age) were raised in a flow-through Pentair system in the Cancer-Genetics Research Center at the University of Florida and are derived from an ongoing zebrafish breeding colony. The colony is infused with new breeders from Zebrafish International Resource Center (ZIRC) every 3 to 4 generations to maintain genetic variation. Environmental conditions for zebrafish breeding have been previously described [3, 4]. Staging of embryos followed that of Kimmel et al. [5]. Fish are maintained at a temperature of 27±1 °C, air saturation at 82%, water pH between 7.2 and 7.3, conductivity of 1,500–1,600 µS/cm, and 14:10 h light/dark cycle. Institutional Animal Care and Use Committee of University of Florida approved all experiments (UF IACUC202300000140). To obtain eggs for experiments, 2 males and 2 females were placed into a standard zebrafish breeding tank and remained in clean, aerated water with artificial foliage overnight. The next day, lights were turned on at approximately 8:30 and eggs collected at 9:00 a.m.

*2.3. PFNA exposure regime*

The embryonic acute toxicity test followed recommendations from the OECD guideline 236 with modification [6]. For each independent experiment, 20 healthy and fertilized embryos were randomly assigned to 25 mL Pyrex glass beakers with 11 mL sterile ERM with designated concentrations spanning environmentally relevant values from 0.1 μg/L to 1000 μg/L PFNA. Fish were distributed in a randomized and staggered fashion. Fish were subjected to a cycle of 14 h light and 10 h darkness at 27 ± 1 °C. On each day of the 7-day experiment, dead embryos/larvae were removed promptly, and an 80% water change was conducted with chemical dilutions that were made fresh daily. Either a Keyence All-in-One Fluorescence Microscope BZ-X710 or EVOS™ FL Auto Imaging System (Thermo Scientific, USA) were used to collect images of the embryos to document the mortality, hatch rate, and deformities. Four fish per beaker with 6 beakers from each treatment group ($n$ = 24 per treatment group) were evaluated. Several replicate experiments ($n$ = 6) were required to obtain sufficient larvae for all toxicity assays and sub-lethal endpoints investigated in this study. Tricaine mesylate (Syncaine, Tricaine-S) was used for euthanasia at 250 mg/L buffered with equal part sodium bicarbonate to a pH between 7.0–7.5.

*2.4. Acridine Orange*

The acridine orange (AO) staining method was conducted to detect apoptotic cells in zebrafish larvae. Briefly, zebrafish larvae were exposed to ERM, 0.1% DMSO, 1, or 100 μg/L PFNA for 7 days. Then, they were washed with ERM and stained with 2 μg/mL AO solution (CAS: 65-61-2, Sigma-Aldrich) for 30 min at room temperature in the absence of light. Ten larvae from each biological replicate were used, totaling 50 larvae per treatment. After washing with ERM (5 times for 30 s), apoptotic cells were visualized with an EVOSTM FL Auto Imaging System (ThermoFisher Scientific, USA) using a GFP filter at 10× magnification. Fluorescence patches of bright green color denoted apoptotic cells. The fluorescence intensity was quantified using the histogram tool of the ImageJ software.

*2.5. Reactive oxygen species*

Embryos were obtained for reactive oxygen species (ROS) assessment as outlined above in section 2.2. Embryos at 6 hours post-fertilization (hpf) were washed 3 times in ERM and fertilized eggs were distributed evenly among small beakers using sterile micropipettes, each containing ~30 embryos in 15 mL of an assigned chemical concentration. The exposure concentrations were ERM, 0.1% DMSO, 0.1, 10, or 1000 μg/L PFNA ($n$ = 6 beakers per experimental group). Following exposure, zebrafish larvae were transferred to 1.7 mL microcentrifuge tubes, flash frozen in liquid nitrogen, and homogenized in 200 μL of ice-cold PBS. Samples were then centrifuged at 12,000 g for 20 min at 4° C (LYNX6000, Thermo Scientific). For the ROS fluorescence assay, 20 μL of supernatant was transferred to a black fluorescence plate and incubated at room temperature for 5 minutes. After incubation, 8.3 μL of H2-DCFDA (1 mg/mL) and 200 μL of PBS were added. The mixture was incubated in the

dark for 30 minutes at 37° C ± 1.0° C, and then the contents were measured with an excitation at 485 nm and emission at 520 nm using a multi-detection microplate reader (New Synergy™ 4, BioTek). Total protein was determined for each sample using a BCA assay (Thermo Scientific) ROS levels were expressed as normalized signal intensity/(μg/mL) protein.

*2.6. RNA-seq*

Samples were collected following the 7-day exposure as outlined in Section 2.3 for RNA-seq. Each beaker of fish containing 14–15 individuals were pooled to generate a biological replicate. Total RNA was extracted using TRIzol (Thermo Fisher Scientific, Waltham, MA USA). The concentration was determined using the Qubit® 2.0 Fluorometer (Ther-moFisher, Grand Island, NY, USA). A total of 12 RNA samples were used for RNA-seq library construction (RIN > 7) (Agilent 2100 Bioanalyzer, Agilent Technologies, Inc.). Biological replicates were as follows: 0.1% DMSO controls ($n$ = 3), 0.1 μg/L PFNA ($n$ = 3), and 10 μg/L PFNA ($n$ = 3).

Total RNA was used for mRNA isolation using the NEBNext Ploy(A) mRNA Magnetic Isolation module (New England Biolabs, catalog # E7490). RNA library construction was then performed with the NEBNext® Ultra™ II Directional RNA Library Prep Kit for Illumina® (New England Biolabs, catalog #E7760) according to the manufacturer's user guide. Individually prepared libraries were pooled by equimolar and sequenced by GeneWiz (Azenta Life Sciences, Burlington, Massachusetts, USA).

Sequence reads were trimmed to remove possible adapter sequences and nucleotides with poor quality using Trimmomatic v.0.36. The trimmed reads were mapped to the Danio rerio GRCz10.89 reference genome available on ENSEMBL using the STAR aligner v.2.5.2b and BAM files were generated. Statistics of mapping the reads to the reference genome. Unique gene hit counts were calculated by using featureCounts from the Subread package v.1.5.2. The hit counts were summarized and reported using the gene_id feature in the annotation file. Only unique reads that fell within exon regions were counted. If a strand-specific library preparation was performed, the reads were strand-specifically counted. Distribution of read counts in libraries were examined before and after normalization. The original read counts were normalized to adjust for various factors such as variations of sequencing yield between samples. These normalized read counts were used to accurately determine differentially expressed genes (Median of Ratios Method default in DESeq2). After extraction of normalized gene hit counts, the gene hit counts table was used for downstream differential expression analysis. Using DESeq2, a comparison of gene expression between each treatment and the control group was performed. The Wald test was used to generate p-values and log2 fold changes. Genes with an adjusted p-value < 0.05 and absolute log2 fold change > 1 were called as differentially expressed genes for each comparison. Excel files containing read count, fpkm, log2 fold change, p-value are provided in Supplemental Data files. All raw and processed

transcriptome data have been deposited in the NCBI Gene Expression Omnibus (GEO) database (GSE324353).

*2.7. Bioinformatics*

Volcano plots to reveal significant transcripts with high log2 expression values. Functional analysis (gene ontology, was conducted for each concentration of PFNA (low, 0.1 µg/L; high, 10 µg/L) in iPathway. Functional analysis [Molecular Signature analysis/Gene Ontology Term Enrichment analysis] was done using iPathwayGuideTM (AdvaitaBio Corporation, https://ipathwayguide.advaitabio.com/). The Chord Diagrams and Pathway Images were generated using iPathwayGuideTM (AdvaitaBio Corporation, https://ipathwayguide.advaitabio.com/) [23, 24]. Analyses utilized pathways from the KEGG (Release 100.0+/11–12 November 2021) and gene ontologies from the Gene Ontology Consortium (4 November 2021). Enrichment analyses incorporated miRNA data from miRBase (v22.1, October 2018) and TargetScan (Mouse v8.0, Human v8.0), regulatory networks from BioGRID (v4.4.203, October 2021), and toxicant and disease associations from the Comparative Toxicogenomics Database and KEGG databases. Compete reports outlining methodology are provided in Supplemental Files.

*2.8. Real-time PCR analysis*

To conduct both real-time PCR assays and VMR, a double batch of fish were bred as per section 2.2. This breeding event included 2 different sets of parents and yielded over 320 eggs. After the toxicity assay, a subset of fish was flash frozen in liquid nitrogen. Samples were then stored at −80 °C for RNA extraction and real time PCR analysis (0.1% DMSO, 0.1 µg/L, 1 µg/L, and 10 µg/L µg/L; $n$ = 3-8 biological replicates per group). Biological replicates varied due to balancing replicates (beakers), concentrations, and larval fish numbers. Fish were treated for 7 days to PFNA prior to sample collection.

Real-time PCR followed our established protocols using TRIzol® Reagent (Life Technologies, Carlsbad, CA, USA) [7]. Following extraction using TRIzol® Reagent, RNA integrity was determined using the RNA 6000 nano kit and 2100 Bioanalyzer (Agilent Technologies, Santa Clara, CA, USA). Samples with RNA integrity values greater than 7 were used for downstream analyses. Genomic DNA was removed using the TURBO DNA free™ Kit as per manufacturer's instructions (ThermoFisher Scientific). The cDNA step was conducted using 500 ng RNA and the iScript™ Select cDNA Synthesis Kit (Bio-Rad, CA, USA). Three "no reverse transcriptase (NRT)" controls were prepared in the same fashion except water was used instead of enzyme. The T100™ Thermal Cycler (BioRad, USA) was used to cycle temperatures needed to generate cDNA as per manufacturer's instruction. The CFX Connect System (BioRad, USA) was used to perform quantitative polymerase chain reaction (RT-qPCR) with SSo-Fast™ EvaGreen® Supermix Kit (BioRad, Hercules, CA, USA). Samples were run in duplicate and followed RT-qPCR cycling parameters described by us [8].

Primers used in this study were obtained from published literature [8-15] and are listed in Supplemental Table S1. The transcripts measured in this study included acetylcholinesterase (*ache*), BCL2-associated X protein (*bax*), B-cell Lymphoma 2 (*bcl2*), Caspase-3 (*casp3*), Catalase (*cat*), ELOVL Fatty Acid Elongase 3 (*elovl3*), ELOVL Fatty Acid Elongase 6 (*elovl6*), Growth Associated Protein 43 (*gap43*), Glial Fibrillary Acidic Protein (*gfap*), Heme Oxygenase 1 (*hmox1*), Heat Shock Protein Family A Member 4 (*hspa4*), Mesencephalic Astrocyte-Derived Neurotrophic Factor (*manf*), Microtubule-Associated Protein Tau A (*maptA*), Microtubule-Associated Protein Tau B (*maptB*), Myelin Basic Protein (*mbp*), Mitochondrially Encoded Cytochrome C Oxidase I (*mt-co1*), Nestin (*nes*), Nuclear Factor, Erythroid 2 Like 2 (*nfe212*), NAD(P)H Quinone Dehydrogenase 1 (*nqo1*), Tumor Protein p53 (*p53*), Sonic Hedgehog Signaling Molecule (*shh*), Superoxide Dismutase 1 (*sod1*), Superoxide Dismutase 2 (*sod2*), and Tubulin beta 3 class III (*tubb3*). Beta-Actin (*bactin*) and Ribosomal Protein S18 (*rps18*) were used to normalize expression levels of all target genes using the CFX Manager (v3.1) software. Normalized expression was obtained for each target gene using CFX Manager™ software (v3.1) (baseline subtracted) and the Cq method was employed.

*2.9. Visual motor response test*

The Visual Motor Response (VMR) test was employed to test for differences in locomotor activity after exposure to PFNA. Zebrafish embryos were collected for the VMR test after multiple embryo toxicity tests. The experimental groups included 0.1% DMSO, ERM, 1 μg/L, 10 μg/L, 100 μg/L, and 1000 μg/L PFNA. Multiple replicate beakers were used for each toxicity experiment (*n* = 5-10 in each experiment).

Larvae were exposed continuously for 7 days to designated concentrations in glass beakers containing 11 mL ERM. In each trial, ~120 zebrafish embryos at 6 hpf were randomly assigned to an experimental group of either ERM control or one concentration of 0.1% DMSO, ERM, 1 μg/L, 10 μg/L, 100 μg/L, and 1000 μg/L PFNA (*n* = 4 fish derived from replicate beakers/treatment, 4 independent experiments). An 80% daily water change was conducted daily to renew the PFNA. The experiments were conducted at a temperature of 27 ± 1 °C and photoperiod of 14:10 h in benchmark mini ovens.

On the seventh day in the mid-afternoon, 2 to 3 normally developed larvae were selected from each replicate beaker and individually placed into a 96-well plate (*n*=16 individuals/treatment). Each well contained 200 μL of ERM. The 96-well plate was placed into DanioVision™ Observation Chamber (Noldus Information Technology, Leesburg, VA) with an infrared analog camera (25 frames/ second) to track larval locomotor activity. The VMR test proceeded as per our established methods [3, 7, 16]. Data were analyzed by normalizing each VMR run to 0.1% DMSO control group = relative value of 1. Then each treatment was analyzed relative to the control group of 1. Following this initial standardization within a VMR run, the 4

runs were combined into a single graph and analyzed to reveal overall patterns across multiple experiments.

*2.10. Statistical analysis*

Statistical analysis and graphing were conducted using GraphPad v9.5.1 (La Jolla, CA, USA). Data were first assessed for normality using a Shapiro-Wilk test and gene expression data were log transformed to approximate a normal distribution. Survival was analyzed with a Kaplan-Meier test (log-rank Mantel-Cox test). Statistical hypothesis testing was not employed to analyze deformity and hatch data as hatch rate was observably unaltered, and deformity frequency was low (<2%) in all treatment groups. Apoptosis, reactive oxygen species [relative fluorescence units (μg/mL protein)], gene expression levels, and the VMR for larval zebrafish were analyzed using a One-Way ANOVA, followed by a Dunnett's multiple comparisons test to the control group (mean ± SD). Significance of difference was determined to be $p < 0.05$.


1. Westerfield, M., *The Zebrafish Book University of Oregon Press.* Eugene, OR, 1995.
2. Wang, S., et al., *Assessing sub-lethal effects of the dinitroaniline herbicide pendimethalin in zebrafish embryos/larvae (Danio rerio).* Neurotoxicology and Teratology, 2022. **89**: p. 107051.
3. Huang, T., et al., *Behavioral and developmental toxicity assessment of the strobilurin fungicide fenamidone in zebrafish embryos/larvae (Danio rerio).* Ecotoxicology and Environmental Safety, 2021. **228**: p. 112966.
4. Perez-Rodriguez, V., et al., *Tebuconazole reduces basal oxidative respiration and promotes anxiolytic responses and hypoactivity in early-staged zebrafish (Danio rerio).* Comparative Biochemistry and Physiology Part C: Toxicology & Pharmacology, 2019. **217**: p. 87-97.
5. Kimmel, C.B., et al., *Stages of embryonic development of the zebrafish.* Developmental dynamics, 1995. **203**(3): p. 253-310.
6. No, O.T., *236: Fish embryo acute toxicity (FET) test.* OECD guidelines for the testing of chemicals, section, 2013. **2**(10.1787).
7. Liang, X., et al., *Biological effects of the benzotriazole ultraviolet stabilizers UV-234 and UV-320 in early-staged zebrafish (Danio rerio).* Environmental pollution, 2019. **245**: p. 272-281.
8. Wang, X.H., et al., *Paraquat affects mitochondrial bioenergetics, dopamine system expression, and locomotor activity in zebrafish (Danio rerio).* Chemosphere, 2018. **191**: p. 106-117.
9. Deng, J., et al., *Hexabromocyclododecane-induced developmental toxicity and apoptosis in zebrafish embryos.* Aquatic Toxicology, 2009. **93**(1): p. 29-36.
10. English, C.D., et al., *Neurotoxicity assessment of the herbicide pethoxamid in zebrafish (Danio rerio) embryos/larvae.* Neurotoxicology and Teratology, 2024. **104**: p. 107369.
11. Guo, Y., Y. Fu, and W. Sun, *50 hz magnetic field exposure inhibited spontaneous movement of zebrafish larvae through ros-mediated Syn2a expression.* International Journal of Molecular Sciences, 2023. **24**(8): p. 7576.
12. Liu, M., et al., *The expression of zebrafish NAD (P) H: quinone oxidoreductase 1 (nqo1) in adult organs and embryos.* Gene Expression Patterns, 2020. **38**: p. 119134.
13. Sarkar, S., et al., *Low dose of arsenic trioxide triggers oxidative stress in zebrafish brain: expression of antioxidant genes.* Ecotoxicology and environmental safety, 2014. **107**: p. 1-8.
14. Northam, C. and C.M. LeMoine, *Metabolic regulation by the PGC-1α and PGC-1β coactivators in larval zebrafish (Danio rerio).* Comparative Biochemistry and Physiology Part A: Molecular & Integrative Physiology, 2019. **234**: p. 60-67.

15. Wu, Q., et al., *Microcystin-LR exposure induces developmental neurotoxicity in zebrafish embryo.* Environmental Pollution, 2016. **213**: p. 793-800.
16. Huang, T., et al., *Exposure to acetochlor impairs swim bladder formation, induces heat shock protein expression, and promotes locomotor activity in zebrafish (Danio rerio) larvae.* Ecotoxicology and Environmental Safety, 2021. **228**: p. 112978.
