# Supplementary material for: Transcriptome and Behavioral Assessment in Larval Zebrafish (Danio rerio) Following Exposure to Perfluorononanoic Acid (PFNA)
Source: Genes (Basel). 2026 May 7;17(5):558. doi: 10.3390/genes17050558 (PMC13206344; doi:10.3390/genes17050558)
Supplement: Supplementary file 1 [file genes-17-00558-s001.zip › Suppl. Table S1.pdf]

Transcriptome and behavioral assessment in larval zebrafish (*Danio rerio*) following exposure to perfluorononanoic acid (PFNA)

Lev Avidan[1†], Cole D. English[1†], Katie A. McDonnell [1], Emma Ivantsova[1], Christopher J. Martyniuk[1,2*]

Supplemental Table S1. Primers used for real-time PCR analysis.

**Gene name**	**Gene symbol**	**Forward (5' to 3')**	**Reverse (5' to 3')**
Acetylcholinesterase	*ache*	GCTAATGAGCAAAAGCATGTGGGC	TATCTGTGATGTTAAGCAGACGAGGCA
Beta-actin	*bactin*	CGAGCAGGAGATGGGAACC	CAACGGAAACGCTCATTGC
BCL2-associated X protein	*bax*	GGCTATTTCAACCAGGGTTCC	TGCGAATCACCAATGCTGT
B-cell Lymphoma 2	*bcl2*	AACCCAAATTCTGCGCAACG	ATCTACCTGGGACGCCATCT
Caspase-3	*casp3*	CCGCTGCCCATCACTA	ATCCTTTCACGACCATCT
Catalase	*cat*	CTCCTGATGTGGCCCGATAC	TCAGATGCCCGGCCATATTC
ELAV-like RNA-binding protein 3	*elavl3*	AGACAAGATCACAGGCCAGAGCTT	TGGTCTGCAGTTTGAGACCGTTGA
Elongation of very long chain fatty acids protein 6	*elovl6*	TCGTGTTACGCAAGCAGAAG	CACCATCTGGGTGATCTGTG
Growth Associated Protein 43	*gap43*	TGCTGCATCAGAAGAACTAA	CCTCCGGTTTGATTCCATC
Glial Fibrillary Acidic Protein	*gfap*	GGATGCAGCCAATCGTAAT	TTCCAGGTCACAGGTCAG
Heme Oxygenase-1	*hmox1*	AAGCAAAGCGGCAGAGAAC	TGGAGCAGTCAGATGAAGTGT
Heat Shock Protein Family A Member 4	*hspa4*	CACTGCGATGCTCCTCACTA	TGTAGAAGCAGGGGACAGAGA
Mesencephalic Astrocyte-Derived Neurotrophic Factor	*manf*	AGATGGAGAGTGTGAAGTCTGTGTG	CAATTGAGTCGCTGTCAAAACTTG
Microtubule-Associated Protein Tau A	*mapta*	TCGTCACAAACCAGGTGGAG	GCTCACGGAACGTCAGTTTG
Microtubule-Associated Protein Tau B	*maptB*	AAGATCGGCTCCACTGAGAACC	GATCCAACCTTTGACTGGGCTT
Myelin Basic Protein	*mbp*	AATCAGCAGGTTCTTCGGAGGAGA	AAGAAATGCACGACAGGGTTGACG
Mitochondrially Encoded Cytochrome C Oxidase Subunit I	*mt-co1*	ACTTAGCCAACCAGGAGCAC	GGGTGGAAGAAGTCAGAAGC
Nuclear factor erythroid 2-related factor 2	*nrf2*	AAGCAGACGGAGGAGGAG	GGAGGTGTTCAGGCAAGG
Nestin	*nes*	ATGCTGGAGAAACATGCCATGCAG	AGGGTGTTTACTTGGGCCTGAAGA
NAD(P)H Quinone Dehydrogenase 1	*nqo1*	TCTTTTTGCAGGATCCGTTCACTCATCT ATCCTGGTGC	TTCTAGAGGCTCGAGATGTATTGGTAGCTACGCTGGATC

Tumor Protein p53	*p53*	GGGCAATCAGCGAGCAAA	ACTGACCTTCCTGAGTCTCCA
Ribosomal Protein S18	*rps18*	TCGCTAGTTGGCATCGTTTATG	CGGAGGTTCGAAGACGATCA
Sonic Hedgehog Signaling Molecule	*shh*	AGACCGAGACTCCACGACGC	TGCAGTCACTGGTGCGAACG
Superoxide Dismutase 1	*sod1 (Cu/Zn SOD)*	CAACACAAACGGCTGCATCA	TTTGCAACACCACTGGCATC
Superoxide Dismutase 2	*sod2 (Mn SOD)*	AGCGTGACTTTGGCTCATTT	ATGAGACCTGTGGTCCCTTG
Alpha 1-tubulin	*α1-tubulin*	AATCACCAATGCTTGCTTCGAGCC	TTCACGTCTTTGGGTACCACGTCA
